# Ethnobotanical, Phytochemical, and Pharmacological Properties of the Subfamily Nepetoideae (Lamiaceae) in Inflammatory Diseases

**DOI:** 10.3390/plants12213752

**Published:** 2023-11-02

**Authors:** Nancy Ortiz-Mendoza, Martha Juana Martínez-Gordillo, Emmanuel Martínez-Ambriz, Francisco Alberto Basurto-Peña, María Eva González-Trujano, Eva Aguirre-Hernández

**Affiliations:** 1 Laboratorio de Productos Naturales, Departamento de Ecología y Recursos Naturales, Facultad de Ciencias, Universidad Nacional Autónoma de México, Mexico City 04510, Mexico; nancy_om@ciencias.unam.mx; 2 Posgrado en Ciencias Biológicas, Unidad de Posgrado, Ciudad Universitaria Coyoacán, Edificio D, 1° Piso, Circuito de Posgrados, Mexico City 04510, Mexico; 3 Departamento de Biología Comparada, Herbario de la Facultad de Ciencias, Universidad Nacional Autónoma de México, Mexico City 04510, Mexico; 4 Instituto de Ecología, A.C., Red de Biodiversidad y Sistemática, Xalapa 91073, Veracruz, Mexico; emmanuel.martinez@inecol.mx; 5 Jardín Botánico, Instituto de Biología, Universidad Nacional Autónoma de México, Mexico City 04510, Mexico; abasurto@ib.unam.mx; 6 Laboratorio de Neurofarmacología de Productos Naturales, Dirección de Investigaciones en Neurociencias, Instituto Nacional de Psiquiatría Ramón de la Fuente Muñiz, Mexico City 14370, Mexico; evag@imp.edu.mx

**Keywords:** inflammation, Lamiaceae, Nepetoideae, secondary metabolites

## Abstract

Nepetoideae is the most diverse subfamily of Lamiaceae, and some species are well known for their culinary and medicinal uses. In recent years, there has been growing interest in the therapeutic properties of the species of this group regarding inflammatory illnesses. This study aims to collect information on traditional uses through ethnobotanical, pharmacological, and phytochemical information of the subfamily Nepetoideae related to inflammatory diseases. UNAM electronic resources were used to obtain the information. The analysis of the most relevant literature was compiled and organised in tables. From this, about 106 species of the subfamily are traditionally recognised to alleviate chronic pain associated with inflammation. Pharmacological studies have been carried out in vitro and in vivo on approximately 308 species belonging to the genera *Salvia*, *Ocimum*, *Thymus*, *Mentha*, *Origanum*, *Lavandula*, and *Melissa*. Phytochemical and pharmacological evaluations have been performed and mostly prepared as essential oil or high polarity extracts, whose secondary metabolites are mainly of a phenolic nature. Other interesting and explored metabolites are diterpenes from the abietane, clerodane, and kaurane type; however, they have only been described in some species of the genera *Salvia* and *Isodon*. This review reveals that the Nepetoideae subfamily is an important source for therapeutics of the inflammatory process.

## 1. Introduction

**Figure 1:**
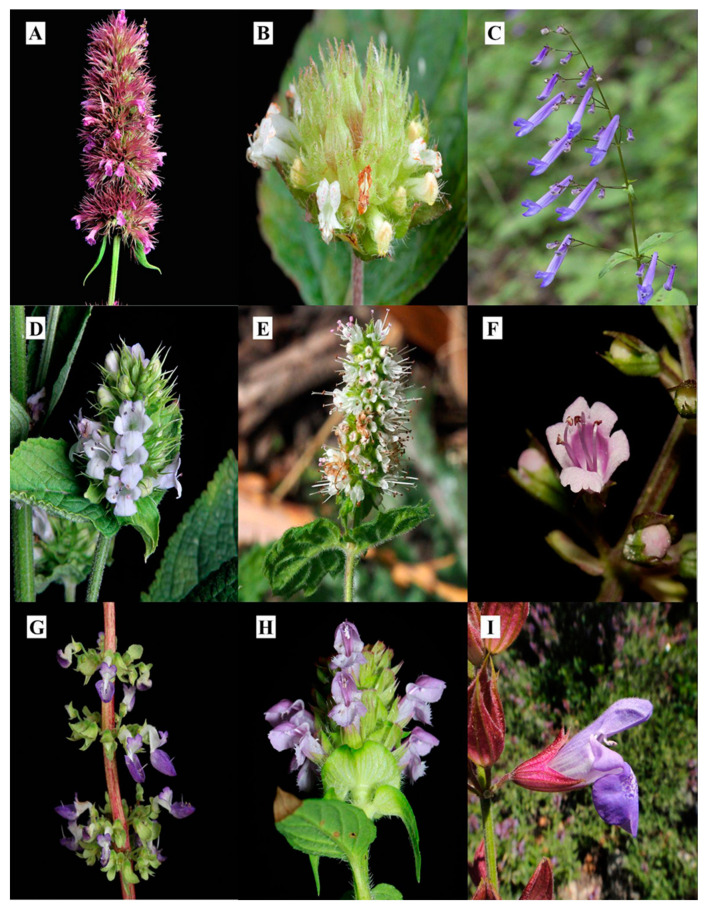
Selected species of the subfamily Nepetoideae (Lamiaceae) that have been studied for their anti-inflammatory properties. (**A**) *Agastache mexicana*, (**B**) *Hyptis atrorubens*, (**C**) *Isodon effusus*, (**D**) *Lepechinia caulescens*, (**E**) *Mentha spicata*, (**F**) *Ocimum carnosum*, (**G**) *Plectranthus scutellarioides*, (**H**) *Prunella vulgaris*, (**I**) *Salvia officinalis*. Photo credits: Canek Ledesma (**A**,**D**); Jonathan Amith (**B**,**H**); Toshihiro Nagata (**C**); Fred Melgert (**E**); Miriam Jiménez (**F**); Francisco Osbel (**G**); Christian Berg (**I**).

Within the Angiosperms, the Lamiaceae is the sixth most diverse family worldwide. It is divided into 12 subfamilies, where Nepetoideae stands out due to its diversity. Based on molecular and morphological data, it is a monophyletic subfamily, with approximately 123 genera and 3685 species [1,2]. Morphologically, Nepetoideae is characterised by herbaceous, shrubby, or rarely arboreal individuals; they are generally aromatic as they contain a diversity of terpenoids and the presence of the well-known rosmarinic acid [3,4]. Many species belonging to genera of this subfamily are known for their medicinal and culinary uses as condiments, e.g., *Ocimum*, *Origanum*, *Thymus*, *Salvia*, *Melissa*, and *Lavandula*, among others [5,6,7,8]. Other genera of economic importance are *Perilla* and *Prunella*. *Perilla frutescens* is used as a condiment in oriental cuisine and *Prunella vulgaris* is outstanding for its medicinal, culinary, and ornamental uses, respectively [9,10]. On the other hand, the need for alternative and complementary therapies to treat several health problems around the world is continuous. Relevant areas looking for relevant information on these issues include agriculture and biological sciences in the fields of biochemistry, genetics, and molecular biology, as well as in the fields of pharmacology, toxicology, and pharmaceuticals. Thus, certain countries, such as India and China, have contributed almost 25% of the publications in this research area [11,12]. An interesting topic is inflammation, which is a complex set of sequential tissue changes to eliminate the initial cause of cellular injury; it presents various signs, such as local redness, swelling, pain, heat, and loss of function [13,14]. In the fields of pharmacology, toxicology, and pharmaceuticals, therapies to treat inflammation are frequently mentioned, as the inflammatory process is implicated in a wide variety of physical and mental diseases that dominate current morbidity and mortality worldwide [15,16]. At the molecular level, several chemical substances are implicated in reducing or increasing inflammation. Frequently, certain cellular stimuli trigger inflammatory processes through the release of proinflammatory cytokines and chemokines (TNF, IL-1β, IL-22, IL-17, IFN-γ, among others). Cytokines can activate endothelial cells and acute phase protein synthesis and recruit immune system cells that play a crucial role in phagocytosis and pathogen destruction. Once the cells of the immune system are activated, they release cytokines and stimulate the release of prostaglandins that mediate a series of signs and symptoms of this process [17]. Finally, for the closure of the inflammatory response, cytokines, such as IL-10, IL-37, and TGF-β, can largely suppress this mechanism and return to homeostasis; if the anti-inflammatory response is not very pronounced, it can lead to vulnerability [18,19]. Currently, glucocorticoids and non-steroidal anti-inflammatory drugs are the most commonly used therapies in the clinic to treat problems related to inflammation. These drugs provide pain relief to the patient. The main mechanism of action of these drugs is the inhibition of prostaglandins and other proteins released in the inflammatory process [20]. However, there is enough evidence demonstrating the risk of myocardial infarction, heart failure, kidney failure, and arterial hypertension as part of the common adverse effects [21,22]. Due to these inconveniences, research on medicinal plants is relevant because they are a large potential reservoir of active metabolites with fewer harmful effects. The genera and species of the subfamily Nepetoideae (Figure 1) are interesting and important for their useful properties. Therefore, this review aims to compile some of the medicinal uses attributed to species belonging to the subfamily Nepetoideae, as well as the pharmacological properties and phytochemical analysis to isolate bioactive metabolites responsible for the anti-inflammatory activity and mechanism of action, reinforcing them as a potential therapy to improve health.

## 2. Results and Discussion

### 2.1. Ethnobotanical Information

**Figure 2:**
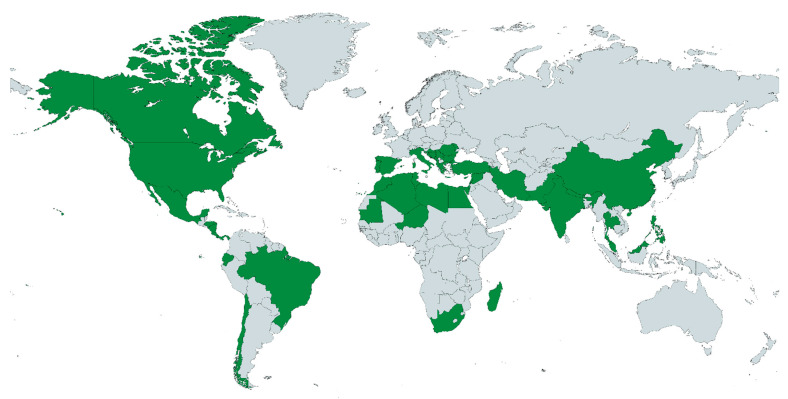
Countries included in reports of traditional uses related to the inflammatory process of species of the subfamily Nepetoideae are shown in green.

According to the Encyclopedic Dictionary of Traditional Mexican Medicine, inflammation is a synonym for swelling; it is caused by blows, infections, rheumatism, local pain, heat, and redness, among others. In traditional medicine, inflammation is almost always understood as a sign or symptom present in various diseases and rarely as a condition [23]. Thus, the search for literature describing traditional uses related to the inflammatory process include 33 genera and 106 species of the Nepetoideae subfamily worldwide (Table S1). Regions where they are used include Chile, Brazil, Ecuador, Nicaragua, Panama, Costa Rica, Mexico, USA, and Canada in the American continent [24,25,26,27,28,29,30,31], Spain, Greece, Italy, Turkey, Algeria, Libya, Morocco, Mauritania, Tunisia, and Israel from the Mediterranean Sea, and China, India, Nepal, and Pakistan from the Himalayan region [32,33,34,35,36,37,38,39,40]. Other regions include the Philippines, Malaysia, and Thailand from Southeast Asia, and some islands, such as Monserrat, Samoa, and Madagascar [41,42,43,44,45,46] (Figure 2).

**Figure 3:**
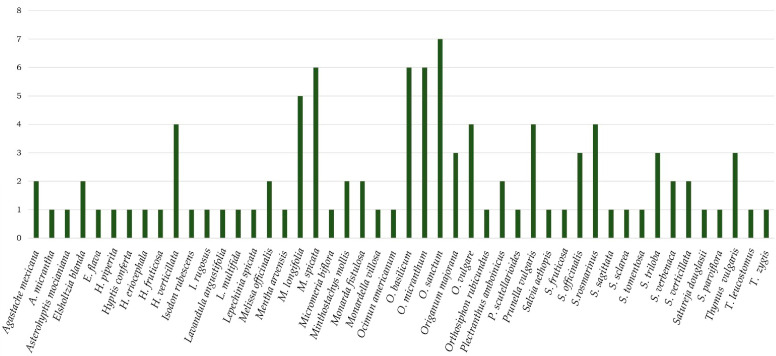
Number of mentions of some species belonging to the Nepetoideae subfamily for traditional uses associated with inflammatory processes.

The genera with the most reports of traditional uses for inflammation-related conditions are *Mentha*, *Ocimum*, and *Salvia*. The most mentioned species was *Mentha longifolia* (L.) Hudson, *M. spicata* L., *Ocimum sanctum* L., and *Salvia rosmarinus* L. (Figure 3) (Table S1). *M. longifolia* and *M. spicata* are used for inflammation of the throat, gums, and eyes. *O. sanctum* is used in India to treat everything from constipated flu to chronic pain. Meanwhile, *S. rosmarinus* is used against neuritis, rheumatism, and uterine fibrosis [33,36,37,47]. The genera distributed in the American continent are *Agastache*, *Cunila*, *Hedeoma*, and *Hyptis*, all of which are used to treat various conditions, such as gastrointestinal pain and inflammation, gingivitis, blows, rheumatism, wounds, earaches, bones, and colic [23,48,49].

*Elsholtzia*, *Isodon*, and *Orthosiphon* are widely used in Oriental medicine for skin conditions (e.g., wounds and psoriasis), the respiratory tract (tonsillitis and pharyngitis), and colic pain, respectively [50,51,52]. Some genera are part of the culinary culture of many countries, such as *Mentha*, *Ocimum*, *Origanum*, and *Thymus*, where they are used as condiments and for gastrointestinal ailments (diarrhea, dysentery, and colic), the respiratory system (asthma, colds, catarrh, and bronchitis), to reduce fever, to reduce swelling, and for some chronic issues, such as uterine fibrosis [32,53,54,55]. A highly cited genus is *Salvia*, known both in Mediterranean cuisine for its culinary use and in traditional medicine in different countries including Mexico, China, and India. Salvias are used to treat wounds, pain, infections, fever, rheumatism, uterine fibrosis, and burns, among others [31,34,52]. *Asterohyptis mociniana* (Benth.) Epling and *Isodon rubescens* (Hemsl.) H. Hara are used to treat gastroenteritis, whereas *Clinopodium brownei* Kuntze and *Plectranthus scutellarioides* Blume are used for swelling. In the cases of *Minthostachys mollis* (Kunth) Griseb. and *Monarda fistulosa* L., they have been reported for bronchitis, while *Calamintha acinos* Man. and *Lepechinia spicata* Willd. have been reported for lung problems [23,24,31,35,52,56,57,58]. All of these genera produce a considerable number of secondary metabolites, which alone or in synergy have beneficial biological properties for human health, making excellent functional foods, which could subsequently be developed as nutraceuticals [59].

### 2.2. Pharmacology

**Figure 4:**
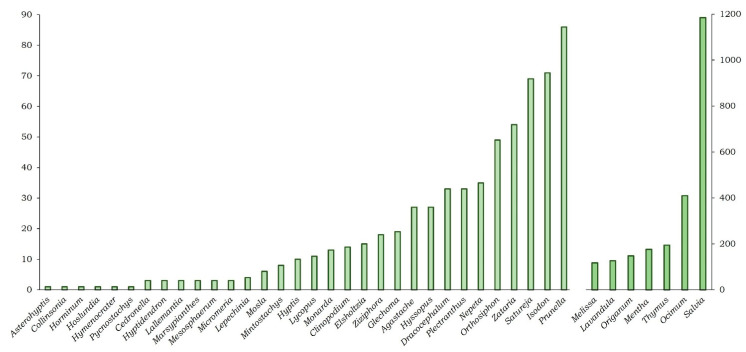
Number of articles related to anti-inflammatory activity indexed in Scopus for the genera of the subfamily Nepetoideae. The number of items for the genera *Melissa*, *Lavandula*, *Origanum*, *Mentha*, *Thymus*, *Ocimum*, and *Salvia* is shown on the right-hand axis, and on the left-hand axis are all of the remaining genera.

**Figure 5:**
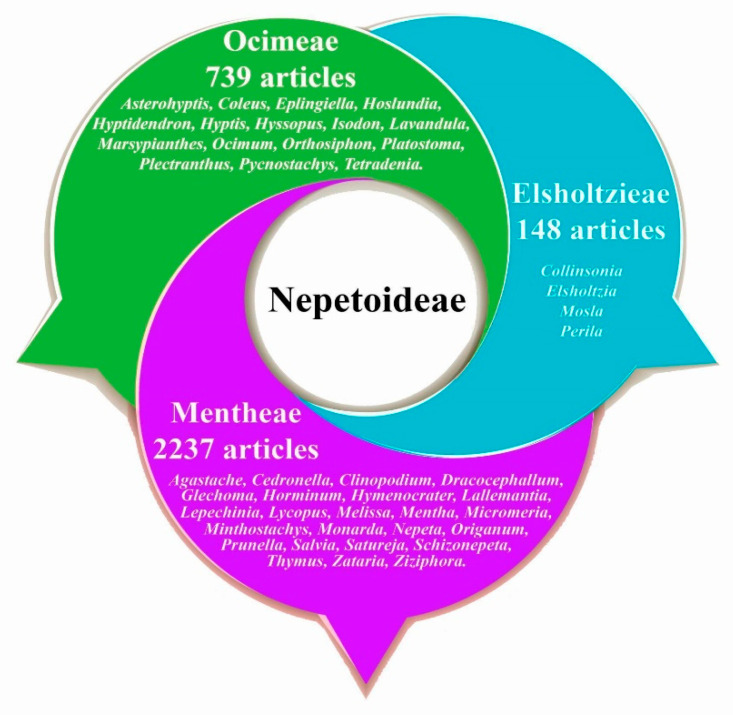
Number of articles related to anti-inflammatory activity indexed in Scopus in relation to the tribes of the subfamily Nepetoideae, including the genus for each tribe.

In general, it is known that polar preparations are the most commonly used medicinal plants in folk medicine, and they are included in the most representative studies explored in bioassays and phytochemical studies [60]. In the case of the Nepetoideae subfamily species, preparations commonly used in folk medicine are produced through infusion or decoction of the whole plant or other independent parts (Table S1). However, not only polar extractions but also non-polar extracts using different organic solvents and the essential oil have been reported to identify and isolate bioactive metabolites with anti-inflammatory activity. A total of 831 species of the Nepetoideae subfamily were obtained with at least one article indexed in any category in the Scopus database. From the 831 species, a total of 39 genera and 308 species were reported in 3124 articles, all of them associated with the anti-inflammatory activity (Table S2). In this regard, the genera with the most publications (100–1400) were *Salvia*, *Ocimum*, *Thymus*, *Mentha*, *Origanum*, *Lavandula*, and *Melissa* (Figure 4). It was followed by 17 genera with 10 to 100 articles (Figure 5), of which 12 belong to the Menthae tribe (*Prunella*, *Satureja*, *Zataria*, *Nepeta*, *Dracocephalum*, *Hyssopus*, *Agastache*, *Glechoma*, *Ziziphora*, *Clinopodium*, *Monarda*, and *Lycopus*), 4 belong to the Ocimeae tribe (*Isodon*, *Orthosiphon*, *Plectrathus*, and *Hyptis*), and only 1 is from the Elsholtziae tribe (*Elsholtzia*) (Figure 4).

**Figure 6:**
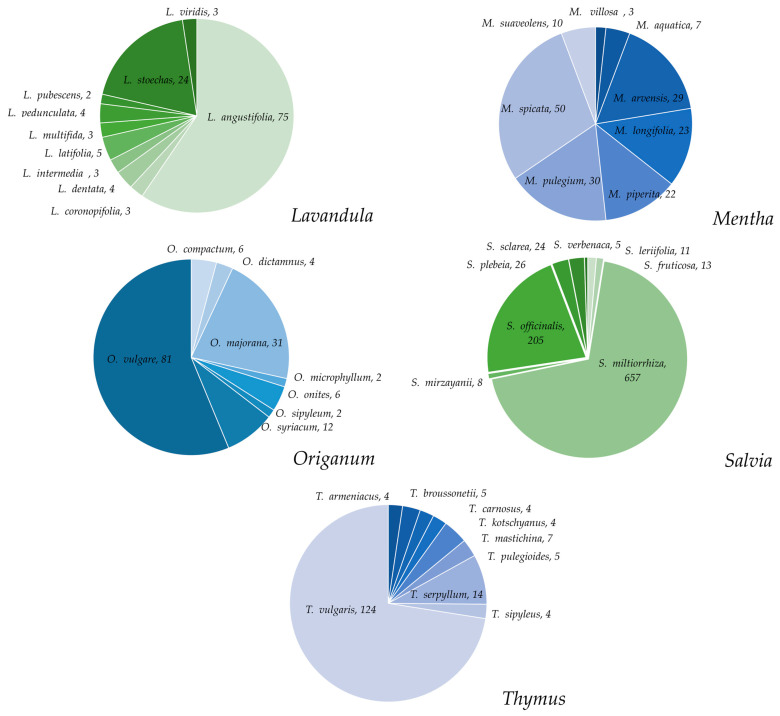
Species of the genera *Lavandula*, *Mentha*, *Origanum*, *Salvia*, and *Thymus* with the highest number of publications related to anti-inflammatory processes.

Within the genus *Salvia*, there are approximately 111 species reported in relation to the inflammatory process. The most studied species are *S. miltiorrhiza* Bunge and *S. officinalis* L. (Figure 6). For *S. miltiorrhiza*, its properties have been reported using the root extracts, where a number of isolated diterpenes called tanshinones were also identified as responsible for the effect on the reduction of proinflammatory cytokines in in vitro assays [61]. The reduction of fibrosis in the liver, heart, lung, and kidney was reported in in vivo models, with improvement against allergies, asthma, and rhinitis using clinical tests [62,63]. Anti-inflammatory effects of *S. officinalis* have been described in in vivo tests prepared as organic extracts of different polarity and aqueous extracts, in which the main component was rosmarinic acid [64]. *S. dolomitica* Codd, *S. frigida* L., *S. nipponica* Miq., *S. petrophilla* G. X. Hu, E. D. Liu, and Yan Liu, *S. plebeia* R. Br., and *S. sclareoides* Brot. reduced the enzymatic activity of elastase and inflammatory mediator molecules, such as nitric oxide (NO), IL-4, IL-13, IL-5, TNF-α, cyclooxygenase (COX)-2, and prostaglandine PGE2 in RAW 264.7 macrophages induced with LPS in organic extracts of different polarities and aqueous extracts [65,66,67,68,69,70]. The polar extracts of *S. chudei* Batt. and Trab., *S. fruticosa* Mill., *S. leriifolia* Benth., *S. macilenta* Boiss., *S. sclarea* L., *S. transsylvanica* (Schur ex Griseb. and Schenk) Schur, and *S. virgata* Ortega demonstrated anti-inflammatory activity in the carrageenan-induced edema model in rats at a dose range of 250–1500 mg/kg. Finally, the acetone extract and the methanol extract of *S. aegyptiaca* L. and *S. moorcroftiana* Wall. ex Benth., respectively, showed antipyretic effects in murine models of hyperthermia (Table S2) [71,72,73,74,75,76,77,78]. After the genus *Salvia*, the species *Ocimum tenuiflorum* L. (syn. *Ocimum sanctum* L.), *Thymus vulgaris* L., *Mentha spicata* L., and *Origanum vulgare* L. are the most studied of their respective genera (Figure 6). The organic and aqueous extracts of *O. tenuiflorum* showed positive effects on the epithelialization and a reduction in the induced edema in murine models. Likewise, phenolic compounds isolated from this species, including rosmarinic acid, decreased the concentration of COX-1 in in vitro models [79,80]. The aerial part of *T. vulgaris* increased the activity of antioxidant enzymes in models of renal and hepatic dysfunction in rabbits, and it also had an effect against cholestasis, chronic hepatitis, and liver fibrosis in clinic studies [81]. Both the organic and aqueous extracts of *M. spicata* decreased edema, granulomas, and mucositis induced in murine models [82]. The essential oil of *O. vulgare* produced significant effects on the proinflammatory cytokines and chemokines [83]. *Lavandula angustifolia* Mill. and *Melissa officinalis* L. have been also explored for their anti-inflammatory effects. Regarding the genus *Lavandula*, several species have been evaluated, expect for *Melissa*, where only *M. officinalis* has been described. With respect to *L. angustifolia*, both the essential oil and the polyphenolic fraction reduced the levels of proinflammatory cytokines in murine models of induced edema, ischemia, chronic inflammatory pain, and sepsis [84,85,86,87,88,89,90,91]. Anti-inflammatory effects of *M. officinalis* were corroborated in a model of edema induced with carrageenan in a murine model, mainly from its ethanol and aqueous extract [92,93,94,95]. The genera explored for their anti-inflammatory effects that belong to the Menthae tribe are shown in Figure 5. In the case of *Prunella*, only *P. vulgaris* L. has been evaluated in this affection. The essential oil, polar extracts, as well as the isolated compounds from the inflorescences, such as the diterpene prunela diterpenol A and the phenolic compounds prunelanate A and prunelanate B, presented activity in the regulation of proinflammatory cytokines in in vitro tests [96,97,98,99]. Triterpenes, such as 2α,3α,23-trihydroxyursa-12,20(30)-dien-28-oic acid, β-amyrin, and eusapic acid, produced effects on histamine suppression in in vitro studies [100]. For the genera *Satureja*, *Zataria*, *Nepeta*, *Glechoma*, and *Lycopus*, few species have been studied in murine models using polar extracts of *Satureja montana* L. and *Nepeta dschuparensis* Bornm, which decreased IL-1β levels in the model of traumatic brain injury and artery occlusion infarction, respectively. *Glechoma longituba* (Nakai) Kuprian attenuated proinflammatory gene expression in retinas exposed to bright light, *Lycopus lucidus* Turcz. ex Benth. inhibited histamine release in allergy models, and *Zataria multiflora* Boiss. improved levels of proinflammatory cytokines in asthma models [101,102,103,104,105]. From *Agastache mexicana* (Kunth) Lint and Epling and *Dracocephalum moldavica* L., which also belong to the Mentheae tribe, the glycosylated flavonoid tilianin has been isolated, where a reduction in the mRNA expression of proinflammatory cytokines was observed in in vivo and in vitro models [106,107]. Likewise, some little-known diterpenes have been isolated from *D. moldavica*, such as dracocephalumoids A-E, uncinatone, trichotomone F, and caryopterisoid C, which suppressed TNF-α, IL-1β, and NO in RAW 264.7 macrophages induced with LPS [108]. Finally, the aqueous extracts of *Hyssopus officinalis* L. and *Ziziphora clinopodioides* Lam. were used for the synthesis of Zn and Fe nanoparticles, demonstrating their anti-inflammatory effect in murine models of carrageenan-induced edema and hemolytic anemia, respectively [109,110]. Diterpenoid-type compounds isolated from the aqueous extracts of species of the Ocimeae tribe have presented anti-inflammatory activity in both in vitro and in vivo models, such as the case of orthosiphol A and B obtained from *Orthosiphon stamineus* Benth., parvifloron D from *Plectranthus ecklonii* Benth., as well as suaveolol and methyl suveolate from *Hyptis suaveolens* (L.) Poit. [111,112,113]. Regarding the genus *Isodon*, such as *I. adenanthus* (Diels) Kudô, *I. enanderianus* (Hand.-Mazz.) H.W. Li, *I. eriocalyx* (Dunn) Kudô, *I. henryi* (Hemsl.) Kudô, *I. leucophyllus* (Dunn) Kudô, *I. rugosiformis* (Hand.-Mazz.) H. Hara, *I. scoparius* C.Y. Wu and H.W. Li) H. Hara, and *I. rubescens* (Hemsl.) H. Hara, a significant amount of kaurane-type diterpenes have been isolated as responsible for the activity on proinflammatory cytokines both in RAW 264.7 macrophages induced with LPS as well as in a variety of models, such as in murine infections of encephalomyelitis, prostatitis, peritonitis, gouty arthritis, and type II diabetes [114,115,116,117,118,119,120]. In the case of *I. ternifolius* (D.Don) Kudô, the lignans ternifoliuslignans A, B, C, D, and E and the glycosylated phenylethanoid 3-carboxy-6,7-dihydroxy-1-(3′,4′-dihydroxyphenyl)-naphthalene were characterized to suppress activity of PGE2 and TNF-α in macrophages induced with LPS [121]. *I. eriocalyx*, from which endophytic fungus *Phomopsis sp.* was first isolated, allowed the isolation of the phomopchalasins A, B, and C with NO inhibitory activity in in vitro assays [122]. Species with few studies reported in the literature were *Cedronella canariensis* (L.) Webb and Berthel., *Hoslundia opposita* Vahl, and *Micromeria biflora* (Buch. Ham. ex D.Don) Benth., prepared as chloroform extracts of flowers, root, and aerial part, respectively, which demonstrated anti-inflammatory effects in the murine model of edema induced with carrageenan [123,124,125]. Polar extracts of *Asterohyptis stellulata* (Benth.) Epling promoted skin regeneration in CD-1 mice. *Micromeria croatica* (Pers.) reduced the expression of proinflammatory cytokines in models of liver injury, and *Mosla chinensis* Maxim. and *M. scabra* attenuated levels of inflammatory mediators in models of ulcerative colitis [126,127,128,129]. From the petroleum ether extracts of *Horminum pyrenaicum* L., abietane-type diterpenes were isolated that suppressed the activity in immunometabolic pathways related to inflammatory processes in murine models [130] (See Table 1). All of this information together demonstrates that different classes of natural compounds are investigated for their anti-inflammatory potential properties in the species of the Nepetoideae subfamily. The chemical structure diversity found in natural products has served as an attractive approach in searching for relevant anti-inflammatory drugs, as it can be possible from this subfamily. Thus, the chemical structure diversity is not a factor in producing similar biological activity; however, the bioactivity can be improved by modifying the structure [131,132]. Due to this, it is important to notice that natural chemical compounds show a wide spectrum of activities and interaction in several molecular targets responsible for the anti-inflammatory activity of these species because it can be more than one at the same time. As an example, several compounds possessing antioxidant properties can prevent and/or reduce oxidative stress, which is relevant in inflammation and neurodegenerative diseases. The activity–structure relationship of several common polyphenols in plants, such as gallic acid reported in some species of the Nepetoideae subfamily, have demonstrated that the higher the number of phenolic hydroxyl groups, the stronger the antioxidant activity that regulates inflammatory mechanisms and pathways [132]. The interaction of some flavonoids in several targets at the same time, such as quercetin derivatives, has been reported using antagonists of inhibitory receptors, such as endogenous opioids, and those of serotoninergic and/or dopaminergic neurotransmission involved in neurodegenerative diseases have been explored by using predictive molecular docking, too, in order to support their important role at peripheral and central levels [131]. Some natural products from a terpenoid nature, such as sclareol, which are also found in species of this subfamily, produced their anti-inflammatory activity by inhibiting not only NO production but also the expression of iNOS and COX-2 proteins, as well as in the MAPK signaling pathway [63]. Meanwhile, tanshinone II activity has been supported by regulating the CCNA2-CDK2 complex and AURKA/PLK1 pathways [133]. Further structure activity relationship studies are encouraged for metabolites found in the Nepetoideae subfamily species, as in other plants, to identify not only the chemical compounds but also their mechanisms of action involved in their potential biological activities as anti-inflammatory therapy to improve health.

### 2.3. Phytochemistry

**Figure 7:**
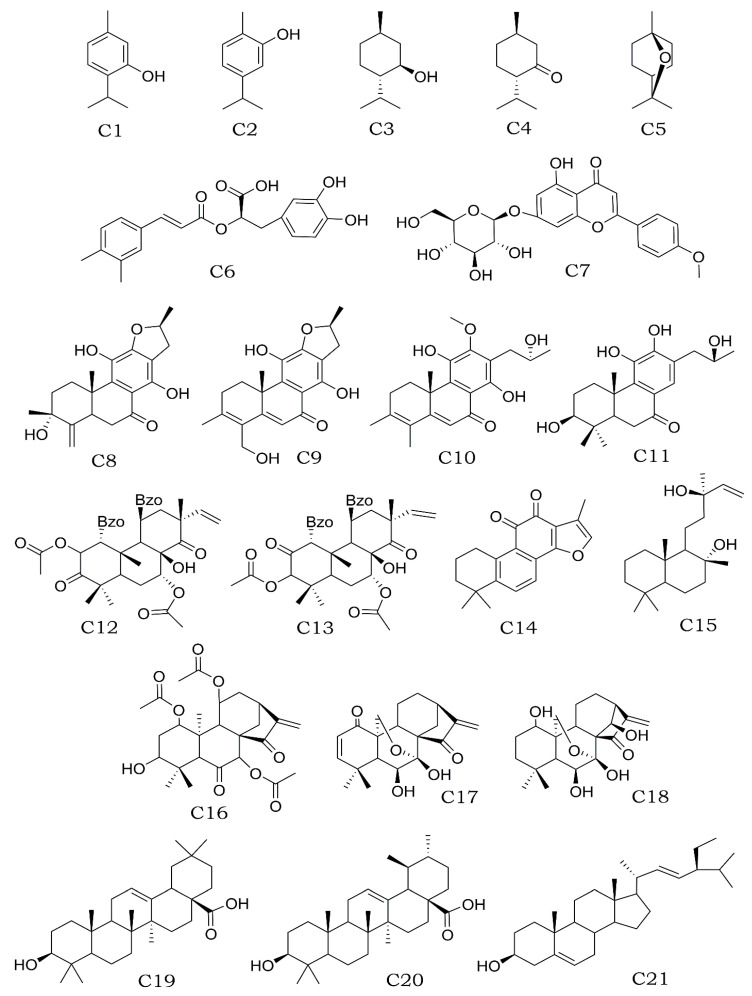
Representative non-polar (C1–C5 and C19–C21) and polar (C6–C18) secondary metabolites with anti-inflammatory activity isolated from species of the subfamily Nepetoideae. Thymol (C1), carvacrol (C2), menthol (C3), menthone (C4), eucalyptol (C5), rosmarinic acid (C6), tilianin (C7), dracocephalmoids A to D (C8–C11), orthosiphol A and B (C12–C13), tanshinone IIA (C14), sclareol (C15), adenanthine (C16), eriocalyxin B (C17), oridonine (C18), oleanolic acid (19), ursolic acid (20), and stigmasterol (21).

Chemical secondary metabolites have been identified and isolated using dissolvents of different polarity through several techniques of extraction [194]. Therefore, phytochemical techniques have allowed for the determination of the main components of some species of the subfamily Nepetoideae. This section generally describes some of the compounds commonly identified in the subfamily (mono- and tri-terpenoids, phenolic acids, and flavonoids). It also mentions the case of clerodane and kaurano-type diterpenes, which have different biological activities (see Section 2.2) and have only been isolated in certain genera, such as *Salvia* and *Isodon*. Table S3 lists the species studied phytochemically and the secondary metabolites isolated from them. Monoterpenes have been the most identified, such as thymol (C1) and carvacrol (C2), which are important components of the essential oils of *Collinsonia*, *Monarda*, *Ocimum*, *Origanum*, *Satureja*, *Thymbra*, *Thymus*, and *Zataria* [83,101,195,196,197,198,199,200]. On the contrary, there are exclusive molecules of certain genera, as exemplified by *Mentha*, where menthol (C3), menthone (C4), and eucalyptol (C5) predominate. In the case of *Lavandula* and *Melissa*, linalool and nerol have been identified as the most important constituents, respectively [94,201,202]. Different phenolic acids have been reported throughout the subfamily, such as caffeic acid, ferulic acid, gallic acid, chlorogenic acid, sinapic acid, and a high presence of rosmarinic acid (C6) [203,204,205,206,207,208]. Similarly, the presence of flavonoids and their glycosylated derivatives, such as quercetin, luteolin, and naringenin, have been very common [126,198,200,201,202]. In contrast, the presence of the flavonoid tilianin (C7) has been reported only in *Agastache* and *Dracocephalum* (Figure 7) [104,105]. It is important to notice that diterpenes are the chemical group less explored throughout the subfamily. The species of *Dracocephalum taliense* Forrest and *Horminum pyrenaicum* L. included the abietanes sugiol, ferruginol, cryptojaponol, and totarol isolated from roots. Dracocephalumoids A, B, C, and D (C8–C11), and orthosiphol A and B (C12–C13) were purified from *Dracocephalum moldavica* L. and *Orthosiphon stamineus* Benth. [108,111,209]. Other genera, such as *Salvia* and *Isodon*, stand out for the great diversity of diterpenes that have been isolated and identified in several of their species. In the case of *Salvia*, terpenes of abietane, clerodane, labdane, and pimarane-type structures have been described. Some examples are tanshinone IIA (C14) and sclareol (C15) isolated from *S. miltiorrhiza* and *S. sclarea*, respectively [61,62,63,64,65,66,67,68,69,70,71]. In the case of the genus *Isodon*, compounds with a Kaurano-type skeleton predominated, and some common cases among the species were adenanthine (C16), eriocalyxin B (C17), and oridonine (C18) [114,117,169]. Finally, the presence of triterpenes, such as oleanolic acid (19), ursolic acid (20), stigmasterol (21), and their derivatives, was very common in the subfamily Nepetoideae (Figure 7) [210,211,212]. Chemical structures of the representative bioactive metabolites are included in Figure 7.

## 3. Materials and Methods

The species selected for this review, without including Mexican sage (previously reported in ref. [213]), were obtained from the Taxonomy Browser of the National Center for Biotechnology Information [214]. Thus, traditional uses were explored using the Economic Botany database and the books available at the Institute of Biology, UNAM. Then, pharmacological and phytochemistry properties were searched using the Scopus database. The literature research covers a period from the past to December 2022. The optimization of this search was made by developing a script based on the Python programming language through its interface 3.11.1 (python.org) and using the Application Programming Interface (API) provided by Scopus (dev.elsevier.com). For the development of the script, the following elements were used: (1) list of species belonging to the Nepetoideae subfamily, (2) SCOPUS website to access its database, (3) access credentials to the database (API-Key), (4) parameters, search algorithm, and information processing, and (5) printing of the results. The search strategy consisted of two phases. In the first, the presence/absence of information on each species of the subfamily Nepetoideae was explored in all of the indexed fields (All fields) in the Scopus database. In the second phase, the species with the presence of information obtained in the first phase were used, and the search was carried out with the following parameters: “Genus species” AND Anti-inflammatory OR Antiinflammatory (e.g., “*Zataria multiflora*” AND anti-inflammatory OR antiinflammatory, delimited to the indexed fields “Article title-Abstract-Keywords.” The articles of the species with information more related to anti-inflammatory processes and their phytochemistry were downloaded and analyzed. The results of this search were organized in tables and graphs, as above.

## 4. Conclusions

In this review, a total of 831 species of the subfamily Nepetoideae were obtained with at least one article indexed in any category based on a search in the Scopus database. From the 831 species, a total of 39 genera and 308 species were reported in 3124 articles, all of them associated with anti-inflammatory activity. Thus, traditional uses of the Nepetoideae subfamily species are reinforced by botanical, pharmacological, and phytochemical information described in the literature related to inflammatory illnesses and compiled in this review. The results revealed that this subfamily plays an essential role as another important source of potential anti-inflammatory drugs from natural products of different chemical natures not only at the central level, in part because of their neuroprotective activity, but also in peripheral systems. The most common extracts prepared not only for phytochemical but also for pharmacological investigation in this review were those obtained from a polar nature. Chemical secondary metabolites have been identified and isolated using dissolvents of different polarities through several techniques of extraction. These results together support medicinal use by preparing infusions or decoctions from the whole plants or from different parts of them.

## Figures and Tables

**Table 1:** Bioactive metabolites with anti-inflammatory properties isolated from species of the Nepetoideae (Lamiaceae) subfamily.

Species	Secondary Metabolites with Anti-Inflammatory Activity	References
*Agastache mexicana* Linton and Epling	Ursolic acid and limonene	[134,135]
*Clinopodium polycephalum* (Vaniot) C.Y. Wu and S.J. Hsuan	Saturol I; 3β-22, 25-dihydroxy-tirucalla-7, 23-dieno; maslinic acid; 2α, 3α-dihydroxyolean-12-en-28-oic acid; hederagenin; 2α, 3α-dihydroxyursolic acid; alphitolic acid; rosmarinic acid; and hesperidin	[136]
*Coleus scutellarioides* (L.) Benth.	Quercetin	[137,138]
*Dracocephalum heterophyllum* Benth.	Rosmarinate, luteolin, and diosmetin	[139,140]
*Dracocephalum kotschyi* Boiss.	Apigenin	[141]
*Dracocephalum moldavica* L.	Tilianin, dracocephalumoid A, uncinatone, trichotomone F, and caryopterisoid C	[107,108]
*Dracocephalum palmatum* Stephan ex Willd.	Cosmosiin, cymaroside, and eriodictyol	[142,143,144]
*Dracocephalum rupestre* Hance	Eriodictyol	[145]
*Elsholtzia ciliata* (Thunb.) Hyl.	Luteolin, caffeic acid, vitexin, pedalin, luteolin-7-*O*-β-d-glucopyranoside, apigenin-5-*O*-β-d-glucopyranoside, apigenin-7-*O*-β-d-glucopyranoside, chrysoeriol-7-*O*-β-d-glucopyranoside, 7,3′-methoxy luteolin-6-*O*-β-d-glucopyranoside, 5,6,4′-trihydroxy-7,3′-dimethoxyflavone, 5-hydroxy-6,7-dimethoxyflavone, 4-(*E*)-caffeoyl-L-threonic acid, 4-*O*-(*E*)-p-coumaroyl-L-threonic acid, and α-linolenic acid	[146,147,148,149,150]
*Elsholtzia rugulosa* Hemsl.	Rugulolide A, nepetoidin B, methyl rosmarinate, and syringaresinol	[151]
*Glechoma longituba* (Nakai) Kuprian.	Apigenin-7-diglucoronide	[105]
*Isodon adenanthus* (Diels) Kudô	Adenantin	[117]
*Isodon amethystoides* (Benth.) H. Hara	Glaucocalyxin A	[152]
*Isodon excisus* (Maxim.) Kudô	Inflexinol and inflexin	[153,154]
*Isodon henryi* (Hemsl.) Kudô	Rabdoternin A and lasiodonin	[155]
*Isodon japonicus* (Burm. f.) H. Hara	Kamebanin, kamebakaurin, oridonin, kamebakaurin, effusanin C, and isodojaponin D	[156,157,158,159]
*Isodon melissoides* (Benth.) H.W. Li	Melissoidesin	[160,161]
*Isodon scoparius* C.Y. Wu and H.W. Li) H. Hara	Scopariusol L	[115]
*Isodon serra* (Maxim.) Kudô	Oridonin, serrin F, 14β-hydroxyrabdocoestin A, serrins H-I, enanderianin N, megathyrin B, enmein, isoserrin A- I, and nodosin	[162,163,164,165,166]
*Isodon sculponeatus* (Vaniot) Kudô	Sculponeatin J, sculponin T, sculponeatin C, and sculponins Y	[167,168]
*Isodon ternifolius* (D.Don) Kudô	Ternifoliuslignane A–D and 3-carboxy-6,7-dihydroxy-1-(3′,4′-dihydroxyphenyl)-naftalene	[121]
*Isodon rubescens* (Hemsl.) H. Hara	Oridonin	[169]
*Isodon wikstroemioides* (Hand.-Mazz.) H. Hara	Isowikstroemins A–D, G, H, J, K, and macrocalyxin B	[170,171]
*Mentha cordifolia* Opiz ex Fresen.	Menthol	[172]
*Mentha longifolia* (L.) Huds.	Longifolin A and eucalyptol	[173,174]
*Nepeta cataria* Benth.	Lamiuside A and verbascoside	[175]
*Ocimum kilimandscharicum* Baker ex Gürke	Camphor and mixture of 1,8 cineole/limonene 1:1	[176]
*Perilla frutescens* (L.) Britton	Ursolic acid, corosolic acid, 3-epicorosolic acid, pomolic acid, tormentic acid, hiptadienic acid, oleanolic acid, augustic acid, 3-epimaslinic acid, luteolin, monogalactosildiacilgliceroles, and rosmarinic acid	[177,178,179,180,181,182,183]
*Plectranthus ecklonii* Benth.	Parvifloron D, mixture of β-sitosterol, and stigmasterol (1:1)	[112]
*Plectranthus ornatus* Codd.	(11*R*,13*E*)-11-acetoxyhalima-5,13-dien-15-oic acid; 1α,6β-diacetoxy-8α,13*R*-epoxy-14-labden-11-one; 1,6-di-*O*-acetylforskolin; 1,6-di-*O*-acetyl-9-deoxyforskolin, 1,6-di-*O*-acetylforskolin, and forskolin	[184]
*Prunella vulgaris* L.	2α, 3α, 23-trihydroxyursa-12,20(30)-dien-28-oic acid; β-amyrin, eusapic acid, 2α, 3α-dihydroxyursolic acid, prunelanate A, and pruneladiterpenol A	[96,97,99,100]
*Salvia digitaloides* Diels	Salviatalin A	[185]
*Salvia lachnostachys* Benth.	Fruticulin A	[186]
*Salvia miltiorrhiza* Bunge	Tanshinona IIA	[61]
*Salvia mirzayanii* Rech. f. and Esfand.	Teuclatriol	[187,188]
*Salvia petrophilla* G. X. Hu, E. D. Liu and Yan Liu	Petrofins A-E	[70]
*Salvia plebeia* R. Br.	8-epi-eudebeiolida C, salviplenoide A, luteoloside, nepitrin, homoplantagenin, luteolin, nepetin, hispidulin, and eupatorin	[189,190,191,192]
*Salvia rosmarinus* (L.) J.B. Walker, B.T. Drew and J.G. González (Syn.)	Carnosic acid	[193]

## Supplementary Materials

The following supporting information can be downloaded at: https://www.mdpi.com/article/10.3390/plants12213752/s1, Table S1: Traditional uses of species from the subfamily Nepetoideae (Lamiaceae) related to the inflammatory process, Table S2: Pharmacological evaluation of the extracts related to inflammatory processes in species of the Nepetoideae (Lamiaceae) subfamily, Table S3: Phytochemistry related to inflammatory processes in species of the Nepetoideae (Lamiaceae) subfamily.
