# Supplementary material for: Ethnobotanical, Phytochemical, and Pharmacological Properties of the Subfamily Nepetoideae (Lamiaceae) in Inflammatory Diseases
Source: Plants (Basel). 2023 Nov 2;12(21):3752. doi: 10.3390/plants12213752 (PMC10648697; doi:10.3390/plants12213752)
Supplement: Supplementary file 1 [file plants-12-03752-s001.zip › Table S1.pdf]

**Table S1.** Traditional uses of species from the subfamily Nepetoideae (Lamiaceae) related to inflammatory processes.

| Species | Vernacular name | Country/State/ Region | Uses | Part of the plant, preparation and/or route of administration | Reference |
|---|---|---|---|---|---|
| *Agastache mexicana* Lint. & Epling | Toronjil morado | Mexico, Monterrey | Hypertension | Flowers and leaves, infusion, oral | Nicholson & Arzeni, 1993 |
| | Toronjil | Mexico, Morelos | Stomachache | Flowers and leaves, infusion, oral | Monroy- Ortiz & Monroy, 2006 |
| *Agastache micrantha* (A. Gray) Woot & Standl. | Húpachi | Mexico, Chihuahua | Gastrointestinal, ear diseases, colics, & pain | Whole plant, infusion, oral | Bye, 1986 |
| *Asterohyptis mocianiana* (Benth.) Epling | NR | Mexico, Chiapas | Gastroenteritis | Aerial parts, infusion, oral | De la Cruz-Jimenez et al., 2014 |
| *Calamintha acinos* Man. | NR | Greece, Anakassia | Lung diseases | Leaves, infusion, oral | Brussell, 2004 |
| *Clinopodium brownei* Kuntze | K' ox yax-chel | Mexico, Chiapas, Zinacantán, | Swelling | Leaves, infusion, oral | Breedlove & Laughlin, 1993 |
| *Collinsonia canadensis* L. | NR | Southern US | To heal wounds | Roots, grinding, cutaneous | Core, 1966 |
| *Cunila stachyoides* Mart. & Galeotti | Cordoncillo | Mexico, Morelos | Constipated cold | Branches, infusion, oral | Argueta et al., 1994 |
| *Dracocephalum forrestii* W.W. Smith | Zhiyanggu | China, Tibet region | Antipyretic | NR | Li et al., 2009 |
| *Elsholtzia blanda* Benth. | Luo pie la ga | China, Jiping Miao | Ulcers on the skin | Fresh leaves, grinding, cutaneous | Huai & Pei, 2004 |
| | Ihasilam | Nepal | Wounds | Leaves, grinding, cutaneous | Manandhar, 1991 |
| *Elsholtzia flava* (Benth.) Benth. | Su bi la ci | China | Psoriasis | Leaves grinding in warm water, cutaneous | Huai & Pei, 2004 |
| *Hedeoma drummondii* Benth. | Mal t'eel, tasamak poleyo | Mexico, Huasteca region | Gastrointestinal pain, constipation, & asthma. | Aerial parts, infusion, oral | Alcorn, 1984 |
| *Hedeoma piperita* Benth. | Hierba de Santo Domingo | Mexico, Michoacán | Abdominal inflammation, & gingivitis | Aerial parts, infusion, oral | Argueta et al., 1994 |

| | | | | | |
|---|---|---|---|---|---|
| *Hyptis* Jacq. spp. | Siwáklik | Costa Rica & Panamá | Antipyretic | Leaves, infusion, oral | Hazlett, 1985 |
| *Hyptis conferta* Phol. ex Benth. | Teurisha | Mexico, Nayarit | Anti-inflammatory | Aerial parts, maceration, cutaneous | Gispert & Rodríguez, 1998 |
| *Hyptis eriocephala* Benth. | Yana Tipo | Ecuador | Pneumonia, stomachache, & back pain | NR | Rios et al., 2007 |
| *Hyptis fruticosa* Salzm. ex. Benth. | Alecrim | Brazil | Rheumatism | Leaves, infusion, oral | Voeks, 1996 |
| *Hyptis obtusiflora* C. Presl. ex Benth. | Secreto de indio | Ecuador | Wounds | Leaves juice, cutaneous | Rios et al., 2007 |
| *Hyptis suaveolens* (L.) Poit. | Kalungiking | Philippines | Toothache | Roots, grinding, cutaneous | Cox & Gereau 1988 |
| | Jogbo | Nigeria | Headache | Aerial parts, infusion, internal | Bhat et al., 1990 |
| *Hyptis tomentosa* Poit. | Nochleb buluk ' sat | Mexico, Chiapas, Zinacantán | Colics & diarrhoea | Leaves, infusion, oral | Breedlove & Laughlin, 1993 |
| *Hyptis verticillata* Jacq. | Wála | Honduras | Stomachache | Leaves, infusion, oral | Lentz, 1986 |
| | Baríorúma | Nicaragua | Pain, hypertension, lung infections, & skin inflammation | Aerial parts, infusion o decoction, oral | Coe & Anderson, 1996 |
| | Haraspata, hierba de dolor, wahiwin saika | | Bone pain & body infections | Roots, infusion, oral | Barret, 1994 |
| | Tihtsan ts'ohool | Mexico, Huasteca region | Foot swelling & edema | Leaves, infusion, bath | Alcorn, 1984 |
| *Isodon rubescens* (Hemls.) H. Hara. | Donglingcao Diwan | China | Tonsillitis, pharyngitis, & stomatitis | NR | Chen et al., 2022 |
| *Isodon rugosus* Codd. | Pemar | Pakistan | Stomachache, respiratory, & cardiovascular diseases | Leaves, roots, infusion, oral. | Ajaid et al., 2021 |
| *Lamium orientale* (Fish & C.A. Mey.) E.H.L. Krause | Güzelce | Turkey | Urinary tract inflammation | Aerial parts, infusion | Selvi et al., 2022 |
| | Ostokhodus | Iran | Antiinflammatory | Extract | Naghibi et al., 2005 |

| | | | | | |
|---|---|---|---|---|---|
| *Lavandula angustifolia* Mill. | Algucena | Southern US | Uterine fibrosis | NR | Balick et al., 2000 |
| *Lavandula multifida* L. | Cantueso | Spain, Almeria | Digestive problems | Aerial parts, infusion, oral | Martínez-Lirola et al., 1996 |
| *Lepechinia spicata* Willd. | Brenilla | Mexico, Michoacan | Bronchitis & cold | NR | Argueta et al., 1994 |
| *Marrubium vulgare L.* | Gandna, kouhi, ferasuon, Yol-ghutli | Iran | Antiinflammatory | Whole plant, decoction | Naghibi et al., 2005 |
| | Horehound | South Africa | Antiinflammatory, expectorant and chest infections | Infusion, oral | Bhat & Moskovitz, 2009 |
| *Melissa officinalis* L. | Toronjil | California | Colics in infants | Aerial parts, decoction, oral | Bocek, 1984 |
| | | Ecuador | Colics | Leaves, infusion, oral | Rios et al., 2007 |
| | NR | Greece | Rheumatism and arthritis | NR | Vokou et al., 1993 |
| | Barangbo, Badranjbuyeh | Iran | Anti-inflammatory | Aerial parts, decoction | Naghibi et al., 2005 |
| | Lemon blam | South Africa | Antispasmodic, relaxant | Infusion, oral | Bhat & Moskovitz, 2009 |
| *Mentha* L. spp. | Yerba buena | Southern US | Uterine fibrosis | NR | Balick et al., 2000 |
| | Hortelã branco | Brazil | Cold & flu | Leaves, infusion, oral | Voeks, 1996 |
| | Yerba buena | Mexico, Huasteca region | Stomachache | Leaves, infusion, oral | Alcorn, 1984 |
| *Mentha arvensis* L. | Podina | India, Haryana | Severe stomachache | Leaves, infusion, oral | Lal & Yadav, 1982 |
| *Mentha canadensis* L. | Hierba buena | Mexico, Morelos | Asthma | Branches, infusion, oral | Monroy-Ortiz & Monroy, 2006 |
| *Mentha longifolia* (L.) Hudson | Wild diosmos, wild mentha | Greece, Katohori | Digestive system disorders | Leaves and branches, infusion, oral | Brussell, 2004 |
| | Tulasipate | Nepal | Sore throat | Leaves, decoction, oral | Bhattarai, 1992 |
| | Tüylü nane | Turkey, Anatolia | Tachycardia & cold | Flowers, decoction, oral | Özgökçe & Özçelik, 2004 |
| | Eşeknanesi | Turkey | Stomachache | Aerial parts, decoction, oral | Fujita et al., 1995 |

| | | | | | |
|---|---|---|---|---|---|
| | Inixina, Peppermint | South Africa | Cold, asthma | Leaves, decoction | Asowata-Ayodele et al., 2016 |
| *Mentha piperita* L. | Peppermint | Greece, Kala Nera | Digestive system disorders, cold, & flu | Aerial parts, infusion, oral | Brussell, 2004 |
| | Menta, bálsamo xiw | Mexico, Yucatán | Diarrhoea & dysentery | Aerial parts | Ankli et al., 1999 |
| | Menta | Mexico, Aguascalientes | Bronchitis | Whole plant, decoction, oral | Dolores et al., 2003 |
| *Mentha spicata* L. | Spear mint | Morocco | Throat ailments, eadache, tiredness, diabetes | Aerial parts | El Menyiy et al., 2022 |
| | Imboza, Spearmint | South Africa | Cold, asthma | Leaves, decoction | Asowata-Ayodele et al., 2016 |
| | Kivirciknane | Turkey | Hemorrhoid | Aerial parts, infusion | Selvi et al., 2022 |
| *Mentha sylvestris* L. | Poneh, Fudenj | Iran | Rheumatism | Leaves, infusion | Naghibi et al., 2005 |
| *Mentha sativa* Willd. | Dub dubia | India | Constipation | NR | Jain & Tarafder, 1966 |
| *Mentha viridis* L. | Hierbabuena | Mexico, Aguascalientes | Stomachache & headache | Chewing leaves | Dolores et al., 2003 |
| | | Mexico, Chiapas | Stomachache, diarrhoea, & intestinal inflammation | Leaves, infusion, oral | Isidro, 1997 |
| *Mentha pulegium* L. | Poejo | Brazil | Stomachache, flu | Whole plant, infusion, oral | Voeks, 1996 |
| | Poleo | Mexico, Oaxaca | Infections | Leaves and branches | Pérez-Nicolas, 2017 |
| | | Mexico, Aguascalientes | Colics, stomachache, & fever | Aerial parts, decoction, oral | Dolores et al., 2003 |
| | | Mexico, Morelos | Flu | Aerial parts, infusion, night steaming | Monroy-Ortiz & Monroy, 2006 |
| *Mentha spicata* L. | Hiĉʃu | China | Throat inflammation | Leaves and roots, decoction, oral | Weckerle et al., 2006 |
| | | | Gingivitis | Fresh leaves, grinding, oral | |

| | | | | | |
|---|---|---|---|---|---|
| | Diosmos, spearmint | Greece | Inflammation of the epiglottis | Leaves and branches, infusion, oral | Brussell, 2004 |
| | Hierbabuena | Mexico, Oaxaca | Infections | Leaves and branches | Pérez-Nicolas, 2017 |
| | Yerba buena | US, California | Eye inflammation | Leaves, infusion, vaporizations | Timbrook, 1990 |
| *Micromeria biflora* (Buch. -Ham. ex D.Don) Benth. | Masinomran | Nepal | Wounds | Whole plant, grinding, cutaneous | Manandhar,1991 |
| *Micromeria frivaldszkyana (Degen) Velen* | NR | Bulgaria | Colds, wounds, skin infections | NR | Mladenova etal., 2021 |
| *Minthostachys mollis* (Kunth) Griseb. | Pichincha | Ecuador, Quito | Bronchitis & asthma | NR | Rios et al., 2007 |
| | Poleo | Ecuador | Headache | NR | Rios et al., 2007 |
| *Monarda fistulosa* L. | NR | Southern US | Cold & bronchitis | Bergamota and Leaves, infusion, oral | Core, 1966 |
| | Poaxu | | Cold | Burnt leaves, to inhale smoke | Kindscher & Hurlburt, 1988 |
| *Monardella villosa* Benth. | Poleo | US, California | Pneumonia | Aerial parts, compresses, cutaneous | Bocek, 1984 |
| *Nepeta cataria* Benth. | Catnip | Romania | colds, flu and fevers, antiseptic, antitussive | leaves and flowering tops | Padure et al., 2006 |
| | Catmint, Catnip | South Africa | Relaxant | Infusion, oral | Bhat & Moskovitz, 2009 |
| *Nepeta menthoides* Boiss. & Buhse. | Ostokhodus | Iran | Rheumatism | Aerial parts, decoction | Naghibi et al., 2005 |
| *Nepeta parviflora* Bieb. | NR | Moldova | Anti inflammatory | NR | Ghendov et al., 2013 |
| *Nepeta pannonica* L. | NR | Moldova | Anti inflamatory | NR | Ghendov et al., 2013 |
| *Ocimun americanum* L. | Peppermint | Nicaragua | Diarrhoea & stomachache | Leaves | Barret, 1994 |
| *Ocimum basilicum* L. | Nangpara | India, Nagaland | Stomachache, cholera, & dysentery | Dry leaves, infusion, oral | Rao & Jamir, 1982 |

| | | | | | |
|---|---|---|---|---|---|
| | NR | India | Headache, earache, fever, & cramps | NR | Jain & Tarafder, 1996 |
| | Reyhan | Iran | Anti-inflammatory | Leaves, Decoction | Naghibi et al., 2005 |
| | Vasilicò | Italia | Mouth/throat inflammations | Leaves, infusion, mouthwashes | Passalacqua et al., 2007 |
| | Albacar | Mexico, Oaxaca | Digestive system | Leaves and branches | Pérez-Nicolas, 2017 |
| | | Mexico, Morelos | Stomachache, headache, & toothache | Aerial parts, infusion, oral | Monroy-Ortiz & Monroy, 2006 |
| | | Mexico, Aguascalientes | Diarrhoea & fever | Aerial parts, decoction, oral | Dolores et al., 2003 |
| | Efinrin | Nigeria | Fever & hemorrhoids | Leaves, maceration, oral | Bhat et al., 1990 |
| | Feslegen | Turkey | Hemorrhoid | Leaves, infusion | Selvi et al., 2022 |
| *Ocimum canum* Sims. | NR | India | Heat in the head, headache, & cramps | NR | Jain & Tarfder, 1996 |
| *Ocimum gratissimum* L. | Romba | Madagascar | Blocked nose & headache | Whole plant, infusion, vaporizations | Quansah, 1988 |
| *Ocimum micranthum* Willd. | Albahaca cimarrona | Mexico, Chiapas | Antipyretic | Leaves, decoction, cutaneous | Isidro, 1997 |
| | Albahaca | Mexico, Huasteca region | Stomachache & headache | Whole plant, infusion, oral | Alcorn, 1984 |
| | Hierba de la rellena | Mexico, Oaxaca | Skin infections & wounds | Branches | Pérez-Nicolas, 2017 |
| | Kakaltun | Mexico, Yucatán | Dysentery | Fruit juice, oral | Ankli et al., 1999 |
| | Albahaca | Nicaragua | Cold, diarrhoea, earache, fever, & headache | Leaves | Barret, 1994 |
| | Barsley | | Pain, hypertension, lung infections, & skin inflammation | Aerial parts, infusion or decoction, oral | Coe & Anderson, 1996 |
| *Ocimum sanctum* L. | Likõp | India | Fatigue | Ground leaves, cutaneous | Dagar, 1988 |

| | | | | | |
|---|---|---|---|---|---|
| | NR | | Chronic chest pain | Leaves mixed with *Adenostemma lavenia* L. and *Blumea balsamífera* L., mixed with water, internal | Dagar & Dagar, 1991 |
| | Tulsi | | Cold | Leaves, infusion, oral | Awasthi, 1991 |
| | Neogi | | Cold & flu | Leaves juice, oral | Neogi et al.,1988 |
| | Tulsi | | Tub | Leaves juice, oral | Siddiqui et al., 1988 |
| | NR | | Pain & constipation | NR | Jain & Tarafder, 1966 |
| | Jagu lu myah | Thailand, Akha | Stomachache | Leaves, infusion, oral | Anderson, 1985 |
| *Ocimum selloi* Benth. | Elixer paragorico | Brazil | Digestive problems | Leaves, infusion, oral | Voeks, 1996 |
| *Origanum majorana* L. | Rigani, oregano | Greece, Katohori | Cold & Flu | Leaves and branches, infusion, oral | Brussell, 2004 |
| | Mejorana | Mexico, Aguascalientes | Stomachache | Whole part, decoction, oral | Dolores et al., 2003 |
| | Sweet marjoram | South Africa | Digestive, tonic | Infusion, oral | Bhat & Moskovitz, 2009 |
| | Mercanköşk | Turkey, Anatolia | Diaphoretic & stomachache | Fresh leaves, eating, oral | Özgökçe & Özçelik, 2004 |
| *Origanum vulgare* L. | Bantulese | India | Flu & fever | Leaves, infusion, oral | Shah & Joshi, 1968 |
| | Orégano | Mexico, Aguascalientes | Colics & stomachache | Leaves and branches, decoction, oral | Dolores et al., 2003 |
| | Oregano, balsam | Montserrat, British territory | Cold | Aerial parts, infusion, oral | Brussell, 2004b |
| | Catir | Turkey, Anatolia | Wound & stomachache | Aerial parts, decoction, oral | Özgökçe & Özçelik, 2004 |
| | Karamercan | Turkey | Hemorrhoid | Aerial part, infusion | Selvi et al., 2022 |
| *Orthosiphon rubicundus* (D. Don) Benth. | Jikipota | India | Colics | Roots, infusion, oral | Pal & Jain,1988 |
| *Nepeta betonicifolia* C.A. Mey. | Sivripisikotu | Turkey | Rheumatism | Aerial parts, infusion | Selvi et al., 2022 |
| *Plectranthus amboinicus* Lour. | Tapete de Oxalá | Brazil | Digestive disorders | Leaves, infusion, oral | Voeks, 1996 |
| | Mili Tini, Coleus | Samoa | Cold | NR | Uhe, 1974 |

| | | | | | |
|---|---|---|---|---|---|
| *Plectranthus scutellarioides* Blume | Manto | Mexico, Chiapas | Swelling | Leaves, decoction, cutaneous | Isidro, 1997 |
| | La ' au Fai Sei | Samoa | Elephantiasis | NR | Uhe, 1974 |
| *Prunella vulgaris* L. | Self-Heal, Heal-all | Canada, Vancouver | Skin inflammation | Leaves, grinding, cutaneous | Turner & Bell, 1971 |
| | Neogi | India | Haemorrhoids | Leaves, grinding, cutaneous | Neogi et al., 1988 |
| | yaxalnichtz'i'lel | Mexico, Chiapas | Fever, inflammation | Leaves, infusion, oral | De la Cruz-Jimenez et al., 2014 |
| | Wirasarak | Southern US | Sores | Whole plant, cutaneous | Kindscher & Hurlburt, 1988 |
| *Salvia absconditiflora* (Montbret & Aucher ex Benth.) Greuter & Burdet | Karasalba | Turkey | Hemorrhoid | Aerial parts, infusion | Selvi et al., 2022 |
| *Salvia aethopis* L. | kizillik | Turkey | Healing wounds | Roots, decoction and grinding, cutaneous | Fujita et al., 1995 |
| *Salvia apiana* Jeps. | Salvia real, salvia mayor | US, California | Headache | Fresh leaves, cutaneous | Timbrook, 1990 |
| *Salvia dentata* Aiton | Bloublomsalies ("blue flower sages"), | South Africa | Treatment of coughs, colds, influenza, and stomach complaints | NR | Rattray et al., 2022 |
| *Salvia divinorum* Epling and Játiva | Pastora | Mexico, Oaxaca | Rheumatism | Leaves, infusion, oral | Casselman et al., 2014 |
| *Salvia elegans* Vahl | Hierba del burro, toronjil de monte | Mexico, Estado de México | Edemas & bruises | Roots and branches, grinding, cutaneous | Argueta et al., 1994 |
| *Salvia fruticosa* Miller | Greek sage | Greece | Anti inflammatory | NR | Ververis et al., 2020 |
| | Small sage | Israel | Rheumatism | Leaves and branches, infusion, oral | Rivera et al., 1994 |
| *Salvia libanotica* Boiss. et Gaill | Salima, asphacus | Lebanon | Colds and abdominal pain | Leaves water extracts | Gali-Muhtasib et al., 2000 |
| *Salvia officinalis* L. | Lysfakia, alisfakia, sage | Greece, Zagora | High blood pressure, lung problems, & malaria sweats | Aerial parts, infusion, oral | Brussell, 2004 |

| | | | | | |
|---|---|---|---|---|---|
| | NR | Greece | Rheumatism and arthritis | NR | Vokou et al., 1993 |
| | Dalmatian sage | Croatia+ Bosnia Herzegovina, Montenegro, Albania, Greece, Italy, Serbia | Stomach inflammation | Leaves | Grdisa et al., 2015 |
| *Salvia lavanduloides* Benth. | Altamiza, salvia morada | Mexico, Morelos | Stomachache, toothache, wounds, & fever | Aerial parts, infusion, oral | Argueta et al., 1994 |
| *Salvia microphylla* Kunth | Diente de acamaya, hierba de mirto | Mexico, Morelos, Puebla, Veracruz & Estado de México | Pain, rashes, infections, stomach inflammation, & wounds | Branches, infusion, oral | Argueta et al., 1994 |
| *Salvia misella* Kunth | NR | Mexico | Swelling | Branches, grinding, cutaneous | Argueta et al., 1994 |
| *Salvia rosmarinus* L. | Romero | Mexico, Aguascalientes | Stomachache & inflammation | Whole plant, decoction, oral | Dolores et al., 2003 |
| | | Spain, Almeria | Rheumatism & neuritis | Aerial parts, alcoholic infusion, cutaneous | Martínez-Lirola et al., 1996 |
| | | | Wounds | Aerial parts, decoction, cutaneous | |
| | | Southern US | Uterine fibrosis | NR | Balick et al., 2000 |
| *Salvia sagittata* Ruiz & Pav. | Quinde shungana | Ecuador | Gingivitis | NR | Rios et al., 2007 |
| *Salvia sclarea* L. | Ayi kulaği | Turkey, Ankora | Haemorrhoids | Aerial parts and roots, decoction, oral | Simsek et al., 2004 |
| *Salvia tomentosa* Miller | Şabla | Turkey | Pain & rheumatism | Aerial parts, decoction, bath | Fujita et al., 1995 |
| *Salvia triloba* L. | Faskomilo, wild alisfakia | Greece, Portaria | Cold & sinusitis | Leaves and branches, infusion, oral | Brussell, 2004 |
| | | | Respiratory problems & sweats from malaria | Leaves, decoction, cutaneous | |
| | | | Analgesic | Shredded leaves, cutaneous | |
| *Salvia verbenaca* L. | NR | Greece, Ano Lehonia | Lung congestion | Leaves and branches, infusion, oral | Brussell, 2004 |

| | | | | | |
|---|---|---|---|---|---|
| | Salamanquesa | NR | Burns | Leaves fresh, grinding, cutaneous | Brussell, 2004 |
| *Salvia verticillata* L. | Ada çayi | Turkey, Anatolia | Cold | Flowers, decoction, oral | Özgökçe & Özçelik, 2004 |
| | NR | Greece, Katohori | Respiratory diseases | Leaves and branches, decoction, internal | Brussell, 2004 |
| *Satureja douglasii* Benth. | Yerba buena | US, California | Toothache | Leaves, decoction, mouthwash | Bocek, 1984 |
| *Satureja hortensis L.* | Marzeh | Iran | Anti-inflammation | Aerial parts, decoction | Naghibi et al., 2005 |
| | Çibriska | Turkey | Urinary inflammations | Flowers, leaves, infusion | Selvi et al., 2022 |
| *Satureja parviflora* (Phil.) Epling | Muña-muña | Chile, Toconce | Stomachache & cold | Leaves, infusion, oral | Aldunate et al., 1983 |
| *Satureja thymbra* L. | NR | Turkey | Anti inflammatory | NR | Karabay–Yavasoglu et al., 2006 |
| *Solenostemon scutellarioides* (L.) Codd | Encerengga teung | Malaysia | Skin diseases | Leaves, infusion, cutaneous | Christensen, 2002 |
| *Teucrium chamaedrys* L. | NR | Greece | Haemorrhoids | NR | Vokou et al., 1993 |
| | Sanciotu | Turkey | Hemorrhoids, rheumatism | Aerial parts, mashed | Selvi et al., 2022 |
| *Teucrium flavum* | Sariyavsan | Turkey | Hemorrhoids | Aerial parts, ext. decoction | Selvi et al., 2022 |
| *Teucrium parviflorum* | Koyunotu | Turkey | Hemorrhoids | Aerial parts, ext. decoction | Selvi et al., 2022 |
| *Teucrium polium* L. | Aciyavsan | Turkey | Hemorrhoids, Rheumatism | Aerial parts, ext. fresh | Selvi et al., 2022 |
| | Maryamnokhodi | Iran | Antiinflammatory | Aerial parts, infusion | Naghibi et al., 2005 |
| *Thymus capitatus* Hoffm. et Link | NR | Mediterranean (Albania, Algeria, Baleares, Corse, Cyprus, East Aegean, Egypt, Greece, Italy, Crete, Lebanon, Sardinia, Sicilia, Spain, Syria, Libya, Morocco, | Dermatitis, pain, cough, pneumonia, bronchitis | NR | Bouyahya et al., 2020 |

| | | | | | |
|---|---|---|---|---|---|
| | | Palestine, Portugal, Tunisia & Turkey) | | | |
| *Thymus daenensis* Celak | Avishan | Iran | Anti-inflammatory | Whole parts, decoction | Naghibi et al., 2005 |
| *Thymus fallax* Fisch. & C.A. Mey. | Catri | Turkey | Anti-inflammatory | Aerial parts, decoction | Selvi et al., 2022 |
| *Thymus hirtus* Willd. *algeriensis Boiss. and Reut* | Djertil", "Hamzoucha", "Mezouqach", and "Khieta", azukenni | Algeria, Libya, Morocco, Mauritania, & Tunisia | Colds, anti-inflammatory, stomach pains, diabetes, rhinosinusitis, antitussive | Aerial parts | Lahlou et al., 2022 |
| *Thymus leucostomus* Hausskn. & Velen. ssp. *leucostomus* | Kekik | Turkey, Ankara | Bronchitis, expectorant, atherosclerosis, & antihypertensive | Aerial parts and roots, infusion, oral | Simsek et al., 2004 |
| | | | Stomachache & bronchitis | Leaves, infusion, internal | |
| | Anakekik | Turkey | Hemorrhoids, renal inflammations | Aerial parts, infusion | Selvi et al., 2022 |
| *Thymus linearis* Benth. ex Benth. | Ghoremachhau | Nepal | Gastrointestinal diseases | Aerial parts, decoction, internal | Bhattarai, 1992 |
| *Thymus longicaulis* C. Presl. | Keklikotu | Turkey | Stomachache | Aerial parts, decoction, internal | Fujita et al., 1995 |
| | Dagkekigi | | Hemorrhoids | Aerial parts, decoction | Selvi et al., 2022 |
| *Thymus longiflorus* Boiss. | Tomillo real | Spain, Almeria | Digestive problems | Leaves and flowers, infusion, oral | Martínez-Lirola et al., 1996 |
| *Thymus migricus* Klokov & Des.-Shost. | Peynirkekigi | Turkey | Anti-inflammatory | Aerial parts, infusion | Selvi et al., 2022 |
| *Thymus praecox* Opiz. | Yaylakekigi | Turkey | Anti-inflammatory | Aerial parts, infusion | Selvi et al., 2022 |
| *Thymus serphyllum* L. | NR | Ano Lehonia, Greece | Cold & digestive problems | Leaves and branches, infusion, oral | Brussell, 2004 |
| *Thymus sipyleus* Boiss. | Sipilkekigi | Turkey | Hemorrhoids | Aerial parts, infusion | Selvi et al., 2022 |
| | Kekik | Turkey | Cold | Aerial parts, decoction, oral | Fujita et al., 1995 |

| | | | | | |
|---|---|---|---|---|---|
| *Thymus transacaucasicus* Ronniger | Kirkekigi | Turkey | Anti-inflammatory | Aerial parts, infusion | Selvi et al., 2022 |
| *Thymus vulgaris* L. | Thyme | South Africa | Antibiotic, anti-inflammatory, antiseptic | Infusion, oral | Bhat & Moskovitz, 2009 |
| | Umakhunkula, Thyme | | | | |
| | Timó | Spain | Anti-inflammatory | Aerial parts, decoction, external | Raja et al., 1997 |
| *Thymus zygioides* Griseb. | Kekik | Turkey | Stomachache | Aerial parts, infusion, oral | Fujita et al., 1995 |
| *Thymus zygis* L. | Tomillo | Spain, Almeria | Toothache & digestive problems | Aerial parts, decoction, oral | Martínez-Lirola et al., 1996 |

## Bibliografía


Ajaib, M., Ishtiaq, M., Bhatti, K.H., Hussain, I., Maqbool, M., Hussain, T., Mushtaq, W., Ghani, A., Azeem, M., Khan, S.M.R., Thind, S., Bashir, R. (2021). Inventorization of traditional ethnobotanical uses of wild plants of Dawarian and Ratti Gali areas of District Neelum, Azad Jammu and Kashmir Pakistan. *Plos one* 16 (7): e0255010.

Alcorn, J. B. (1984). *Huastec Mayan Ethnobotany*. University of Texas Press.

Aldunate, C., Armesto, J. J., Castro, V., & Villagran, C. (1983). Ethnobotany of Pre-Altiplanic Community in the Andes of Northern Chile. *Economic Botany 37(1): 120-135*

Anderson, E. F. (1986). Ethnobotany of Hill Tribes of Northern Thailand. I. Medicinal Plants of Akha I. *Economic Botany* 40 (1): 38-53

Ankli, A., Sticher, O., Heinrich, M., & Anldi, A. (1999). medical ethnobotany of the yucatec maya: healers' consensus as a quantitative criterion. *Economic Botany* 53 (2): 144-160.

Argueta, A., Cano L., Rodarte, M. and Gallardo, C. (1994). *Atlas de las plantas de la medicina tradicional mexicana*. Instituto Nacional Indigenista.

Asowata-Ayodele, A. M., Afolayan, A.J., Otunola, G.A. (2016). Ethnobotanical survey of culinary herbs and spices used in the traditional medicinal system of Nkonkobe Municipality, Eastern Cape, South Africa. *South. African Journal of Botany* 104: 69–75.

Awasthi, A. K. (1991). Ethnobotanical studies of the negrito islanders of Andaman Islands, India-The great Andamanese. *Economic Botany* 45 (2): 274-280.

Balick, M. J., Kronenberg, F., Ososki, A. L., Fugh-berman, A., Roble, M., Lohr, P., Atha Balick, D., & Reiff, M. (2000). Medicinal plants used by latino healers for women's health conditions in new york city. *Economic Botany* 54 (3): 344-357.

Barret, B. (1994). Medicinal plants of Nicaragua´s Atlantic coast. *Economic Botany* 48 (1): 8-20.

Bhat, R. B., Etejere, E. O., & Oladipo, V. T. (1990). Ethnobotanical Studies from Central Nigeria. *Economic Botany* 44(3):382-390.

Bhat, R. B. & Moskovitz, G. (2009). Herbal medicinal teas from South Africa. *Fyton* 78: 67-73.

Bhattarai, N. K. (1992). Medical ethnobotany in the karnali zone, Nepal. *Economic Botany* 46 (3):257-261.

Bocek, B. (1925). Ethnobotany of Costanoan Indians, California, Based on Collections by Jonh P. Harrington. *Economic Botany* 38 (2): 240-255.

Bouyahya, A., Chamkhib, I., Guaouguaouc, F., Benalid, T., Balahbibe, A., El Omarif, N., Tahag, D., El-Shazlyh, M., & El Menyiyj, N. (2020). Ethnomedicinal use, phytochemistry, pharmacology, and food benefits of Thymus capitatus. *Journal of Ethnopharmacology* 259: 112925

Breedlove, D., Laughlin R. (1993). *The flowering of man: A Tzotzil Botany of Zinacantán*. Smithsonian Institution Press.

Brussell, D. (2004). A medicinal plant collection from Montserrat, west indies. *Economic Botany* 58(S) s203-s220.

Brussell, D. (2004b). Medicinal plants of Mt. Pelion, Greece. *Economic Botany*, 58(S): 176s-202s.

Bye, R. A. (1986). Medicinal plants of the Sierra Madre: comparative study of Tarahumara and Mexican market plants. *Economic Botany* 40 (1): 103-124.

Casselman, I., Nock, C. J., Wohlmuth, H., Weatherby, R. P., & Heinrich, M. (2014). From local to global - Fifty years of research on Salvia divinorum. In *Journal of Ethnopharmacology* 151 (2): 768–783. doi.org/10.1016/j.jep.2013.11.032.

Christensen, H. (2002). *Ethnobotany of the Iban & the Kelabit*. Oko-Tryk Press.

Chen, X., Dai, X., Liu, Y., HE, X., Gong, G. (2022). Isodon rubescens (Hemls.) Hara.; A comprehensive review in traditional uses, phytochemistry, and pharmacological activities. *Frontiers in pharmacology* 13: 766581.

Coe, F. G. & Anderson, G. J. (1996). Ethnobotany of the Garífuna of Nicaragua. *Economic Botany* 50 (1): 71-107.

Core, E. L. (1966). Ethnobotany of the Southern Appalachian Aborigines. *Economic Botany* 21(3): 198-214.

Cox Bodner, C. & Gereau, R. E. (1988). A Contribution to Bontoc Ethnobotany. *Economic Botany* 42 (3): 307-369.

Dagar, H. S. (1989). Plant Folk Medicines among Nicobarese Tribals of Car Nicobar Island, India. *Economic Botany* 43 (2): 215-224.

Dagar, H. S. & Dagar, J. C. (1990). Plant Folk Medicines among the Nicobarese of Katchal Island, India. *Economic Botany* 45 (1): 114-119.

De La Cruz-Jiménez, L., Guzmán-Lucio, M., & Viveros-Valdez, E. (2014). Traditional medicinal plants used for the treatment of gastrointestinal diseases in Chiapas, México. *World Applied Sciences Journal*, *31*(4), 508–515. doi.org/10.5829/idosi.wasj.2014.31.04.8381

Dolores, B. M., Croce, H. M., Cerda L.M. (2003). *Plantas útiles de la región semiárida de Aguascalientes*. Universidad autónoma de Aguascalientes.

El Menyiy, N., Naceiri Mrabti, H., El Omari, N., EI Bakili, A., Bakrim, S., Mekkaoui, M., Balahbib, A., Amiri-Ardekani, E., Ullah, R., Alqahtani, A., Shahat, A., and Bouyahya, A. (2022). Medicinal Uses, Phytochemistry, Pharmacology, and Toxicology of Mentha spicata. *Evidence-Based Complementary and Alternative Medicine* Volume 2022, Article ID 7990508, 32 pages https://doi.org/10.1155/2022/7990508

Fujita, T., Sezik, E., Tabata, M., Yesilada, G., Takeda, Y., Tanaka, T. & Takaishi, Y. (1995). Traditional medicine in Turkey VII. Folk medicine in middle and west black sea regions. *Economic Botany* 49 (4): 406-422.

Gali-Muhtasib, H., Hilan, C., & Khater, K. (2000). Traditional uses of Salvia libanotica (East Mediterranean sage) and the effects of its essential oils. *Journal of Ethnopharmacology* 71: 513–520

Ghendov, V., Doina, D. & Ciocarlan, N. 2013. Habitat assessment of Nepeta L. species with anti-inflammatory potential in the flora of Republic of Moldova In Biotehnologii avansate – realizări şi perspective: Simpozionul ştiinţific naţional cu participare internaţională, 24-25 octombrie 2013, Chişinău. Chișinău, Republica Moldova: Tipografia Academiei de Ştiinţe a Moldovei, 2013, Ediția III-a, p. 158

Gispert, C.M, Rodríguez G. H. (1988). *Los coras: plantas alimentarias y medicinales de su ambiente natural*. CONACULTA.

Grdisa, M., Jug-Dujakovic, M., Loncaric, M., Carovic, K., Nincevic, T., Liber, Z., Rodosavljevic, I. & Sativic, Z. (2015). Dalmatian sage (Salvia officinalis L.). A review of biochemical contents, medical propierties and genetic diversity. *Agriculturae Conspectus Scientificus* 80 (2):69-78.

Hazlett, D. L. (1986). Ethnobotanical Observations from Cabecar and Guaymi Settlements in Central America. *Economic Botany* 40 (3): 339-352.

Huai, H. Y., Pei, S. J. (2004). Plants used medicinally by folk healers of the Lahu people from the autonomous county of Jinping Miao, Yao, and Dai in southwest China. *Economic Botany* 58 (S): 265s-273s.

Isidro, V., M. (1997). *Etnobotánica de los Zoques de Tuxtla Gutierrez, Chiapas*. Instituto de historia natural.

Jain, S. K., & Tarafder, C. R. (1970). Medicinal plant-lore of the Santals (A Revival of P. O. Bodding's Work). *Economic Botany* 24: 241-278.

Karabay–Yavasoglu, N., Baykan, S., Ozturk, B., Apaydin, S., and Tuglular, I. (2006). Evaluation of the Antinociceptive and Anti-inflammatory Activities of Satureja thymbra L. Essential Oil. *Pharmaceutical Biology* 48 (8): 585-591.

Kindscher, K., & Hurlburt, D. P. (1998). Huron smith's ethnobotany of the Hocak (Winnebago). Economic Botany 52(4): 352-372.

Lal, S. D., & Yadav, B. K. (1983). Folk Medicines of Kurukshetra District (Haryana), India. *Economic Botany* 37 (3): 299-305.

Lahlou, R.A.; Samba, N.; Soeiro, P.; Alves, G.; Gonçalves, A.C.; Silva, L.R.; Silvestre, S.; Rodilla, J.; Ismael, M.I. Thymus hirtusWilld. ssp. algeriensis Boiss. and Reut: A Comprehensive Review on Phytochemistry, Bioactivities, and Health-Enhancing Effects. *Foods* 2022, 11, 3195. https://doi.org/10.3390/foods11203195

Lentz, D. L. (1986). Ethnobotany of the Jicaque of Honduras. *Economic Botany* 40 (2): 210-219.

Li, S., Yang, X., Li, Y., Shen, Y., Feng, L., Wang, Y., Zeng, H., Liu, X, Zhang, W. (2009). Chemical constituents of *Dracocephalum forrestii*. *Planta Medica* 75: 1591-1596.

Manandhar, N. P. (1991). Medicinal plant-lore of Tamang tribe of Kabhrepalanchok district, Nepal. *Economic Botany* 45 (1): 58-71.

Martínez-Lirola, M. J., Gonzalez-Tejero, M. R., Molero-Mesa (1996). Ethnobotanical resources in the province of Almería, Spain: campos de nijar. *Economic Botany* 50 (1): 40-56.

Mladenova, T.; Stoyanov, P.; Denev, P.; Dimitrova, S.; Katsarova, M.; Teneva, D.; Todorov, K.; Bivolarska, A. 2021. Phytochemical Composition, Antioxidant and Antimicrobial Activity of the Balkan Endemic Micromeria frivaldszkyana (Degen) Velen. (Lamiaceae). *Plants* 10, 710. https://doi.org/

10.3390/plants10040710Monroy-Ortiz, C., Monroy, R. (2006). *Las plantas, compañeras de siempre: La experiencia en Morelos*. Universidad Autónoma del Estado de Morelos.

Naghibi, F., Mosaddegh, M., Mohammadi, S., Ghorbani, A. (2005). Labiatae Family in folk Medicine in Iran from Ethnobotany to Pharmacology. Iranian Journal of Pharaceutical Research 2:63-79.

Neogi, B., Prasad, M. N. & Rao, A. (1989). Ethnobotany of Some Weeds of Khasi and Garo Hills, Meghalaya, Northeastern India. *Economic Botany* 43 (4): 471-479.

Nicholson, M. S., & Arzeni, C. B. (1993). The market medicinal plants of Monterrey, Nuevo León, México. *Economic Botany* 47 (2): 184-192.

Özgökçe, F. & Özçelik, H. (2004). Ethnobotanical aspects of some Anatolia, Turkey. *Economic Botany* 48 (4): 697-704.

Padure, I., Badulescu, L., Burzo, I., & Dan, M. 2006. The essential oils of Nepeta L. genus (Lamiaceae, Nepetoideae) in Romania. 4th Conference on Medicinal and Aromatic Plants of South-East European Countries

Pal, D. C. & Jain, S. K. (1989). Notes on Lodha medicine in Midnapur district, west bengal, India. *Economic Botany* 43 (4): 464-470.

Passalacqua, N.G., Guarrera, P.M., De Fine, G. (2007). Contribution to the knowledge of the folk plant medicine in Calabria region (Southern Italy). Fitoterapia 78: 52-68.

Pérez-Nicolás, M., Vibrans, H., Romero-Manzanares, A., Saynes-Vásquez, A., Luna-Cavazos, M., Flores-Cruz, M., & Lira-Saade, R. (2017). Patterns of Knowledge and Use of Medicinal Plants in Santiago Camotlán, Oaxaca, Mexico. *Economic Botany*, *71*(3): 209–223. doi.org/10.1007/s12231-017-9384-0

Quansah, N. (1988). Ethnomedicine in the Maroantsetra Region of Madagascar. *Economic Botany*, 42 (3): 370-375.

Raja, D., Blanché, C. Vallès-Xirau, J. (1997). Contribution to the knowledge of the pharmaceutical ethnobotany of La Segarra region (Catalonia, Iberian Peninsula). Journal of Ethnopharmacology 57: 149-160.

Rao, R. & Jamir, N. (1982). Ethnobotanical studies in Nagaland. *Economic Botany*, 36(2): 176-181.

Rattray, R.D.; Mokwena, L.; Stander, M.A.; VanWyk, B.-E. 2022. Essential Oil Composition and Traditional Uses of Salvia dentata, a Poorly Known Medicinal Plant from Namaqualand, South Africa. *Molecules* 2022, 27, 6478. https://doi.org/10.3390/molecules27196478.

Rios, M., Koziol, M., Borgtoft, H., Granda, G. (2007). *Plantas útiles del Ecuador*. Abya-Yala.

Rivera, D., Obón, C., Cano, F. (1994). The botany, history and traditional uses of three- lobed sage (*Salvia fruticosa* Miller) (Labiatae). *Economic Botany* 48 (2): 190-195.

Shah, N. & Joshi, M. (1968). An Ethnobotanical Study of the Kumaon Region of India. *Economic Botany*, 25: 414-422.

Selvi, S., Polat, R., Çakilcioglu, U., Celep, F., Firmenci, T. (2022). An ethnobotanical review on medicinal plants of the Lamiaceae family in Turkey. Turkish Journal of Botany 46(4):1

Siddiqui, M. B., Mashkoor, A. & Husain, W. (1989). Traditional treatment of skin diseases in Uttar Pradesh, India. *Economic Botany*, 25: 414-422.

Simsek, I., Aytekin, F., Yesilada, E. & Yildirimli, S. (2004). An ethnobotanical survey of the Beypazari, Ayas, and Gudul district towns of Ankara province (Turkey*). Economic Botany*, 58(4): 705-720.

Timbrook, J. (1990). Ethnobotany of Chumash Indians, California, Based on Collections by Jonh P. Harrington. *Economic Botany* 44 (2): 236-253.

Turner, N. & Bell, M. (1971). The Ethnobotany of the Coast Salish Indians of Vancouver Island. *Economic Botany* 25: 63-99.

Uhe, G. (1974). Medicinal plants of Samoa a preliminary survey of the use of plants for medicinal purposes in the Samoan Islands. *Economic Botany* 28: 1-30.

Ververis, A., Savvidou, G., Ioannou, K., Nicolaou, P. Christodoulou, K., and Plioukas, M. 2020. Greek Sage Exhibits Neuroprotective Activity against Amyloid Beta-Induced Toxicity. *Evidence-Based Complementary and Alternative Medicine* Volume 2020, Article ID 2975284, 10 pages https://doi.org/10.1155/2020/2975284

Voeks, R. A. (1996). Tropical forest healers and habitat preference. *Economic Botany* 50 (4): 381-400.

Vokow, D., Katradi, K. & Kokkini, S. (1993). Ethnobotanical survey of Zagori (Epirus, Greece), a renowned centre of folk medicine in the past. Journal of Ethnopharmacology 39:187-196.

Weckerle, C., Huber, F., Yongping, Y. & Weibang, S. (2006). Plant knowledge of the shuhi in the hengduan mountains, southwest China. *Economic Botany* 60 (1): 3-23.
