# Supplementary material for: Ethnobotanical, Phytochemical, and Pharmacological Properties of the Subfamily Nepetoideae (Lamiaceae) in Inflammatory Diseases
Source: Plants (Basel). 2023 Nov 2;12(21):3752. doi: 10.3390/plants12213752 (PMC10648697; doi:10.3390/plants12213752)
Supplement: Supplementary file 1 [file plants-12-03752-s001.zip › Table S2.pdf]

Table S2. Pharmacological evaluation of the extracts related to inflammatory processes in species of the Nepetoideae (Lamiaceae) subfamily.

Species	Plant part(s)	Pharmacology activity	Extract(s)	Reference
Agastache mexicana Lint. & Epling	Aerial parts & inflorescences	Antinociceptive effects at 10-1000 mg/kg, i.p. in both neurogenic and inflammatory phases in the formalin test in Wistar rats. Antinociceptive and anti-inflammatory activities in the writing (ED_{50}= 2 mg/kg, i.p.) & formalin (ED_{50}= 44 mg/kg i.p.) tests in Swiss albino mice and Wistar rats, respectively. Reduction on the colonic inflammatory cytokines at doses of 3-300 mg/kg, p.o. using the oxazolone-induced colitis model in male BALB/c mice.	Essential oil, hexane, ethyl acetate, & methanol extracts.	González-Ramírez et al., 2012; Verano et al., 2013; González-Ramírez et al., 2021.
Agastache rugosa Kuntze	Aerial parts & leaves	Antioxidant activity using 2,2-difenil-1-picrilhidrazilo (DPPH) with a scavenging concentration fifty (SC_{50}= 77.4 ± 1.9 μg/mL). Reduction on the nitric oxide (NO) and nitric oxide synthase (NOS), and down regulation of the mARN of IL-6. NO decrease with an inhibitory concentration fifty (IC_{50}) =7.9 μg/mL, and in the reactive oxygen species (ROS) IC_{50}=4.3 μg/mL, NOS IC_{50}=30.0 μg/mL. Reduction of DPPH with a SC_{50}=2.9 mg/mL; ROS SC_{50}=1.4 mg/mL, and NO SC_{50}=1.7 mg/mL.	Ethyl acetate, aqueous fermented extract with *Lactobacillus rhamnosus* HK-9, & aqueous extract.	Kim et al., 2017; Lim et al., 2017; Lee et al., 2020.
Asterohyptis stellulata (Benth.) Epling	Aerial parts	Reduction of DPPH, SC_{50}= 112.84 ± 3.71 μg/mL. Promotion of skin regeneration in male mice CD-1 et/et line.	Methanol extract	Álvarez-Santos et al., 2022.
Cedronella canariensis (L.) Webb & Berthel.	Flowers	Anti-inflammatory activity in carrageenan-induced edema model in HC/CFLP mice (1000 mg/kg, p.o.).	Chloroformic fraction from methanol extract.	Lopez-García et al., 1991.
Clinopodium bolivianum (Benth.) Kuntze	Aerial parts	Reduction of IL-8 in urothelial cells infected with *Escherichia coli.*	Aqueous extract	Mohanty et al., 2017.
Clinopodium chinense (Benth.) Kuntze	Whole plant	Inhibition on the inflammation induced by LPS-TLR4-NF-κB-iNOS/COX-2 signaling pathway in RAW264.7 cells. Decrease expression of PR, MMP-9, & VEGF. Increase in levels of TXB2 and the expression of VEGF. Oxidative stress inhibition by antioxidant activity.	Aqueous extract	Yu et al., 2019; Kim et al., 2020; Li et al., 2020; Li et al., 2022; Wang et al., 2023.

Species	Plant part(s)	Pharmacology activity	Extract(s)	Reference
Clinopodium gracile (Benth.) Kuntze	Aerial parts	Inhibition of mast cell-derived cells that cause allergic inflammation.	Aqueous extract	Park et al., 2010.
Clinopodium polycephalum (Vaniot) C.Y. Wu & S.J. Hsuan	Root & stem	Anti-inflammatory activity in RAW 264.7 macrophages induced with LPS.	Ethanol extract 70%.	Liu et al., 2022.
Clinopodium vulgare L.	Flowers	Modulation of COX-2 inhibitors. Suppresses NF-κB activation by preventing Iκ-B phosphorylation and inhibits the phosphorylation of p38 and SAPK/JNK MAPKs. It down-regulates iNOS expression with a drastic decrease of NO production, inhibits MMP-9 activation, but does not affect COX-2.	Aqueous extract	Petrova et al., 2023; Dobrev, 2021; Amirova et al., 2019; Burk et al., 2009.
Coleus amboinicus Lour.	Leaves	Administration reduced cisplatin-induced renal cell damage and an inhibition on the process of kidney fibrosis in Wistar rats. Promote the anti-inflammatory cytokine TGF-β1 and glutathione peroxidase (GPx) concentrations to regulate cell proliferation, apoptosis, chemotaxis, immunity, inflammatory response, and tissue repair (fibrosis) of kidney tissue. It could effectively reduce the blood urea nitrogen, serum creatinine, GPx, & the TGF-β1 concentrations in the uric acid induced rats.	Ethanol extract	Sahrial & Solfaine 2019; Solfaine et al. 2021.
Coleus scutellarioides (L.) Benth.	Leaves	Anti-inflammatory properties of quercetin by inhibiting the production of PGs through inhibition of COX-2 activity.	Hexane & essential oil.	Mustaricihie et al., 2017; Mustaricihie et al., 2022.
Collinsonia canadensis L.	Aerial parts	Reduction of the NO concentrations.	Thymol & carvacrol.	Mahomoodall et al., 2021.
Dracocephalum heterophyllum Benth.	Whole plant	Extracts can effectively suppress CD4+ T cell proliferation & prolong the survival and reduced mortality in mice. Rosmarinate, luteolin & diosmetin suppress abnormal CD4+ T-cell proliferation	Ethanol- ethyl acetate, petroleum ether, ethyl acetate, *n*-BuOH, & water.	Shi et al. 2016; Bian et al., 2020.

Species	Plant part(s)	Pharmacology activity	Extract(s)	Reference
Dracocephalum forrestii W.W. Sm.	Whole plant	Ethanol extract and 4-hydroxi-3-methoxyphenylethanol-8-O-[(6-O-siringoil)-β-D-glucopiranoside] (1) decrease NO levels in macrophages RAW 264.7. Inhibition of 33.3%-41.6% at 100 μg/mL.	Ethanol extract	Li et al., 2009.
Dracocephalum kotschyi Boiss.	Aerial parts and leafy branches	Ethyl acetate extract and apigenin produce anti-inflammatory effects in colitis induced by acetic acid model in male Wistar rats (10-40 mg/kg & 5-20 mg/kg, p.o.). The ethyl acetate extract reduces the expression of iNOS, COX-2, IL-1β, tumor necrosis factor (TNF)-$\boldsymbol{\alpha}$ in LPS-stimulated J774.1 mouse macrophages at 25 μg/mL. Prevent hydroxyproline & malondialdehyde increase levels in fibrosis model induced by bleomycin in Wistar rats (20-80 mg/kg, p.o.) Extracts prevent the severity of gastric ulcer induction by indomethacin in male Wistar rats (80 and 40 mg/kg, p.o.)	Ethyl acetate, hydroalcoholic, aqueous extract, & flavonoid extract.	Sandraei et al. 2017; Kalantar et al., 2018; Hosseini-Sharifabad et al, 2021; Minaiyan et al., 2021.
Dracocephalum moldavica L.	Aerial parts	Suppression of PCNA, NF-κB p65, ICAM-1, & CAM-1 in rat vascular smooth muscle cells induced by TNF-α. Tilianin reduces mRNA of TNF-α, NF-κB, MCP-1 & iNOS at 50 μM in macrophages induced by LPS. It suppresses the release of NO & PGE2. Inhibits the mRNA and protein expression level of iNOS & COX-2, respectively. Reduces the release of proinflammatory cytokines such as IL-6, IL-1β in RAW 364.7 cells induced with LPS. Dracocefalumoides A, uncinatona, tricotonoma F, & cariopterissoide C produced inhibition against TNF-α, IL-1β & NO im macrophages RAW 264.7 induced by LPS, IC_{50} = 1.12 – 5.84 μM.	Ethanol & ethanol-aqueous extracts.	Xing et al., 2013; Shen et al., 2019; Kim et al., 2021; Nie et al., 2021; Sheychenko et al., 2021.
Dracocephalum palmatum Stephan ex Willd.	Aerial parts. The plant's young shoots and flowers.	Anti-inflammatory, diuretic, gastrointestinal improvement, & alcoholism treatment. Cosmosiin possesses anti-inflammatory, insulin mimetic, & cancer preventive activities. Cynaroside produces anti-atherosclerotic, anti-inflammatory, anti-diabetic and cardioprotective effects. Eriodictyol possesses anticancer, antioxidant, antidiabetic, anti-inflammatory, & cytoprotective properties.	Decoction, infusion, & tincture.	Olennikov et al., 2013; Andreyeva et al., 2020; Chirikova et al., 2021.

Species	Plant part(s)	Pharmacology activity	Extract(s)	Reference
Dracocephalum rupestre Hance	Whole plant	Total phenolic & flavonoids contribute to the high radical-scavenging activity. Fraction EA protects on the hepatic injury by *in vivo* antioxidant action. Eriodictyol produced neuroprotective effects on memory deficits and neuroinflammation in mice subjected to permanent middle cerebral artery occlusion (pMCAO).	Ethanol-aqueous (EA) from petroleum ether or ethyl acetate extracts	Ferreira et al., 2016; Zhu et al., 2017.
Elsholtzia ciliata (Thunb.) Hyl.	Leaves & aerial parts	Anti-inflammatory activity at 100 mg/kg, p.o. in the formalin test in Swiss Webster mice. Attenuates renal inflammation in renal interstitial fibrosis induced by unilateral ureteral obstruction model in male Sprague Dawley (300 & 500 mg/kg, p.o. for 14 days). Reduction on the levels of proinflammatory cytokines (TNF-α, IL-6) and prostaglandin E2 induced by LPS in mouse macrophages. Luteolin, caffeic acid, vitexin, pedalin, luteolin-7-O-β-D-glucopyranoside, apigenin-5-O-β-D-glucopyranoside, apigenin-7-O-β-D-glucopyranoside chrysoeriol-7-O-β-D-glucopyranoside, 7,3'-methoxy luteolin-6-O-β-D-glucopyranoside, 5,6,4′-trihydroxy-7,3′-dimethoxyflavone, 5-hydroxy-6,7-dimethoxyflavone, 4-(E)-caffeoyl-L-threonic acid, 4-O-(E)-p-coumaroyl-L-threonic acid, α-linolenic acid, all of them reduced ROS released in macrophages J774A.1 LPS-induced at 10-2.5 μM doses. Inhibits mast cell-mediated allergic inflammatory reactions by suppressing histamine release and proinflammatory cytokine expression, and involvement of calcium, NF-κB & p38 MAPK in these effects.	Hexane, dichloromethane, ethanol, petroleum ether, diethyl ether, ethyl acetate, & aqueous extracts.	Wang et al., 2022; Zhang et al., 2021; Zotsenko et al., 2021; Nguyen et al., 2021; Pudziuvelyte et al., 2020; Kim et al., 2016; Kim et al., 2011.
Elsholtzia densa Benth.	Aerial parts	Anti-inflammatory effects by inhibition of 27.27% at 300 mg/kg, p.o. in the carrageenan induced- edema in Wistar rats. Significant decrease of the secretion of proinflammatory cytokines TNF-α, IL-1β, & IL-6 of 80.93%, 75.33%, & 67.09%, respectively, in serum of Wistar rats.	Methanol extract	Zargar et al., 2019.
Elsholtzia rugulosa Hemsl.	Aerial parts	All the isolated compounds were evaluated for the anti-inflammatory activity by assessing the inhibition of LPS-induced NO production in RAW264.7 mouse macrophages. Compounds such as rugulolides A, nepetoidin B, methyl rosmarinate, & syringaresinol, mildly inhibited NO production with IC_{50} values ranging from 12.46 to 23.10 μM, and others were inactive ($IC_{50} > 50$ μM).	Aqueous-ethanol (70%) extract.,	Yang et al., 2021.

Species	Plant part(s)	Pharmacology activity	Extract(s)	Reference
Elsoltzia stachyodes (Link) C.Y. Wu	Whole plant	Anti-inflammatory effect observed by 25 ± 1.08 inhibition at 200 mg/kg, p.o. in the edema induced with carrageenan in Wistar rats.	Aqueous extract	Banerjee et al., 2021.
Elsholtzia stauntonii Benth.	Aerial parts	Non-toxic extract (class V) according to the Sidorov classification. Antinociceptive and anti-inflammatory effects in the writhing test and edema induced with carrageenan test in a murine model using 100 mg/kg, p.o.	Ethanol extract	Zotsenko et al., 2021.
Elsoltzia splendens Nakai	Aerial parts	Antinociceptive and anti-inflammatory effects by inhibition of 44.6% using a dosage of 400 mg/kg, p.o. in the croton oil-induced ear edema and 50% in the writhing test in ICR mice. A PGE_2 inhibition was also observed in RAW 264.7 macrophages induced with LPS.	Ethanol extract 75%.	Kim et al., 2003.
Eplingiella fruticosa (Salzm. ex Benth.) Harl & J.F.B. Pastore	Leaves	Anti-inflammatory activity was evaluated using the paw oedema model induced by 1% carrageenan.	Ethanol extract 90%.	Andrade et al., 2010; Beserra-Filho et al., 2019.
Glechoma hederacea L.	Whole plant	Inhibition of NO, (IL)-12p70, IL-12p40, & TNF-α in LPS-induced mouse peritoneal macrophages. Anti-inflammatory activity in the luciferase assay in HepG2 cells, where methyl isoferuloyl-7-(3,4-dihydroxyphenyl) lactate reduced NF-κB activation in 45.4% at 20μM; methyl rosmarinate reduced 44.3% & ethyl rosmarinate, benzyl-4'-hydroxy-benzoyl-3'-O-β-D-glucopyranoside in 57.0% at 20μM. Inhibition of PPAR-α, PPAR-γ, NF-κB, E-selectin, IL-8 in LPS-induced endothelial cells. Protective effect in rat model of induced cholestasis by attenuation of NF-κb & AP-1. Attenuation of NO levels, regulation of iNOS, COX-2, HO-1, & TNF-α in RAW 264.7 macrophages induced with LPS.	Aqueous, ethanol, & methanol destanified extract. Essential oil.	An et al., 2006; Kim et al., 2011; Vogl et al., 2013; Wang et al., 2017; Chou et al., 2018.
Glechoma longituba (Nakai) Kuprian.	Whole plant	Apigenin-7-diglucoronide produced attenuation of proinflammatory gene expression in retinas exposed to bright light in BALB/c mice.	Aqueous extract	Bian et al., 2017.
Horminum pyrenaicum L.	Root	Suppression of (IFN-γ-)-dependent immunometabolic pathways, tryptophan breakdown via IDO-1, & neopterin formation by GTP-CH-I. Pathways related to inflammatory processes.	Petroleum ether extract	Becker et al., 2018.
Hoslundia opposita Vahl	Root	Antinociceptive, anti-inflammatory, & antipyretic effects in Swiss mice and Wistar rats using the writhing test (22.7% inhibition at 100 mg/kg, p.o.), carrageenan-induced edema	Chloroform extract	Olajide et al., 1998.

Species	Plant part(s)	Pharmacology activity	Extract(s)	Reference
		(76% inhibition at 100 mg/kg, p.o.), and yeast-induced hyperthermia (significant decrease at 100 mg/kg).		
Hyptis capitata Jacq.	Leaves & seeds	The anti-inflammatory potential of the extract by determining their ability to inhibit NO production using LPS activated macrophages, and the effect on the production of TNF-α, & interleukins 1β or 6 (IL-1β & IL-6) in macrophages.<br>Inhibitor of the NO production in *in vitro* assay.	96% ethanol extract. Alkaloids, coumarins, glycosides, flavonoids, quinones.	Castro et al., 2021.
Hyptis crenata Pohl ex Benth.	Leaves & aerial parts.	Reduction of the pro-inflammatory cytokines (IL-6, MCP-1, IFN-γ, TNF-α, & IL-12p70) & increase in the anti-inflammatory cytokine IL-10, as well as an inhibitory myeloperoxidase activity.	Aqueous extract	De Jesus et al., 2009; Coelho-De-Souza et al., 2021; Allves-Soares et al., 2022; Shipa et al., 2022; De Lima et al., 2023.
Hyptis pectinata (L.) Poit.	Flowers	Antinociceptive and anti-inflammatory activities in the 2nd phase of the formalin test (37.4% inhibition at 200 mg/kg, p.o.), carrageenan-induced peritonitis (reduction of leukocyte migration 3.97 * 106 cells/mL at 200 mg/kg, p.o.), & TPA-induced edema (0.27 ± 0.02 mm decrease at 3.0 mg/ear) in Swiss mice.	Dichlorometha ne fraction from ethanol extract (60%).	Santana et al., 2022.
Hyptis suaveolens (L.) Poit.	Leaves	Anti-inflammatory activity in the edema induced with carrageenan in albino rats. Suaveolol ID_{50} = 0.71 μmol/cm^2 & methyl suveolate ID_{50} = 0.60 μmol/cm^2 in the TPA-induced edema.	Ethanol extract	Almeida-Bezerra et al., 2022.
Hyptis umbrosa Salzm. ex Benth.	Leaves	Antinociceptive and anti-inflammatory activity in writhing test by significant inhibition at 100-400 mg/kg & formalin test with significant inhibition in the first phase at 400 mg/kg, p.o. in Swiss mice.	Ethanol extract 95%.	Anjos et al., 2017.
Hissopus cuspidatus Boriss.	Aerial parts	Attenuation of the LPS-induced inflammatory responses by inhibition of iNOS and COX-2 expression through suppression of NF-κB activation. JAX2 significantly inhibits airway remodeling in asthma, inhibiting the proliferation and migration of hASMCs, releasing inflammatory factors and metalloproteinases, activating the ERK1/2 signal pathway, & promoting the secretion of anti-inflammatory factors.	Ethanol extract	Qin et al., 2023; Aihaiti et al., 2023; Cai et al., 2023; Ling-Fei, K. et al, 2023; Liu et al., 2021; Liu et al., 2021b: Yuan et al., 2019; Zhao et al., 2013.

Species	Plant part(s)	Pharmacology activity	Extract(s)	Reference
Hyssopus officinalis L.	Aerial parts	Anti- inflammatory effect of the extract at 200 mg/kg, p.o. in the edema induced by carrageenan in Wistar rats. Inflammation reduced at 5 mg/kg by modulation of the IL-1B & IL-10 in the carrageenan-induced edema. Immune and inflammatory regulation of the airways in BALB/c mouse model of chronic asthma. A decrease in levels of eosinophils & IgE in bronchial alveolar fluid at 0.04 g/10 g, p.o.	Aqueous extract, aqueous-nanoparticles of ZnO, methanol extract.	Ma et al., 2014; Mohammad et al., 2019; Micovic et al., 2022.
Isodon adenanthus (Diels) Kudô	Leaves	Adenanthine prevents encephalomyelitis by inhibiting NF-κB signaling in C57BL/6 mice.	Adenanthine	Yin et al., 2013.
Isodon amethystoides (Benth.) H. Hara	Whole plant	Glaucocalyxin A inhibited LPS-induced production of NO and reversed the morphological changes in primary microglia. It suppressed expression of iNOS & COX-2 dose-dependently at the mRNA and protein levels. The production of proinflammatory cytokines such as TNF-a, IL-1b & IL-6 was inhibited by suppressing their transcriptional activity.	Methanol extract	Xiang et al. 2014.
Isodon coetsa (Buch.-Ham. ex D. Don) Kudô	Leaves	Clastogenic activity with increasing concentrations and time durations. The major abnormalities included chromosome fragments, stickiness, ring chromosomes, chromosome bridges, pulverization, binucleate cell, micronucleus, ball metaphase, chromosome laggards, and shift in microtubule organizing center. Possible toxic activity of the extract suggests judicious use.	Aqueous extract	Neelamkavil & Thoppil, 2013; 2014.
Isodon excisus (Maxim.) Kudô	Leaves	Inflexinol (1, 5, 10 μM) suppressed the expression of iNOS & COX-2, as well as the production of NO in LPS-stimulated RAW 264.7 cells and astrocytes. Consistent with the inhibitory effect on iNOS and COX-2 expression, inflexinol also inhibited transcriptional and DNA binding activity of NF-κB via inhibition of IκB degradation as well as p50 and p65 translocation into nucleus.<br>Inflexin exhibits antiinflammatory effects suppressing NO release in primary culture of rat microglia cells and BV-2 microglial cells. Inflexin also significantly inhibited the release of proinflammatory mediators and the release of iNOS, COX-2, IL-1β, IL-6 and TNF-α in LPS-stimulated BV-2 microglial cells. The anti-inflammatory properties of inflexin are mediated by down-regulation of NF-κB and Akt but not MAPKs activation in BV-2 microglial cells.	Methanol extract	Lee et al., 2007; Ko et al., 2010.

Species	Plant part(s)	Pharmacology activity	Extract(s)	Reference
Isodon henryi (Hemsl.) Kudô	Whole plant	Rabdoternin A, lasiodonin : Inhibición de NO en macrófagos RAW 264.7 inducidos con LPS (IC_{50}= 6.70, 5.85 μM, respectivamente)	Ethanol extract 70%.	Cheng et al., 2022.
Isodon japonicus (Burm. f.) H. Hara	Aerial parts	Inhibitory effects on LPS-induced NO & PGE_2 production in RAW264.7 cells. Kamebanin (IC_{50} = 0.06 μM), kamebacetal A (IC_{50} = 0.58 μM), kamebakaurin (IC_{50} = 0.15 μM), excisanin A (IC_{50} = 0.35 μM).<br>Oridonin strongly suppressed particulate-induced cell death accompanied by the release of IL-1α & IL-1β in mouse and human macrophages. The extract attenuated expression of NO, iNOS & TNF-α in BV2 microglial cell LPS-stimulated. The extract inhibited compound 48/80-induced systemic reactions and plasma histamine release in mice. Decreased the PCA reaction activated by antidinitrophenyl (DNP) IgE antibody. Kamebakaurin inhibited the LPS-induced production of NO in activated microglial cells. The mRNA and protein levels of inducible iNOS and COX-2 were also decreased. Effusanin C inhibited the production of inflammatory mediators NO, IL-1β, and TNF-α in macrophages and dendritic cells.<br>Pretreatment with isodojaponin D (5 and 10 μg/ml) prior to treatment with LPS significantly decreased LPS-induced production of COX-2 and iNOS in a dose-dependent manner. In addition, LPS-induced pro-inflammatory cytokines (IL-1β, IL-6, and TNF-α) were also decreased by pretreatment with isodojaponin D.	Methanol extract & Aqueous extract.	Hwang et al. 2001; Shin et al., 2004; Hong et al., 2009; Lim et al. 2010; Kim et al., 2011; Kim et al., 2013; Kang, 2018; Ikoma et al., 2022.
Isodon melissoides (Benth.) H.W. Li	Aerial parts	Overproduction of pro-inflammatory cytokines was significantly decreased in the essential oil treated cells in LPS-induced inflammation in HaCaT cells in a dose-depende nt manner without any cytotoxic effect.<br>Melissoidesin inhibited the growth of human leukemia cell lines and primary acute myeloid leukemia (AML) blasts via induction of apoptosis, with the evidence of mitochondrial DWm loss, ROS production, caspases activation, & nuclear fragmentation.	Essential oil	Yu et al., 2007; Kumar et al., 2021.
Isodon scoparius C.Y. Wu & H.W. Li) H. Hara	Aerial parts	Scopariusol L inhibited NO in RAW 264.7 macrophages induced with LPS (IC_{50}= 0.6 $\mu M^{*}L^{-1}$)	Acetone extract 70%.	Jiang et al., 2018.
Isodon serra (Maxim.) Kudô	Aerial parts	Oridonin (12 μg/mL) inhibited BAFF expression in mouse macrophages, and *in vivo* administration (9 mg/kg) ameliorated the serological and clinical manifestations of systemic lupus erythematosus in MRLlpr/lpr mice. Serrin F, 14b-hydroxyrabdocoestin A,	Non described	Zhang et al., 2005; Li et al., 2010; Zhou et al. 2013; Wan et al., 2016; Xing et al., 2020.

Species	Plant part(s)	Pharmacology activity	Extract(s)	Reference
		serrin H, serrin I, enanderianin N, & megathyrin B strongly inhibited NO production in LPS-stimulated RAW264.7 cells.<br>Enmein produced potential immunosuppressive action in *in vitro* and i*n vivo* models. Isoserrin A, B, C, D, E, F, G, H & I exhibited notable NO production inhibition in LPS-stimulated BV-2 cells (IC_{5O}= 15.6-7.3 μM). Nodosin depressed the murine ear-swelling extent and the IL-2 levels in the blood serum.		
Isodon sculponeatus (Vaniot) Kudô	Aerial parts	Sculponeatin J produced cytotoxic activity against five human tumor cell lines with IC_{50} values ranging from 1.8 mmol/L to 3.3 mmol/L, and it also inhibited NO production in LPS-stimulated RAW264.7 cells, with IC_{50} value of 3.3 mmol/L. Sculponin T & sculponeatin C showed moderate cytotoxicity in the same tumor cell lines. Sculponins Y exhibited weak cytotoxic activity against HL-60, SMMC-7721, MCF-7, & SW-480 cell lines. It also inhibited NO production in LPS-stimulated RAW264.7 cells with IC_{50} value of 13.8 μM.	70% aqueous acetone, and then partitioned between ethyl acetate & water.	Jiang et al., 2014a; 2014b.
Isodon ternifolius (D.Don) Kudô	Root	Ternifoliuslignane A-D & 3-carboxy-6,7-dihydroxy-1-(3′,4′-dihydroxyphenyl)-naphthalene produced NO inhibition in LPS-induced RAW 264.7 macrophages (IC_{50} = 9.98–29.14 μM).	Methanol extract	Zhang et al., 2018.
Isodon rubescens (Hemsl.) H. Hara	Aerial parts	Isojiangrubesina B, isojiangrubesina E, effusanin A, longikaurin A, & 7,14-O-(1-metiletilidene) oridonin produced inhibition of NO in RAW 264.7 macrophages induced with LPS (IC50 =1.2, 1.3, 1.3, 0.8, 1.1 μM, respectively). Orodinin was covalent and specific inhibitor for the NLRP3 inflammasome in mouse models of peritonitis, gouty arthritis, & type 2 diabetes. It significantly suppressed the production of MMP1, MMP3 and MMP13. It attenuated NO and PGE2 production, as well as iNOS and COX-2 expression in IL-1β-induced human osteoarthritis chondrocytes.	Ethanol extract 80%, acetone extract 70%.	Du et al., 2013;<br>Zhang et al., 2017;<br>He et al., 2018;<br>Jia et al., 2019.
Isodon wikstroemioides (Hand.-Mazz.) H. Hara	Aerial parts	Isowikstroemins A–D were evaluated for their cytotoxicity against five human tumor cell lines & exhibited significant activity with IC_{50} values ranging from 0.9 to 7.0 μM. Isowikstroemins A–D and G exhibited inhibitory activity against NO production in LPS-activated RAW264.7 macrophages. Isowikstroemins H, J, & macrocalyxin B were evaluated for their cytotoxicity against human tumor cell lines (HL-60, SMMC-7721, A-549, MCF-7, SW-480), and exhibited significant cytotoxicity with IC50 values ranging from (0.84 ± 0.02) to (4.09 ± 0.34) μmol/ L .	70% aqueous acetone partitioned between ethyl acetate and water.	Wu et al., 2014; 2015.

Species	Plant part(s)	Pharmacology activity	Extract(s)	Reference
		Isowikstroemins H, J, K, & macrocalyxin B exhibited inhibitory activity against NO production in LPS-activated RAW264.7 macrophages.		
Lallemantia royleana (Benth.) Benth.	No identified	Anti-inflammatory activity in rheumatoid arthritis. The natural mucilage, used in the gel, possesses anti-inflammatory properties. suggesting exerting a synergistic action of niosomal gel system at the target site to improve overall effect	Mucilage	Bhardwaj et al., 2020; Bhardwaj & Bhatia, 2020.
Lavandula angustifolia Mill.	Leaves & flowers	Anti-inflammatory effect of the EE with 96% inhibition at 1600 mg/kg, p.o. in the second phase of the formalin test in mouse . FP produced 70% inhibition at 1600 mg/kg, p.o., whereas AE produced 90% inhibition at 200 mg/kg, p.o. Antiaging effect by decreasing MDA levels and increased SOD and GSH-Px at 17.4, 34.8 and 69.6 mg/kg. Downregulation of genes encoding IL-6 & IL-1A in human macrophages infected with *S. aureus*. Effect on ROS production in WBP and human neutrophils. Anti-inflammatory effect in the edema induced with croton oil (0.25-1 mg/ear, by decreasing NO and MPO in Swiss mouse. In the edema induced by carrageenan at 75-250 mg/kg, p.o. & in the dextran-induced edema (75-100 mg/kg, p.o. decreases MPO activity). Reduction of TNF-α, IL-1β, IL-10 at 100-300 mg/kg, i.p. in a rat model of ischemia. Anti-inflammatory effect in the. edema induced with TPA (58.66% inhibition at 100 mg/kg) by decreasing levels of TNF-α, NF-κB, & COX-2. Reduction of mechanical hyperalgesia in chronic inflammatory and neuropathic pain conditions in mouse models. Reduction of proinflammatory cytokines (IL-8, IL-1β and NF-κB) in human THP-1 macrophages induced with LPS. Anti-inflammatory effect in sepsis model in Sprague-Dawley rats by reducing TNF-α (BALF: 64%, serum: 59%; IL-1β (BALF: 63%, serum: 66%)); IL-6 (BALF: 54 %, serum: 59%), as well as 56-76% reduction in induced edema in murine models.	Ethanol extract (EE), polyphenolic fraction (PF), essential oil (AE), aqueous-ethanol extract 75%.	Hajhashemi et al., 2003; Zhao et al., 2015; Giovannini et al., 2016; Georgiev et al., 2017; Cardia et al., 2018; Souri et al., 2019; Chen et al., 2020; Donatello et al., 2020; Slighoua et al., 2022; Pandur et al., 2021; Xie et al., 2022.
Lavandula bipinnata (L.) O. Ktze.	Aerial parts	50% inhibition of COX-2 in human cell lines.	Ethanol extract	Shaikh et al., 2014.
Lavandula coronopifolia Poir.	Aerial parts	Significant inhibition ($p<0.05$) and powerful inhibition in COX-2 (100% at 50, 100, & 200 μg/mL)	Essential oil	Abeer et al., 2020; Nassef et al., 2022.
Lavandula dentata L.	Aerial parts	Griess assay and ELISA to analyze different inflammatory markers.	Methanolic extract	Algieri et al., 2016; Contreras et al., 2018; Abdelhakim et al., 2023.

Species	Plant part(s)	Pharmacology activity	Extract(s)	Reference
Lavandula latifolia Medik.	Aerial parts	Anti-inflammatory effects in the carrageenin-induced edema test.	Essential oils from commercial sources.	Shimizu et al., 1990; Carrasco et al., 2015; Vairinhos and Miguel, 2020; Lucca et al., 2022; Karaka et al., 2023.
Lavandula multifida L.	Aerial parts	A dose-dependent anti-inflammatory effect, where the ethanol extract was the most active. Aqueous extract exerted a significant activity only at 300 and 1000 mg cm^2 (24% and 33% edema reduction, respectively).	Aqueous & ethanol extracts.	Sosa et al., 2005.
Lavandula pubescens Decne.	Aerial parts	It exhibited strong antioxidant (IC_{50} 0.16–0.18 µL/mL), antiacetylcholinesterase (IC_{50} 0.9 µL/mL), antibutyrylcholinesterase (IC50 6.82 µL/mL), & antilipase (IC_{50} 1.08 µL/mL) effects.	Essential oil (EO)	Ali-Shtayeh et al., 2020; Ahmad et al., 2022; Acero et al., 2022.
Lavandula stoechas L.	Aerial parts & flowers.	Anti-inflammatory activity in the edema induced with carrageenan (38% inhibition at 200 mg/kg p.o.). Anti-inflammatory effect on LPS-stimulated macrophages. EE= 74±7 % inhibition at 5-10% b.w. & FP= 85.1 ± 6.2 inhibition at 5-10% b.w. Inhibition of iNOS/NO signaling and gene expression for IL-1β and COX-2 in LPS-induced RAW264.7macrophages.	Methanol extract, essential oil.	Amira et al., 2012; Zuzarte et al., 2013; Zoubi et al., 2016; Kulabas et al., 2018.
Lavandula viridis L'Her.	Aerial parts	A potent effect on ROS inhibition, as well as a strong anti-inflammatory potential due to the modulation of the proinflammatory signaling cascade NF-κB	Essential oil	Skala et al., 2020; Zuzarte et al., 2022.
Lavandula x intermedia	Aerial parts	A dose-dependent anti-inflammatory activity with strong inhibition of NO production even at low concentration (0.03% v/v).	Essential oil	Carbone et al., 2018; Karakas & Matpan 2022.
Lepechinia meyenii (Walp.) Epling	Leaves	Flavonoids showed the best immunomodulating effect. This fraction diminished the superoxide anion production in a concentration dependent manner. A diminution in the expression of TNFα, IL8, & IL17 in mononuclear leukocytes.	Methanol extract 70%.	Arenas-Chávez et al., 2018.
Lycopus lucidus Turcz. ex Benth.	Aerial parts & leaves	The extract decreased the local allergic reaction, passive cutaneous anaphylaxis, histamine release, secretion of TNF-α and IL-6 in mice ICR model. *In vitro* models gathered suggest that the extract could suppress high glucose-induced vascular inflammatory processes.	Aqueous extract	Shin et al., 2005: Lee et al., 2008; Kim et al., 2021; Min et al., 2021.

Species	Plant part(s)	Pharmacology activity	Extract(s)	Reference
		Protection in cultured rat cortical neurons from H_2O_2-induced neuronal injury by significantly inhibiting NLRP3 inflammasome activation (10, 25, and 50 μg/mL). Reduced levels of pro-inflammatory cytokines in a mice model of atopic dermatitis.		
Marsypianthes chamaedrys (Vahl) Kuntze	Aerial parts & leaves	Phospholipase A_2 inhibition at 35 to 70 mg/mL. Anti-inflammatory activity at 2 g/kg) by inhibition of *Evans Blue* diffusion into peritoneal cavities. Inhibition of migration of inflammatory cells.	Aqueous extract	Ruppelt et al., 1991; Magalhães et al., 2011
Melissa officinalis L.	Leaves	Antinociceptive effects in the writhing test (IC_{50}= 241.9 mg/kg, p.o.) & formalin test (inhibition of 33.75 in the 1st phase and 48% 2nd phase at 100 mg/kg) in Swiss mice. Anti-inflammatory effects in the carrageenan-induced pleurisy (60% inhibition of leukocytes and 52% of polymorphonuclear cells 56%) at 200 mg/kg, p.o. in Wistar rats. Anti-inflammatory effects (61.67% inhibition) at 200 mg/kg p.o. EE 63.89% inhibition at 200 mg/kg, p.o. & AC 76.67% inhibition at 100 mg/kg, p.o. in the carrageenan-induced edema test.	Ethanol (EE) & aqueous extracts (AE), essential oil.	Guginski et al., 2009; Müzell et al., 2013; Bohunihi et al., 2013; Zam et al., 2002; Draginic et al., 2022.
Mentha aquatica L.	Aerial parts	Chemopreventive effects against vemurafenib-induced cutaneous side-effects in a carcinogenesis mouse model.	Essential oil	Chang et al., 2019.
Mentha arvensis L.	Whole plant & aerial parts	Inhibition of NO, PGE2, IL-1β, Il-6 in RAW 264.7 macrophages & HaCaT cells induced with LPS. Attenuation of atopic dermatitis in a mouse model. Anti-inflammatory effect in murine induced edema model: leaf= 68.30% inhibition, root= 48.80% inhibition, & stem=10.70% inhibition.	Essential oil & ethanol extract.	Feng et al., 2015; Thawkar et al., 2016; Demirci et al., 2021; Kim et al., 2021b.
Mentha cordifolia Opiz ex Fresen.	Leaves	Menthol: Inhibition of lymphocyte proliferation (88.7% at 50 μg/mL) & IFN-γ inhibition (80% at 800 μg/mL) in PBMCs.	Ethanol extract	Bayat et al., 2019; Villaseñor et al., 2002.
Mentha longifolia (L.) Huds.	Whole plant	Reduces IL-1β, IL-6, TNF-α in mouse models of Alzheimer's. Decreased NO, mRNA for iNOS, & TNF-α in J774.1A macrophages at 0.20 mg/mL. Decreased MPO, PGE2, AST, ALT, & increased GSH and FRAP in a murine model of liver injury. Longifolin A significantly decreased NO levels in RAW 264.7 macrophages. Eucalyptol at 400 mg/kg, p.o. reduced MPO, MDA, TNF-α, & IL-6 levels with an increase in GSH in plasma of rats with acetic acid-induced colitis.	Ethanol extract 70%, hexane extract & essential oil.	Karimian et al., 2013; Ibrahim et al., 2016; Murad et al., 2016; Dadkhah et al. 2018; Moshrefi-Araghi et al., 2021; Wang et al., 2022.

Species	Plant part(s)	Pharmacology activity	Extract(s)	Reference
Menta piperita L.	Leaves & aerial parts	Decreased levels of PLA2, IgE, IL-4, & total protein using 5 μg/mL in Wistar rats pretreated with ovalbumin. Anti-inflammatory effect (40-120 mg/kg, p.o.). Inhibition of TNF-α, IL-6, NO, & PGE2 in RAW 264.7 macrophages induced with LPS.	Aqueous extract-Au-Nps, ethanol extract, & essential oil.	Abdolmaleki et al., 2013; Sun et al., 2014; Li et al., 2017; Yi et al., 2022.
Mentha pulegium L.	Aerial parts & leaves	Protein denaturation, inhibition IC_{50}= 88.31 ± 1.37% v/v. Anti-inflammatory effects in the carrageenan-induced edema (28% reduction at 300 μg/cm^2). Decreased IL-6, MCP-1, & TNF-α in RAW 264.7 macrophages induced with LPS. Anti-inflammatory effect (22.16% inhibition at 12.5 mg/kg, p.o.). in the edema induced by carrageenan in mice.	Essential oil, ethanol & aqueous extracts.	Moussaid et al., 2011; Brahmi et al., 2018; Luis & Domingues, 2021; Messaoudi et al., 2022; Rocha et al., 2019.
Mentha spicata L.	Whole plant & leaves	Decreased jujenal tissue and attenuation of IL-1β and β-glucuronidase in feces. Greater inhibition with the administration of ethyl acetate extract than methanol in granuloma induced in murine models. Where irinotecan-induced mucositis in murine models.	Hexane, ethyl acetate, methanol, chloroform, & aqueous extracts.	el Menyiy et al., 2022.
Mentha suaveolens Ehrh.	No identified	Anti-inflammatory activity in the carrageenan-induced rat paw oedema test (51% of inhibition at 100 mg/kg as compared to the control).	Essential oil & methanol extract.	Moreno et al., 2002; El-Kashoury et al. 2012: Mogosan et al., 2017; Lee et al., 2021.
Mentha x villosa	Leaves	Essential oil and piperitone oxide possess antinociceptive activity probably by an indirect anti-inflammatory effect, which does not involve the central nervous system.	Essential oil	Souza et al., 2009.
Micromeria biflora (Buch. Ham. ex D.Don) Benth.	Aerial parts	Antinociceptive & anti-inflammatory effects in BALB/c mice. Thermal stimulation (delay in reaction time at 2.5-20 mg/kg, p.o.) & edema induced with carrageenan (80.98% inhibition at 15 mg/kg, p.o.).	Chloroform extract & Salicilalazina	Alhojani et al., 2022.
Micromeria croatica (Pers.)	Aerial parts	Reduces expression of TGF-β1 & α-SMA at 400 mg/kg, p.o. in murine models of liver injury.	Ethanol extract 70%.	Vladimir-Knežević et al., 2015.
Minthostachys verticillata (Griseb.) Epling	Aerial parts, leaves, & steams	Significant reduction in the colon weight/length ratio, oxidative stress, & expression levels of IL-1β, iNOS, & COX-2 at 250-500 mg/kg in colitis rat model. Inhibition of β-h release in basophils and lymphocytes from allergic patients. Modulator of the innate immune	Aqueous extract &	Gonzalez-Pereira et al., 2005; Cariddi et al., 2006; Montironi et al., 2019;

Species	Plant part(s)	Pharmacology activity	Extract(s)	Reference
		response in mammary glands of female Balb/c mice challenged with *E. faecium* decreasing the infiltration of polymorphonuclear neutrophils and IL-1β & TNF-α mRNA expression. EO increased the expression of IL-10 in the last hours of infection. Anti-inflammatory activity and potential for managing bovine mastitis.	essential oil (EO).	Rodriguez-Basso et al., 2021; Sharun et al., 2021.
Monarda didyma L.	Aerial parts	Essential oil decreased expression of pro-inflammatory cytokine IL-6 and the increased expression of miR-146a suggesting the involvement of the Toll-like receptor-4 signaling pathway. Where possible bioactive compounds are mainly aromatic monoterpenes & phenolic monoterpenes.	Essential oil	Cotê et al., 2021; Fraternale et al., 2022.
Monarda fistulosa L.	No identified	Anti-inflammatory activity at 100 and 200 mg/kg dosage in the carrageenan-induced rat paw oedema test.	Hydroalcoholic extract	Murata et al., 2013; Shanaida et al., 2021a; Shanaida et al., 2021b.
Mosla chinensis Maxim.	Stem & leaf	In the recipe "Shenshusan", one of its components is *M. chinensis* that showed significant anti-inflammatory activity at 340 mg/kg in mice. *M. chinensis* extract ameliorated inflammation by a 'significant reduction in the production of ROS, increasing the activity of antioxidative enzymes, suppressed the secretion of inflammatory mediators (NO, PGE2), & cytokines (TNF-α, IL-6, IL-1β). The extract also restrained the activation of mitogen-activated protein kinases (MAPKs) signaling pathway.	Essential oil & methanol extract 80%.	Wang et al., 2021; Zhong et al., 2022.
Mosla scabra (Thunb.) C.Y.Wu & H.W.Li	Aerial parts	Anti-inflammatory effects (10, 30 and 90 mg/kg, p.o.) by inhibition of proinflammatory cytokines through the inhibition of MAPK & NF-κB in LPS and ALI (acute lung injury) in mice.	Flavonoid fraction	Chen et al., 2013.
Nepeta bracteata Benth.	Aerial parts	Abietane-type diterpenoids, norabietane-type & ursane-type triterpenoids produced anti-inflammatory effects on the LPS-stimulated RAW 264.7 cells. All compounds significantly down-regulated the TNF-α inflammatory factor, except for nepetabrate J.<br>Amide alkaloid & diterpenoids were elucidated by HR-ESI-MS, NMR, and CD analyses. The anti-inflammatory activity of all compounds was explored using LPS-stimulated RAW 264.7 cells. It is worth noting that all were able to inhibit NO production with IC_{50} values < 50 μM and little effect on RAW 264.7 macrophage viability. Remarkable inhibition with IC_{50} values of 19.2 & 18.8 μM, respectively. Meanwhile, screening on HCT-8 cells demonstrated that nepetabrates B and D also had moderate cytotoxic activities with	Ethanol extract 95%.	Zhang et al., 2021; Yang et al., 2022.

Species	Plant part(s)	Pharmacology activity	Extract(s)	Reference
		IC_{50} values of 36.3 & 41.4 μM, respectively, which is related to their anti-inflammatory effects.		
Nepeta cataria Benth.	Aerial parts	Verbascoside produced inhibition of calcineurin activity in an *in vitro* assay with a IC_{50}= 4.5 μM. Ant-inflammatory effects were explored in tail immersion test and acetic acid-induced nociception in mice.	Essential oil	Modnicki et al. 2007; Prescott et al., 2011; Pargaien et al., 2020; Acimovic et al., 2022.
Nepeta crispa Willd.	Aerial parts	Antinociceptive effects explored in the tail-flick test (54.7% inhibition at 200 mg/kg, i.p.), formalin test (30-200 mg/kg significantly reduced nociceptive behavior in the neurogenic and inflammatory phases).	Essential oil	Ali et al., 2012.
Nepeta deflersiana Schweinf. ex Hedge	Aerial parts	Ethyl acetate & n-butanol extracts showed a decrease in oxidative stress and inhibition of both NF-kB and iNOS activities with no cytotoxic effects on four human cancer cell lines (SK-OV-3, SK-MEL, KB, and BT-549). In contrast, there is moderate cytotoxic action against one of the two tested noncancerous kidney cell lines (LLC-PK1) with an IC_{50} of 16 and 55 μg/ml for ethyl acetate and chloroform extracts, respectively. The oral administration of ethanolic extract (50 & 100 mg/kg, b.w) downregulated the expression of pro-inflammatory cytokines (TNFα, IL-6, & IL-10) and apoptotic markers (caspase-3 and Bax). It upregulated the anti-apoptotic protein Bcl2. Furthermore, NDEE pretreatment significantly downregulated cardiac NF-κB (p65) expression, NF-κB-DNA binding activity, and MPO activity. Histological data showed that pretreatment reduced myonecrosis, edema, and infiltration of inflammatory cells and restored the architecture of cardiomyocytes.	Petroleum ether, chloroform, ethyl acetate, n-butanol, ethanol extracts.	Al Taweel et al., 2017; Orfali et al., 2018; Ahmad et al., 2022.
Nepeta dschuparensis Bornm	Leaves	Reduction on the infarct volume and a significant decrease in the level of IL-β in a model of cerebral artery occlusion and on motor coordination disorder induced by cerebral ischemia in Sprague-Dawley rats.	Ethanol extract	Mousavi et al., 2017.
Nepeta laxiflora Benth.	Aerial parts	Decreased NO in PC12 cells treated with H_2O_2 (50% inhibition at 50-200 μg/mL).	Methanol extract	Khankandi et al., 2019.
Nepeta menthoides Boiss. & Buhse	Aerial parts	It was explored in the formalin test, xylene test, & hot water tail-immersion test at doses of 70, 350, 700, 1400, & 2800 mg/kg. It was observed to have anti-inflammatory effects at all doses, particularly at a dosage of 2800 mg/kg. As well as in the anti-nociceptive effect in chronic phases. Pretreatment with this ethanolic extract attenuated the development of chronic morphine dependence.	Hydroalcoholic & ethanol extracts.	Memariani et al., 2018; Süntar et al., 2018.

Species	Plant part(s)	Pharmacology activity	Extract(s)	Reference
Nepeta meyeri Benth.	Aerial parts	Acetone extract exhibited a significant concentration-dependent inhibition of DPPH and NO radical. Furthermore, *N. meyeri* showed very high reducing power. In DPPH radical and NO• scavenging assays the IC_{50} value of extract was 672.2 μg/ml and 165.32 μg/ml, respectively. The data obtained from these *in vitro* models clearly demonstrated antioxidant potential of acetone extract of *N. meyeri*.	Acetone extract & essential oil.	Cigremis et al. ,2010; Süntar et al., 2018.
Nepeta pogonosperma Jamzad & Assadi	Aerial parts	Essential oil reduced the pain sensation in tail flick and formalin test in both phases (200 mg/kg). In paw edema test significantly reduced the inflammation at 100 and 200 mg/kg.	Essential oil	Ali et al., 2012; Khalighi-Sigaroodi et al., 2013.
Nepeta tenuifolia Benth.	No identified	Inhibition of MAPK activation. Anti-inflammatory activity via TLR4 inhibition, which led to MAPK and NF-κB-mediated signaling in LPS-stimulated macrophages. LPS/IFN-γ in RAW264.7 macrophage cells. could reduce the expression of the NLRP3, iNOS, p65, IL-1β mRNA and P2X7R proteins and significantly reduce NO level.	Essential oil	Shan et al., 2021.
Ocimum basilicum L.	Roots	Root extract decreased levels of proinflammatory cytokines in mouse models with induced asthma. Linalool decreased the levels of proinflammatory cytokines in RAW 264.7 macrophages induced with LPS.	Organic & aqueous extracts.	Aminian et al., 2022.
Ocimum kilimandscharicum Baker ex Gürke	Aerial parts	Immunostimulatory effect in fish *Clarias batrachus.* Inhibited the carrageenan-induced edema (100 and 300 mg/kg), mechanical and cold hyperalgesia. Inhibited the carrageenan induced pleurisy reducing the migration of total leukocytes in mice by 82% at a 30 mg/kg. Camphor inhibited all articular parameters induced by zymosan in mice (knee edema, leukocyte infiltration, mechanical hyperalgesia, and NO) at 30 mg/kg. Inhibited the carrageenan induced pleurisy reducing the migration of total leukocytes in mice by 83% at a dosage of 15 mg/kg. A mixture of 1,8 cineole/limonene 1:1: inhibited the carrageenan induced pleurisy reducing the migration of total leukocytes in mice by 80% at 6 mg/kg.	Essential oil & aqueous extract.	de Lima et al., 2014; Nahak & Kanta, 2014; dos Santos et al., 2021.
Ocimum labiatum (N.E. Br.) A.J. Paton	Leaves	*O. labiatum* extract at a 25 μg/mL non-cytotoxic concentration of extract significantly inhibited the production of pro-inflammatory cytokines, such as IL-2, IL-4, IL-6, and IL-17A. Except for the dual acting pro- or anti-inflammatory cytokine, IL-6, which was upregulated, a non-cytotoxic 50 μM concentration of the isolated labdane diterpenoid compound significantly decreased the production of all the pro-inflammatory cytokines. In the anti-inflammatory pathway studies, the compound also inhibited AP-1 significantly at 50 μM. The extract demonstrated strong dose dependent antioxidant activity with IC_{50}	Ethanol extract	Kapewangolo et al., 2015; Lambrechts & Lall, 2019.

Species	Plant part(s)	Pharmacology activity	Extract(s)	Reference
		values ranging from 13 ± 0.8 to 54.86 ± 1.28 μg/mL, while the terpene had no antioxidant property. The extract and diterpenoid decreased the production of the inflammatory mediator NO, at non-cytotoxic concentrations. The CE_{50} of the extract in TZM-bl and PBMCs was 62.6 ± 0.6 and 30.1 ± 0.4 μg/mL, while that of the compound was 112.6 ± 0.2 and 70 ± 0.4 μM, respectively.		
Ocimum sanctum L.	Aerial parts	Aqueous and ethanol extracts decrease epithelialization period in lesion models in rats. Decreases the percentage of inflammation in rat models of edema induced with carrageenan and croton oil at doses of 500 mg/kg, p.o. Apigenin, rosmarinic acid, isotimusin, isothymonin, cirsimaritin, cirsilineol, eugenol show COX-1 inhibition in *in vitro* models with different percentages of inhibition at a concentration range of 10-1000 μM.	Organic & aqueous extracts.	Pattanayak et al., 2010; Singh et al., 2018.
Ocimum selloi Benth.	Leaves	inhibited the carrageenan-induced pleurisy in female Swiss mice at 30, 100, and 300 mg/kg.	Aqueous extract, essential oil.	Moraes et al., 2002; Piva et al., 2021.
Origanum spp.	Aerial parts	Essential oil lowering TNF-α levels in bronchoalveolar fluid of LPS-induced mice. Decreased mRNA of TNF-α, IL-1B, IL-6, MCP-1, & IFN-γ in the jejunum of pigs fed for 28 days with essential oil and induced with endotoxins. Essential oil-NPs decreased NO levels in RAW 264.7 macrophages induced with LPS. Decreased levels of IL-8, IL-1B, & TNF-α in *in vivo* and *in vitro* models of inflammation induced by *Propionibacterium acnes*. Ethanol-water extract protected against hepatotoxic damage in rat models. Ethanol extract at doses of 100-5000 mg/kg, p.o. improved the symptoms of colitis in rats.<br>*O. syriacum* methanol extract at concentration of 10-100 μg/mL decreases IL-6 & IL-10 levels in peripheral mononuclear cells.<br>*O. tyttanthum* essential oil shows inhibition of pro-inflammatory enzymes in *in vitro* models. Carvacrol and thymol exhibited anti-inflammatory effects in *in vivo*, *in vitro,* and clinical studies.<br>*O. microphyllum* infusion produced cell growth inhibition associated with decrease in IL-8 levels in the HT29 colon and in PC3 prostate cancer cells lines. Even though the anti-inflammatoty activity was not correlated with total phenolic contents, but the antioxidant activity was positively correlated with total phenolics in all herbal infusions.<br>*O. sipyleum* can be considered as a good source of phenolic compounds such as rosmarinic acid and phlorizin. These compounds are considered as pro-oxidant/pro-inflammatory	Organic (essential oil, ethyl acetate, ethanol, or methanol) & aqueous extracts.	Kogiannou et al., 2013; Zengin et al., 2019; El kharraf et al., 2020; Hamamouchi et al., 2021; Sharifi-Rad et al., 2021; Al-Mijalli et. al., 2022.

Species	Plant part(s)	Pharmacology activity	Extract(s)	Reference
		biomarkers induced by the extracts, in rat colon challenged with LPS, together with the inhibition of selected bacterial and fungi strains involved in ulcerative colitis and the antiproliferative effect exerted only by ethyl Acetate extract, further support protective effects in inflamed colon as well.<br>*O. compactum* Benth. produced anti-inflammatory activity in Wistar rats in tail-Flick test and in the writhing test in OF1 mice at 100 mg/kg.<br>Anti-inflammatory effect using 5-lipoxygenase inhibition test (IC_{50}= 0.68 ± 0.02 μg/mL) and carrageenan-induced paw edema model (56.53% inhibition of edema at 100 mg/kg, p.o.).		
Orthosiphon aristatus (Blume) Miq.	Aerial parts & leaves	Anti-inflammatory effect in *in vitro* model. Inhibition of 3,5-U-hyaluronase (19.38% at 10 μg/mL). Inhibition of NO, PGE_2, & ROS in RAW 264.7 macrophages induced with LPS.	Ethanol extract	Hsu et al., 2010; Vijayan et al., 2017.
Orthosiphon stamineus Benth.	No identified	Anti-inflammatory effect of EM in mouse models at 250-1000 mg/kg, p.o. Anti-inflammatory effects at 500-1000 mg/kg, p.o., in the edema induced with carrageenan. Orthosifol A and B produced strong anti-inflammatory activity against tumors induced in murine models. Antipyretic effect of EE at 50-1000 mg/kg, p.o., in murine models induced with yeast.	Ethanol extract (EM), chloroform extract (EC), ethanol extract (EE).	Singh et al., 2015.
Perilla frutescens (L.) Britton	Leaves & seeds	Reduction of the mRNA expression of IL-6, IL-8, TNF-α, COX-2, & iNOS in RAW 264.7 macrophages induced with LPS. 12-Tetradecanoylphorbol 13-acetate (TPA)-induced edema (50% inhibition at ID_{50}=0.09-0.3 mg/per ear). Ursolic acid, corosolic acid, 3-epicorosolic, pomolic acid, stormy acid, hyptadienic acid, oleanolic acid, augustic acid, & 3-epimaslinic acid produced anti-inflammatory effects. Reduction of body weight loss, diarrhea, and bleeding at 20-200 mg/kg, p.o., in mouse models of colitis. Involvement of luteolin by inhibition of edema induced with TPA. Monogalactosyldiacylglycerols inhibited NO, TNF-α, & IL-6 in RAW 264.7 macrophages induced with LPS. Rosmarinic acid (RA) reduced inflammation and oxidative stress and reduced tumor size in skin cancers induced with 7,12-dimethylbenz[a]anthracene (DMBA). The anti-inflammatory targets of RA in tumor therapy are cyclooxygenase-2 (COX-2) and NF-κB. RA inhibited COX-2 activity and downregulated ERK1/2 to exert anti-inflammatory effects in lung, breast, and liver cancer cells. Inflammatory factors, including TNF-α, IL-1β, IL-6, &	Ethanol, methanol, & aqueous extracts. Essential oil.	Banno et al., 2004; Huang et al., 2014; Ueda et al., 2002; Zuo et al., 2002; Liu et al., 2022b; Kangwan et al., 2021; Zhao et al., 2022.

Species	Plant part(s)	Pharmacology activity	Extract(s)	Reference
		transforming growth factor-β (TGF-β), were reduced after anti-tumor therapy with RA. The anti-inflammatory effect of RA in tumorigenesis has been related to the inhibition of TLR4/NF-κB & STAT3. RA was shown to suppress CRC inflammation by impairing the IL-6/STAT3 and NF-κB pathways. In hepatocellular carcinoma (HCC), H22 tumor-bearing mice were treated with intraperitoneal injection of RA 75, 150, and 300 mg/kg. Inhibition of inflammatory cytokines (IL-1β, IL-6, TNF-α, TGF-β), angiogenic factors (VEGF), & phosphorylation of p65.		
Platostoma africanum P. Beauv.	Aerial parts	Anti-inflammatory effects of EH produced 45.19% inhibition at 400 mg/kg and ED= 76.10% inhibition at 400 mg/kg in the ovalbumin-induced edema in rats.	Hexane extract (EH) & dichlorometha ne extract.	Aladedunye et al., 2008.
Plectranthus amboinicus (Lour.) Spreng	Leaves	At 500 mg/kg b.w. increased concentrations of the cytokine TGF-β1 and GPx in Wistar rats.	Ethanol extract 90%.	Solfaine et al., 2021.
Plectranthus barbatus Andrews	Leaves	Attenuation in the production of IL-2, IL-6, IL-10, TNF, & IL-17A at 25 μg/mL in mononuclear cells infected with HIV-1.	Ethanol extract	Kapewangolo et al., 2013.
Plectranthus caninus Vatke	Aerial parts	Anti-inflammatory effects in the edema induced with carrageenan (38.96 ± 16.59% inhibition at 300 mg/mL) in Swiss mice.	Essential oil	Tadesse et al., 2011.
Plectranthus ecklonii Benth.	Aerial parts	NO inhibition in RAW 264.7 macrophages induced by LPS at 100 μM. Parvifloron D, a mixture of β-sitosterol & stigmasterol (1:1) were involved in the anti-inflammatory effect.	Parvifloron D, β-sitosterol, & stigmasterol	Andrade et al., 2018.
Plectranthus ornatus Codd.	Aerial parts	Molecular docking shows potential anti-inflammatory effect for 1,6-di-O-acetyl-9-deoxyforskolin docked to IL-6 (−12.16 kcal/mol), TNF-α (−15.18 kcal/mol) and GM-CSF (−13.84 kcal/mol) and for 1,6-di-O-acetylforskolin docked at IL-8 (−16.26 kcal/mol). *In vivo* studies demonstrated that oral administration of forskolin (10 mg/kg) exerts a strong anti-inflammatory effect by reducing paw swelling (87.79%), comparable to the standard drug indomethacin (10 mg/kg, 93.89%). The labdane diterpene inhibited NO production by LPS in RAW 264.7 cells with an IC_{50} value of 40.1 μM. (11R*,13E)-11-acetoxyhalima-5,13-dien-15-oic acid, 1α,6β-diacetoxy-8α,13R*-epoxy-14-labden-11-one and 1,6-di-O-acetylforskolin		Sitarek et al., 2022.

Species	Plant part(s)	Pharmacology activity	Extract(s)	Reference
		& 1,6-di-O-acetyl-9-deoxyforskolin possess anti-inflammatory effect in LPS-induced ARPE-19 cells by reducing the level of expression of IL-6, IL-8, TNF-α and GM-CSF genes.		
Plectranthus zeylanicus Benth.	Whole plant	5-lipoxygenase (5-LO) activity was potently suppressed (inhibition >85%), in A23187-stimulated human neutrophils (IC_{50} = 6.6 & 12 μg/mL, respectively), & inhibited isolated human recombinant 5-LO (IC_{50} = 0.7 & 1.2 μg/mL, respectively). Inhibition of 5-LO activity with IC_{50} values of 1.3 μg/mL in cell-free assay using isolated human recombinant 5-LO; and 5.1 μg/mL in cell-based assays (human neutrophils challenged with A23187 plus exogenous arachidonic acid).	Hexane & dichlorometha ne extracts.	Napagoda et al., 2014.<br>Napagoda et al., 2022.
Prunella laciniata L.	Aerial parts	*In vitro* NO production by LPS-stimulated murine peritoneal macrophages of 6 weeks old mice with two aqueous extracts at a concentration of 400 μg/mL was significantly inhibited (1.501 and 0.347 μg/mL, respectively) without affecting cell viability.	Aqueous extract	Sebnem-Harput et al., 2006.
Prunella vulgaris L.	Aerial parts; leaves and stem; whole plant; fruit spikes	Anti-inflammatory effect in the model of inflammation induced with TNF-α in HASMCs and in the model of ovalbumin-induced conjunctivitis in rats. Inhibition of IL-1β, caspase-1, & NLRP3 in corneal tissue. Participation of 2α,3α,23-trihydroxyursa-12,20(30)-dien-28-oic acid, β-amyrin & eusapic acid at 46.7, 57.9, & 54.2% suppression of histamine in *in vitro* models, respectively. Inhibition of NO, IL-6 and PEG2, iNOS, COX-2, TNF-α mRNA in RAW 264.7 macrophages induced with LPS. Anti-inflammatory effect in rats. Dimethylbenzene-induced oedema (25 % inhibition).<br>2α, 3α-dihydroxyursolic acid. Anti-in flammatory effect. Decreased β-hexosaminidase in RBL-2H3 cells (IC_{50}=57μM).<br>Prunelanato A y Pruneladiterpenol A: Decrease of NO in RAW 264.7 macrophages induced with LPS. (IC_{50}=6.77 y 8.61 μM). Significant enhancement of procollagen type I mRNA, TNF-α and IL-6 levels in UVB-activated MMPs.	Methanol, ethanol 50% & aqueous extracts. Hexane fraction from ethanol extract, & essential oil.	Park et al., 2013<br>Li et al., 2020<br>Choia et al., 2016<br>Hwang et al., 2013<br>Yan, 2016<br>Tang et al., 2022<br>Ryu et al., 2000<br>Zheng et al., 2022<br>Zhang et al., 2018b.
Pycnostachys reticulata (E. Mey.) Benth.	Roots	A 68.7% inhibition of the COX-2 at 0.25 μg/μL in an *in vitro* test.	Methanol extract 50%.	Fawole et al., 2010.

Species	Plant part(s)	Pharmacology activity	Extract(s)	Reference
Salvia aegyptiaca L.	Whole plant	Decreased temperature in a model of hyperthermia induced in mice at 0.25-2 g/kg.	Acetone extract.	Al-Yousuf et al., 2002.
Salvia aethiophis (Kunth) Lint. & Epling	Roots	Inhibition of 5-lipoxygenase activity in an *in vitro* assay with human neutrophils. Reduction in the leukocyte accumulation & inhibition of cytokines. Participation of luteolin & apigenin.	Acetone extract.	Hernández-Pérez et al., 1995; Benrezzouk et al., 2001; Nworu and Akah, 2015; Vulganová et al., 2019.
Salvia africana-caerulea L.	Aerial parts	Ability in scavenging NO. Activator of the human peroxisome, proliferator-activated receptor gamma, inhibitor of the 5-lipoxygenase enzyme.	Aqueous, methanol, & chloroform extracts.	Komatou et al., 2006; Kamatou et al., 2010; Bonito et al., 2011; Afonso et al., 2019.
Salvia albicaulis Benth.	Aerial parts	5-lipoxygenase (5-LO) activity was inhibited by 50% (IC_{50}) when adding the 5-LO enzyme and the absorbance arising from the modification. It may be attributed to the high percentage of sesquiterpenes, such as the linoleic acid (diene 1–4 converted into diene 1–3). IC_{50} 39±4 & 65±6 μg/ml, respectively.	Essential oil .	Kamatou et al., 2007.
Salvia apiana Jeps.	Aerial parts	The potency to counteract NO production was evaluated in an LPS-activated RAW 264.7 macrophage model and its potency corresponded to about one-third of the drug dexamethasone (EC_{50} = 49.9 ± 2.5 μg/mL & 16.0 ± 1.0 μg/mL, respectively).	Aqueous extract	Afonso et al., 2019.
Salvia ceratophylla L.	Aerial parts	Inhibition of NO production and NF-κB activation, as well as in the edema induced using an *in vivo* asssay.	Hexane, ethyl acetate, & methanol extracts.	Shehadeh et al., 2014; Bonesi et al., 2017; Abu-Darwish et al., 2020.
Salvia chamelaeagnea Bergius	Aerial parts	5-lipoxygenase assay was used as an indication of the anti-inflammatory activity. The extract displayed poor inhibition of the 5-lipoxygenase enzyme, with IC_{50} greater than 100 μg/mL. Essential oil exhibited promising anti-inflammatory activity by inhibiting the 5-lipoxygenase enzyme with IC_{50} values from 43.4 ± 3.5 to 77.3 ± 5.2 μg/mL. Compounds such as 1,8-cineole displayed anti-inflammatory activity and monoterpenes such as *α*-pinene and sesquiterpenes & β-caryophyllene inhibited the *in vitro* 5-lipoxygenase activity.	Methanol-chloroform extract, essential oil.	Kamatou et al., 2006; Kamatou et al., 2010.
Salvia chudei Batt. & Trab.	Aerial parts	Anti-inflammatory activity in the edema induced with carrageenan showing a 58% inhibition at 250-1000 mg/kg, b.w.	Aqueous extract	Semaoui et al., 2021.

Species	Plant part(s)	Pharmacology activity	Extract(s)	Reference
Salvia desoleana Atzei & P. Picci	Aerial parts & leaves	Inhibition of carrageena n- and histamine- induced oedema in rats.	Essential oil	Peana & Satta, 1993; Peana et al., 1999; Ceschel et al., 2000; Peana & moretti, 2002.
Salvia digitaloides Diels	Roots	Inhibitory effects against the release of both O_2 and elastase by human neutrophils, with IC_{50} values of 48.98 ± 1.60 & 25.22 ± 4.41 μM, respectively. These results suggested that Salviatalin A may represent a good candidate as a new anti-inflammatory agent.	Methanol extract	Shwu-Jen et al., 2010.
Salvia dolomitica Codd.	Aerial parts	Inhibition of 5-lipoxygenase in an *in vitro* assay with a IC_{50}: 65±6 μg/mL.	Essential oil	Kamatou et al., 2007.
Salvia dominica L.	Leaves & root s	The anti-inflammatory activity of sesterterpenes was observed.	Acetone extract	Dal Piaz et al., 2010.
Salvia frigida L.	leaves	Reduction of proinflammatory cytokine levels and improvement of induced dermatitis in mice after topical administration.	Phenolic fraction	Hassan et al., 2022.
Salvia fruticosa Mill.	Root & aerial parts	Anti-inflammatory effect in the carrageenan-induced edema (44% inhibition at 200 mg/kg, i.p. in rats.	Methanol extract	Boukhary et al., 2016.
Salvia glutinosa L.	Aerial parts	Reduced NO production in LPS-treated RAW 264.7 cells with an IC_{50}= 304.93±11.19 μg/mL.	Ethanol extract 70%.	Nicolescu et al., 2022.
Salvia hierosolymitana Boiss.	Aerial parts	The topical anti-inflammatory activity was evaluated as inhibition of the Croton oil-induced ear oedema in mice. Inflammatory response was induced on the inner surface of the right ear (surface: about 1 cm^2) by application of 80 μg of Croton oil dissolved in vehicle. At a dose of 300 μg/cm^2. The extracts reduced oedema by involving 3b,23-dihydroxyurs-12-en- 28-oic acid, 2a,3b-dihydroxyolean-12-en-28-oic & maslinic acid, 2a,3b,23-trihydroxyolean-12-en-28-oic or arjunolic acid were the most active with 54%, 51%, & 60%, respectively.	Petroleum ether, chloroform, chloroform:met hanol (9:1), & methanol extracts.	De Felice et al., 2006; de Souza et al., 2021.
Salvia hypoleuca DC. ex Benth.	Leaves & fruits	Inhibition of the carrageenan induced paw edema. Prevented NO production in the aqueous humor and the expression of the iNOS enzyme in a dose-dependent manner.<br>Inhibited the development of EIU and suppressed LPS-induced iNOS expression.<br>Inhibited the release of NF-κB-dependent cytokines including TNF-*α* and IL-6. Opioid system and inhibition of proinflammatory mediators.	Ethanol extract	Estakhr & Javdan, 2011; Estakhr & Javdan, 2011a; Javdan & Estakhr, 2011b; Parsaei et al., 2016.

Species	Plant part(s)	Pharmacology activity	Extract(s)	Reference
Salvia japonica Thunb.	Aerial parts	The anti-inflammatory activities were measured via the mice ear edema induced by TPA. Ibuprofen was used as a positive control, EO was diluted with peanut oil in BALB/c mice. The optimum dose of EO was 100 mg/kg, which produced 31.15 ± 1.38% of swelling-inhibition. The EO was like ibuprofen in the degree of reduction of COX-2, NF-κB (p65), & IL-6, and better than ibuprofen in the degree of reduction of TNF-α. Exhibited promising anti-inflammatory activity by inhibiting the expression of IL-1, IL-6, TNF-α, and COX-2 in rats with adjuvant arthritis. At a concentration of 100 mg/kg exhibited inhibitory effects on arthritis similar to ibuprofen.	Essential oil (EO)	Li et al., 2021.; Sun et al., 2022.
Salvia lachnostachys Benth.	Leaves	The anti-inflammatory effects of the EE & fruticulin A were evaluated in mice using experimental models of inflammation (paw oedema & pleurisy induced by carrageenan injection). Oral administration of 30, 100, and 300 mg/kg and fruticulin A (0.3 and 3.0 mg/kg) decreased the total leucocytes number in pleural lavage, protein extravasation, and paw oedema in a dose-dependent manner. Fruticulin A significantly decreased paw oedema in mice at doses of 1.0 and 3.0 mg/kg, with no significant difference between the doses. In the pleurisy test, the number of total leukocytes decreased significantly in mice treated with 30, 100, or 300 mg/kg compared to the control group, with no significant difference between doses.	Ethanol extract	Piccinelli et al., 2014.
Salvia lanceolata Lam.	Leaves	Exhibited poor anti-inflammatory activity with a IC_{50} >100µg/mL against 5-lipoxygenase (5-LO).	Methanol-chloroform (1:1) extract.	Kamatou et al., 2010.
Salvia lanigera Poir.	Leaves	The *in vitro* anti-inflammatory potential of the extract was evaluated by determining the membrane stabilization of human red blood cells and the percent inhibition of the COX1/2, 5LOX, and sPLA2-V enzymes. The extract showed maximum membrane stabilization (≤ 91%, at 100 µg/mL) and the reference drug diclofenac sodium (90.75%). Strong inhibition of 5-LOX and COX-1 enzymes at 100 µg/mL. At the concentration of 100 µg/mL, the membrane stabilization was 92.10 ± 1.27%, and very close to that of diclofenac sodium (90.75 ± 1.06%).	Ethanol extract	Alonazi, et al., 2021.

Species	Plant part(s)	Pharmacology activity	Extract(s)	Reference
Salvia leriifolia Benth.	Aerial parts, leaves & seeds	Anti-inflammatory effect in the xylene or acetic acid induced edema in rats using 2.5-5 g/kg. NO inhibition in RAW 264.7 macrophages induced with LPS with an IC_{50}= 165 µg/mL.	Aqueous extract & essential oil.	Hosseinzadeh & Yavary, 1999; Loizzo et al., 2009; Hosseinzadeh et al., 2003; Modarres et al., 2014.
Salvia macilenta Boiss.	Aerial parts	Anti-inflammatory effects in murine models induced with beta-amyloid at 50 mg/kg/10 days, p.o.	Methanol extract	Taheri et al., 2021.
Salvia miltiorrhiza Bunge	Roots	Tanshinone IIA: Downregulation of proinflammatory cytokines in *in vitro* models. It was effective against allergies, asthma, and rhinitis in clinical models. While in murine models an attenuation of proinflammatory cytokines was observed. It presented anticancer properties through reduction of proinflammatory cytokines in murine models and relief against fibrosis in liver, heart, lung, kidneys using high polarity extracts as well as from isolated compounds in *in vitro* and *in vivo* models.		Feng et al., 2021; Mahalaksmi et al., 2021; Yang et al., 2022.
Salvia mirzayanii Rech. f. & Esfand.	Aerial parts	A 66.2 ± 8% inhibition of NO at 50 µg/mL in LPS-treated RAW 264.7 cells. The LPS-induced DNA binding activity of NF-κB in THP-1 was significantly decreased by teuclatriol (312 & 390 mM). Teuclatriol reduced the production of TNF-α in a dose-dependent manner.	Methanol extract	Zarshenas et al., 2014; Ziaei et al., 2015; Amirghofran et al., 2011.
Salvia moorcroftiana Wall. ex Benth.	Whole plant	Anti-inflammatory and antipyretic effects at 300 mg/kg, p.o., in murine models.	Methanol extract	Hussain et al., 2017.
Salvia muirii (L.) Bol.	Aerial parts	Exhibited poor anti-inflammatory activity with an IC_{50} >100 µg/mL against 5-LO.	Methanol:chloroform (1:1) extract.	Kamatou et al., 2006; Kamatou et al., 2010.
Salvia multicaulis Vahl	Aerial parts	Topical anti-inflammatory activity against Croton oil-induced edema in CD-1 mice (47 – 300 µg/cm^2).	Hexane & ethyl acetate extracts.	Shehadeh et al., 2014; Rowshan & Najafian, 2020.
Salvia nipponica Miq.	Root & leaves	Elastase inhibitory activity in human neutrophils induced with formyl-methionyl-l-leucyl-l-phenylalanine/cytochalasin B.	Methanol extract	Chan et al., 2011.

Species	Plant part(s)	Pharmacology activity	Extract(s)	Reference
Salvia officinalis L.	Leaves	Anti-inflammatory properties in murine models, whose main component involved in the effect was ursolic acid.	Organic and aqueous extracts.	Miraj & Kiani, 2016.
Salvia palaestina Benth.	No identified	An *in vitro* anti-inflammatory potential at a concentration of 100 μg/mL using the LO enzyme inhibition assay. The highest inhibition rates were observed with an IC_{50} values of SP-EtOAc and SP-CH2Cl determined as 93.37 and 83.25 mg/mL, respectively. An IC_{50} values of rosmarinic acid (78% LO inhibitory activity) and methyl rosmarinate (96% LO inhibitory activity) were calculated as 0.21 and 0.02 μM, respectively.	Fractionation of the methanol extract, dichlorometha ne (SP-CH2CL2) & ethyl acetate (SP-EtOAc).	Sina-Içen et al., 2021.
Salvia petrophilla G. X. Hu, E. D. Liu & Yan Liu	Whole plant	Inhibición de NO en macrófagos RAW 264.7 inducidos con LPS IC_{50} = 29-40 μM.	Acetone extract	Zou et al., 2022.
Salvia plebeia R. Br.	Leaves & aerial parts	Inhibition of TNF-α, IL-5, & NO in RAW 264.7 macrophages induced with LPS. Inhibition of eosinophils, IL-4, IL-13, & mucus production in BAL B/c mouse model of asthma. Decreased NO & iNOS mRNA expression in RAW 264.7 macrophages induced with LPS. 8-epi-eudebeiolide C inhibited NO & mRNA expression for iNOS in RAW 264.7 macrophages induced with LPS with an IC_{50}= 17.9 μM. Salviplenoide A inhibited NO, iNOS, COX-2, & NF-κB mRNA expression in LPS-induced RAW 264.7 macrophages. Luteoloside, nepitrin, homoplantagenin, luteolin, nepetin, hispidulin, & eupatorine inhibited NO, PGE2, & iNOS mRNA expression.	Ethanol & methanol extracts.	Jeong et al., 2012; Akram et al., 2015; Jang et al., 2016; Jang et al., 2017; Zou et al., 2018.
Salvia prionitis Hance	Aerial parts	All compounds were evaluated for their inhibitory effects on NO production stimulated by LPS in RAW264.7 mouse peritoneal macrophages cell. L -NMMA (NG-Monomethyl- L -arginine, Monoacetate Salt) was used as a positive control with an IC_{50} =32.55 μM. Prionidipene and salvilenone showed potent inhibitory activity with the IC_{50} = 14.56 & 15.11 μM, respectively.	Organic extracts	Li et al., 2018.

Species	Plant part(s)	Pharmacology activity	Extract(s)	Reference
Salvia radula Benth.	Aerial parts	Displayed moderate anti-inflammatory activity with an IC_{50} = 78.8 ± 5.89 μg/mL against 5-LO.	Methanol-chloroform (1:1) extract.	Kamatou et al., 2010.
Salvia repens Burch. ex Benth.	Aerial parts	Poor anti-inflammatory activity with an IC_{50} >100 μg/mL against 5-LO.	Methanol-chloroform (1:1) extract.	Kamatou et al., 2010.
Salvia rosmarinus (L.) J.B. Walker, B.T. Drew & J.G. González (Syn. *Rosmarinus officinalis* L.)	Aerial parts	Essential oil reduced the paw edema similarly to the indomethacin treated group (positive control, 10 mg/kg, p.o) in a dose-dependent manner with an ED_{50}= 300 mg/kg. Granulomatous tissue formation was inhibited by 59% compared to the negative control group (saline solution, 0.5 ml, p.o), and ear edema was inhibited by 77%. A Significant inhibition in the carrageenan-induced edema formation 1–4 h after carrageenan treatment, also, significantly reduced the volume of pleural inflammatory exudate and the number of migrated cells. Inhibition of arachidonic acid cascade enzymes by the presence of 1,8-cineole & inhibition of NF-κB by some monoterpenes. Carnosic acid was a potent NO inhibitor at low concentrations (6.2 μg/mL). A reduced skin lesions in mice with atopic dermatitis and reduced the amounts of immunoglobulin E, neutrophils, and inflammatory cytokines in the blood, as well as reduced NO production in LPS-treated RAW 264.7 cells.	Essential oil	Sousa et al., 2019; Malvezzi et al., 2020.
Salvia runcinata L. f.	Aerial parts	5-LOX and 5 LO inhibitors.	Methanol extract; methanol:chloroform extract. Essential oil.	Komatou et al., 2005; Kamatou et al., 2008; Kamatou & Viljoen, 2010; Komatou et al., 2010a.
Salvia sagittata Ruiz & Pav.	Aerial parts	Treatment of the EE did not affect the cell viability, while it showed a remarkable ability to reduce LPS-induced production of proinflammatory cytokines and increase the expression of HO-1 in the primary cultures of porcine aortic endothelial cells. It is possible to hypothesize that the cinnamic acid derivative gives the main contribution to the anti-inflammatory effects of the extract.	Ethanol extract (EE)	Mahmoud, et al., 2019; Tubon et al., 2019.
Salvia sclarea L.	Aerial parts & leaves.	Inhibition of IL-1β, IL-6, & TNF-α in rat model of periodontal inflammation at 200 mg/kg, p.o., and in the carrageenan-induced edema (73% inhibition at 250 mg/kg, s.c.). Sclareol reduced the expression of markers of myocardial injury at 5 mg/kg/day in C57BL/6 mice.	Essential oil & ethanol extract.	Moretti et al., 1997; Zhong et al., 2015; Kostic et al., 2017; Yang et al., 2022.

Species	Plant part(s)	Pharmacology activity	Extract(s)	Reference
		Diminution in the expression levels of MMPs, iNOS, & COS-2 in isolated rabbit chondrocytes at 10 μg/mL, as well as in the cartilage degradation in rabbit osteoarthritis model.		
Salvia sclareoides Brot.	Aerial parts	Moderate inhibition of COX-1 in an *in vitro* assay with an IC_{50} = 271.0 ± 4.0 μg/mL.	Aqueous extract	Batista et al., 2017.
Salvia splendens Sellow ex Nees	Aerial parts	Anti-inflammatory effects of the aqueous extract (100 mg/kg/day, p.o.) was observed close to the effect of the standard indomethacin.	Methanol extract 70%, aqueous & ethyl acetate extracts.	El Sawi et al., 2021.
Salvia syriaca L.	Aerial parts	Anti-inflammatory activity was observed in the xylene-induced ear edema. The dexamethasone and ethanolic extract at doses of 50 and 100 mg/kg, i.p. significantly reduced the ear edema in a dose-dependent manner. The order of the topical anti-inflammatory effect for the Hex extracts was: *S. syriaca* > *S. palaestina*> *S. spinosa* > *S. multicaulis* > *S. dominica* > *S. ceratophylla. However, for EtOAc extracts the rank order was: S. multicaulis* > *S. palaestina* > *S. ceratophylla* > *S. spinosa* > *S. syriaca* > *S. dominica. Whereas MeOH extracts rank order was: S. spinosa* > *S. multicaulis* > *S. dominica* > *S. palaestina* > *S. syriaca* > *S. ceratophylla.*	Ethanol, hexane, ethyl acetate & methanol extracts.	Eidi et al., 2011; Shehadeh et al., 2014.
Salvia transsylvanica (Schur ex Griseb. & Schenk) Schur	Aerial parts	Anti-inflammatory effect in the edema induced with carrageenan (75% inhibition at 1000 mg/kg)	Ethanol extract 70%.	Maklad et al., 1999.
Salvia virgata Ortega	Aerial parts	Anti-inflammatory effect in rats in the edema induced with carrageenan at 100 mg/kg, p.o.	Methanol extract	Akkol et al., 2008; Küpeli et al., 2008;Grzegorczyk-Karolak et al., 2022.
Satureja cuneifolia Ten.	Aerial parts in flowering	Anti-inflammatory effect was observed in the first hour, but it was reduced after 4 h.	Organic extracts	Momtaz & Abdollahi 2010; Taskin et al. 2020.

Species	Plant part(s)	Pharmacology activity	Extract(s)	Reference
Satureja hortensis L.	Leaves & flowers, aerial parts, seeds.	Antinociceptive and anti-inflammatory effects were observed using the writhing test in the presence of EE= 2000 mg/kg, p.o. & AE= 200 mg/kg, p.o. As well as in the formalin test, EE= 500-2000 mg/kg, p.o., PF= 250-1000 mg/kg, p.o., & AE= 50-200 mg/kg, p.o. MP= 1000 mg/kg, p.o. & EO=200 mg/kg reduced edema induced by carrageenan. In the writhing test, EE produced 97% inhibition at 200 mg/kg, i.p., FP=87% inhibition at 200 mg/kg; & AE= 67% inhibition at 200 μL/kg, p.o. Whereas in the carrageenan-induced edema, EE=53% inhibition at 200 mg/kg, i.p.	Ethanol extract (EE), Polyphenolic fraction (PF). Essential oil (EO).	Hajhashemi et al., 2002; Hajhashemi et al., 2012.
Satureja khuzistanica Jamzad	Aerial parts	Decreased IL-1β at 50-200 mg/kg, i.p., in the carrageenan-induced edema with a potent inhibition at 150 mg/kg, i.p. in Wistar rats.	Essential oil & ethanol extract.	Amanlou et al., 2005; Abbasloo et al., 2016; Darabad et al., 2022.
Satureja montana L.	Aerial parts	A 60.37% inhibition of NO in a model of liver injury in Wistar rats.	Methanol extract 50%.	Miguel et al., 2021; Milijasevic et al., 2022.
Satureja sahendica Bornm.	Aerial parts & seeds.	Topical administration shortened the inflammatory phase, accelerated cellular proliferation, and increased fibroblast distribution per 1 mm^2, collagen deposition, and rapid epithelialization in comparison with control animals. Using an infected wound, it reduces the risk of infection and improves the healing process by promoting the transition from inflammation to proliferation and increasing expression of IGF-1, FGF-2, VEGF, TGF-ß, and CXCL-1.	Essential oil	Salehi et al., 2018; Omarizadeh et al., 2021.
Schizonepeta multifida Briq.	No identified	Greater than 50% inhibition of NO in mouse macrophages.	No identified	Fushiya et al., 2000.
Tetradenia riparia (Hochst.) Codd.	Leaves & stem	Anti-inflammatory activity & NO attenuation in an *in vitro* assay.	Essential oil	Ghuman et al., 2019; Garzoli et al., 2022; Shimira, 2022.
Thymbra capitata (L.) Cav.	Aerial parts	Inhibition of the TNF-α at 20 μg/mL. Inhibition of the 5-LO in an *in vitro* assay. Anti-inflammatory activity in the carrageenan-induced edema model in Sprague-Dawley rats using 100 mg/kg, p.o.	Essential oil	Albano & Miguel, 2011<br>Aazza et al., 2016<br>Taşkın et al., 2018<br>Aazza et al., 2016; Albano et al., 2011; Carrasco et al., 2016;

Species	Plant part(s)	Pharmacology activity	Extract(s)	Reference
				Llorent-Martínez et al., 2022; Miguel et al., 2021; Saoulajan et al., 2022; Tsioutsiou et al., 2022.
Thymus albicans Hoffmanns. & Link	Flowering parts	Inhibition of nitrites production by LPS-stimulated macrophages in a dose-dependent manner. A significant reduction by 27.16 and 41.32% was observed at the non-cytotoxic concentrations of 0.32 and 0.64 μL/mL, respectively.	Essential oil	Aazza et al., 2016; Roxo et al., 2020.
Thymus armeniacus Klokov & Des.-Shost.	Aerial parts	Anti-inflammatory effect in tail immersion method in mice and acetic acid-induced nociception. Inhibits the activity of ASIC1a and ASIC3 isoforms. Participation of the lamiuside A & verbascoside in *in vitro* inhibition of calcineurin activity (IC_{50}= 4.5 μM, 10.4 μM).	Essential oil	Modnicki et al., 2007; Prescott et al., 2011; Pargaien et al., 2020; Acimovic et al., 2022.
Thymus caespititius Brot.	Flowers, stems & leaves	*T. caespititius* appears to have anti-inflammatory potential based on its promising inhibitory activity on NO production. Non-phenolic compounds have a major rol in this action. -*T. caespititius* & *T. capitata* Eos showed the best capacity for preventing lipid peroxidation & scavenging the free radicals. *T. capitata and T. caespititius* EOs were also the best samples for inhibiting lipoxygenase.	Essential oil	Aazza et al., 2016; Alonso et al., 2017.
Thymus camphoratus Hoffmanns. & Link	Aerial parts in flowering	The essential oil was more effective since a higher iNOS inhibition was achieved (72% vs 43.56%). Inhibition of COX-2 expression (40.93% vs 7.1%), probably through inhibition of the NFκB.	Essential oil	Albano & Miguel, 2011; Zuzarte et al., 2018.
Thymus carnosus Boiss.	Aerial parts	An immunostimulatory effect was observed in fish *Clarias batrachus.* Inhibition of the carrageenan-induced edema (100 and 300 mg/kg), and in the mechanical and cold hyperalgesia. Inhibition of the carrageenan induced pleurisy reducing the migration of total leukocytes in mice by 82% at 30 mg/kg. Camphor from the essential oil inhibited all articular parameters induced by zymosan in mice (knee edema, leukocyte infiltration, mechanical hyperalgesia and NO) at 30 mg/kg. Inhibited the carrageenan induced pleurisy reducing the migration of total leukocytes in mice by 83% at 15 mg/kg. Mixture of 1,8 cineole/limonene 1:1: from the essential oil inhibited the carrageenan induced pleurisy reducing the migration of total leukocytes in mice by 80% at 6 mg/kg.	Essential oil & aqueous extract.	Nahak and Kanta, 2014; dos Santos et al., 2021; de Lima et al., 2014.

Species	Plant part(s)	Pharmacology activity	Extract(s)	Reference
Thymus citriodorus (Pers.) Schreb.	Aerial parts	NO inhibition in RAW 264.7 macrophages induced with LPS.	Essential oil	Oliveira et al., 2022.
Thymus daenensis Celak	Aerial parts	The herbal cream experimentally and histopathologically revealed a bum wound healing activity probably due to the antioxidant and anti-inflammatory activity of its phytochemical contents, especially phenolic compounds. *Thyme daenensis* extracts have antioxidant and anti-inflammatory properties and can improve liver injury in mice by decreasing pro-inflammatory TNF and IL-6 cytokines.	Essential oil & methanol extract.	Akbarinia et al., 2008; Babaeizadeh et al., 2016; Soosani & Sazegar, 2018.
Thymus herbabarona Loisel.	flowers, leaves and stems	*T. caespititius* has anti-inflammatory potential based on its promising inhibitory activity on NO production. *T. herba*-barona did not show anti-inflammatory potential since it was not able to inhibit the production of NO at concentrations without cytotoxicity to the cells. All the extracts revealed a high NO scavenging ability.	Crude extracts & phenolic fraction.	Zuzarte et al., 2013; Alfonso et al., 2017.
Thymus kotschyanus Boiss. & Hohen.	Leaves	Inhibition of the carrageenan-induced pleurisy in female Swiss mice at 30, 100, and 300 mg/kg.	Aqueous extract & essential oil.	Moraes et al., 2002; Piva et al., 2021.
Thymus linearis Benth.	Leaves & stems	Extracts were examined for their anti-inflammatory activities against carrageenan induced paw edema. Aqueous, methanol, & n-hexane extracts have produced analgesic, anti-inflammatory, and antipyretic activities. Both oils and extracts also exhibited significant pharmacological activities as an antipyretic, analgesic, and anti-inflammatory compared to the standards paracetamol, indomethacin, and ibuprofen tested on Swiss albino mice.	Hexane, aqueous extract & methanolic extracts. Essential oil.	Chandra et al., 2016; Qadir et al., 2016.
Thymus longicaulis C. Presl.	No identified	Extracts collected from different seasons exhibited a varying COX-2 gene expression inhibition in THP-1 macrophages.	Hydroalcoholic extract	Galasso et al., 2014.
Thymus mastichina L.	Aerial parts	Lipoxygenase inhibition with BE at IC_{50}=30.5 µg/mL & EO at IC_{50} 0.73 mg/mL. Reduction of the NO production in LPS-stimulated macrophages.	Butanol extract (BE) & essential oil (EO).	Albano & Miguel 2011; Aazza et al., 2016; Oliveira et al., 2023.
Thymus praecox Opiz	Aerial parts	ME demonstrated anti-inflammatory effect with 22.69% in the altered edema size after the first hour of carrageenan injection compared to fraction methanol extract. ME inhibitory	Petroleum ether extract,	Cam et al., 2019; Taskin et al., 2019.

Species	Plant part(s)	Pharmacology activity	Extract(s)	Reference
		effect increased for four hours and reached maximum by 76.44% Antioxidant active compounds play an important role in anti-inflammatory activity. Treatment decreased the levels of proinflammatory cytokines such as TNF-α, IL-1β and IL-6.	chloroform extract, & methanol extract (ME).	
Thymus quinquecostatus Celak	No identified	The essential oil (20, 40, & 80 mg/kg) showed a dose-dependent analgesic effect. Ear edema was suppressed in a concentration-dependent manner when 60, 120, & 240 μg/ear of thyme essential oil was administered through the ear edema test in mice.	Essential oil	Oh et al., 2008; Kim et al., 2022.
Thymus pubescens Boiss. & Kotschy ex Čelak.	Aerial parts	Anti-inflammatory effect in the edema induced with carrageenan (66.9% inhibition at 400, i.p.) in Wistar rats.	Methanol extract	Mahmoudi et al., 2008.
Thymus pulegioides L.	Aerial parts	Down-regulated NF-kB & simultaneously enhanced AP-1 activation in HepG2 cells.	Ethanol extract	Stalinska et al., 2005; Pavel et al., 2011.
Thymus serpyllum L.	Aerial parts	Decreases the inflammatory state in a murine model of obesity (50-150 mg/kg, p.o.) and reduces the IL-1β, IL-6, TNF-γ in mouse models with irritable bowel syndrome induced with deoxycholic acid.	Aqueous extract, & standardized extract at 70%, & 15% arabic gum & 15% maltodextrin.	Raal et al., 2004; Ruiz-Malagón et al., 2022; Ruiz-Malagón et al., 2022b.
Thymus sipyleus Boiss.	No identified	Anti-inflammatory activity by suppressing the overproduction of TNF, NO, & 5-LOX inhibitory effect.	Methanol, ethanol, & hexane extracts.	Günes et al., 2017; Demirci et al., 2018; Ustuner et al., 2019; Llorente-Martínez et al., 2022.
Thymus vulgaris L.	Aerial parts	Increase antioxidant enzymes such as glutathione S-transferase & superoxide dismutase in rabbit models of renal and hepatic dysfunction. A 80% decrease in NO levels in murine models with inflammation. Anti-inflammatory effect in humans with cholestasis, chronic hepatitis, & liver fibrosis.	Essential oil	Bacalbasa et al., 2022; Pandur et al., 2022.

Species	Plant part(s)	Pharmacology activity	Extract(s)	Reference
Zataria multiflora Boiss.	Aerial parts	Improvement of the IL-8 levels and lymphocytes in guinea pig induced COPD models at 0.4-1.6 mg/mL, p.o. Enhanced levels of proinflammatory cytokines in guinea pig model of asthma at 0.2-08 mg/mL. Anti-inflammatory effects at 500-900 mL/kg, i.p. on edema induced in Wistar rats.	Essential oil & ethanol extract.	Khazdair et al., 2018; Ghorani et al., 2022.
Ziziphora clinopodioides lam.	Aerial parts & leaves	Inhibition of proinflammatory cytokines in a mouse model of hemolytic anemia. Anti-inflammatory effect in a murine model of arteriosclerosis in Sprague-Dawley rats. Histamine induced edema. (50.41% inhibition at 500 mg/kg, i.p.). Decrease in the IL-4, IL-5, IL-10, & IFN-α in murine models of hemolytic anemia.	Aqueous extract-Fe-NPs, flavonoid fraction, methanol & aqueous extracts-Sn-NPs.	Chen et al., 2002; Shabbir et al., 2018; Ahmeda et al. 2020; Wu et al., 2020; Zhang et al., 2021; Ulikhanyan et al., 2022.
Ziziphora tenuior L.	Aerial parts	NO inhibition in RAW 264.7 macrophages induced with LPS. Decrease in apoptotic and inflammatory gene expression in the liver and lung. Improvement in functional and endocrine disturbances of estradiol valerate- induced polycystic ovary syndrome in rats and modulate the hormone level by anti-inflammatory effects.	Essential oil & hydroalcoholic extract.	Nabiuni et al., 2015; Abu-Darwish et al., 2016; Kianpour et al., 2021.

ALT: Alanina aminotransferasa
AP-1: factor de transcripción
AST: Aspartato aminotransferasa
BALF: Fluido broncoalveolar
cAMP: Adenosín monofosfato cíclico
CD-1 et/et: Mutante alopecica de linea CD-1
COX-2. Ciclooxigenasa 2
DPPH: 2,2-difenil-1-picrilhidracilo
FRAP: Capacidad de reducción férrica del plasma
GAE: Equivalente de ácido gálico
GSH: Glutatión
GSH-Px: Glutatión peroxidasa
HaCaT: Queratinocitos inmortalizados humanos
HASMCs: Células aórticas de musculo liso humanas
HepG2: Línea celular de cáncer de hígado humana
IC_{50}= Concentración inhibitoria máxima
ICAM-1: Molécula de adhesión intercelular-1
IFN-γ: Interferon gamma
IgE: Inmunoglobulina E
iNOS: Óxido nítrico sintasa
LPS: Lipopolisacárido
MCP-1: Proteína quimioatrayente de monocitos1
MDA: Malondialdehído
TNF-α: Factor de necrosis celular alfa
VCAM-1: Molécula de adhesión celular vascular-1
WBP: Proteína recombinante humana
α-SMA: Actina de músculo liso α
MMPS: Metaloproteinasas de la matriz extracelular:
MPO: Mieloperoxidasa
NF-κB p65: Factor nuclear κB p65
NF-κB: Factor nuclear kappa
NO: Óxido nítrico
PANC-1C: Células cancerígenas pancreáticas humanas
PBMCs: Celulas mononucleares de sangre periférica humanas
PCNA: Antígeno nuclear de células en proliferación
PGE2: Prostaglandina E2
PI3K / Akt / mTOR: Ruta de señalización intracelular
PLA2: Phospholipasa A2
PMA: Forbol-12-miristato-13-acetato
PPAR: Receptor activado por proliferador de peroxisomas
RBL-2H3: Células de leucemia basófilas
ROS: Especies reactivas de oxigeno
SC_{50}= Actividad antioxidante
SOD: Super óxido dismutasa
TGF-β1: factor de crecimiento tumoral-β1
Thp-1: Línea celular monocitica de leucemia humana

Aazza, S., El-Guendouz, S. & Miguel, M.G. (2016). Antioxidant, Anti-inflammatory and anti-hyperglycaemic activities of essential oils from *Thymbra capitata, Thymus albicans, Thymus caespititius, Thymus carnosus, Thymus lotocephalus* and *Thymus mastichina* from Portugal. *Natural Product Communications* 11(7). doi:10.1177/1934578X1601100739

Abbasloo, E., Dehghan, F., Khaksari, M., Najafipour, H., Vahidi, R., Dabiri, S., Sepehri, G., & Asadikaram, G. (2016). The anti-inflammatory properties of *Satureja khuzistanica* Jamzad essential oil attenuate the effects of traumatic brain injuries in rats. *Scientific Reports*, 6, 31866. doi.org/10.1038/srep31866

Abdolmaleki, A., Rajabi, A., Sanginabadi, F. (2013). Evaluation of the analgesic and anti-inflammatory effects of aqueous extract of peppermint (*Mentha piperita*). *Scientific Journal of Kurdistan University of Medical Sciences* 18(1), 67-74.

Abu-Darwish, M. S., Cabral, C., Ali, Z., Wang, M., Khan, S. I., Jacob, M. R., Efferth, T. (2020). *Salvia ceratophylla* L. from south of Jordan: New insights on chemical composition and biological activities. *Natural Products and Bioprospecting*, 10(5), 307-316. doi:10.1007/s13659-020-00259-9.

Acero, N., Ortega, T., Villagrasa, V., Leon, G., Muñoz-Mingarro, D., Castillo, E., González-Rosende, M.E., Borrás, S., Rios, J.L., Bosch-Morell, F., Martínez-Solís, I. (2023). Phytotherapeutic alternatives for neurodegenerative dementias: Scientific review, discussion and therapeutic proposal. *Phytotherapy Research*, 37, 1176–1211. doi:10.1002/ptr.7727.

Aćimović, M., Šeregelj, V., Simić, K., Varga, A., Pezo, L., Vulić, J., & Čabarkapa, I. (2022). Chemical profile of *Nepeta cataria* l. var. citriodora (becker) essential oil and in vitro evaluation of biological activities. *Acta Scientiarum Polonorum, Hortorum Cultus*, 21(4), 67–74. https://doi.org/10.24326/asphc.2022.4.7

Adriana Estrella, G. R., María Eva, G. T., Alberto, H. L., María Guadalupe, V. D., Azucena, C. V., Sandra, O. S., Noé, A. V., & Francisco Javier, L. M. (2021). Limonene from *Agastache mexicana* essential oil produces antinociceptive effects, gastrointestinal protection and improves experimental ulcerative colitis. *Journal of Ethnopharmacology*, 280. https://doi.org/10.1016/j.jep.2021.114462.

Afonso AF, Pereira OR, Fernandes ÂSF, Calhelha RC, Silva AMS, Ferreira ICFR, Cardoso SM. (2019b). The health-benefits and phytochemical profile of *Salvia apiana* and *Salvia farinacea* var. *victoria* Blue decoctions. Antioxidants (Basel) 8(8):241. https://doi: 10.3390/antiox8080241. PMID: 31349575; PMCID: PMC6721217.

Afonso AF, Pereira OR, Neto RT, Silva AMS, Cardoso SM. (2017). Health-Promoting Effects of *Thymus herba-barona, Thymus pseudolanuginosus*, and *Thymus caespititius* Decoctions. International Journal of Molecular Sciences 2017, 18, 1879. doi:10.3390/ijms18091879 .

Afonso, A. F., Pereira, O. R., Fernandes, Â., Calhelha, R. C., Silva, A. M. S., Ferreira, I. C. F., & Cardoso, S. M. (2019a). Phytochemical composition and bioactive effects of *Salvia africana*, *Salvia officinalis* 'icterina' and *Salvia mexicana* aqueous extracts. Molecules, 24(23) doi:10.3390/molecules24234327

Ahmad, F., Kamaraj, B., Mohamed, J.M.M., El-Sagheer, A.M., Emam, A.S.A. (2022). Ethnopharmacological and phytochemical study of major species from Labiatae family in Saudi Arabia: A Systematic Review. *International Journal of Applied Pharmaceutics*, 14 (ThematicSpecial Issue), pp. 44–57. DOI: https://dx.doi.org/10.22159/ijap.2022.v14ti.40

Ahmed, M.E.O., Abdelgadir, A.A., Ahmed, E.M. (2021). Traditional use of medicinal plants in Central Sudan Arabian. *Journal of Medicinal and Aromatic Plants*, 7(1), pp. 29–73. DOI: 10.48347/IMIST.PRSM/ajmap-v7i1.22273

Ahmeda, A., Mahdavi, B., Zaker, F., Kaviani, S., Hosseini, S., Zangeneh, M. M., Zangeneh, A., Paydarfar, S., & Moradi, R. (2020). Chemical characterization and anti-hemolytic anemia potentials of tin nanoparticles synthesized by a green approach for bioremediation applications. *Applied Organometallic Chemistry*, 34(3) e5433. doi.org/10.1002/aoc.5433

Aihaiti, K., Li, J., Xu, N.-N., Tang, D., Aisa, H.A. (2023). Monoterpenoid derivatives from *Hyssopus cuspidatus* Boriss. and their bioactivities. *Fitoterapia*, 165, 105432. https://doi.org/10.1016/j.fitote.2023.105432

Akbarinia, A., Sharifi-Ashabadi, A., Sefidkon, F. (2008). Influence of harvest dynamics on herb and oil yield of *Thymus kotschyanus* and *Thymus daenensis* cultivated at two sites. *Acta Horticulturae*, 786, pp. 229–234. Doi: 10.17660/ActaHortic.2008.786.25

Akkol, E. K., Göger, F., Koşar, M., & Başer, K. H. C. (2008). Phenolic composition and biological activities of *Salvia halophila* and *Salvia virgata* from Turkey. *Food Chemistry*, *108*(3), 942–949. doi.org/10.1016/j.foodchem.2007.11.071

Akram, M., Syed, A. S., Kim, K. A., Lee, J. S., Chang, S. Y., Kim, C. Y., & Bae, O. N. (2015). Heme oxygenase 1-mediated novel anti-inflammatory activities of *Salvia plebeia* and its active components. *Journal of Ethnopharmacology*, 174, 322–330. doi.org/10.1016/j.jep.2015.08.028

Aladedunye, F. A., Okorie, D. A., & Ighodaro, O. M. (2008). Anti-inflammatory and antioxidant activities and constituents of *Platostoma africanum* P. Beauv. *Natural Product Research*, 22(12), 1067–1073. doi.org/10.1080/14786410802264004

Albano, S. M., & Miguel, M. G. (2011). Biological activities of extracts of plants grown in Portugal. *Industrial Crops and Products*, 33(2), 338–343. doi:10.1016/j.indcrop.2010.11.012

Algieri, F., Rodriguez-Nogales, A., Vezza, T., ...Rodriguez-Cabezas, M.E., Galvez, J. (2016). Anti-inflammatory activity of hydroalcoholic extracts of *Lavandula dentata* L. and *Lavandula stoechas* L. *Journal of Ethnopharmacology*, 190, pp. 142–158. https://doi.org/10.1016/j.jep.2016.05.063

Ali, T., Javan, M., Sonboli, A., & Semnanian, S. (2012). Antinociceptive and anti-inflammatory activities of the essential oil of *Nepeta crispa* Willd. in experimental rat models. *Natural Product Research*, *26*(16), 1529–1534. doi.org/10.1080/14786419.2011.565284

Ali-Shtayeh, M.S., Abu-Zaitoun, S.Y., Dudai, N., Jamous, R.M. (2020). Downy lavender oil: a promising source of antimicrobial, antiobesity, and anti-Alzheimer's disease agents. *Evidence-Based Complementary and Alternative Medicine*, 2020, 5679408. doi: 10.1155/2020/5679408.

Aljohani, A. S. M., Alhumaydhi, F., Rauf, A., Hamad, E., & Rashid, U. (2022). *In Vivo* and *In Vitro* Biological Evaluation and Molecular Docking Studies of Compounds Isolated from *Micromeria biflora* (Buch. Ham. ex D.Don) Benth. *Molecules*, 27(11), 3377. doi.org/10.3390/molecules27113377

Almeida-Bezerra, J. W., Rodrigues, F. C., Lima Bezerra, J. J., Vieira Pinheiro, A. A., Almeida De Menezes, S., Tavares, A. B., Costa, A. R., Augusta De Sousa Fernandes, P., Bezerra Da Silva, V., Martins Da Costa, J. G., Pereira Da Cruz, R., Bezerra Morais-Braga, M. F., Melo Coutinho, H. D., Teixeira De Albergaria, E., Meiado, M. V., Siyadatpanah, A., Kim, B., & Morais De Oliveira, A. F. (2022). Traditional Uses, Phytochemistry, and Bioactivities of *Mesosphaerum suaveolens* (L.) Kuntze. In *Evidence-based Complementary and Alternative Medicine*, 1-23 doi.org/10.1155/2022/3829180

Al-Mijalli, S. H., Mrabti, N. N., Ouassou, H., Sheikh, R. A., Assaggaf, H., Bakrim, S., Abdallah, E. M., Alshahrani, M. M., Al Awadh, A. A., Lee, L. H., AlDhaheri, Y., Sahebkar, A., Zengin, G., Attar, A. A., Bouyahya, A., & Mrabti, H. N. (2022). Chemical Composition and Antioxidant, Antimicrobial, and Anti-Inflammatory Properties of *Origanum compactum* Benth Essential Oils from Two Regions: In Vitro and In Vivo Evidence and In Silico Molecular Investigations. *Molecules*, 27(21). https://doi.org/10.3390/molecules27217329

Alonazi M.A., Jemel I., Moubayed N., Alwhibi M., El-Sayed N.N.E., Ben Bacha A. (2021). Evaluation of the *in vitro* anti-inflammatory and cytotoxic potential of ethanolic and aqueous extracts of *Origanum syriacum* and *Salvia lanigera* leaves. *Environmental Science and Pollution Research* 28(16):19890-19900. https://doi: 10.1007/s11356-020-11961-z.

Al-Taweela, A.M., Raish, M., Perveen, S., Fawzy, G.H., Ahmad, A., Ansari, M.A., Mudassarg, S., Ganaie, M.A. (2017). *Nepeta deflersiana* attenuates isoproterenol-induced myocardial injuries in rats: Possible involvement of oxidative stress, apoptosis, inflammation through nuclear factor (NF)-κB downregulation. *Phytomedicine*, 34, 67–75. doi: http://dx.doi.org/10.1016/j.phymed.2017.08.003

Álvarez-Santos, N., Estrella-Parra, E. A., Benítez-Flores, J. del C., Serrano-Parrales, R., Villamar-Duque, T. E., Santiago-Santiago, M. A., González-Valle, M. del R., Avila-Acevedo, J. G., & García-Bores, A. M. (2022). *Asterohyptis stellulata*: Phytochemistry and wound healing activity. *Food Bioscience*, *50*, 102150. doi.org/10.1016/j.fbio.2022.102150

Alves-Silva, J.M., Pedreiro, S., Cavaleiro, C., Figueirinha, A., Salgueiro, L. (2023). Effect of *Thymbra capitata* (L.) Cav. on Inflammation, Senescence and Cell Migration. *Nutrients*, 15(8), 1930. https://doi.org/10.3390/nu15081930

Alves-Soares, R., de Oliveira, H.D., Campos, D.D.C.O., Leal-Cardoso, J.H., Coelho-de-Souza, A.N. (2022). The Essential Oil of *Hyptis crenata* Inhibits the Increase in Secretion of Inflammatory Mediators. *Plants*, 11(22), 3048. https://doi.org/10.3390/plants11223048

Al-Yousuf, M. H., Bashir, A. K., Ali, B. H., Tanira, M. O. M., & Blunden, G. (2002). Some effects of *Salvia aegyptiaca* L. on the central nervous system in mice. *Journal of Ethnopharmacology*, 81, 121–127. www.elsevier.com/locate/jethpharm

Alzweiri, M., Sarhan, A.A., Mansi, K., Hudaib, M., Aburjai, T. (2011). Ethnopharmacological survey of medicinal herbs in Jordan, the Northern Badia region. *Journal of Ethnopharmacology*, 137(1), pp. 27–35. https://doi.org/10.1016/S0378-8741(02)00057-0

Amanlou, M., Dadkhah, F., Farsam, H., Salehnia, A., & Dehpour, A. R. (2005). An anti-inflammatory and anti-nociceptive effect of hydroalcoholic extract of *Satureja khuzistanica* Jamzad extract. *Journal Pharmacology and Pharmaceutics Science*, *8*(1), 102–106. www.cspscanada.org

Aminian, A. R., Mohebbati, R., & Boskabady, M. H. (2022). The Effect of *Ocimum basilicum* L. and Its Main Ingredients on Respiratory Disorders: An Experimental, Preclinical, and Clinical Review. *Frontiers in Pharmacology* 12 805391. doi.org/10.3389/fphar.2021.805391

Amira, S., Dade, M., Schinella, G., & Rios, J.-L. (2012). Anti-inflammatory, anti-oxidant, and apoptotic activities of four plant species used in folk medicine in the Mediterranean basin T cell leukemia control via Ras-Raf pathway inhibition with peptides View project Mediterranean Plants for Skin Inflammation and Psoriasis View project. *Article in Pakistan Journal of Pharmaceutical Sciences*. www.researchgate.net/publication/51879323

Amirova, K.M., Dimitrova, P., Marchev, A.S., Aneva, I.Y., Georgiev, M.I. (2019). *Clinopodium vulgare* L. (wild basil) extract and its active constituents modulate cyclooxygenase-2 expression in neutrophils. *Food and Chemical Toxicology*, 124, pp. 1–9. https://doi.org/10.1016/j.fct.2018.11.054

An, H. J., Jeong, H. J., Um, J. Y., Kim, H. M., & Hong, S. H. (2006). *Glechoma hederacea* inhibits inflammatory mediator release in IFN-γ and LPS-stimulated mouse peritoneal macrophages. *Journal of Ethnopharmacology*, *106*(3), 418–424. doi.org/10.1016/j.jep.2006.01.024

Andrade, J. M., Custódio, L., Romagnoli, A., Reis, C. P., Rodrigues, M. J., Garcia, C., Petruccioli, E., Goletti, D., Faustino, C., Fimia, G. M., & Rijo, P. (2018). Antitubercular and anti-inflammatory properties screening of natural products from *Plectranthus* species. *Future medicinal chemistry*, 10(14), 1677–1691. https://doi.org/10.4155/fmc-2018-0043

Andreyeva, A.A., Sivtseva, V.V., Pashayeva, A.T., Chirikova, N.D., Okhlopkova, Z.M., Kim, S.W., Huseynova, I.M., Zulfugarov, I.S. (2020). Dragonhead as a model plant for biotechnology of biologically active compounds. *Azerbaijan Pharmaceutical and Pharmacotherapy Journal*, 1, 31-35.

Anjos, K. S. D., Araújo-Filho, H. G., Duarte, M. C., Costa, V. C. O., Tavares, J. F., Silva, M. S., Almeida, J. R. G. S., Souza, N. A. C., Rolim, L. A., Menezes, I. R. A., Coutinho, H. D. M., Quintans, J. S. S., & Quintans-Júnior, L. J. (2017). HPLC-dad analysis, antinociceptive and anti-inflammatory properties of the ethanolic extract of *Hyptis umbrosa* in mice. *EXCLI Journal*, *16*, 14–24. doi.org/10.17179/excli2016-698

Antão, A.R., Bangay, G., Domínguez-Martín, E.M., Díaz-Lanza, A.M., Ríjo, P. (2021). *Plectranthus ecklonii* Benth: A comprehensive review into its phytochemistry and exerted biological activities. *Frontiers in Pharmacology*, 12, 768268. doi: 10.3389/fphar.2021.768268.

Arenas-Chavez, C.A., Wiche-Salinas, T., Valencia-Mercado, I., Calle-Valdez, R., Vera-Gonzales, C., Malaga-Contreras, S., Huanqui-Guerra, C. (2018). Efecto antiiflamatorio de la fracción flavonoide de *Lepechinia meyenii* (Walp.) Epling (*Salvia*) sobre leucocitos de pacientes con artritis reumatoide. *Revista Peruana de Medicina Experimental y Salud Pública*, 35(1), 55-61 doi: 10.17843/rpmesp.2018.351.3600.

Asadollahi, M., Firuzi, O., Heidary Jamebozorgi, F., Alizadeh, M., & Jassbi, A. R. (2019). Ethnopharmacological studies, chemical composition, antibacterial and cytotoxic activities of essential oils of eleven *Salvia* in Iran. *Journal of Herbal Medicine*, 17-18 doi:10.1016/j.hermed.2018.11.006

Azevedo, M.-M., Pinheiro, C., Dias, A.C.P., Pinto-Ribeiro, F., Baltazar, F. (2015). Impact of an educational hands-on project on the antimicrobial, antitumor and anti-inflammatory properties of plants on portuguese students' awareness, knowledge, and competences. *International Journal of Environmental Research and Public Health*, 12(3), pp. 2437–2453. https://doi.org/10.3390/ijerph120302437

Bacalbasa, N., Balescu, I., Stoica, C., Pop, L., Varlas, V., Martac, C., Voichitoiu, A., & Gaspar, B. (2022). The anti-inflammatory, anti-infectious and anti-cancerous effects of *Thymus vulgaris*. *Romanian Journal of Medical Practice*, *17*, 21–23. doi.org/10.37897/RJMP. 2022.S3.6

Bakhtiarian, A., Aarabi Moghaddam, F., Zamani, M.M., Farahanikia, B., Khanavi, M. (2011). Anti-inflammatory effect of *Thymus kotschyanus* Boiss. & Hohen. extract on rat's hind paw edema induced by carrageenan. *Journal of Medicinal Plants*, 10(37), pp. 25–32.

Banerjee, P., Kumar, T., Sarangi, S. C., Meetei, U. D., Devi, A. S., & Kumar, R. (2021). Anti-inflammatory potential of aqueous extract of *Elsoltzia stachyodes* on experimental models of inflammation in rats. *Journal of Natural Science, Biology and Medicine*, *12*(1), 103–108. doi.org/10.4103/jnsbm.JNSBM_5_20

Banno, N., Akihisa, T., Tokuda, H., Yasukawa, K., Higashihara, H., Ukiya, M., Watanabe, K., Kimura, Y., Hasegawa, J.-I., & Nishino, H. (2004). Triterpene Acids from the Leaves of *Perilla frutescens* and Their Anti-inflammatory and Antitumor-promoting Effects. *Bioscience, Biotechnology and Biochemistry*, *68*(1), 85–90.

Batista, D., Falé, P. L., Serralheiro, M. L., Araújo, M. E., Dias, C., Branco, I., Grosso, C., Coelho, J., Palavra, A., Madeira, P. J. A., Martins, A., & Rauter, A. P. (2017). Phytochemical Characterization and Biological Evaluation of the Aqueous and Supercritical Fluid Extracts from *Salvia sclareoides* Brot. *Open Chemistry*, *15*(1), 82–91. doi.org/10.1515/chem-2017-0011

Bayat, M., Kalantar, K., & Amirghofran, Z. (2019). Inhibition of interferon-γ production and T-bet expression by menthol treatment of human peripheral blood mononuclear cells. *Immunopharmacology and Immunotoxicology*, *41*(2), 267–276. doi.org/10.1080/08923973.2019.1588294

Becker, K., Schwaiger, S., Waltenberger, B., Fuchs, D., Pezzei, C. K., Schennach, H., Stuppner, H., & Gostner, J. M. (2018). Immunomodulatory Effects of Diterpene Quinone Derivatives from the Roots of *Horminum pyrenaicum* in Human PBMC. *Oxidative Medicine and Cellular Longevity*, 2018. doi.org/10.1155/2018/2980295

Belozerova, O.A.; Osmakov, D.I.; Vladimirov, A.; Koshelev, S.G.; Chugunov, A.O.; Andreev, Y.A.; Palikov, V.A.; Palikova, Y.A.; Shaykhutdinova, E.R.; Gvozd, A.N.; et al. (2020). Sevanol and Its Analogues: Chemical Synthesis, Biological Effects and Molecular Docking. *Pharmaceuticals*, 13, 163. https://doi.org/10.3390/ph13080163

Benrezzouk, R., Terencio, M. C., Ferrandiz, M. L., Hernandez-Perez, M., Rabanal, R., & Alcaraz, M. J. (2001). Inhibition of 5-lipoxygenase activity by the natural anti-inflammatory compound aethiopinone. *Inflammation Research*, 50(2), 96-101. doi:10.1007/s000110050730.

Beserra-Filho, J.I.A., de Macêdo, A.M., Leão, A.H.F.F., Bispo, J.M.M., Santos, J.R., de Oliveira-Melo, A.J., Passos Menezes, P.D., Duarte, M.C., de Souza Araújo, A.A., Silva, R.H., Quintans-Júnior, L.J., Ribeiro, A.M. (2019). *Eplingiella fruticosa* leaf essential oil complexed with β-cyclodextrin produces a superior neuroprotective and behavioral profile in a mice model of Parkinson's disease. *Food and Chemical Toxicology*, 124, 17–29. https://doi.org/10.1016/j.fct.2018.11.056.

Bhardwaj, S., & Bhatia, S. (2020). Development and characterization of niosomal gel system using *Lallementia royaleana* Benth. mucilage for the treatment of rheumatoid arthritis. *Iranian Journal of Pharmaceutical Research*: IJPR, 19(3), 465–482. doi.org/10.22037/ijpr.2020.112887.14003

Bian, J., Wang, K., Wang, Q,., Wang, P., Wang, T., Shi, W., Ruan, Q. (2020). *Dracocephalum heterophyllum* (DH) exhibits potent anti-proliferative effects on autoreactive CD4+ T cells and ameliorates the development of experimental autoimmune uveitis. *Frontiers in Immunology*, 11:575669. doi: 10.3389/fimmu.2020.575669

Bian, M., Zhang, Y., Du, X., Xu, J., Cui, J., Gu, J., Zhu, W., Zhang, T., & Chen, Y. (2017). Apigenin-7-diglucuronide protects retinas against bright light-induced photoreceptor degeneration through the inhibition of retinal oxidative stress and inflammation. *Brain Research*, *1663*, 141–150. doi.org/10.1016/j.brainres.2017.03.019

Bonesi, M., Loizzo, M. R., Acquaviva, R., Malfa, G. A., Aiello, F., & Tundis, R. (2017). Anti-inflammatory and antioxidant agents from Salvia genus (Lamiaceae): An assessment of the current state of knowledge. *Anti-Inflammatory and Anti-Allergy Agents in Medicinal Chemistry*, 16(2), 70-86. doi:10.2174/1871523016666170502121419

Borges, R. S., Ortiz, B. L. S., Pereira, A. C. M., Keita, H., & Carvalho, J. C. T. (2019). *Rosmarinus officinalis* essential oil: A review of its phytochemistry, anti-inflammatory activity, and mechanisms of action involved. *Journal of Ethnopharmacology* (Vol. 229, pp. 29–45). Elsevier Ireland Ltd. https://doi.org/10.1016/j.jep.2018.09.038

Boukhary, R., Raafat, K., Ghoneim, A. I., Aboul-Ela, M., & El-Lakany, A. (2016). Anti-Inflammatory and Antioxidant Activities of *Salvia fruticosa*: An HPLC Determination of Phenolic Contents. *Evidence-Based Complementary and Alternative Medicine, 2016*. doi.org/10.1155/2016/7178105

Bounihi, A., Hajjaj, G., Alnamer, R., Cherrah, Y., & Zellou, A. (2013). In vivo potential anti-inflammatory activity of *Melissa officinalis* l. essential oil. *Advances in Pharmacological Sciences, 2013*. doi.org/10.1155/2013/101759

Bouyahya, A., Chamkhi, I., Menyiy, N.E., Simal-Gandara, J., El Omari, N. (2023). Traditional use, phytochemistry, toxicology, and pharmacological properties of *Lavandula dentata* L.,: A comprehensive review. *South African Journal of Botany*, 154, pp. 67–87.

Brahmi, F., Nury, T., Debbabi, M., Hadj-Ahmed, S., Zarrouk, A., Prost, M., Madani, K., Boulekbache-Makhlouf, L., & Lizard, G. (2018). Evaluation of antioxidant, anti-inflammatory and cytoprotective properties of ethanolic mint extracts from Algeria on 7-ketocholesterol-treated murine RAW 264.7 macrophages. *Antioxidants*, *7*(12), 1-15. doi.org/10.3390/antiox7120184

Burk, D.R., Senechal-Willis, P., Lopez, L.C., Hogue, B.G., Daskalova, S.M. (2009). Suppression of lipopolysaccharide-induced inflammatory responses in RAW 264.7 murine macrophages by aqueous extract of *Clinopodium vulgare* L. (Lamiaceae). *Journal of Ethnopharmacology*, 2009, 126(3), pp. 397–405.

Cai, X., Mao, Y., Shen, X., ...He, J., Zhang, M. (2023). The extract from *Hyssopus cuspidatus* Boriss. Prevents bronchial airway remodeling by inhibiting mouse bronchial wall thickening and hASMC proliferation and migration. *Journal of Ethnopharmacology*, 2023, 303, 116047.

Calvo, M.I., Cavero, R.Y. (2015). Medicinal plants used for neurological and mental disorders in Navarra and their validation from official sources. *Journal of Ethnopharmacology*, 169, pp. 263–268.

Cam ME, Hazar-Yavuz AN, Yildiz S, Ertas B, Ayaz Adakul B, Taskin T, Alan S, Kabasakal L. (2019). The methanolic extract of *Thymus praecox* subsp. skorpilii var. skorpilii restores glucose homeostasis, ameliorates insulin resistance and improves pancreatic β-cell function on streptozotocin/nicotinamide-induced type 2 diabetic rats. *Journal of Ethnopharmacology* 231 (2019) 29–38. https://doi.org/10.1016/j.jep.2018.10.028.

Carbone C, Martins-Gomes C, Caddeo C, Silva AM, Musumeci T, Pignatello R, Puglisi G, Souto EB. (2018). Mediterranean essential oils as precious matrix components and active T ingredients of lipid nanoparticles. *International Journal of Pharmaceutics* 548 (2018) 217–226. doi.org/10.1016/j.ijpharm.2018.06.064

Cardia, G. F. E., Silva-Filho, S. E., Silva, E. L., Uchida, N. S., Cavalcante, H. A. O., Cassarotti, L. L., Salvadego, V. E. C., Spironello, R. A., Bersani-Amado, C. A., & Cuman, R. K. N. (2018). Effect of Lavender (*Lavandula angustifolia*) Essential Oil on Acute Inflammatory Response. *Evidence-Based Complementary and Alternative Medicine*, *2018*. doi.org/10.1155/2018/1413940.

Cariddi, L. N., González-Pereyra, M. L., Gambero, M. L., Demo, M. S., Isola, M. C., Franzoni, L., Sabini, L. I., & Maldonado, A. M. (2006). Minthostachys verticillata (Griseb.) Epling: A South American plant with antiinflammatory effect in vitro. *Allergy and Clinical Immunology International*, 18(6), 234–241. https://doi.org/10.1027/0838-1925.18.6.234.

Carrasco, A., Martinez-Gutierrez, R., Tomas, V., Tudela, J. (2015). *Lavandula angustifolia* and *Lavandula latifolia* Essential Oils from Spain: Aromatic Profile and Bioactivities. *Planta Medica*, 82(1-2), pp. 163–170.

Carrasco, A., Perez, E., Cutillas, A. -., Martinez-Gutierrez, R., Tomas, V., & Tudela, J. (2016). *Origanum vulgare* and T*hymbra capitata* essential oils from spain: Determination of aromatic profile and bioactivities. *Natural Product Communications*, 11(1), 113-120. doi:10.1177/1934578x1601100133

Castro, J.P., Franco, L.A., Diaz, F. (2021). Anti-inflammatory screening of plant species from the Colombian Caribbean Coast. *Journal of Applied Pharmaceutical Science*, 11(4), 106-117. doi: 10.7324/JAPS.2021.110413.

Ceschel, G. C., Maffei, P., Moretti, M. D. L., Demontis, S., & Peana, A. T. (2000). *In vitro* permeation through porcine buccal mucosa of *Salvia desoleana* atzei and picci essential oil from topical formulations. *International Journal of Pharmaceutics*, 195(1-2), 171-177. doi:10.1016/S0378-5173(99)00381-6

Chan, H. H., Hwang, T. L., Su, C. R., Reddy, M. V. B., & Wu, T. S. (2011). Anti-inflammatory, anticholinesterase and antioxidative constituents from the roots and the leaves of *Salvia nipponica* Miq. var. *formosana*. *Phytomedicine*, *18*(2–3), 148–150. doi.org/10.1016/j.phymed.2010.06.017

Chang, C. T., Soo, W. N., Chen, Y. H., & Shyur, L. F. (2019). Essential Oil of *Mentha aquatica* var. *Kenting* water mint suppresses two-stage skin carcinogenesis accelerated by BRAF inhibitor vemurafenib. *Molecules*, 24(12). https://doi.org/10.3390/molecules24122344

Chen, J., Wang, J. B., Yu, C. H., Chen, L. Q., Xu, P., & Yu, W. Y. (2013). Total flavonoids of *Mosla scabra* leaves attenuates lipopolysaccharide-induced acute lung injury via down-regulation of inflammatory signaling in mice. *Journal of ethnopharmacology*, 148(3), 835–841. doi.org/10.1016/j.jep.2013.05.020

Chen, S., Fang, A., Zhong, Y., & Tang, J. (2022). *Ziziphora clinopodioides* Lam leaf aqueous extract mediated novel green synthesis of iron nanoparticles and its anti-hemolytic anemia potential: A chemobiological study. *Arabian Journal of Chemistry*, 15(3), 103561. doi.org/10.1016/j.arabjc.2021.103561

Chen, X., Zhang, L., Qian, C., Du, Z., Xu, P., & Xiang, Z. (2020). Chemical compositions of essential oil extracted from *Lavandula angustifolia* and its prevention of TPA-induced inflammation. *Microchemical Journal*, *153*. doi.org/10.1016/j.microc.2019.104458

Cheng, E., Chi, J., Li, Y. X., Zhang, W. J., Huang, N., Wang, Z. M., Dai, L. P., & Xu, E. P. (2022). Diverse ent-kaurane diterpenoids from *Isodon henryi*. *Tetrahedron Letters*, *108*. doi.org/10.1016/j.tetlet.2022.154119

Chirikova, N.K., Nokhsorov V.V., Nikolaev, W.M. (2021). Ethnopharmacological research of plant resources of central.

Choia, H., Kim, T., Kim, S.-H., & Kim, J. (2016). Anti-allergic inflammatory triterpenoids isolated from the spikes of *Prunella vulgaris*. *Natural Product Communications*, *11*(1), 2–31.

Chou, S., Lai, C. Lai, C., Chao, W. (2018). Chemical composition, antioxidant, anti-melanogenic and anti-inflammatory activities o *Glechoma hederacea* (Lamiaceae) essential oil. *Industrial Crops and Products* 122, 675-685. doi.org/10.1016/j.indcrop.2018.06.032.

Choudhary, M.I., Siddiqui, Z.A., Nawaz, S.A., Atta-ur-Rahman. (2006). Microbial transformation and butyrylcholinesterase inhibitory activity of (-)-caryophyllene oxide and its derivatives. *Journal of Natural Products*, 69(10), pp. 1429–1434.

Cigremis, Y., Ulukanli, Z., Ilcim, A., Akgoz, M. (2010). In vitro antioxidant and antimicrobial assays of acetone extracts from *Nepeta meyeri* Bentham. *European Review for Medical and Pharmacological Sciences*, 14, 661–668.

Coelho MN, Soares PAG, Frattani FS, Camargo LMM, Tovar AMF, de Aguiar PF, Zingali RB, Mourão PAS, Costa SS. Polysaccharide composition of an anticoagulant fraction from the aqueous extract of *Marsypianthes chamaedrys* (Lamiaceae). *International Journal of Biological Macromolecules* 145 (2020) 668–681. doi.org/10.1016/j.ijbiomac.2019.12.176 0141-8130/.

Coelho-De-souza, A.N., Alves-Soares, R., Oliveira, H.D., Guimarães-Pereira, J., Leal-Cardoso, J.H. (2021). The essential oil of *Hyptis crenata* Pohl ex Benth. Presents an antiedematogenic effect in mice. *Brazilian Journal of Medical and Biological Research*, 54(3), pp. 1–9, e9422

Contreras, M.D.M., Algieri, F., Rodriguez-Nogales, A., Gálvez, J., Segura-Carretero, A. (2018). Phytochemical profiling of anti-inflammatory *Lavandula* extracts via RP–HPLC–DAD–QTOF–MS and –MS/MS: Assessment of their qualitative and quantitative differences. *Electrophoresis*, 2018, 39(9-10), pp. 1284–1293.

Côté, H., Pichette, A., St-Gelais, A., Legault, J. (2021). The biological activity of *Monarda didyma* L. essential oil and its effect as a diet supplement in mice and broiler chicken. *Molecules*, 26, 3368. doi: https://doi.org/10.3390/molecules26113368.

Dadkhah, A., Fatemi, F., Rasooli, A., Malayeri, M. R. M., & Torabi, F. (2018). Assessing the effect of *Mentha longifolia* essential oils on cox-2 expression in animal model of sepsis induced by caecal ligation and puncture. *Pharmaceutical Biology*, *56*(1), 495–504. doi.org/10.1080/13880209.2018.1510972

Dal Piaz F., Imparato S., Lepore L., Bader A., De Tommasi N. 2010. A fast and efficient LC-MS/MS method for detection, identification and quantitative analysis of bioactive sesterterpenes in *Salvia dominica* crude extracts. *Journal Pharmaceutical Biomedical Analysis* 51(1):70-77. https://10.1016/j.jpba.2009.08.006.

Darabad, A., Rahimi, M., Rafati, H. (2022). Continuous hydrodistillation-ultrasonication flow batch-mode operation system to enhance essential oil extraction from *Satureja khuzistanica* and evaluation of in vitro anti-inflammatory activity. *Industrial Crops and Products* 188(A), 115540. doi.org/10.1016/j.indcrop.2022.115540

De Felice A., Bader A., Leone A., Sosa S., Loggia R.D., Tubaro A., De Tommasi N. 2006. New polyhydroxylated triterpenes and anti-inflammatory activity of *Salvia hierosolymitana*. *Planta Medica* 72(7):643-649. https://doi: 10.1055/s-2006-931573

De Jesus, N.Z.T., Lima, J.C.D.S., Da Silva, R.M., Espinosa, M.M., Martins, D.T.D.O. (2009). Ethnobotanical survey of plants popularly used as anti-ulcer and anti-inflammatory in Pirizal, Nossa Senhora do Livramento, MT, Brazil | Levantamento etnobotânico de plantas popularmente utilizadas como antiúlceras e antiinflamatórias pela comunidade de Pirizal, Nossa Senhora do Livramento-MT, Brasil. *Revista Brasileira de Farmacognosia*, 19(1 A), pp. 130–139.

De Lima, M.N.N., Guimarães, B.A., de Castro, A.L.S., ...Maia, J.G.S., Figueiredo, P.L.B. (2023). Chemical composition and antinociceptive and anti-inflammatory activity of the essential oil of *Hyptis crenata* Pohl ex Benth. from the Brazilian Amazon. *Journal of Ethnopharmacology*, 300, 115720.

De Lima, V. T., Vieira, M. C., Kassuya, C. A. L., Cardoso, C. A. L., Alves, J. M., Foglio, M. A., De Carvalho, J. E., & Formagio, A. S. N. (2014). Chemical composition and free radical-scavenging, anticancer and anti-inflammatory activities of the essential oil from *Ocimum kilimandscharicum*. *Phytomedicine*, 21(11), 1298–1302. https://doi.org/10.1016/j.phymed.2014.07.004.

De Macedo, L. M., Dos Santos, É. M., Militão, L., Tundisi, L. L., Ataide, J. A., Souto, E. B., & Mazzola, P. G. (2020). Rosemary (Rosmarinus officinalis L., syn salvia rosmarinus spenn.) and its topical applications: A review. *Plants* (Vol. 9, Issue 5). MDPI AG. https://doi.org/10.3390/plants9050651.

De Oliveira Ferreira, E., Soares Dias, M.Y., Rocha de Lima, N.M., Tavares Neves, K.R., Santos do Carmo, M.R., Viana Lima, F.A., Aragão Fonteles, A., Fontenele Menezes, A.P., Matos de Andrade, G. (2016). Neuroinflammatory response to experimental stroke is inhibited by eriodictyol. *Behavioural Brain Research*, 312, 321-332. http://dx.doi.org/10.1016/j.bbr.2016.06.046.

De Pinho, J.P.M., Silva, A.S.B., Pinheiro, B.G., ...Da Cunha Sousa, P.J., Magalhães, P.J.C. (2012). Antinociceptive and antispasmodic effects of the essential oil of *Ocimum micranthum*: Potential anti-inflammatory properties. *Planta Medica*, 78(7), pp. 681–685.

De Sousa Araújo, T.A., Alencar, N.L., de Amorim, E.L.C., de Albuquerque, U.P. (2008). A new approach to study medicinal plants with tannins and flavonoids contents from the local knowledge. *Journal of Ethnopharmacology*, 120(1), pp. 72–80.

Demirci, F., Karaca, N., Tekin, M., Demirci, B. (2018). Anti-inflammatory and antibacterial evaluation of *Thymus sipyleus* Boiss. subsp. *sipyleus* var. *sipyleus* essential oil against rhinosinusitis pathogens. *Microbial Pathogenesis*, 122, pp. 117–121.

Demirci, F., Karadağ, A. E., Biltekin, S. N., & Demirci, B. (2021). In Vitro ACE2 and 5-LOX Enzyme Inhibition by Menthol and Three Different Mint Essential Oils. *Natural Product Communications*, *16*(11). doi.org/10.1177/1934578X211055014

Dobrev, H.P. (2021). Treatment of numerous hand warts with *Clinopodium vulgare* tea. *Wiener Medizinische Wochenschrift*, 171(3-4), pp. 82–83.

Donatello, N. N., Emer, A. A., Salm, D. C., Ludtke, D. D., Bordignon, S. A. S. R., Ferreira, J. K., Salgado, A. S. I., Venzke, D., Bretanha, L. C., Micke, G. A., & Martins, D. F. (2020). *Lavandula angustifolia* essential oil inhalation reduces mechanical hyperalgesia in a model of inflammatory and neuropathic pain: The involvement of opioid and cannabinoid receptors. *Journal of Neuroimmunology*, *340*. doi.org/10.1016/j.jneuroim.2020.577145

Dos Santos, E., Leitão, M. M., Aguero Ito, C. N., Silva-Filho, S. E., Arena, A. C., Silva-Comar, F. M. de S., Nakamura Cuman, R. K., Oliveira, R. J., Nazari Formagio, A. S., & Leite Kassuya, C. A. (2021). Analgesic and anti-inflammatory articular effects of essential oil and camphor isolated from *Ocimum kilimandscharicum* Gürke leaves. *Journal of Ethnopharmacology*, 269. https://doi.org/10.1016/j.jep.2020.113697

Draginic, N., Andjic, M., Jeremic, J., Zivkovic, V., Kocovic, A., Tomovic, M., Bozin, B., Kladar, N., Bolevich, S., Jakovljevic, V., & Milosavljevic, I. (2022). Anti-inflammatory and Antioxidant Effects of *Melissa officinalis* Extracts: A Comparative Study. *Iranian Journal of Pharmaceutical Research*, *21*(1). doi.org/10.5812/ijpr-126561

Du, Y., Jin, Y., Liu, P., Shi, X., Xu, H., Wang, Q., & Zhang, L. (2013). Quantitative analysis of ten diterpenoids in rat bile after oral administration of *Isodon rubescens* extract by high performance liquid chromatography-electrospray ionization tandem mass spectrometry. *Journal of Liquid Chromatography and Related Technologies*, *36*(9), 1264–1279. doi.org/10.1080/10826076.2012.686137

Dubinnyi, M.A., Osmakov, D.I., Koshelev, S.G., ...Arseniev, A.S., Grishin, E.V. 2012. Lignan from thyme possesses inhibitory effect on ASIC3 channel current. *Journal of Biological Chemistry*, 287(39), pp. 32993–33000.

Eidi A., Eidi TMM, Mozaffarian V., Rustaiyan A., Mazooji A.S., Khaboori Z., Nabiuni F. 2011. Antinociceptive and Anti-inflaminatory Effects of Ethanolic Extract of *Salvia syriaca* L. in Mice. *International Journal of Pharmacology* 7(3): 394-399, 2011 DOI: 10.3923/p.2011.394.399

El Kharraf, S., El-Guendouz, S., Farah, A., Bennani, B., Mateus, M. C., El Hadrami, E. M., & Miguel, M. G. (2021). Hydrodistillation and simultaneous hydrodistillation-steam distillation of *Rosmarinus officinalis* and *Origanum compactum*: Antioxidant, anti-inflammatory, and antibacterial effect of the essential oils. *Industrial Crops and Products*, 168. https://doi.org/10.1016/j.indcrop.2021.113591.

El Menyiy, N., Mrabti, H. N., el Omari, N., Bakili, A. E. I., Bakrim, S., Mekkaoui, M., Balahbib, A., Amiri-Ardekani, E., Ullah, R., Alqahtani, A. S., Shahat, A. A., & Bouyahya, A. (2022). Medicinal Uses, Phytochemistry, Pharmacology, and Toxicology of *Mentha spicata*. In *Evidence-based Complementary and Alternative Medicine* (Vol. 2022). Hindawi Limited. doi.org/10.1155/2022/7990508

El Sawi, S.A., Sleem, A.A., Merghany, R.M. 2021. *In vivo* antioxidant, anti-inflammatory and anti-hyperglycemic effects of *Salvia splendens* aerial parts in male albino rat models. *Journal of Herbs, Spices & Medicinal Plants* 27(2): 149-160. https://doi.org/10.1080/10496475.2021.1891173.

El Yaagoubi, M., Mechqoq, H., El Hamdaoui, A., ...Msanda, F., El Aouad, N. (2021). A review on Moroccan *Thymus* species: Traditional uses, essential oils chemical composition and biological effects. *Journal of Ethnopharmacology*, 278, 114205.

El-Askary, H., El-Kashoury, E., Kandil, Z., Salem, M., & Sleem, A. (2012). Chemical composition and biological activities of the essential oil of *Mentha suaveolens* Ehrh. Z. Naturforsch, 67(3), 571–579.

Esquivel, B., Bustos-Brito, C., Sánchez-Castellanos, M., Nieto-Camacho, A., Ramírez-Apan, T., Joseph-Nathan, P., Quijano, L. (2017). Structure, absolute configuration, and antiproliferative activity of Abietane and Icetexane diterpenoids from *Salvia ballotiflora*. *Molecules*, 22(10), 1690. doi: 10.3390/molecules22101690.

Estakhr, J., & Javdan, N. (2011). Evaluation of anti-inflammatory effects of ethanolic extract of *Salvia hypoleuca*. *Pharmacologyonline*, 2, 879-885.

Estakhr, J., & Javdan, N. (2011a). *Salvia hypoleuca*; a new source of compounds which are useful for treatment of ocular inflammation. *Pharmacologyonline*, 3, 338-346. Retrieved from www.scopus.com

Fawole, O. A., Amoo, S. O., Ndhlala, A. R., Light, M. E., Finnie, J. F., & van Staden, J. (2010). Anti-inflammatory, anticholinesterase, antioxidant, and phytochemical properties of medicinal plants used for pain-related ailments in South Africa. *Journal of Ethnopharmacology*, *127*(2), 235–241. doi.org/10.1016/j.jep.2009.11.015

Feng, J., Liu, L., Yao, F., Zhou, D., He, Y., & Wang, J. (2021). The protective effect of tanshinone IIA on endothelial cells: a generalist among clinical therapeutics. In *Expert Review of Clinical Pharmacology* 14(2), 239–248. doi.org/10.1080/17512433.2021.1878877

Feng, X., Wang, X., Liu, Y., & Di, X. (2015). Linarin Inhibits the Acetylcholinesterase Activity In-vitro and Ex-vivo. In *Shaheed Beheshti University of Medical Sciences and Health Services Iranian Journal of Pharmaceutical Research* 14(3), 949-954.

Fiuza, T., Rezende M., Sabóia-Morais, S., Tresvenzol, L., Ferreira H., Paula, J. 2008. Estudo das folhas e caule de *Hyptidendron canum* (Pohl ex Benth.) Harley, Lamiaceae. *Revista Brasileira de Farmacognosia Brazilian Journal of Pharmacognosy* 20(2): 192-200.

Fraternale, D., Dufat, H., Albertini, M.C., Bouzidi, C., D'Adderio, R., Coppari, S., Di Giacomo, B., Melandri, D., Ramakrishna, S., Colomba, M. (2022). Chemical composition, antioxidant and anti-inflammatory properties of *Monarda didyma* L. essential oil. *PeerJ*, 10, e14433. doi: 10.7717/peerj.14433.

Fushiya, S., Batkhuu, J., Takano, F., Hayasaka, H., Ohba, K., Yoshizaki, F., Dashi, T., Batsuren, D., & Sanchir, C. (2000). Effects of Mongolian plants on nitric oxide production in activated macrophages. *Natural Medicines*, *54*(6), 352–357.

García-Díaz, J. A., Navarrete-Vázquez, G., García-Jiménez, S., Hidalgo-Figueroa, S., Almanza-Pérez, J. C., Alarcón-Aguilar, F. J., Gómez-Zamudio, J., Cruz, M., Ibarra-Barajas, M., & Estrada-Soto, S. (2016). Antidiabetic, antihyperlipidemic and anti-inflammatory effects of tilianin in streptozotocin-nicotinamide diabetic rats. *Biomedicine & Pharmacotherapy*, 83, 667–675. doi.org/10.1016/j.biopha.2016.07.023

Garzoli, S., Alarcón-Zapata, P., Seitimova, G., ...Pezzani, R., Vitalini, S. (2022). Natural essential oils as a new therapeutic tool in colorectal cancer. *Cancer Cell International*, 22(1), 407.

Georgiev, Y. N., Paulsen, B. S., Kiyohara, H., Ciz, M., Ognyanov, M. H., Vasicek, O., Rise, F., Denev, P. N., Yamada, H., Lojek, A., Kussovski, V., Barsett, H., Krastanov, A. I., Yanakieva, I. Z., & Kratchanova, M. G. (2017). The common lavender (*Lavandula angustifolia* Mill.) pectic polysaccharides modulate phagocytic leukocytes and intestinal Peyer's patch cells. *Carbohydrate Polymers*, *174*, 948–959. doi.org/10.1016/j.carbpol.2017.07.011

Ghorani, V., Beigoli, S., Khazdair, M. R., & Boskabady, M. H. (2022). The effect of *Zataria multiflora* on respiratory allergic and immunologic disorders, experimental and clinical evidence: A comprehensive review. In *Phytotherapy Research* 36(3), 1135–1155. doi.org/10.1002/ptr.7382

Ghuman, S., Ncube, B., Finnie, J.F., Mcgaw, L.J., Njoya, E.M., Coopoosamy, R.M., Van-Staden, J., 2019. Antioxidant, anti-inflammatory and wound healing properties of medicinal plant extracts used to treat wounds and dermatological disorders. *South African Journal of Botany*. 126, 232–240. doi.org/10.1016/j.sajb.2019.07.013.

Giovannini, D., Gismondi, A., Basso, A., Canuti, L., Braglia, R., Canini, A., Mariani, F., & Cappelli, G. (2016). *Lavandula angustifolia* mill. Essential oil exerts antibacterial and anti-inflammatory effect in macrophage mediated immune response to staphylococcus aureus. *Immunological Investigations*, *45*(1), 11–28. doi.org/10.3109/08820139.2015.1085392

González-Ramírez, A., González-Trujano, M. E., Pellicer, F., & López-Muñoz Francisco, J. (2012). Anti-nociceptive and anti-inflammatory activities of the *Agastache mexicana* extracts by using several experimental models in rodents. *Journal of Ethnopharmacology*, 142(3), 700–705. doi.org/10.1016/j.jep.2012.05.04

Guginski, G., Luiz, A. P., Silva, M. D., Massaro, M., Martins, D. F., Chaves, J., Mattos, R. W., Silveira, D., Ferreira, V. M. M., Calixto, J. B., & Santos, A. R. S. (2009). Mechanisms involved in the antinociception caused by ethanolic extract obtained from the leaves of *Melissa officinalis* (lemon balm) in mice. *Pharmacology Biochemistry and Behavior*, *93*(1), 10–16. doi.org/10.1016/j.pbb.2009.03.014

Güneş, S., Savran, A., Paksoy, M. Y., Koşar, M., & Çakılcıoğlu, U. (2017). Ethnopharmacological survey of medicinal plants in Karaisalı and its surrounding (Adana-turkey). *Journal of Herbal Medicine*, 8, 68-75. doi:10.1016/j.hermed.2017.04.002

Hajhashemi, V., Ghannadi, A., & Karim Pezeshkian, S. (2002). Antinociceptive and anti-inflammatory effects of *Satureja hortensis* L. extracts and essential oil. *Journal of Ethnopharmacology*, *82*, 83–87. www.elsevier.com/locate/jethpharm

Hajhashemi, V., Ghannadi, A., & Sharif, B. (2003). Anti-inflammatory and analgesic properties of the leaf extracts and essential oil of *Lavandula angustifolia* Mill. *Journal of Ethnopharmacology*, *89*(1), 67–71. doi.org/10.1016/S0378-8741(03)00234-4

Hajhashemi, V., Zolfaghari, B., & Yousefi, A. (2012). Antinociceptive and anti-inflammatory activities of *Satureja hortensis* seed essential oil, hydroalcoholic and polyphenolic extracts in animal models. *Medical Principles and Practice*, *21*(2), 178–182. doi.org/10.1159/000333555

Hamamouchi, J., El Mahi, M., & El Abbes Faouzi, M. (2021). Investigation of *Origanum compactum* essential oil for analgesic and anti-inflammatory activities. E3S Web of Conferences, 319. https://doi.org/10.1051/e3sconf/202131901104

Harput US, Saracoglu I, Ogihara Y. 2006. Effects of two *Prunella* species on lymphocyte proliferation and nitric oxide production. Phytotherapy Research 20(2):157-159. https://doi: 10.1002/ptr.1805. PMID: 16444671.

Hassan, Z. Y., Hassan, T. Y., & Abu-Raghif, A. R. (2022). Evaluation the Effectiveness of Phenolic Compound of *Salvia frigida* on Induced Atopic Dermatitis in Experimental Mice. *Iraqi Journal of Pharmaceutical Sciences*, *31*(1), 154–166. doi.org/10.31351/vol31iss1pp154-166

He, H., Jiang, H., Chen, Y., Ye, J., Wang, A., Wang, C., Liu, Q., Liang, G., Deng, X., Jiang, W., & Zhou, R. (2018). Oridonin is a covalent NLRP3 inhibitor with strong anti-inflammasome activity. *Nature Communications*, *9*(1). doi.org/10.1038/s41467-018-04947-6

Hernández-Herrera, A.D., Luna-Herrera, J., Del Rocío González-Martínez, M., Prieto-Hinojosa, A.I., Turcios-Esquivel, A.M., Castillo-Maldonado, I., Delgadillo-Guzmán, D., Ramírez-Moreno, A., Bustos-Brito, C., Esquivel, B., Vega-Menchaca, M.D., Pedroza-Escobar, D. (2023). Immunomodulatory activity of diterpenes over innate immunity and cytokine production in a human alveolar epithelial cell line infected with *Mycobacterium tuberculosis*. *Current Molecular Pharmacology*, 16(6), 682-689. doi: 10.2174/1874467215666221005115007.

Hernandez-Perez, M., Rabanal, R. M., De La Torre, M. C., & Rodriguez, B. (1995). Analgesic, anti-inflammatory, antipyretic and haematological effects of aethiopinone, an o-naphthoquinone diterpenoid from salvia aethiopis roots and two hemisynthetic derivatives. *Planta Medica*, 61(6), 505-509. doi:10.1055/s-2006-959358

Hong, S. S., Lee, C., Lee, C. H., Park, M., Lee, M. S., Hong, J. T., Lee, H., Lee, M. K., & Hwang, B. Y. (2009). A new furofuran lignan from *Isodon japonicus*. *Archives of Pharmacal Research*, 32(4), 501–504. https://doi.org/10.1007/s12272-009-1404-x

Hosseinzadeh, H. & Yavary, M. (1999). Anti-inflammatory effect of *Salvia leriifolia* Benth. leaf extract in mice and rats. *Pharmaceutical and Pharmacological Letters*, *9*(2), 60–61.

Hosseinzadeh, H., Haddadkhodaparast, M. H., & Arash, A. R. (2003). Antinociceptive, antiinflammatory and acute toxicity effects of *Salvia leriifolia* benth. Seed extract in mice and rats. *Phytotherapy Research*, *17*(4), 422–425. doi.org/10.1002/ptr.1154

Hsu, C. L., Hong, B. O. H., Shan, Y. U., & Yen, G. C. (2010). Antioxidant and Anti-Inflammatory effects of *Orthosiphon aristatus* and its bioactive compounds. *Journal of Agricultural and Food Chemistry*, *58*(4), 2150–2156. doi.org/10.1021/jf903557c

Huang, B. P., Lin, C. H., Chen, Y. C., & Kao, S. H. (2014). Anti-inflammatory effects of *Perilla frutescens* leaf extract on lipopolysaccharide stimulated RAW264.7 cells. *Molecular Medicine Reports*, *10*(2), 1077–1083. doi.org/10.3892/mmr.2014.2298

Hussain, L., Sajid Hamid Akash, M., Imran Qadir, M., Pharm Sci, P. J., Iqbal, R., Irfan, M., & Imran Qadir, M. (2017). Analgesic, anti-inflammatory, and antipyretic activity of *Salvia moorcroftiana*. *Article in Pakistan Journal of Pharmaceutical Sciences* 30(2). www.researchgate.net/publication/316007134.

Hwang, B., Lee, J., Koo, T., Kim, H., Hong, Y., Ro, J., Lee, K., & Lee, J. (2001). Kaurane diterpenes from *Isodon japonicus* inhibit nitric oxide and prostaglandin E2 production and NF-KB activation in LPS-stimulated macrophage RAW264.7 cells. *Planta Medica*, 67, 406–410.

Hwang, Y. J., Lee, E. J., Kim, H. R., & Hwang, K. A. (2013). NF-κB-targeted anti-inflammatory activity of *Prunella vulgaris* var. lilacina in macrophages RAW 264.7. *International Journal of Molecular Sciences*, *14*(11), 21489–21503. doi.org/10.3390/ijms141121489.

Ibrahim A. Y., Shahat A., Mohamed T., Elshamy A., Alqahtani A., Saleh I., Hassan E. & Hegazy M. 2020. In vitro anti-inflammatory and acetylcholinesterase inhibition efficiency of plant extracts from Sinai-Egypt. I*ndian Journal of Traditional Knowledge*, 19 (3): 635-641.

Ibrahim, A. Y., Youness, E. R., Shalaby, A. S., & Magd El-Din, M. (2016). Anti-acetylcholinesterase, Anti-inflammatory and Anti-oxidant Activities of *Mentha longifolia* for Treating Alzheimer Disease. *Der Pharmacia Lettre*, *8*(7), 34–39.

Ikoma, K., Takahama, M., Kimishima, A., Pan, Y., Taura, M., Nakayama, A., Arai, M., Takemura, N., & Saitoh, T. (2022). Oridonin suppresses particulate-induced NLRP3-independent IL-1α release to prevent crystallopathy in the lung. *International Immunology*, 34(10), 493–504. https://doi.org/10.1093/intimm/dxac018

Jang, H. H., Cho, S. Y., Kim, M. J., Kim, J. B., Lee, S. H., Lee, M. Y., & Lee, Y. M. (2016). Anti-inflammatory effects of *Salvia plebeia* R. Br extract in vitro and in ovalbumin-induced mouse model. *Biological Research*, *49*(1). doi.org/10.1186/s40659-016-0102-7

Jang, H. H., Lee, S., Lee, S. J., Lim, H. J., Jung, K., Kim, Y. H., Lee, S. W., & Rho, M. C. (2017). Anti-inflammatory Activity of Eudesmane-Type Sesquiterpenoids from *Salvia plebeia*. *Journal of Natural Products*, *80*(10), 2666–2676. doi.org/10.1021/acs.jnatprod.7b00326

Javdan, N., & Estakhr, J. (2011b). Evaluation of the effects of *Salvia hypoleuca* on the expression of cytokines: IL-6, IL-10 and TNF-α in high fat diet-fed mice towards a cure for diabetes mellitus. *Pharmacologyonline*, 2, 842-852. Retrieved from www.scopus.com

Jeong, H., Sung, M., Kim, Y., Ham, H., Choi, Y., & Lee, J. (2012). Anti-inflammatory activity of *Salvia plebeia* R. Br. leaf through heme oxygenase-1 induction in LPS-stimulated RAW264.7 macrophages. *Journal of the Korean Society of Food Science and Nutrition*, *41*(7), 888–894. doi.org/10.3746/jkfn.2012.41.7.888

Jia, T., Cai, M., Ma, X., Li, M., Qiao, J., & Chen, T. (2019). Oridonin inhibits IL-1β-induced inflammation in human osteoarthritis chondrocytes by activating PPAR-γ. *International Immunopharmacology*, 69, 382–388. doi.org/10.1016/j.intimp.2019.01.049

Jiang, H., Li, X., Sun, H., Zhang, H., Puno, P. (2018). Scopariusols L-T, nine new ent-kaurane diterpenoids isolated from *Isodon scoparius*. *Chinese Journal of Natural Medicines*, 16(6), 456–464.

Jiang, H.-Y., Wang, W.-G., Zhou, M., Wu, H.-Y., Zhan, R., Du, X., Pu, J.-X., Sun, H.-D. (2014a). 6,7-seco-ent-Kaurane diterpenoids from *Isodon sculponeatus* and their bioactivity. *Chinese Chemical Letters*, 25, 541–544. http://dx.doi.org/10.1016/j.cclet.2014.01.041

Jiang, H.-Y., Wang, W.-G., Zhou, M., Wu, H.-Y., Zhan, R., Li, X.-N., Du, X., Li, Y., Pu, J.-X., Sun, H.-D. (2014b). Diterpenoids from *Isodon sculponeatus*, *Fitoterapia*, 93, 142–149. http://dx.doi.org/10.1016/j.fitote.2013.12.025.

Kamatou, G. P. P., & Viljoen, A. M. (2010). A review of the application and pharmacological properties of α-bisabolol and α-bisabolol-rich oils. *JAOCS, Journal of the American Oil Chemists' Society*, 87(1), 1-7. doi:10.1007/s11746-009-1483-3

Kamatou, G. P. P., Makunga, N. P., Ramogola, W. P. N., & Viljoen, A. M. (2008). South african *Salvia* species: A review of biological activities and phytochemistry. *Journal of Ethnopharmacology*, 119(3), 664-672. doi:10.1016/j.jep.2008.06.030

Kamatou, G. P. P., Van Zyl, R. L., Van Vuuren, S. F., Viljoen, A. M., Figueiredo, A. C., Barroso, J. G. & Tilney, P. M. (2006). Chemical composition, leaf trichome types and biological activities of the essential oils of four related *Salvia* species indigenous to southern Africa. *Journal of Essential Oil Research*, 18, 72-79. doi:10.1080/10412905.2006.12067125

Kamatou, G. P. P., Viljoen, A. M., & Steenkamp, P. (2010). Antioxidant, antiinflammatory activities and HPLC analysis of south african *Salvia* species. *Food Chemistry*, 119(2), 684-688. doi:10.1016/j.foodchem.2009.07.010.

Kamatou, G. P. P., Viljoen, A. M., Gono-Bwalya, A. B., Van Zyl, R. L., Van Vuuren, S. F., Lourens, A. C. U., Steenkamp, P. (2005). The *in vitro* pharmacological activities and a chemical investigation of three south african *Salvia* species. *Journal of Ethnopharmacology*, 102(3), 382-390. doi:10.1016/j.jep.2005.06.034

Kamatou, G.P.P., Van Zyl, R.L., Van Vuuren, S.F., Viljoen, A.M., Figueiredo, A.C., Barroso, J.G., Pedro, L.G. (2006). Biological activities and composition of *Salvia muirii* L. essential oil. *Journal of Essential Oil Research* 18, 48-51. https://doi.org/10.1080/10412905.2006.12067119.

Kamatou, G.P.P., Viljoen, A.M., Figueiredo, A.C., Tilney, P.M., Van Zyl, R.L., Barroso, J.G., Pedro, L.G., Van Vuuren, S.F. (2007). Trichomes, essential oil composition and biological activities of *Salvia albicaulis* Benth. And *S. dolomitica* Codd, two species from the Cape region of South Africa. *South African Journal of Botany* 73: 102-108. https://doi.org/10.1016/j.sajb.2006.08.001

Kang, H. (2018). Anti-inflammatory mechanism of I*sodon Japonicas* (Burm) hara on lipopolysaccharide-induced neuroinflammation. *Tropical Journal of Pharmaceutical Research*, 17(1), 85–89. https://doi.org/10.4314/tjpr.v17i1.13

Kangwan, N., Pintha, K., Khanaree, C., Kongkarnka, S., Chewonarin, T., & Suttajit, M. (2021). Anti-inflammatory effect of *Perilla frutescens* seed oil rich in omega-3 fatty acid on dextran sodium sulfate-induced colitis in mice. *Research in Pharmaceutical Sciences*, *16*(5), 464–473. doi.org/10.4103/1735-5362.323913

Kapewangolo, P., Hussein, A. A., & Meyer, D. (2013). Inhibition of HIV-1 enzymes, antioxidant and anti-inflammatory activities of *Plectranthus barbatus*. *Journal of Ethnopharmacology*, 149(1), 184–190. doi.org/10.1016/j.jep.2013.06.019

Kapewangolo, P., Omolo, J.J., Bruwer, R., Fonteh, P., Meyer, D. (2015). Antioxidant and anti-inflammatory activity of *Ocimum labiatum* extract and isolated labdane diterpenoid. *Journal of Inflammation* 12, 4.doi: 10.1186/s12950-015-0049-4.

Karaca, N., Demirci, B., Gavahian, M., Demirci, F. (2023). Enhanced Bioactivity of Rosemary, Sage, Lavender, and Chamomile Essential Oils by Fractionation, Combination, and Emulsification. *ACS Omega*, 8(12), pp. 10941–10953.

Karakaş, Ö., & Bekler, F. M. (2022). Essential oil compositions and antimicrobial activities of *Thymbra spicata* L. var. *spicata* L., *Lavandula X intermedia* Emeric ex Loisel., *Satureja macrantha* C. A. MEYER and *Rosmarinus*

officinalis L. *Brazilian Archives of Biology and Technology*, 65, 1-11. doi:10.1590/1678-4324-2022210297.

Karimian, P., Kavoosi, G., & Amirghofran, Z. (2013). Anti-inflammatory effect of *Mentha longifolia* in lipopolysaccharide- stimulated macrophages: Reduction of nitric oxide production through inhibition of inducible nitric oxide synthase. *Journal of Immunotoxicology*, *10*(4), 393–400. doi.org/10.3109/1547691X.2012.758679

Kenari, H.M., Yousefsani, B.S., Eghbalian, F., ...Jamshidi, A.H., Mahroozade, S. (2021). Herbal recommendations for treatment of COVID-19 symptoms according to Persian medicine. *Journal of Medicinal Plants*, 20(77), pp. 1–14

Khalighi-Sigaroodi, F., Ahvazi, M., Ebrahimzadeh, H., & Rahimifard, N. (2013). Chemical composition of the essential oil and antioxidant activities, total phenol and flavonoid content of the extract of *Nepeta pogonosperma*. *Journal of Medicinal Plants*, 12(48), 185–199.

Khan, S., & Iqbal Yatoo, M. (n.d.). Potential herbs for bovine mastitis research-a mini review Anti-Neoplastic effects of Garcinol View project NFBSFARA project in 2010-11 session View project. https://www.researchgate.net/publication/358925222

Khankandi, H. P., Behzad, S., Mojab, F., Ahmadian-Attari, M. M., & Sahranavard, S. (2019). Effects of some Lamiaceae species on NO production and cell injury in hydrogen peroxide-induced stress. *Iranian Journal of Pharmaceutical Research*, *18*(2), 826–835. doi.org/10.22037/ijpr.2019.1100685

Khazdair, M. R., Ghorani, V., Alavinezhad, A., & Boskabady, M. H. (2018). Pharmacological effects of *Zataria multiflora* Boiss L. and its constituents focus on their anti-inflammatory, antioxidant, and immunomodulatory effects. In *Fundamental and Clinical Pharmacology* (Vol. 32, Issue 1, pp. 26–50). Blackwell Publishing Ltd. doi.org/10.1111/fcp.12331

Khouchlaa, A., Et-Touys, A., Lakhdar, F., ...El Idrissi, A.E.Y., Zaakour, F. (2022). Ethnomedicinal use, phytochemistry, pharmacology, and toxicology of *Salvia verbenaca* L.: A review. *Biointerface Research in Applied Chemistry*, 12(2), pp. 1437–1469.

Kim, B. W., Koppula, S., Kim, I. S., Lim, H. W., Hong, S. M., Han, S. D., Hwang, B. Y., & Choi, D. K. (2011). Anti-neuroinflammatory activity of kamebakaurin from *Isodon japonicus* via inhibition of c-Jun NH2-terminal kinase and p38 mitogen-activated protein kinase pathway in activated microglial cells. *Journal of Pharmacological Sciences*, 116(3), 296–308. https://doi.org/10.1254/jphs.10324FP

Kim, B., Han, Y. E., & Lee, H. J. (2017). Antioxidant and anti-inflammatory effects of the ethyl acetate fraction of the *Agastache rugosa* extract. *Korean Journal of Food Science and Technology*, *49*(3), 331–337. doi.org/10.9721/KJFST.2017.49.3.331

Kim, D., Lee, S.K., Park, K.-S., Park, H.-J. 2020. Isolation of the constituents from *Clinopodium chinense* var. *shibetchense* and inhibition activity on cancer cell growth and nitric oxide production. *Korean Journal of Pharmacognosy*, 51(2), pp. 93–99.

Kim, H., Hong, J. Y., Jeon, W. J., Lee, J., Baek, S. H., & Ha, I. H. (2021). *Lycopus lucidus* Turcz. exerts neuroprotective effects against H2O2-induced neuroinflammation by inhibiting nlrp3 inflammasome activation in cortical neurons. *Journal of Inflammation Research*, 14, 1759–1773. https://doi.org/10.2147/JIR.S305031

Kim, H.-H., Yoo, J.-S., Lee, H.-S., ...Shin, T.-Y., Kim, S.-H. (2011). *Elsholtzia ciliata* inhibits mast cell-mediated allergic inflammation: Role of calcium, p38 mitogen-activated protein kinase and nuclear factor-kB. *Experimental Biology and Medicine*, 236(9), pp. 1070–1077.

Kim, J. Y., Kim, H. S., Kim, Y. J., Lee, H. K., Kim, J. S., Kang, J. S., Hong, J. T., Kim, Y., Hwang, B. Y., & Han, S. B. (2013). Effusanin C inhibits inflammatory responses via blocking NF-κB and MAPK signaling in monocytes. *International Immunopharmacology*, 15(1), 84–88. https://doi.org/10.1016/j.intimp.2012.11.002

Kim, J., Song, S., Lee, I., Kim, Y., Yoo, I., Ryoo, I., & Bae, K. (2011). Anti-inflammatory activity of constituents from *Glechoma hederacea* var. l*ongituba*. *Bioorganic and Medicinal Chemistry Letters*, *21*(11), 3483–3487. doi.org/10.1016/j.bmcl.2011.02.002

Kim, K. M., Kim, S. Y., Mony, T. J., Bae, H. J., Han, S. D., Lee, E. S., Choi, S. H., Hong, S. H., Lee, S. D., & Park, S. J. (2021). *Dracocephalum moldavica* ethanol extract suppresses LPS-induced inflammatory responses through inhibition of the jnk/erk/nf-κb signaling pathway and il-6 production in raw 264.7 macrophages and in endotoxic-treated mice. *Nutrients*, *13*(12). doi.org/10.3390/nu13124501

Kim, M.; Sowndhararajan, K.; Kim, S. 2022. The Chemical Composition and Biological Activities of Essential Oil from Korean Native Thyme Bak-Ri-Hyang (*Thymus quinquecostatus* Celak.). *Molecules* 2022,27,4251. doi.org/10.3390/molecules27134251.

Kim, S. Y., Han, S. D., Kim, M., Mony, T. J., Lee, E. S., Kim, K. M., Choi, S. H., Hong, S. H., Choi, J. W., & Park, S. J. (2021b). *Mentha Arvensis* essential oil exerts anti-inflammatory in LPS-stimulated inflammatory responses via inhibition of ERK/nf-κb signaling pathway and anti-atopic dermatitis-like effects in 2,4-dinitrochlorobezene-induced BALB/C mice. *Antioxidants*, *10*(12). doi.org/10.3390/antiox10121941

Kim, T.-W., Kim, Y.-J., Seo, C.-S., ...Lee, M.-Y., Jung, J.-Y. (2016). *Elsholtzia ciliata* (Thunb.) Hylander attenuates renal inflammation and interstitial fibrosis via regulation of TGF-ß and Smad3 expression on unilateral ureteral obstruction rat model. *Phytomedicine*, 23(4), pp. 331–339.

Kim, W., Son, K., Chang, H., Bae, K., Kang, S., Kim, H. (2003). Anti-Inflammatory activity of *Elsholtzia splendens*. *Archives of Pharmacal Researchs*, 26(3), 232–236.

Ko, H.M., Koppula, S., Kim, B.-W., Kim, I.S., Hwang, B.Y., Suk, K., Park, E.J., Choi, D.-K. (2010). *European Journal of Pharmacology*, 633, 98–106. doi:10.1016/j.ejphar.2010.02.011.

Kogiannou, D.A.A., Kalogeropoulos, N., Kefalas, P., Polissiou, M.G., Kaliora, A.C. (2013). Herbal infusions; their phenolic profile, antioxidant and anti-inflammatory effects in HT29 and PC3 cells. *Food and Chemical Toxicology*, 61, 152–159. http://dx.doi.org/10.1016/j.fct.2013.05.027.

Kosar, M.; Goger, F.; Can Baser, K.H. *In vitro* antioxidant properties and phenolic composition of *Salvia virgata* Jacq. from Turkey. *J. Agric. Food Chem*., 2008, 6, 2369-2374.

Kostić, M., Kitić, D., Petrović, M. B., Jevtović-Stoimenov, T., Jović, M., Petrović, A., & Živanović, S. (2017). Anti-inflammatory effect of the *Salvia sclarea* L. ethanolic extract on lipopolysaccharide-induced periodontitis in rats. *Journal of Ethnopharmacology*, 199, 52–59. doi.org/10.1016/j.jep.2017.01.020

Kulabas, S. S., Ipek, H., Tufekci, A. R., Arslan, S., Demirtas, I., Ekren, R., Sezerman, U., & Tumer, T. B. (2018). Ameliorative potential of *Lavandula stoechas* in metabolic syndrome via multitarget interactions. *Journal of Ethnopharmacology*, *223*, 88–98. doi.org/10.1016/j.jep.2018.04.043

Kumar, A., Singh, S., Kumar, A., Umrao Bawankule, D., Tandon, S., Kumar, A., Swaroop Verma, R., Saikia, D. (2021). Chemical composition, bactericidal kinetics, mechanism of action, and anti-inflammatory activity of *Isodon melissoides* (Benth.) H. Hara essential oil, *Natural Product Research*, 35:4, 690-695. https://doi.org/10.1080/14786419.2019.1591399.

Laura González Pereyra, M., Cariddi, L. N., Ybarra, F., Isola, M. C., Demo, M. S., Sabini, L., & Maldonado, A. M. (n.d.). Immunomodulating properties of *Minthostachys verticillata* on human lymphocytes and basophils. www.revistasmedicasmexicanas.com.mx

Lee, J.W., Lee, M.S., Kim, T.H., Lee, H.J., Hong, S.S., Noh, Y.H., Hwang, B.Y., Ro, J.S., Hong, J.T. (2007). Inhibitory effect of Inflexinol on Nitric Oxide Generation and iNOS expression via inhibition of NF-κB activation. *Mediators of Inflammation*, 2007, 93148. doi: 10.1155/2007/93148.

Lee, Y. J., Kang, D. G., Kim, J. S., & Lee, H. S. (2008). *Lycopus lucidus* inhibits high glucose-induced vascular inflammation in human umbilical vein endothelial cells. Vascular Pharmacology, 48(1), 38–46. https://doi.org/10.1016/j.vph.2007.11.004

Lee, Y., Lim, H. W., Ryu, I. W., Huang, Y. H., Park, M., Chi, Y. M., & Lim, C. J. (2020). Anti-Inflammatory, Barrier-Protective, and Antiwrinkle Properties of *Agastache rugosa* Kuntze in Human Epidermal Keratinocytes. *BioMed Research International*, *2020*. doi.org/10.1155/2020/1759067

Li C., Luo Y., Zhang W., Cai Q., Wu X., Tan Z., Chen R., Chen Z., Wang S., Zhang L. 2021. A comparative study on chemical compositions and biological activities of four essential oils: *Cymbopogon citratus* (DC.) Stapf, *Cinnamomum cassia* (L.) Presl, *Salvia japonica* Thunb. and *Rosa rugosa* Thunb. *Journal of Ethnopharmacology* 280:114472. https://doi: 10.1016/j.jep.2021.114472

Li L, Zhou M, Xue G, Wang W, Zhou X, Wang X, Kong L, Luo J. 2018. Bioactive seco-abietane rearranged diterpenoids from the aerial parts of *Salvia prionitis*. *Bioorganic Chemistry* 81:454-460. https://doi: 10.1016/j.bioorg.2018.08.040

Li, J., Du, J., Sun, L., Liu, J., & Quan, Z. (2010). Anti-Inflammatory Function of Nodosin via Inhibition of IL-2. *The American Journal of Chinese Medicine* (Vol. 38, Issue 1). www.worldscientific.com

Li, L., Huang, Q., Duan, X., Han, L., Peng, D. (2020). Protective effect of *Clinopodium chinense* (Benth.) O. Kuntze against abnormal uterine bleeding in female rats. *Journal of Pharmacological Sciences*, 143(1), pp. 1–8

Li, L.-L., Huang, Q., Peng, D.-Y., Qi, J.-J., Yao, M. (2022). Anti-inflammatory and hemostatic effects of total extract, saponins, and flavonoids of *Clinopodium chinense* in female rats with abnormal uterine bleeding and mechanism. *Zhongguo Zhongyao Zazhi*, 47(12), pp. 3372–337

Li, Y. X., Liu, Y. B., Ma, A. Q., Bao, Y., Wang, M., & Sun, Z. L. (2017). In vitro antiviral, anti-inflammatory, and antioxidant activities of the ethanol extract of *Mentha piperita* L. *Food Science and Biotechnology*, 26(6), 1675–1683. doi.org/10.1007/s10068-017-0217-9

Li, Y.-M., Zou, H.-Y., Hu, H.-Y., Di, L., Li, H.-L., Hu, Z., Lin, L., Liao, D., Xia, B., & Lin, Y. (2020). Effect of water extract of *Prunella vulgaris* on NLRP3/Caspase-1/IL-1B pathway in rats with allergic conjunctivitis. *Chinese Journal of New Drugs*, 29(8), 929–933. doi.org/10.1097/ACI.0000000000000100

Lim, H. W., Lee, Y., Huang, Y. H., Yoon, J. Y., Lee, S. H., Kim, K., & Lim, C. J. (2017). Enhancement of skin antioxidant and anti-inflammatory potentials of *Agastache rugosa* leaf extract by probiotic bacterial fermentation in human epidermal keratinocytes. *Microbiology and Biotechnology Letters*, *45*(1), 35–42. doi.org/10.4014/mbl.1701.01002.

Lim, J. Y., Won, T. J., Hwang, B. Y., Kim, H. R., Hwang, K. W., Sul, D., & Park, S. Y. (2010). The new diterpene isodojaponin D inhibited LPS-induced microglial activation through NF-kappaB and MAPK signaling pathways. *European Journal of Pharmacology*, 642(1–3), 10–18. https://doi.org/10.1016/j.ejphar.2010.05.047

Ling-Fei, K., Xiao-Juan, R., Pan, Y., ...Tian-Le, G., Cai, T. (2023). The influence of *Hyssopus cuspidatus* Boriss extract on lipid mediators metabolism network in asthmatic mice. *Frontiers in Pharmacology*, 14, 1066643.

Lino, C.S., Gomes, P.B., Lucetti, D.L., ...Silva, M.G.V., Viana, G.S.B. (2005). Evaluation of antinociceptive and antiinflammatory activities of the essential oil (EO) of *Ocimum micranthum* Willd. from northeastern Brazil. *Phytotherapy Research*, 19(8), pp. 708–712.

Liu, D., Wang, Y., Lu, Z., Lv, F., Bie, X., & Zhao, H. (2022b). Separation, characterization, and anti-inflammatory activities of galactoglycerolipids from *Perilla frutescens* (L.) Britton. *Natural Product Research*. doi.org/10.1080/14786419.2022.2095637.

Liu, X., Su, J., Wang, G., ...Wang, S., Huang, Y. (2021). Discovery of phenolic glycoside from *Hyssopus cuspidatus* attenuates LPS-induced inflammatory responses by inhibition of iNOS and COX-2 expression through suppression of NF-κB activation. *International Journal of Molecular Sciences*, 22(22), 12128.

Liu, X., Wang, G., Lv, C., ...Wang, S., Huang, Y. (2021). Two new phenolic acids and one new phenolic glycoside from *Hyssopus cuspidatus* Boriss and their anti-inflammatory activities. *Phytochemistry Letters*, 46, pp. 15–20.

Liu, Y., Song, H., Xu, J., Bi, G., & Meng, D. (2022). Anti-inflammatory abietanes diterpenes and triterpenoids isolated from *Clinopodium polycephalum*. *Fitoterapia, 161*. doi.org/10.1016/j.fitote.2022.105244

Llorent-Martínez, E. J., Ruiz-Medina, A., Zengin, G., Ak, G., Jugreet, S., Mahomoodally, M. F., Chiavaroli, A. (2022). New biological and chemical evidences of two Lamiaceae species (*Thymbra capitata* and *Thymus sipyleus* subsp. *rosulans*): In vitro, in silico and ex vivo approaches. *Molecules*, 27(24) doi:10.3390/molecules27249029

Loizzo, M., Menichini, F., Tundis, R., Bonesi, M., Conforti, F., Nadjafi, F., Statti, G., Frega, N., & Menichini, F. (2009). In vitro biological activity of *Salvia lerrifolia* Benth essential oil relevant to the treatment of Alzheimer´s disease. *Journal of Oleo Science, 58*(8), 443–446.

Ĺopez-Garcia, R.E., Rabanal, R.M., Darias, V., Martín-Herrera, D., Carreiras, M.C. and Rodríguez, B. (1991), A preliminary study of *Cedronella canariensis* (L.) var. Canariensis extracts for antiinflammatory and analgesic activity in rats and mice. *Phytotherapy Research*, 5: 273-275. doi.org/10.1002/ptr.2650050610

Lucca, L.G., Romão, P.R.T., Vignoli-Silva, M., da VeigaJunior, V.F., Koester, L.S. (2022). *In vivo* Acute Anti-Inflammatory Activity of Essential Oils: A Review. *Mini-Reviews in Medicinal Chemistry*, 22(11), pp. 1495–1515.

Luís, Â., & Domingues, F. (2021). Screening of the potential bioactivities of pennyroyal (*Mentha pulegium* L.) essential oil. *Antibiotics*, *10*(10). doi.org/10.3390/antibiotics10101266

Ma, X., Ma, X., Ma, Z., Wang, J., Sun, Z., Yu, W., Li, F., & Ding, J. (2014). Effect of *Hyssopus officinalis* L. on inhibiting airway inflammation and immune regulation in a chronic asthmatic mouse model. *Experimental and Therapeutic Medicine*, *8*(5), 1371–1374. doi.org/10.3892/etm.2014.1978

Magalhães, A., Santos, G. B., Verdam, M. C., Fraporti, L., Malheiro, A., Lima, E. S., & Dos-Santos, M. C. (2011). Inhibition of the inflammatory and coagulant action of Bothrops atrox venom by the plant species *Marsypianthes chamaedrys*. *Journal of Ethnopharmacology*, 134(1), 82–88. doi.org/10.1016/j.jep.2010.11.062.

Mahalakshmi, B., Huang, C. Y., Lee, S. da, Maurya, N., kiefer, R., & Bharath Kumar, V. (2021). Review of Danshen: From its metabolism to possible mechanisms of its biological activities. In *Journal of Functional Foods* 85. Elsevier Ltd. doi.org/10.1016/j.jff.2021.104613

Mahmoud AM, Wilkinson FL, Sandhu MA, Dos Santos JM, Alexander MY. 2019. Modulating oxidative stress in drug-induced injury and metabolic disorders: the role of natural and synthetic antioxidants. *Oxidative Medicine and Cellular Longevity* 2019:3206401. https://doi: 10.1155/2019/3206401.

Mahmoudi, M., Morteza-Semnani, K., & Mojra, E. (2008). Anti-inflammatory and antinociceptive activity of *Thymus pubescens* extract. *Fitoterapia*, *79*(5), 361–365. doi.org/10.1016/j.fitote.2008.04.004

Mahomoodally, M. F., Devi Dursun, P., & Venugopala, K. N. (2021). *Collinsonia canadensis* L. In *Naturally Occurring Chemicals against Alzheimer's Disease*, 373–377. Elsevier. doi.org/10.1016/B978-0-12-819212-2.00031-1

Maklad, Y. A., Aboutabl, E. A., El-Sherei, M. M., & Meselhy, K. M. (1999). Bioactivity studies of *Salvia transsylvanica* (Schur ex Griseb) grown in Egypt. *Phytotherapy Research*, *13*(2), 147–150. doi.org/10.1002/(SICI)1099-1573(199903)13:2<147:AID-PTR397>3.0.CO;2-E

Martins-Gomes, C., Taghouti, M., Schäfer, J., Silva, A.M., Nunes, F.M. 2018. Chemical characterization and bioactive properties of decoctions and hydroethanolic extracts of *Thymus carnosus* Boiss. *Journal of Functional Foods*, 43, pp. 154–164.

Mayadah B. Shehadeh, Silvio Sosa, Ghadeer A.R.Y. Suaifan, Rula M. Darwish, Anna Giangaspero, Antonio Vassallo, Laura Lepore, Sawsan A. Oran, Hana Hammad, Aurelia Tubaro, Nunziatina De Tommasi, Roberto Della Loggia. 2014. Topical Anti-Inflammatory Potential of Six *Salvia* Species Grown in Jordan. *Jordan Journal of Pharmaceutical Sciences*, 7, No. 2, 2014.

Mehmet, S. I., Gürbüz, I., Bedir, E., Günbatan, T., Demirci, F., 2021. Isolation of rosmarinic acid and methyl rosmarinate as lipoxygenase inhibitors from *Salvia palaestina* Benth. by activity-guided fractionation. *South African Journal of Botany* 141: 177-182. https://doi.org/10.1016/j.sajb.2021.04.030

Memariani, Z., Rahimi, A., Farzaei, M.H., Zakaria Nejad, N. (2019). *Nepeta menthoides* Boiss. & Buhse, an endemic species in Iran: A review of traditional uses, phytochemistry and pharmacology. *Journal of Herbmed Pharmacology*, 8(3), 194-204. doi: 10.15171/jhp.2019.29.

Messaoudi, M., Rebiai, A., Sawicka, B., Atanassova, M., Ouakouak, H., Larkem, I., Egbuna, C., Awuchi, C. G., Boubekeur, S., Ferhat, M. A., Begaa, S., & Benchikha, N. (2022). Effect of extraction methods on polyphenols, flavonoids, mineral elements, and biological activities of essential oil and extracts of *Mentha pulegium* L. *Molecules*, *27*(1). doi.org/10.3390/molecules27010011

Mićović, T., Katanić Stanković, J. S., Bauer, R., Nöst, X., Marković, Z., Milenković, D., Jakovljević, V., Tomović, M., Bradić, J., Stešević, D., Stojanović, D., & Maksimović, Z. (2022). In vitro, in vivo and in silico evaluation of the anti-inflammatory potential of *Hyssopus officinalis* L. subsp. aristatus (Godr.) Nyman (Lamiaceae). *Journal of Ethnopharmacology*, 293. doi.org/10.1016/j.jep.2022.115201

Miguel, M. G., da Silva, C. I., Farah, L., Braga, F. C., & Figueiredo, A. C. (2021). Effect of essential oils on the release of TNF-α and CCL2 by LPS-stimulated THP-1 cells. *Plants*, *10*(1), 1–14. doi.org/10.3390/plants10010050

Milijasevic, B., Steinbach, M., Mikov, M., Raskovic, A., Capo, I., Zivkovic, J., Borisev, I., Canji, J., Teofilovic, B., Vujcic, M., & Popovic, M. (2022). Impact of winter savory extract (*Satureja montana* L.) on biochemical parameters in serum and oxidative status of liver with application of the principal component analysis in extraction solvent selection. *European Review for Medical and Pharmacological Sciences*, *26*, 4721–4734.

Miller KI, Qing C, Sze DM, Neilan BA. (2012). Investigation of the Biosynthetic Potential of Endophytes in Traditional Chinese Anticancer Herbs. *PLoS ONE* 7(5): e35953. doi:10.1371/journal.pone.0035953

Min, G. Y., Kim, E. Y., Hong, S., Kim, J. H., Kim, M., Kim, E. J., Park, J. H., Sohn, Y., & Jung, H. S. (2021). *Lycopus lucidus* Turcz. ameliorates DNCB-induced atopic dermatitis in BALB/c mice. *Molecular Medicine Reports*, 24(6). https://doi.org/10.3892/mmr.2021.12467.

Minaiyan, M., Sadraei, H., Asghari, G., Khanabadi, M., & Minaiyan, M. (2017). Anti-inflammatory effect of apigenin and hydroalcoholic extract of *Dracocephalum kotschyi* on acetic acid-induced colitis in rats. *Research in Pharmaceutical Science*s (Vol. 12, Issue 4).

Minaiyan, M., Sadraei, H., Yousefi, I., Sajjadi, S. E., & Talebi, A. (2021). Evaluation of the effect of hydroalcoholic and flavonoid-enriched extracts of *Dracocephalum kotschyi* on indomethacin-induced gastric ulcer in rats. *Research in Pharmaceutical Sciences*, 16(2), 141–152. https://doi.org/10.4103/1735-5362.310521

Miraj, S., & Kiani, S. (2016). A review study of therapeutic effects of *Salvia officinalis* L. *Der Pharmacia Lettre*, *8*(6), 299–303. www.researchgate.net/publication/305147951

Miranda R.S., de Jesus B.D.S.M., da Silva Luiz S.R., Viana C.B., Adão Malafaia C.R., Figueiredo F.S., Carvalho T.D.S.C., Silva M.L., Londero V.S., da Costa-Silva T.A., Lago J.H.G., Martins R.C.C. (2022). Antiinflammatory activity of natural triterpenes-An overview from 2006 to 2021. *Phytother Research* 36(4):1459-1506. https://doi: 10.1002/ptr.7359.

Modarres, M., Asili, J., Lahouti, M., Gangali, A., Iranshahy, M., & Sahebkar, A. (2014). Simultaneous determination of rosmarinic acid, salvianolic acid B and caffeic acid in *Salvia leriifolia* Benth. root, leaf and callus extracts using a high-performance liquid chromatography with diode-array detection technique. *Journal of Liquid Chromatography and Related Technologies*, *37*(12), 1721–1730. doi.org/10.1080/10826076.2013.807466

Mogosan, C., Vostinaru, O., Oprean, R., Heghes, C., Filip, L., Balica, G., Moldovan, R. I., & Schmidt, T. J. (2017). A comparative analysis of the chemical composition, anti-inflammatory, and antinociceptive effects of the essential oils from three species of *Mentha* cultivated in Romania. *Molecules*, 22(2). https://doi.org/10.3390/molecules22020263

Mohanty, S., Kamolvit, W., Zambrana, S., Sandström, C., Gonzales, E., Östenson, C. G., & Brauner, A. (2017). Extract of *Clinopodium bolivianum* protects against E. coli invasion of uroepithelial cells. *Journal of Ethnopharmacology*, *198*, 214–220. doi.org/10.1016/j.jep.2017.01.011

Mohsenzadeh, A., Ahmadipour, S., Ahmadipour, S., & Asadi-Samani, M. (2016). A review of the most important medicinal plants effective on cough in children and adults. *Der Pharmacia Lettre*, 8(1), 90-96.

Momtaz, S. and Abdollahi, M.. (2010). An Update on Pharmacology of *Satureja* Species; From Antioxidant, Antimicrobial, Antidiabetes and Anti-hyperlipidemic to Reproductive Stimulation. *International Journal of Pharmacology* 6 (4): 346-353.

Montironi, I. D., Reinoso, E. B., Paullier, V. C., Siri, M. I., Pianzzola, M. J., Moliva, M., Campra, N., Bagnis, G., Ferreira LaRocque-de-Freitas, I., Decote-Ricardo, D., Freire-de-Lima, C. G., Raviolo, J. M., & Cariddi, L. N. (2019). *Minthostachys verticillata* essential oil activates macrophage phagocytosis and modulates the innate immune response in a murine model of Enterococcus faecium mastitis. *Research in Veterinary Science*, 125, 333–344. https://doi.org/10.1016/j.rvsc.2019.07.015

Moraes, L. A. S., Facanali, R., Ortiz, M., Marques, M., Ming, L. C., Angela, M., & Meireles, A. (2002). Phytochemical characterization of essential oil from *Ocimum selloi*. 74(1), 183–186. www.scielo.br/aabc

Moreno, L., Bello, R., Primo, E., & Esplugues, J. (2002). Pharmacological properties of the methanol extract from *Mentha suaveolens* Ehrh. *Phytotherapy Research*, 16(S1), 10–13. https://doi.org/10.1002/ptr.744

Moretti, M. D. L., Peana, A. T., & Satta, M. (1997). A study on anti-inflammatory and peripheral analgesic action of *Salvia sclarea* oil and its main components. *Journal of Essential Oil Research*, *9*(2), 199–204. doi.org/10.1080/10412905.1997.9699459

Moshrefi-Araghi, A., Nemati, H., Azizi, M., Moshtaghi, N., & Shoor, M. (2021). Association of Genetic Structure and Diversity in Iranian Wild Germplasms of *Mentha longifolia* L. Based on Phenotypical, Biochemical, and Molecular Markers. *Chemistry and Biodiversity*, *18*(4). doi.org/10.1002/cbdv.202001044

Mousavi Nia, A., Pari Kalantaripour, T., Basiri, M., Vafaee, F., Asadi-Shekaari, M., Eslami, A., & Darvish Zadeh, F. (2017). *Nepeta Dschuparensis* Bornm Extract Moderates COX-2 and IL-1β Proteins in a Rat Model of Cerebral Ischemia. *Iran Journal of Medicine Sciences*, *42*(2), 179–186.

Mousavi, Z., Hosaini, A. S., Asgarpanah, J., & Najafizadeh, P. (2016). Antinociceptive effect of the endemic species *Nepeta depauperata* Benth. *Jundishapur Journal of Natural Pharmaceutical Products*, *11*(2). doi.org/10.17795/jjnpp-25623

Moussaid, M., Elamrani, A. E., Bourhim, N., & Benaissa, M. (2011). *In Vivo* Anti-Inflammatory and *In Vitro* Antioxidant Activities of Moroccan Medicinal Plants. In *NPC Natural Product Communications* (Vol. 6, Issue 10).

Murad, H. A. S., Abdallah, H. M., & Ali, S. S. (2016). *Mentha longifolia* protects against acetic-acid induced colitis in rats. *Journal of Ethnopharmacology*, *190*, 354–361. doi.org/10.1016/j.jep.2016.06.016

Murata, T., Oyama, K., Fujiyama, M., Oobayashi, B., Umehara, K., Miyase, T., & Yoshizaki, F. (2013). Diastereomers of lithospermic acid and lithospermic acid B from *Monarda fistulosa* and *Lithospermum erythrorhizon*. *Fitoterapia*, 91, 51–59. https://doi.org/10.1016/j.fitote.2013.08.009

Mustarichie, R., Moektiwardojo, M., Apriani Dewi W. (2017). Isolation, Identification, and Characteristic of Essential Oil of Iler (*Plectranthus scutellarioides* (L.) R.Br leaves. *Journal of Pharmaceutical Sciences and Research*, 9(11), 2218-2223.

Mustarichie, R., Ramdhani, D., Mekar Saptarini, N. (2022). The anti-inflammatory tablet formulation of *Coleus* (*Plectranthus scutellariodes*) leaves extract using Kollicoat® protect coating. *International Journal of Applied Pharmaceutics*, 14(4), 159-162.

Müzell, D., Lunardelli, A., Leite, C. E., Medeiros Fagundes, R., Saciura, V. C., Reichel, C. L., Rodrigues De Oliveira, J., & Vieira Astarita, L. (2013). Nephroprotective and Anti-inflammatory Effects of Aqueous Extract of *Melissa officinalis* L. on Acetaminophen-induced and Pleurisy-induced Lesions in Rats. *Brazilian Archives of Biology and Technology*, *56*(3), 383–392.

Nahak, G., & Sahu, R. (2014). Immunomodulatory activity of aqueous leaf extract of *Ocimum kilimandscharicum* Guerke in *Clarias batrachus*. *Int. J. Pharm. Sci. Rev. Res.*, 28(2), 137–146.

Napagoda M, Gerstmeier J, Butschek H, Lorenz S, De Soyza S, Qader M, Nagahawatte A, Wijayaratne GB, Schneider B, Svatoš A, Jayasinghe L, Koeberle A, Werz O. 2022. *Plectranthus zeylanicus*: A rich source of secondary metabolites with antimicrobial, disinfectant and anti-inflammatory activities. *Pharmaceuticals (Basel)* 15(4):436. https://doi: 10.3390/ph15040436.

Napagoda M, Gerstmeier J, Wesely S, Popella S, Lorenz S, Scheubert K, Svatoš A, Werz O. 2014. Inhibition of 5-lipoxygenase as anti-inflammatory mode of action of *Plectranthus zeylanicus* Benth. and chemical characterization of ingredients by a mass spectrometric approach. *Journal of Ethnopharmacology* 151(2):800-809. https:// doi: 10.1016/j.jep.2013.11.004

Naseef H., Al-Maharik N., Rabba A, Sharifi-Rad M., Hawash M., Jaradat N. 2022. Phytochemical characterization and assessments of antimicrobial, cytotoxic and anti-inflammatory properties of *Lavandula coronopifolia* Poir. volatile oil from Palestine. *Arabian Journal of Chemistry* (2022) 15, 104069. doi.org/10.1016/j.arabjc.2022.104069

Neelamkavil, S.V., Thoppil, J.E. (2013). C-mitotic effect of aqueous extracts of *Isodon coetsa* (Buch.-Ham. ex D. Don) Kudo (Lamiaceae). *Nucleus*, 56(2), 117–122. https://doi.org/10.1007/s13237-013-0083-7.

Neelamkavil, S.V., Thoppil, J.E. (2014). Toxicological evaluation of polar and nonpolar components of *Isodon coetsa* (Lamiaceae). *Turkish Journal of Botany*, 38, 252-257. doi: 10.3906/bot-1306-4.

Nguyen, D.T.X., Tran, H., Schwaiger, S., Stuppner, H., Marzocco, S. (2021). Effect of Non-Volatile Constituents of *Elsholtzia ciliata* (Thunb.) Hyl. from Southern Vietnam on Reactive Oxygen Species and Nitric Oxide Release in Macrophages. *Chemistry and Biodiversity*, 18(1), e2000577.

Nicolescu, A., Babotă, M., Ilea, M., Dias, M. I., Calhelha, R. C., Gavrilaș, L., Rocchetti, G., Crișan, G., Mocan, A., Barros, L., & Pârvu, A. E. (2022). Potential therapeutic applications of infusions and hydroalcoholic extracts of Romanian glutinous sage (*Salvia glutinosa* L.). *Frontiers in Pharmacology*, 13. https://doi.org/10.3389/fphar.2022.975800

Nie, L., Li, R., Huang, J., Wang, L., Ma, M., Huang, C., Wu, T., Yan, R., Hu, X. (2021) Abietane diterpenoids from *Dracocephalum moldavica* L. and their anti-inflammatory activities in vitro. *Phytochemistry*. 184:112680. doi: 10.1016/j.phytochem.2021.112680.

Nworu, C. S., & Akah, P. A. (2015). Anti-inflammatory medicinal plants and the molecular mechanisms underlying their activities. *African Journal of Traditional, Complementary and Alternative Medicines*, 12(S1), 52-61. doi:10.4314/ajtcam.v12i5.3S.

Oh TH, Kim SS, Yoon WJ, Kim JY, Yang EJ, Lee NH, Hyun CG. 2009. Chemical composition and biological activities of Jeju *Thymus quinquecostatus* essential oils against *Propionibacterium* species inducing acne. *The Journal of General and Applied Microbiology,* 55 I 1 Pages 63-68. doi.org/10.2323/jgam.55.63

Olajide, O. A., Oladiran, O. O., Awe, S. O., & Makinde, J. M. (1998). Pharmacological evaluation of *Hoslundia opposita* extract in rodents. *Phytotherapy Research*, *12*(5), 364–366. doi.org/10.1002/(SICI)1099-1573(199808)12:5<364::AID-PTR292>3.0.CO;2-N

Olennikov, D.N., Chirikova, N.K., Okhlopkova, Z.M., Zulfugarov, I.S. (2013). Chemical composition and antioxidant activity of *Tánara Ótó* (*Dracocephalum palmatum* Stephan), a medicinal plant used by the North-Yakutian nomads. *Molecules*, 18(11), 14105-14121. doi: 10.3390/molecules181114105.

Oliveira, A. S., Rolo, J., Gaspar, C., Cavaleiro, C., Salgueiro, L., Palmeira-de-Oliveira, R., Ferraz, C., Coelho, S., Pastorinho, M. R., Sousa, A. C., Teixeira, J. P., Martinez-de-Oliveira, J., & Palmeira-de-Oliveira, A. (2022). Chemical characterization and bioactive potential of *Thymus × citriodorus (*Pers.) Schreb. preparations for anti-acne applications: Antimicrobial, anti-biofilm, anti-inflammatory and safety profiles. *Journal of Ethnopharmacology*, *287*. doi.org/10.1016/j.jep.2021.114935

Oliveira, A. S., Rolo, J., Gaspar, C., Ramos, L., Cavaleiro, C., Salgueiro, L., Palmeira-de-Oliveira, R., Teixeira, J. P., Martinez-de-Oliveira, J., & Palmeira-de-Oliveira, A. (2023). *Thymus mastichina* (L.) L. and *Cistus ladanifer* L. for skin application: chemical characterization and in vitro bioactivity assessment. *Journal of Ethnopharmacology*, 302. https://doi.org/10.1016/j.jep.2022.115830

Orfali, R., Siddiqui, N.A., Alam, P., Alhowiriny, T.A., Al-Taweel, A.M., Al-Yahya, S., Majrashi, N.M., Mehmood, R., Khan, S.I., Perveen, S. (2018). Biological evaluation of different extracts of aerial parts of *Nepeta deflersiana* and standardization of active extracts using 8-Epi-7-Deoxyloganic acid and ursolic acid by Validated HPTLC method", *Evidence-Based Complementary and Alternative Medicine*, 2018, 8790769, 11. doi: https://doi.org/10.1155/2018/8790769.

Osmakov, D.I., Koshelev, S.G., Belozerova, O.A., ...Grishin, E.V., Kozlov, S.A. (2015). Biological activity of Sevanol and its analogues. *Russian Journal of Bioorganic Chemistry*, 41(5), pp. 543–547

Osmakov, D.I., Koshelev, S.G., Belozerova, O.A., ...Grishin, E.V., Kozlov, S.A. (2015).The Biological Activity of the Sevanol and Its Analogues. *Bioorganicheskaia khimiia*, 41(5), pp. 606–611

Paksoy, M.Y., Selvi, S., Savran, A. (2016). Ethnopharmacological survey of medicinal plants in UlukIşla (Niğde-Turkey). *Journal of Herbal Medicine*, 6(1), pp. 42–48.

Pandey, A.K., Singh, P., Tripathi, N.N. (2014). Chemistry and bioactivities of essential oils of some *Ocimum* species: An overview. *Asian Pacific Journal of Tropical Biomedicine*, 4(9), pp. 682–694.

Pandur, E., Balatinácz, A., Micalizzi, G., Mondello, L., Horváth, A., Sipos, K., & Horváth, G. (2021). Anti-inflammatory effect of lavender (*Lavandula angustifolia* Mill.) essential oil prepared during different plant phenophases on THP-1 macrophages. *BMC Complementary Medicine and Therapies*, *21*(1). doi.org/10.1186/s12906-021-03461-5

Pandur, E., Micalizzi, G., Mondello, L., Horváth, A., Sipos, K., & Horváth, G. (2022). Antioxidant and Anti-Inflammatory Effects of Thyme (*Thymus vulgaris* L.) Essential Oils Prepared at Different Plant Phenophases on Pseudomonas aeruginosa LPS-Activated THP-1 Macrophages. *Antioxidants*, *11*(7). doi.org/10.3390/antiox11071330

Pargaien, A. V., Bisht, N., Joshi, H., Pargaien, S., & Adhikari, M. (n.d.). Analysis of ethanomedicinally potential extract of *Nepeta cataria*. In Copyright@ EM International (Vol. 26).

Park, S. H., Koo, H. J., Sung, Y. Y., & Kim, H. K. (2013). The protective effect of *Prunella vulgaris* ethanol extract against vascular inflammation in TNF-α-stimulated human aortic smooth muscle cells. *BMB Reports*, *46*(7), 352–357. doi.org/10.5483/BMBRep.2013.46.7.214

Park, S., Kim, S., Suk, K., Lee, H., Kwon, T., Ju, M., Jeon, H., Kim, D., Lim, J., Shin, T. (2010) *Clinopodium gracile* inhibits mast cell-mediated allergic inflammation: involvement of calcium and nuclear factor-kappaB. *Experimental Biological Medicine* 235(5), 606-613. doi: 10.1258/ebm.2010.009292. PMID: 20463301

Parsaei, P., Bahmani, M., Karimi, M., Naghdi, N., Asadi-Samani, M., & Rafieian-Kopaei, M. (2016). A review of analgesic medicinal plants in Iran. *Der Pharmacia Lettre*, 8(2), 43-51. Retrieved from www.scopus.com

Pattanayak, P., Behera, P., Das, D., & Panda, S. (2010). *Ocimum sanctum* Linn. A reservoir plant for therapeutic applications: An overview. In *Pharmacognosy Reviews* 4(7), 95–105. doi.org/10.4103/0973-7847.65323

Pavel, M., Vostinaru, O., Mogosan, C., & Ghibu, S. (2011). Phytochemical and pharmacological research on some extracts obtained from Serpylli herba. *Farmacia*, 59(1), 77–84.

Peana, A. T., & Moretti, M. D. L. (2002). Pharmacological activities and applications of *Salvia sclarea* and *Salvia desoleana* essential oils doi:10.1016/S1572-5995(02)80012-6 Retrieved from www.scopus.com

Peana, A. T., Moretti, M. D. L., & Juliano, C. (1999). Chemical composition and antimicrobial action of the essential oils of *Salvia desoleana* and *S. sclarea*. *Planta Medica*, 65(8), 752-754. doi:10.1055/s-2006-960857

Peana, A., & Satta, M. (1993). A preliminary research on essential oils of *Salvia sclarea* l. and *Salvia desoleana* A. et p. *Pharmacological Research*, 27, 25-26. doi:10.1006/phrs.1993.1048

Petrova, M., Dimitrova, L., Dimitrova, M., ...Lazarova, M., Tasheva, K. (2023). Antitumor and Antioxidant Activities of *In Vitro* Cultivated and Wild-Growing *Clinopodium vulgare* L. Plants. *Plants*, 12(8), 1591

Piccinelli A.C., Figueiredo de Santana Aquino D., Morato P.N., Kuraoka-Oliveira A.M., Strapasson R.L., Dos Santos E.P., Stefanello M.É., Oliveira R.J., Kassuya C.A. 2014. Anti-inflammatory and antihyperalgesic activities of ethanolic extract and fruticulin A from *Salvia lachnostachys* leaves in mice. *Evidence Based Complementary and Alternative Medicine* 2014:835914. https://doi: 10.1155/2014/835914

Piva, R. C., Verdan, M. H., Branquinho, L. S., Kassuya, C. A. L., & Cardoso, C. A. L. (2021). Anti-inflammatory activity and chemical composition of aqueous extract and essential oil from leaves of *Ocimum selloi* Benth. Journal of Ethnopharmacology, 275. https://doi.org/10.1016/j.jep.2021.114136

Prescott, T. A. K., Veitch, N. C., & Simmonds, M. S. J. (2011). Direct inhibition of calcineurin by caffeoyl phenylethanoid glycosides from *Teucrium chamaedrys* and *Nepeta cataria*. *Journal of Ethnopharmacology*, 137(3), 1306–1310. https://doi.org/10.1016/j.jep.2011.07.063

Pudziuvelyte, L., Liaudanskas, M., Jekabsone, A., Sadauskiene, I., (2020). Bernatoniene, J. *Elsholtzia ciliata* (Thunb.) Hyl. extracts from different plant parts: Phenolic composition, antioxidant, and anti-inflammatory activities. *Molecules*, 25(5), 1153.

Qadir MI, Parveen A, Abbas K, Ali M.. 2016. Analgesic, anti-inflammatory and anti-pyretic activities of *Thymus Linearis*. Pak. J. Pharm. Sci., 29, 2, 2016, pp.591-594.

Qi, J., Zhang, Q., Li, L., ...Wang, N., Peng, D. (2022). Spectrum-effect relationship between UPLC-Q-TOF-MS fingerprint and anti-AUB effect of *Clinopodium chinense* (Benth.) O. Kuntze. *Journal of Pharmaceutical and Biomedical Analysis*, 217, 114828. https://doi.org/10.1016/j.jpba.2022.114828.

Raal, A., Paaver, U., Arak, E., & Orav, A. (2004). Content and composition of the essential oil of *Thymus serpyllum* L. growing wild in Estonia. *Medicina (Kaunas)*, *40*(8), 795–800.

Righi, N., Boumerfeg, S., Deghima, A., ...Coimbra, M.A., Baghiani, A. (2021). Phenolic profile, safety assessment, and anti-inflammatory activity of *Salvia verbenaca* L. *Journal of Ethnopharmacology*, 272, 113940.

Rocha, J., Direito, R., Lima, A., Mota, J., Gonçalves, M., Duarte, M. P., Solas, J., Peniche, B. F., Fernandes, A., Pinto, R., Ferreira, R. B., Sepodes, B., & Figueira, M. E. (2019). Reduction of inflammation and colon injury by a Pennyroyal phenolic extract in experimental inflammatory bowel disease in mice. *Biomedicine and Pharmacotherapy*, *118*. doi.org/10.1016/j.biopha.2019.109351

Rodríguez Basso, A., Carranza, A., Zainutti, V. M., Bach, H., & Gorzalczany, S. B. (2021). Pharmacologycal activity of peperina (*Minthostachys verticillata*) on gastrointestinal tract. *Journal of Ethnopharmacology*, 269. https://doi.org/10.1016/j.jep.2020.113712

Rowshan, V., & Najafian, S. (2020). Polyphenolic contents and antioxidant activities of aerial parts of *Salvia multicaulis* from the Iran flora. *Natural Product Research*, 34(16), 2351-2353. doi:10.1080/14786419.2018.1533832

Roxana, S. P., Vivian, M. C., Yazmín, R. C., Daisy, C. Q., Victor, G. C., David, M. D., . . . Lourdes, A. V. (2012). Effects of a hydroalcoholic extract from cuban population of *Salvia coccinea* L. on a model of acute inflammation. *Revista Electronica De Veterinaria*, 13(6).

Roxo, M., Zuzarte, M., Gonçalves, M.J. et al. 2020. Antifungal and anti-inflammatory potential of the endangered aromatic plant *Thymus albicans.* (2020) 10:18859. doi.org/10.1038/s41598-020-75244-w.

Ruiz-Malagón, A. J., Rodríguez-Sanchez, M. J., Rodríguez-Sojo, M. J., Vezza, T., Pischel, I., Algieri, F., Rodríguez-Cabezas, M. E., Rodríguez-Nogales, A., & Gálvez, J. (2022). Intestinal anti-inflammatory and visceral analgesic effects of a *Serpylli herba* extract in an experimental model of irritable bowel syndrome in rats. *Frontiers in Pharmacology*, *13*. doi.org/10.3389/fphar.2022.967644

Ruiz-Malagón, A. J., Rodríguez-Sojo, M. J., Hidalgo-García, L., Molina-Tijeras, J. A., García, F., Pischel, I., Romero, M., Duarte, J., Diez-Echave, P., Rodríguez-Cabezas, M. E., Rodríguez-Nogales, A., & Gálvez, J. (2022b). The Antioxidant Activity of *Thymus serpyllum* Extract Protects against the Inflammatory State and Modulates Gut Dysbiosis in Diet-Induced Obesity in Mice. *Antioxidants*, *11*(6). doi.org/10.3390/antiox11061073

Ruppelt, B.M., Pereira, E.F., Gonçalves, L., & Pereira, N.A. (1991). Pharmacological screening of plants recommended by folk medicine as anti-snake venom-I. Analgesic and anti-inflammatory activities. *Memorias do Instituto Oswaldo Cruz*, 86(2), 203-5.

Ryu, S. Y., Oak, M.-H., Yoon, S.-K., Cho, D.-I., Yoo, G.-S., Kim, T.-S., Kim, K.-M., Thieme, G., Stuttgart, V., & York, N. (2000). Anti-Allergic and Anti-Inflammatory Triterpenes from the Herb of *Prunella vulgaris*. *Planta Medica*, *66*, 358.

Sahrial I, Solfaine R (2019). *Coleus amboinicus* extract increases transforming growth factor-1β expression in Wistar rats with cisplatin-induced nephropathy, *Veterinary World*, 12(8): 1346-1351. doi: 10.14202/vetworld.2019.1346-1351.

Salehi, B., Mishra, A. P., Shukla, I., Sharifi-Rad, M., Contreras, M. del M., Segura-Carretero, A., Fathi, H., Nasrabadi, N. N., Kobarfard, F., & Sharifi-Rad, J. (2018). Thymol, thyme, and other plant sources: Health and potential uses. In *Phytotherapy Research* (Vol. 32, Issue 9, pp. 1688–1706). John Wiley and Sons Ltd. doi.org/10.1002/ptr.6109

Santana, F. R., de Santana Souza, M. T., Camargo, E. A., & Silva, J. A. da. (2022). Anti-inflammatory and antinociceptive effects of a pectinolide-enriched fraction from *Mesosphaerum pectinatum* (L.) Kuntze. *Journal of Ethnopharmacology*, 115916. doi.org/10.1016/j.jep.2022.115916

Saoulajan, C., Boujida, N., El Mihyaoui, A., El Baakili, A., Alshahrani, M. M., Lee, L. -., & Bouyahya, A. (2022). Phytochemistry, pharmacological investigations, industrial applications, and encapsulation of *Thymbra capitata* L., a review. *Trends in Food Science and Technology*, 129, 463-491. doi:10.1016/j.tifs.2022.10.014.

Sarikurkcu, C., Sabih Ozer, M., Eskici, M., Tepe, B., Can, S., Mete, E., 2010. Essential oil composition and antioxidant activity of *Thymus longicaulis* C. Presl subsp. longicaulis var. longicaulis. *Food Chem. Toxicol.* 48, 1801–1805.

Semaoui, R., Ouafi, S., Machado, S., Barros, L., Ferreira, I. C. F. R., & Oliveira, M. B. P. P. (2021). Infusion of aerial parts of *Salvia chudaei* Batt. & Trab. from Algeria: Chemical, toxicological and bioactivities characterization. *Journal of Ethnopharmacology*, *280*. doi.org/10.1016/j.jep.2021.114455

Shabbir, A., Batool, S. A., Basheer, M. I., Shahzad, M., Sultana, K., Tareen, R. B., Iqbal, J., & Saeed-ul-Hassan. (2018). *Ziziphora clinopodioides* ameliorated rheumatoid arthritis and inflammatory paw edema in different models of acute and chronic inflammation. *Biomedicine and Pharmacotherapy*, *97*, 1710–1721. doi.org/10.1016/j.biopha.2017.11.118

Shaikh, R., Pund, M., Dawane, A., Iliyas, S. (2014). Evaluation of anticancer, antioxidant, and possible anti-inflammatory properties of selected medicinal plants used in Indian traditional medication. *Journal of Traditional and Complementary Medicine* 4(4), 253-257.

Shan, M., Jiang, Y., Fu, Y., ...Zhang, L., Wu, Q. (2021). A review of the botany, traditional uses, phytochemistry and pharmacology of *Nepeta tenuifolia* Briq. *Phytochemistry Reviews*, 20(5), pp. 991–1012

Shanaida, M., Hudz, N., Jasicka-Misiak, I., & Wieczorek, P. P. (2021). Polyphenols and Pharmacological Screening of a *Monarda fistulosa* L. dry Extract Based on a Hydrodistilled Residue By-Product. *Frontiers in Pharmacology*, 12. https://doi.org/10.3389/fphar.2021.563436

Shanaida, M., Jasicka-Misiak, I., Bialon, M., Korablova, O., & Wieczorek, P. P. (2021). Chromatographic profiles of the main secondary metabolites in the *Monarda fistulosa* l. Aerial part. *Research Journal of Pharmacy and Technology*, 14(4), 2179–2184. https://doi.org/10.52711/0974-360X.2021.00385

Shanmugam, G., Sundaramoorthy, A., & Shanmugam, N. (2021). Biosynthesis of silver nanoparticles from leaf extract of *Salvia coccinea* and its effects of anti-inflammatory potential in human monocytic THP-1 cells. *ACS Applied Bio Materials*, 4(12), 8433-8442. doi:10.1021/acsabm.1c00963

Sharifabad, A. H., Sadraei, H., Hashemnia, M., Sajjadi, S. E., & Mirdamadi, Z. (2021). Effect of hydroalcoholic and aqueous extracts of *Dracocephalum kotschyi* on bleomycin induced pulmonary fibrosis. *Journal of HerbMed Pharmacology*, 10(2), 209–217. https://doi.org/10.34172/jhp.2021.23

Sharifi-Rad, M., Berkay Yılmaz, Y., Antika, G., Salehi, B., Tumer, T. B., Kulandaisamy Venil, C., Das, G., Patra, J. K., Karazhan, N., Akram, M., Iqbal, M., Imran, M., Sen, S., Acharya, K., Dey, A., & Sharifi-Rad, J. (2021). Phytochemical constituents, biological activities, and health-promoting effects of the genus *Origanum*. In *Phytotherapy Research* (Vol. 35, Issue 1, pp. 95–121). John Wiley and Sons Ltd. doi.org/10.1002/ptr.6785

Shehadeh, M. B., Sosa, S., Suaifan, Darwish, R. M., Giangaspero, A., Vassallo, A., Lepore, L., Oran, S., Hammad, H., Tubaro, A., De Tommasi, N., Loggia, R. D. (2014). Topical anti-inflammatory potential of six *Salvia* species grown in Jordan. *Jordan Journal of Pharmaceutical Sciences*, 7(2), 153-161. doi:10.12816/0026806

Shen, W., Anwaier, G., Cao, Y., Lian, G., Chen, C., Liu, S., Tuerdi, N., & Qi, R. (2019). Atheroprotective Mechanisms of Tilianin by Inhibiting Inflammation Through Down-Regulating NF-κB Pathway and Foam Cells Formation. *Frontiers in Physiology*, *10*. doi.org/10.3389/fphys.2019.00825

Sheychenko, O. P., Sheychenko, V. I., Goryainov, S. v., Zvezdina, E. v., Kurmanova, E. N., Ferubko, E. v., Uyutova, E. v., Potanina, O. G., & Fadi, K. (2021). Chemical composition and biological activity of the dry extract "Rosmatin" from the herb of *Dracocephalum moldavica* L. *Khimiya Rastitel'nogo Syr'ya*, *3*, 253–264. doi.org/10.14258/JCPRM.2021039161

Shi, Q.Q., Dang, J., Wen, H.X., Yuan, X., Tao, Y.D., Wang, Q.L. (2016). Anti-hepatitis, antioxidant activities and bioactive compounds of *Dracocephalum heterophyllum* extracts. *Botanical Studies*, 57(1):16. doi: 10.1186/s40529-016-0133-y.

Shimizu, M., Shogawa, H., Matsuzawa, T., ...Suzuki, S., Berganza, A. (1990). Anti-inflammatory Constituents of Topically Applied Crude Drugs: IV: Constituents And Anti-inflammatory Effect of Paraguayan Crude Drug "Alhucema" (*Lavandula latifolia* Vill.). *Chemical and Pharmaceutical Bulletin*, 38(8), pp. 2283–2284

Shin, T. Y., Kim, S. H., Choi, C. H., Shin, H. Y., & Kim, H. M. (2004). *Isodon japonicus* decreases immediate-type allergic reaction and tumor necrosis factor-α production. *International Archives of Allergy and Immunology*, 135(1), 17–23. https://doi.org/10.1159/000080038

Shin, T. Y., Kim, S. H., Suk, K., Ha, J. H., Kim, I. K., Lee, M. G., Jun, C. D., Kim, S. Y., Lim, J. P., Eun, J. S., Shin, H. Y., & Kim, H. M. (2005). Anti-allergic effects of *Lycopus lucidus* on mast cell-mediated allergy model. *Toxicology and Applied Pharmacology*, *209*(3), 255–262. doi.org/10.1016/j.taap.2005.04.011

Shipa, S.J., Khandokar, L., Bari, M.S., ...Haque, M.A., Mohamed, I.N. (2022). An insight into the anti-ulcerogenic potentials of medicinal herbs and their bioactive metabolites. *Journal of Ethnopharmacology*, 293, 115245.

Singh, D., & Chaudhuri, P. K. (2018). A review on phytochemical and pharmacological properties of Holy basil (*Ocimum sanctum* L.). In *Industrial Crops and Products* (Vol. 118, pp. 367–382). Elsevier B.V. doi.org/10.1016/j.indcrop.2018.03.048

Singh, M. K., Gidwani, B., Gupta, A., Dhongade, H., Kaur, C. D., Kashyap, P. P., & Tripathi, D. K. (2015). A review of the medicinal plants of genus *Orthosiphon* (Lamiaceae). *International Journal of Biological Chemistry*, *9*(6), 318–331. doi.org/10.3923/ijbc.2015.318.331

Sitarek, P., Kowalczyk, T., Synowiec, E., Merecz-Sadowska, A. Bangay, G., Princiotto, S. Sliwinski, T.; Rijo, P. 2022. An evaluation of the novel biological properties of diterpenes isolated from *Plectranthus ornatus* Codd. *In vitro* and in silico. Cells 11, 3243. https://doi.org/10.3390/cells11203243.

Slighoua, M., Mahdi, I., ez-zahra Amrati, F., Boucetta, N., Cristo, F. di, Boukhira, S., el youbi el Hamsas, A., Tattou, M. I., Grafov, A., Bari, A., & Bousta, D. (2022). Pharmacological effects of *Lavandula officinalis* Chaix and its polyphenols: Focus on their in vivo estrogenic and anti-inflammatory properties. *South African Journal of Botany*, *146*, 354–364. doi.org/10.1016/j.sajb.2021.11.014

Solfaine, R., Muniroh, L., Sadarman, Apriza, & Irawan, A. (2021). Anti-inflammatory effect of *Coleus amboinicus* leaves extract on uric acid-induced nephrotoxicity rats. *Advances in Animal and Veterinary Sciences*, 9(10), 1553-1558. doi:10.17582/journal.aavs/2021/9.10.1553.1558.

Sosa S, Altinier G, Politi M, Braca A, Morelli I, Della Loggia R. 2005. Extracts and constituents of *Lavandula multifida* with topical anti-inflammatory activity. *Phytomedicine* 12 (2005) 271–277. doi:10.1016/j.phymed.2004.02.007.

Souri, F., Rakhshan, K., Erfani, S., Azizi, Y., Maleki, S., & Aboutaleb, N. (2019). Natural lavender oil (*Lavandula angustifolia*) exerts cardioprotective effects agasint myocardial infarction by targeting inflammation and oxidative stress. *Inflammopharmacology*, 27(4), 799–807.

Sousa PJ, Linard CF, Azevedo-Batista D, Oliveira AC, Coelho-de-Souza AN, Leal-Cardoso JH.. 2009. Antinociceptive effects of the essential oil of *Mentha x villosa* leaf and its major constituent piperitenone oxide in mice. *Brazilian Journal of Medical and Biological Research* (2009) 42: 655-659.

Staliñska, K., Guzdek, A., Rokicki, M., & Koj, A. (n.d.). Transcription factors as targets of the anti-inflammatory treatment. A cell culture study with extracts from some mediterranean diet plants. www.jpp.krakow.pl

Sudaramoorthy, A., Shanmugam, G., & Shanmugam, N. (2021). Inhibitory effect of *Salvia coccinea* on inflammatory responses through NF-κB signaling pathways in THP-1 cells and acute rat diabetes mellitus. *Acta Histochemica*, 123(5) doi:10.1016/j.acthis.2021.151735

Sun J., Sun P., Kang C., Zhang L., Guo L., Kou Y. 2022. Chemical composition and biological activities of essential oils from six Lamiaceae folk medicinal plants. *Frontiers in Plant* Science 13:919294. https://doi: 10.3389/fpls.2022.919294.

Sun, Z., Wang, H., Wang, J., Zhou, L., & Yang, P. (2014). Chemical composition and anti-inflammatory, cytotoxic and antioxidant activities of essential oil from leaves of *Mentha piperita* grown in China. *PLoS ONE*, *9*(12). doi.org/10.1371/journal.pone.0114767

Süntar, I., Nabavi, S.M., Barreca, D., Fischer, N., Efferth, T. (2018). Pharmacological and chemical features of *Nepeta* L. genus: Its importance as a therapeutic agent. *Phytotherapy Research*, 32, 185–198. doi: 10.1002/ptr.5946.

Taarit, M.B., Msaada, K., Hosni, K., Chahed, T., Marzouk, B. (2010). Essential oil composition of *Salvia verbenaca* L. growing wild in Tunisia. *Journal of Food Biochemistry*, 34(1), pp. 142–151.

Tadesse, S., Mazumder, A., Bucar, F., Veeresham, C., & Asres, K. (2011). Chemical composition and biological activities of the essential oil of *Plectranthus caninus* Roth. *Pharmacognosy Journal*, *3*(26), 1–7. doi.org/10.5530/pj.2011.26.1

Taheri, S., Khalifeh, S., Shajiee, H., & Ashabi, G. (2021). Dietary uptake of *Salvia macilenta* extract improves Nrf2 antioxidant signaling pathway and diminishes inflammation and apoptosis in amyloid beta-induced rats. *Molecular Biology Reports*, *48*(12), 7667–7676. doi.org/10.1007/s11033-021-06772-5.

Tang, Y. Q. N., Deng, J., Li, L., Yan, J., Lin, L. M., Li, Y. M., Lin, Y., & Xia, B. H. (2022). Essential oil from *Prunella vulgaris* L. as a valuable source of bioactive constituents: In vitro anti-bacterial, anti-viral, immunoregulatory, anti-inflammatory, and chemical profiles. *South African Journal of Botany*, *151*, 614–627. doi.org/10.1016/j.sajb.2022.10.029

Taşkın T, Emin Çam M, Bulut G, Nur Hazar-Yavuz A, Kabasakal L, Bitiş L. (2018) Antioxidant and anti-inflammatory activities of *Phlomis pungens* and *Coridothymus capitatus*. *Marmara Pharmaceutical Journal*: 22(1), 80-85. dx.doi.org/10.12991/mpj.2018.44

Taşkın, T., Çam, M.E., Taşkın, D. et al. 2019. In vitro and In vivo biological activities and phenolic characterization of *Thymus praecox* subsp. skorpilii var. skorpilii. *Journal of Food Measurement and Characterization* (2019) 13:536–544 https://doi.org/10.1007/s11694-018-9967-1

Taskin, T., Dogan, M., Cam, M. E., Sahin, T., & Senkardes, I. (2020). In vitro anti-urease, antioxidant, anticholinesterase, cytotoxic and in vivo anti-inflammatory potential of *Satureja cuneifolia* Ten. *Notulae Scientia Biologicae*, 12(2), 222–232. doi.org/10.15835/nsb12210687

Tepe, B., Cakir, A., & Sihoglu Tepe, A. (2016). Medicinal Uses, Phytochemistry, and Pharmacology of *Origanum onites* (L.): A Review. *Chemistry and Biodiversity* (Vol. 13, Issue 5, pp. 504–520). Wiley-VCH Verlag. https://doi.org/10.1002/cbdv.201500069

Thawkar, B., Jawarkar, A. G., Kalamkar, P., Pawar, K., Kale, M. K. (2016). Phytochemical and pharmacological review of *Mentha arvensis*. *International Journal of Green Pharmacy*, *10*(2), 71–76. www.researchgate.net/publication/316256954

Tsioutsiou, E. E., Amountzias, V., Vontzalidou, A., Dina, E., Stevanović, Z. D., Cheilari, A., & Aligiannis, N. (2022). Medicinal plants used traditionally for skin related problems in the south balkan and east mediterranean Region—A review. *Frontiers in Pharmacology*, 13 doi:10.3389/fphar.2022.936047

Tubon I, Zannoni A, Bernardini C, Salaroli R, Bertocchi M, Mandrioli R, Vinueza D, Antognoni F, Forni M. 2019. *In vitro* anti-inflammatory effect of *Salvia sagittata* ethanolic extract on primary cultures of porcine aortic endothelial cells. *Oxidative Medicine and Cellular Longevity* 2019:6829173. https://doi: 10.1155/2019/6829173

Ueda, H., Yamazaki, C., & Yamazaki, M. (2002). Luteolin as an anti-inflamamtory and anti-allergic constituent of *Perilla frutescens*. *Biological Pharmaceutical Bulletin*, *25*(9), 1197–1202.

Ulikhanyan, G., Galstyan, H., Dumanyan, K., Beglaryan, M., Poghosyan, G., Shekoyan, V., Ananikyan, H., Ananikyan, V., Goginyan, V., Ulikhanyan, G., Chichoyan, N., & Taschyan, S. (2022). The study of the biological activities of *Ziziphora clinopodioides*. *Brazilian Journal of Pharmaceutical Sciences*, *58*. doi.org/10.1590/s2175-97902022e19331

Ustuner, O., Anlas, C., Bakirel, T., ...Donmez, C., Koca-Caliskan, U. (2019). *In vitro* evaluation of antioxidant, anti-inflammatory, antimicrobial and wound healing potential of *Thymus sipyleus* Boiss. subsp. *rosulans* (Borbas) Jalas. *Molecules*, 24(18), 3353.

Vairinhos, J., Miguel, M.G. (2020). Essential oils of spontaneous species of the genus *Lavandula* from Portugal: A brief review. *Zeitschrift fur Naturforschung - Section C Journal of Biosciences*, 75(78), pp. 233–245.

Vazirian, F., Samadi, S., Abbaspour, M., ...Mohammadpour, A.H., Emami, S.A. (2022). Evaluation of the efficacy of *Thymus kotschyanus* extract as an additive treatment in patients with ulcerative colitis: a randomized double-blind placebo-controlled trial. *Inflammopharmacology*, 30(6), pp. 2145–2152

Verano, J., González-Trujano, M. E., Déciga-Campos, M., Ventura-Martínez, R., & Pellicer, F. (2013). Ursolic acid from *Agastache mexicana* aerial parts produces antinociceptive activity involving TRPV1 receptors, cGMP and a serotonergic synergism. *Pharmacology Biochemistry and Behavior*, 110, 255–264. https://doi.org/10.1016/j.pbb.2013.07.020

Vijayan, C., Murugan, K., Reji, S., & Nair, G. (2017). Screening biological activities of *Orthoshiphon aristatus*: some observations. *International Journal of Advanced Research*, *5*(12), 757–764. doi.org/10.21474/IJAR01/6016

Villaseñor, I. M., Angelada, J., Canlas, A. P., & Echegoyen, D. (2002). Bioactivity studies on β-sitosterol and its glucoside. *Phytotherapy Research*, *16*(5), 417–421. doi.org/10.1002/ptr.910

Vladimir-Knežević, S., Cvijanović, O., Blažeković, B., Kindl, M., Štefan, M. B., & Domitrović, R. (2015). Hepatoprotective effects of *Micromeria croatica* ethanolic extract against CCl4-induced liver injury in mice. *BMC Complementary and Alternative Medicine*, *15*(1). doi.org/10.1186/s12906-015-0763-8

Vogl, S., Picker, P., Mihaly-Bison, J., Fakhrudin, N., Atanasov, A. G., Heiss, E. H., Wawrosch, C., Reznicek, G., Dirsch, V. M., Saukel, J., & Kopp, B. (2013). Ethnopharmacological in vitro studies on Austria's folk medicine - An unexplored lore *in vitro* anti-inflammatory activities of 71 Austrian traditional herbal drugs. *Journal of Ethnopharmacology*, *149*(3), 750–771. doi.org/10.1016/j.jep.2013.06.007

Vulganová, K., Maliar, T., Maliarová, M., Nemeček, P., Viskupičová, J., BaláŽová, A., & Sokol, J. (2019). Biologically valuable components, antioxidant activity and proteinase inhibition activity of leaf and callus extracts of *Salvia* sp. *Nova Biotechnologica et Chimica*, 18(1), 25-36. doi:10.2478/nbec-2019-0004

Wan, J., Liu, M., Jiang, H. Y., Yang, J., Du, X., Li, X. N., Wang, W. G., Li, Y., Pu, J. X., & Sun, H. D. (2016). Bioactive ent-kaurane diterpenoids from *Isodon serra*. *Phytochemistry*, 130, 244–251. doi.org/10.1016/j.phytochem.2016.05.014

Wang, M., Dai, H., Li, X., ...Wang, L., Xue, M. (2010). Structural elucidation of metabolites of tanshinone I and its analogue dihydrotanshinone I in rats by HPLC-ESI-MSn. *Journal of Chromatography B: Analytical Technologies in the Biomedical and Life Sciences*, 878(13-14), pp. 915–924.

Wang, M., Wang, Y., Shi, L., Lu, Y., Zhu, H., Liang, L., & Sun, Z. L. (2022). A New Phenylpropanoid-Substituted Flavan-3-ol from Aerial Part of *Mentha longifolia*. *Chemistry of Natural Compounds*, *58*(2), 237–239. doi.org/10.1007/s10600-022-03649-2

Wang, X., Cheng, K., Liu, Z., Sun, Y., Zhou. L., Xu. M., Dai, X., Xiong, Y., Zhang, H. (2021). Bioactive constituents of *Mosla chinensis*-cv. Jiangxiangru ameliorate inflammation through MAPK signaling pathways and modify intestinal microbiota in DSS-induced colitis mice. *Phytomedicine* 93,153804. doi: 10.1016/j.phymed.2021.153804.

Wang, Y. Y., Lin, S. Y., Chen, W. Y., Liao, S. L., Wu, C. C., Pan, P. H., Chou, S. T., & Chen, C. J. (2017). *Glechoma hederacea* extracts attenuate cholestatic liver injury in a bile duct-ligated rat model. *Journal of Ethnopharmacology*, *204*, 58–66. doi.org/10.1016/j.jep.2017.04.011

Wang, Y., Shao, Z., Song, C., ...Zhou, G., Meng, D. (2023). *Clinopodium chinense* Kuntze ameliorates dextran sulfate sodium-induced ulcerative colitis in mice by reducing systematic inflammation and regulating metabolism. *Journal of Ethnopharmacology*, 309, 116330.

Wang, Z., Hwang, S.H., Guillen Quispe, Y.N., Gonzales Arce, P.H., Lim, S.S. (2017). Investigation of the antioxidant and aldose reductase inhibitory activities of extracts from Peruvian tea plant infusions. *Food Chemistry*, 231, 222–230. http://dx.doi.org/10.1016/j.foodchem.2017.03.107.

Wu, H.-Y., Wang, W.-G., Du, X., Yang, J., Pu, J.-X., Sun, H.-D. (2015). Six new cytotoxic and anti-inflammatory 11, 20-epoxy-ent-kaurane diterpenoids from *Isodon wikstroemioides*. *Chinese Journal of Natural Medicines*, 13(5), 383–389.

Wu, H.-Y., Wang, W.-G., Jiang, H.-Y., Du, X., Li, X.-N., Pu, J.-X., Sun, H.-D. (2014). Cytotoxic and anti-inflammatory ent-kaurane diterpenoids from *Isodon wikstroemioides*. *Fitoterapia*, 98, 192–198. http://dx.doi.org/10.1016/j.fitote.2014.08.012.

Wu, S.J., Chan, H.H., Hwang, T.L., Qian, K., Morris-Natschke, S., Lee, K.H., Tian-Shung Wu, T.S. 2010. Salviatalin A and salvitrijudin A, two diterpenes with novel skeletons from roots of *Salvia digitaloides* and anti-inflammatory evaluation. Tetrahedron Letters 51(32):4287-4290. DOI: https://10.1016/j.tetlet.2010.06.048.

Wu, Y., Wang, Y., Liu, X., Jiang, L., Guli, A., Sailike, J., Sun, X., Abuduwaili, N., Tuoliuhan, H., Maney, K., & Nabi, X. (2020). *Ziziphora clinopodioides* flavonoids based on network pharmacology attenuates atherosclerosis in rats induced by high-fat emulsion combined with vitamin D3 by down-regulating VEGF/AKT/NF-κB signaling pathway. *Biomedicine and Pharmacotherapy*, 129. doi.org/10.1016/j.biopha.2020.110399.

Xia F, Wu CY, Yang XW, Li X, Xu G. 2015. Diterpenoids from the Roots of *Salvia yunnanensis*. *Nat. Prod. Bioprospect.* (2015) 5:307–312. doi.10.1007/s13659-015-0080-4.

Xiang, Z., Wu, X., Liu, X., Jin, Y. (2014). Glaucocalyxin A: a review. *Natural Product Research*, 28:24, 2221-2236. https://doi.org/10.1080/14786419.2014.934235.

Xie, Q., Wang, Y., & Zou, G. L. (2022). Protective effects of lavender oil on sepsis-induced acute lung injury via regulation of the NF-κB pathway. *Pharmaceutical Biology*, *60*(1), 968–978. doi.org/10.1080/13880209.2022.2067570

Xing, H., An, L., Song, Z., Li, S., Wang, H., Wang, C., Zhang, J., Tuerhong, M., Abudukeremu, M., Li, D., Lee, D., Xu, J., Lall, N., & Guo, Y. (2020). Anti-Inflammatory ent-Kaurane Diterpenoids from *Isodon serra*. *Journal of Natural Products*, 83(10), 2844–2853. https://doi.org/10.1021/acs.jnatprod.9b01281

Xing, J., Peng, K., Cao, W., Lian, X., Wang, Q., & Wang, X. (2013). Effects of total flavonoids from *Dracocephalum moldavica* on the proliferation, migration, and adhesion molecule expression of rat vascular smooth muscle cells induced by TNF-*α*. *Pharmaceutical Biology*, *51*(1), 74–83. doi.org/10.3109/13880209.2012.711839.

Xu G, Peng LY, Lu L, Weng ZY, Zhao Y, Li XL, Zhao QS, Sun HD. 2005. Two New Abietane Diterpenoids from *Salvia yunnanensis*. *Planta Med* 2006; 72: 84±86. DOI 10.1055/s-2005-873184.

Yan, D. (2016). Substitutability of *Prunella vulgaris* L. stem, leaf and ear based on HPLC-ESI-MS analysis and anti-inflammatory and antioxidant activity. *Chinese Pharmaceutical Journal*, *24*, 792–797. pesquisa.bvsalud.org/portal/resource/pt/wpr-859105

Yang, E.-L., Hou, Y., Ma, G.-X., Zou, L.-J., Xu, X.-D., Wu, H.-F., Yang, J.-S., Wei, H.-W., Fan, C.-Z., Sun, Z.-C. Shi, L.-L. (2022). Abietane-Type diterpenoids from *Nepeta bracteata* Benth. and their anti-inflammatory activity. *Frontiers in Chemistry*, 10, 944972. doi: 10.3389/fchem.2022.944972.

Yang, F., Pu, H.-Y., Yaseen, A., Chen, B., Li, F. Gu, Y.-Ch., Shen, X.-F., Wang, M.-K. Guo, D.-L., Wang, L. (2021). Terpenoid and phenolic derivatives from the aerial parts of *Elsholtzia rugulosa* and their anti-inflammatory activity. *Phytochemistry*, 181: 112543. https://doi.org/10.1016/j.phytochem.2020.112543.

Yang, N., Zou, C., Luo, W., Xu, D., Wang, M., Wang, Y., Wu, G., Shan, P., & Liang, G. (2022). Sclareol attenuates angiotensin II -induced cardiac remodeling and inflammation via inhibiting MAPK signaling. *Phytotherapy Research*, 1-14 doi.org/10.1002/ptr.7635

Yang, Z., Qi, J., Ping, D., Sun, X., Tao, Y., Liu, C., & Peng, Y. (2022). *Salvia miltiorrhiza* in thorax and abdomainal organ fibrosis: A review of its pharmacology. *Frontiers in Pharmacology* (Vol. 13). Frontiers Media S.A. doi.org/10.3389/fphar.2022.999604.

Yazbek, P.B., Tezoto L., Cassas F., Rodrigues E.. 2016. Review Plants used during maternity, menstrual cycle and other women's health conditions among Brazilian cultures. Journal of Ethnopharmacology 179 (2016) 310–331. dx.doi.org/10.1016/j.jep.2015.12.054

Yi, W., Chen, R., Xie, F., Xu, C., & Tian, W. (2022). Anti-inflammatory and immunomodulatory properties of *Mentha piperita* green-formulated gold nanoparticles and its effect on ovalbumin-induced asthma and lung pathological changes in rats. *Journal of Experimental Nanoscience*, *17*(1), 163–172. doi.org/10.1080/17458080.2022.2033730

Yin, Q.-Q., Liu, C.-X., Wu, Y.-L., Wu, S.-F., Wang, Y., Zhang, X., Hu, X.-J., Pu, J.-X., Lu, Y., Zhou, H.-C., Wang, H.-L., Nie, H., Sun, H.-D., & Chen, G.-Q. (2013). Preventive and Therapeutic Effects of Adenanthin on Experimental Autoimmune Encephalomyelitis by Inhibiting NF-κB Signaling. *The Journal of Immunology*, *191*(5), 2115–2125. doi.org/10.4049/jimmunol.1203546

Yu, Y., Xing, N., Xu, X., ...Sun, G., Sun, X. (2019). Tournefolic acid B, derived from *Clinopodium chinense* (Benth.) Kuntze, protects against myocardial ischemia/reperfusion injury by inhibiting endoplasmic reticulum stress-regulated apoptosis via PI3K/AKT pathways. *Phytomedicine*, 52, pp. 178–186.

Yu, Z.-Y., Liang, Y.-G., Xiao, H., Shan, Y.-J., Dong, B., Huang, R., Fu, Y.-L., Zhao, Z.-H., Liu, Z.-Y., Zhao, Q.-S., Wang, S.-Q., Chen, J.-P., Mao, B.-Z., Cong, Y.-W. (2007). Melissoidesin G, a diterpenoid purified from *Isodon melissoides*, induces leukemic-cell apoptosis through induction of redox imbalance and exhibits synergy with other anticancer agents. *International Journal of Cancer*, 121, 2084–2094. doi: 10.1002/ijc.22945.

Yuan, F., Liu, R., Hu, M., Sun, Y., He, J. JAX2, an ethanol extract of *Hyssopus cuspidatus* Boriss., can prevent bronchial asthma by inhibiting MAPK/NF-κB inflammatory signaling. *Phytomedicine*, 57, pp. 305–314.

Zam, W., Quispe, C., Sharifi-Rad, J., López, M. D., Schoebitz, M., Martorell, M., Sharopov, F., Fokou, P. V. T., Mishra, A. P., Chandran, D., Kumar, M., Chen, J. T., & Pezzani, R. (2022). An Updated Review on The Properties of *Melissa officinalis* L.: Not Exclusively Anti-anxiety. In *Frontiers in bioscience* 14(2). doi.org/10.31083/j.fbs1402016

Zargar, O. A., Bashir, R., Ganie, S. A., & Hamid, R. (2019). The effect of *Elsholtzia densa* methanolic extract modulates inflammation in vitro and in vivo. *Oriental Pharmacy and Experimental Medicine*, *19*(1), 49–58. doi.org/10.1007/s13596-018-0346-6.

Zarshenas, M. M., & Krenn, L. (2015). Phytochemical and pharmacological aspects of *Salvia mirzayanii* Rech. f. & Esfand. *Journal of Evidence-Based Complementary and Alternative Medicine*, 20(1), 65–72. https://doi.org/10.1177/2156587214553938

Zengin, G., Ferrante, C., Orlando, G., Zheleva-Dimitrova, D., Gevrenova, R., Recinella, L., Chiavaroli, A., Leone, S., Brunetti, L., Aumeeruddy, M.Z., Aktumsek, A., Mahomoodally, M.F., Angelini, P., Covino, S., Venanzoni, R., Tirillini, B., Menghini, L. (2019). Chemical profiling and pharmaco-toxicological activity of *Origanum sipyleum* extracts: Exploring for novel sources for potential therapeutic agents. *Journal of Food Biochemistry*, 43, e13003. doi: https://doi.org/10.1111/jfbc.13003.

Zhang, J., Yan, X., Xu, S., Wang, D., Liu, H. (2021) High-performance thin-layer chromatographic quantification of four active compounds in total flavonoids of *Ziziphora clinopodioides* Lam. and TLC–DPPH test for screening antioxidant components. *JPC-J Planar Chromat* 34, 481–492. doi.org/10.1007/s00764-021-00147-z

Zhang, M., Chen, M., Hou, Y., Fan, C., Wei, H., Shi, L., Ma, G., Zhang, J. (2021). Inflammatory and cytotoxic activities of Abietane terpenoids from *Nepeta bracteata* Benth.. *Molecules*, 26, 5603. https://doi.org/10.3390/molecules26185603.

Zhang, M., Hwang, E., Lin, P., Gao, W., Ngo, H. T. T., & Yi, T. H. (2018b). *Prunella vulgaris* L. Exerts a Protective Effect Against Extrinsic Aging Through NF-κB, MAPKs, AP-1, and TGF-β/Smad Signaling Pathways in UVB-Aged Normal Human Dermal Fibroblasts. *Rejuvenation Research*, *21*(4), 313–322. doi.org/10.1089/rej.2017.1971

Zhang, Q., Porto, N.M., Guilhon, C.C., ...Fernandes, P.D., Boylan, F. (2021). Pharmacognostic study on *Elsholtzia ciliata* (Thumb.) Hyl.: Anatomy, phytochemistry and pharmacological activities. *Pharmaceuticals*, 14(11), 1152.

Zhang, Y. Y., Jiang, H. Y., Liu, M., Hu, K., Wang, W. G., Du, X., Li, X. N., Pu, J. X., & Sun, H. D. (2017). Bioactive ent-kaurane diterpenoids from *Isodon rubescens*. *Phytochemistry*, *143*, 199–207. doi.org/10.1016/j.phytochem.2017.08.009

Zhang, Y., Liu, J., Jia, W., Zhao, A., Zhang, T. L., Liu, Y., Jia, J., Zhao, W., & Li, A. (2005). Distinct immunosuppressive effect by *Isodon serra* extracts. *International Immunopharmacology* (Vol. 5). http://www.sciencedirect.com/science/article/pii/S1567576905001803

Zhang, Y., Wang, K., Chen, H., He, R., Cai, R., Li, J., Zhou, D., Liu, W., Huang, X., Yang, R., Deng, S., Li, J., & Guan, X. (2018). Anti-inflammatory lignans and phenylethanoid glycosides from the root of *Isodon ternifolius* (D.Don) Kudô. *Phytochemistry*, *153*, 36–47. doi.org/10.1016/j.phytochem.2018.05.017

Zhao, J., Xu, F., He, J.H., Gu, Z.Y., Ma, L. (2013). [Study on chemical constituents of *Hyssopus cuspidatus*. Zhong yao cai = Zhongyaocai = *Journal of Chinese medicinal materials*, 36(1), pp. 54–57.

Zhao, J., Xu, F., Huang, H., Ji, T., Li, C., Tan, W., Chen, Y., Affiliations, L. M., & Pharm Sci, P. J. (2015). Evaluation on bioactivities of total flavonoids from *Lavandula angustifolia*. *Pakistan Journal of Pharmaceutical Sciences*, *28*(4), 1245–1251. pubmed-ncbi-nlm-nih-gov.pbidi.unam.mx:2443/26142514/

Zhao, J.; Xu, L.; Jin, D.; Xin, Y.; Tian, L.;Wang, T.; Zhao, D.; Wang, Z.;Wang, J. 2022. Rosmarinic acid and related dietary supplements: potential applications in the prevention and treatment of Cancer. *Biomolecules* 12, 1410. https://doi.org/10.3390/biom12101410

Zheng, X. Q., Song, L. X., Qiu, H., Yang, Y. B., Han, Z. Z., Wang, Z. T., & Gu, L. H. (2022). Novel phenolic and diterpenoid compounds isolated from the fruit spikes of *Prunella vulgaris* L. and their anti-inflammatory activities. *Phytochemistry Letters*, *49*, 60–64. doi.org/10.1016/j.phytol.2022.03.005

Zhong, J., Muhammad, N., Yang, X. Li, J. (2022). Antimicrobial, antioxidant, anti-inflammatory activities of eight essential oils obtained from traditional chinese medicines using supercritical fluid extraction coupled molecular distillation. *Journal of Essential Oil-Bear Plants* 25 (5) 1145 – 1158.

Zhong, J., Muhammad, N., Yang, X., Li, J. (2022). Antimicrobial, antioxidant, anti-inflammatory activities of eight essential oils obtained from traditional Chinese medicines using supercritical fluid extraction coupled molecular distillation. *Journal of Essential Oil Bearing Plants*, 25:5, 1145-1158, doi: 10.1080/0972060X.2022.2142484.

Zhong, Y., Huang, Y., Santoso, M. B., & Wu, L.D. (2015). Sclareol exerts anti-osteoarthritic activities in interleukin-1β-induced rabbit chondrocytes and a rabbit osteoarthritis model. *International Journal of Clinical and Experimental Pathology* 8(3), 2365-2374.

Zhou, L., Sun, L., Wu, H., Zhang, L., Chen, M., Liu, J., & Zhong, R. (2013). Oridonin ameliorates lupus-like symptoms of MRLlpr/lpr mice by inhibition of B-cell activating factor (BAFF). *European Journal of Pharmacology*, 715(1–3), 230–237. https://doi.org/10.1016/j.ejphar.2013.05.016

Zhu, C.-S., Liu, K., Wang, J.-L., Li, J.-F., Liu, M.-F., Hao, N., Lin, Y.-X., Xiao, Z.-F. (2018). Antioxidant activities and hepatoprotective potential of *Dracocephalum rupestre* Hance extract against CCl4-induced hepatotoxicity in Kunming mice. *Journal of Food Biochemistry*, 42:e12484. https://doi.org/10.1111/jfbc.12484

Ziaei, A., Hoppstädter, J., Kiemer, A. K., Ramezani, M., Amirghofran, Z., & Diesel, B. (2015). Inhibitory effects of teuclatriol, a sesquiterpene from *Salvia mirzayanii*, on nuclear factor-κB activation and expression of inflammatory mediators. *Journal of Ethnopharmacology*, 160, 94–100. https://doi.org/10.1016/j.jep.2014.10.041

Zotsenko, L., Kyslychenko, V., Kalko, K., & Drogovoz, S. (2021). The study of phenolic composition and acute toxicity, anti-inflammatory and analgesic effects of dry extracts of some *Elsholtzia* genus (Lamiaceae) species. *Archives*, *2*, 637–649. httppharmacologyonline.silae.it

Zou, Y. H., Zhao, L., Xu, Y. K., Bao, J. M., Liu, X., Zhang, J. S., Li, W., Ahmed, A., Yin, S., & Tang, G. H. (2018). Anti-inflammatory sesquiterpenoids from the Traditional Chinese Medicine *Salvia plebeia*: Regulates pro-inflammatory mediators through inhibition of NF-κB and Erk1/2 signaling pathways in LPS-induced Raw 264.7 cells. *Journal of Ethnopharmacology*, *210*, 95–106. doi.org/10.1016/j.jep.2017.08.034

Zou, Z. Q., Ning, D. S., Liu, Y., Fu, Y. X., Li, L. C., & Pan, Z. H. (2022). Five new germacrane sesquiterpenes with anti-inflammatory activity from *Salvia petrophila*. *Phytochemistry Letters*, *47*, 111–114. doi.org/10.1016/j.phytol.2021.12.001

Zoubi, Y. E., El, L., Sidi, M., Ben, M., Boukhira, S., Ben, S. M., & Farah, A. (2016). Phytochemical Screening, Anti-inflammatory Activity and Acute Toxicity of Hydro-ethanolic, Flavonoid, Tannin and Mucilage Extracts of *Lavandula stoechas* L. from Morocco Valorisation des plantes médicinales View project pharmacological activities of plants extracts View project. *Article in International Journal of Pharmacognosy and Phytochemical Research* 8(1), 31-37.www.researchgate.net/publication/287935765

Zuo, J., Zhang, T. H., Xiong, L., Huang, L., Peng, C., Zhou, Q. M., & Dai, O. (2022). Two Pairs of 7,7′-Cyclolignan Enantiomers with Anti-Inflammatory Activities from *Perilla frutescens. Molecules*, 27(18). doi.org/10.3390/molecules27186102

Zuzarte M, Francisco V, Neves B, Liberal J, Cavaleiro C, Canhoto J, Salgueiro L, Cruz MT. (2022). *Lavandula viridis* L΄Hér. Essential Oil Inhibits the Inflammatory Response in Macrophages Through Blockade of NF-KB Signaling Cascade. *Front. Pharmacol.* 12:695911. doi: 10.3389/fphar.2021.695911

Zuzarte, M., Alves-Silva, J.M., Alves, M., ...Salgueiro, L., Cruz, M.T. (2018). New insights on the anti-inflammatory potential and safety profile of *Thymus carnosus* and *Thymus camphoratus* essential oils and their main compounds. *Journal of Ethnopharmacology*, 2018, 225, pp. 10–17

Zuzarte, M., Gonçalves, M. J., Cavaleiro, C., Cruz, M. T., Benzarti, A., Marongiu, B., Maxia, A., Piras, A., & Salgueiro, L. (2013). Antifungal and anti-inflammatory potential of *Lavandula stoechas* and *Thymus herbabarona* essential oils. *Industrial Crops and Products*, *44*, 97–103. doi.org/10.1016/j.indcrop.2012.11.002.
