# Supplementary material for: Ethnobotanical, Phytochemical, and Pharmacological Properties of the Subfamily Nepetoideae (Lamiaceae) in Inflammatory Diseases
Source: Plants (Basel). 2023 Nov 2;12(21):3752. doi: 10.3390/plants12213752 (PMC10648697; doi:10.3390/plants12213752)
Supplement: Supplementary file 1 [file plants-12-03752-s001.zip › Table S3.pdf]

Table S3. Phytochemistry related to inflammatory processes in species of the Nepetoideae (Lamiaceae) subfamily.

Specie	Plant part	Phytochemistry	Reference
Agastache mexicana Lint. & Epling	Aerial parts; inflorescences	Ursolic acid, limonene	González-Ramírez et al., 2012; Verano et al. 2013; González-Ramírez et al., 2021
Agastache rugosa Kuntze	Aerial parts; leaves	Rosmarinic acid	Kim et al., 2017; Lim et al., 2017; Lee et al., 2020
Asterohyptis stellulata (Benth.) Epling	Aerial parts	2-(3,4-dihydroxyphenil)-5,7-dihydroxy-3-[2,3,4-trihydroxy-5-(hydroxymethyl) cyclohexoxyl]; chromen-4-one; quercetin-3-O-glicoside; 2-O-(4-hydroxy-cinnamoyl), 4′-O-D-glucopyranoside.	Álvarez-Santos et al., 2022
Cedronella canariensis (L.) Webb & Berthel.	Flowers	Cedronelle, *d*-pinocarvone, tyrosol-derived fatty acids, β-sitosterol.	Lopez-García et al., 1991
Clinopodium bolivianum (Benth.) Kuntze	Aerial extract	Phenolic compounds and sugars.	Mohanty et al., 2017
Clinopodium chinense (Benth.) Kuntze	Whole parts	Naringenin, rutin,<br>hesperidin, quercitrin, kaempferitrin, caffeic acid, quercetol, kaempferol, ponciretin 7-O-α-L-rhamnopyranosyl-(1→6)-β-D-glucopyranoside, naringenin 7-O-α-L-rhamnopyranosyl-(1→6)-β-D-glucopyranoside, 3-O-{β-D-glucopyranosyl-(1→2)-[β-D-glucopyranosyl-(1→3)]-β-D-fucopyranosyl}-saikogenin F, 3-O-{β-D-glucopyranosyl-(1→2)-[β-D-glucopyranosyl-(1→3)]-β-D-fucopyranosyl}-21β-hydroxysaikogenin F, tournefolic acid B.	Yu et al., 2019; Kim et al., 2020; Li et al., 2020; Li et al., 2022; Wang et al., 2023
Clinopodium polycephalum (Vaniot) C.Y. Wu & S.J. Hsuan	Root and stem	Saturol I; 3β-22, 25-dihydroxy-tirucalla-7, 23-dieno;maslinic acid; 2α, 3α-dihydroxyolean-12-en-28-oic acid; hederagenin; 2α, 3α-dihydroxyursolic acid; alphitolic acid	Liu et al., 2022
Clinopodium tomentosum (Kunth)	Aerial parts	Rosmarinic acid; hesperidin	Tubon et al., 2020.
Clinopodium vulgare L.	Flowers	Rosmarinic acid; neochlorogenic acid; catechin; chlorogenic acid, quercetin apigenin, caffeic acid	Petrova et al., 2023; Dobrev, H.P. 2021; Amirova et al., 2019; Burk et al., 2009.

Specie	Plant part	Phytochemistry	Reference
Coleus scutellarioides (L.) Benth.	Leaves	Phytol, germacrene-d, caryophyllene, neofitadien, δ-cadinene, α-humulene; 6, 10, 14-trimethyl-2-pentadecanone; copaene, (-) caryophyllene oxide, α-selinene, methyl Eugenol, Octadecane, α-cadinol, 1,2-dimethyl-3-ethylbenzene, nonadecane, 1,3-diisopropenyl-6-methyl-cyclohexene, β-sesquiphellandrene, dokosan, vitamin A aldehyde, (+)-Aromadendrene, nerolidol, acetamide, α-calacorene, torreyol, 9-octadecenoic acid, 7-octene-4-ol, 1,1′-oxybis-dodecane, trimethyl-tetrahydronaphthalene, 2-methyl-4-(1-methyl ethyl-2-cyclohexenone, 1-(3,7-dimethyl-1-octenyl)-cyclopropanol, 5-benzofuran acetic acid, 6, 10-dimethyl-2-undecanone, α-cubebene, 1-(1-oxo-15-tetracocenil)-pyrrolidine, heneiosen, linalool, tetracosahexaene, neomenthol, heptacosane, olealdehyde, isophytol.	Mustaricihie et al., 2017; Mustaricihie et al., 2022.
Collinsonia canadensis L.	Aerial parts	Thymol; carvacrol	Mahomoodally et al., 2021
Dracocephalum heterophyllum Benth.	Whole plant	oleanolic acid, ursolic acid, pomolic acid, 2α- hydroxyl ursolic acid, apigenin-7-O-rutinoside, luteolin, diosmetin, rosmarinic acid, methyl rosmarinate., dehydrodipine-9-β-D-glucoside, verbascoside.	Shi et al. 2016; *Bian et al., 2020
Dracocephalum forrestii W.W. Sm.	Whole plant	4-hydroxy-3-methoxyphenylethanol-8-O-[(6-O-syringoyl)-β-D-glucopyranoside]; 3,4,5-trimethoxyphenyethanol β-D-glucopyranoside; 4-O-[β-D-glucopyranosyl-(1 → 3)-α-L-rhamnopyranosyl]phenylethylcinnamamide; 9″-O-n-butyl lithospermate	Li et al., 2009
Dracocephalum kotschyi Boiss.	Aerial parts and leafy branches	Apigenin	Sandraei et al. 2017; Kalantar et al., 2018; Hosseini-Sharifabad et al, 2021; Minaiyan et al., 2021
Dracocephalum taliense Forrest	Root	12-methoxy-18-hydroxy-sugiol; 2α,3α-dihydroxy-11α,12α-epoxi-urs-28,13β-olide; sugiol; abieta-8,11,13-triene; dehydroabietane; ferruginol; cryptojaponol; inuroyleanol; callitrisic acid; 11,14-dihydroxy-12,19-dimethoxy-7-oxo-8,11,13-abietatrien-19,20-olide; totarol; 7α-hydroxytotarol; sempervirol; cyclocoulterone; *E*-ergosta-6,9,22-triene-3β,5β,8α-triol; (22*E*)-ergosta-6,22-diene-3β,5β,8α-triol; estigmast-4-en-6β-ol-3-one	Deng et al., 2018
Dracocephalum moldavica L.	Aerial parts	Tilianine; oleanolic acid; Dracocefalumoids A-E, uncinatone; rosmarinic acid; caffeic acid; feluric acid; luteolin-7-O-β-D-glucuronide; tilianine; cosmosiin; apigenin-7-O-β-D-glucuronide; chrysoeriol-7-O-β-D-glucuronide; apigenin-7-O-β-D-(6"-O-malonyl) glucoside; acacetin-7-O-β-D-glucuronide; acacetin-7-O-β-D-(6"-malonyl) glucoside	Xing et al., 2013; Shen et al., 2019; Kim et al., 2021; Nie et al., 2021; Sheychenko et al., 2021

Specie	Plant part	Phytochemistry	Reference
Dracocephalum palmatum Stephan ex Willd.	Aerial parts	caffeic acid, 3-*O*-caffeoylquinic acid, rosmarinic acid, salvianolic acid B, caftaric acid, cichoric acid, umbelliferone, aesculetin, apigenin, cosmosiin, apigenin-7-*O*-β-D-glucuronopyranoside, isorhoifolin, luteolin, cynaroside, luteolin-7-*O*-β-D-glucuronopyranoside, luteolin-4⊚*O*-β-D-glucopyranoside, scolymoside, naringenin, naringenin-7-*O*-β-D-glucopyranoside, eriodictyol, eriodictyol-7-*O*-β-D-glucopyranoside, ursolic acid, oleanolic acid.	Olennikov et al. 2013; Andreyeva et al. 2020; Chirikova et al. 2021
Dracocephalum rupestre Hance	Whole plant	Eriodictyol, Phenolic and flavonoid compounds.	Ferreira et al. 2016; Zhu et al. 2017
Elsholtzia blanda Benth.	Whole plant	5, 5′-dihydroxy-7-methoxy-6,8,3′′, 3′′-tetramethylpiran-(3′, 4′)flavone; 5, 5′-dihydroxy-7-(*α*-methyl)butyroxyl-6,8,3′′,3′′-tetramethylpiran(3′,4′)-flavone; 5, 5′-dihydroxy-6, 7-methylenedioxy-8,3′′,3′′-trimethylpyran(3′,4′)-flavone.	Zheng et al., 2001
Elsholtzia ciliata (Thunb.) Hyl.	Leaves; aerial parts	Ursolic acid, Oroxylin A; caffeic acid, luteolin, apigetrin, rosmarinic acid, linarin, luteoloside, apigenin, kumatakenin; rutin, hyperoside, quercitrin, avicularin, chlorogenic acid, p-coumaric acid, luteolin-7-O-glucoside, apigenin-7-O-glucoside, diosmetin. vitexin, pedalin, luteolin-7-O-β-D-glucopyranoside, apigenin-5-O-β-D-glucopyranoside, apigenin-7-O-β-D-glucopyranoside chrysoeriol-7-O-β-D-glucopyranoside, 7,3⊚methoxy luteolin-6-O-β-D-glucopyranoside, 5,6,4′-trihydroxy-7,3′-dimethoxyflavone,5-hydroxy-6,7-dimethoxyflavone, 4-(E)-caffeoyl-L-threonic acid, 4-O-(E)-p-coumaroyl-L-threonic acid, *α*-linolenic acid; Vitexina, pedalina, lueolin, negletein, linarin, apigenin, apigetrin, diosmetin; butin; isokanin; sulfuretin, neoisoliquiritin, kumatakenin; orientin; isoorientin; swertisin; chrysoeriol; osmundacetone; gnaphaliin C; everlastoside; danshensu; pinoresinol; terpinolene; elsholtzia ketone	Wang et al., 2022; Zhang et al., 2021; Zotsenko et al., 2021; Nguyen et al., 2021; Pudziuvelyte et al., 2020; Kim et al., 2016; Kim et al., 2011
Elsholtzia densa Benth.	Aerial parts	Quercetin, 3-glycoside, apigenin, 7-(2″-acetyl-6″-methylglucuronide), betulinic acid.	Zargar et al., 2019
Elsholtzia rugulosa Hemsl.	Aerial parts	(±)-rugulolides A-C, rugulolide D. luteolin, luteolin-3⊚*O*-β-D-glucopyranoside, luteolin-7-*O*-β-D-glucopyranoside, quercetin-3-*O*-β-D-glucopyranoside, quercetin-3⊚O-β-D-glucopyranoside, apigenin, apigenin-4′-*O*-*α*-D-glucopyranoside, apigenin-7-*O*-β-D-glucopyranoside, eriodictyol 7-*O*-β-D-glucopyranoside, nepetoidin A, nepetoidin B, rosmarinic acid, methyl rosmarinate, syringaresinol, pinoresinol-4⊚*O*-β-D-glucopyranoside, pinoresinol-4,4⊚*O*-β-D-bisglucopyranoside, maltol 6′-*O*-(5-*O*-*p*-	Yang et al. 2021

Specie	Plant part	Phytochemistry	Reference
		coumaroyl)-β-D-apiofuranosyl-β-D-glucopyranosid, shimobashiraside A, prunasin, citrusin C, phenyl-β-D-glucopyranoside, 2-phenylethyl β-D-glucopyranoside, naginata ketone, oresbiusin A, tanshinol.	
Elsoltzia stachyodes (Link) C.Y. Wu	Whole plant	Tannis, flavonoids, triterpenes and saponines	Banerjee et al. 2021
Elsholtzia stauntonii Benth.	Aerial parts	Galic acid; hydroxyphenyl acetic acid; chlorogenic acid; caffeic acid; syringic acid; benzoic acid; trans-ferulic acid; sinapic acid; trans-cinnamic acid; quinic acid; rutin; quercetin-3-β-glucoside; naringenin; neohesperidin; quercetin; luteolin; Kaempferol	Zotsenko et al., 2021
Eplingiella fruticosa (Salzm. ex Benth.) Harley & J.F.B. Pastore	leaves	α-pinene, camphene, sabinene, β-pinene, limonene, 1,8-cineole, camphor, borneol, δ-elemene, α-cubebene, α-ylangene, β-elemene, β-caryophyllene, trans-muurola-3,5- diene, α-humulene, cis-murrola-4(14),5- diene, trans-murrola4(14),5-diene, Bicyclogermacrene, cis-calamenene, trans-cadina-1,4- diene, α- cadinene, spathulenol, globulol, epi-α-cadinol, α-cadinol	Beserra-Filho et al. 2019
Glechoma hederacea L.	Whole plant	Methyl isoferuloyl-7-(3,4-dihydroxyphenyl) lactate, methyl rosmarinate, ethyl rosmarinate, benzyl-4′-hydroxy-benzoyl-3′-O-β-D-glucopyranoside, 3′-O-methyl-rosmarinic acid, rosmarinic acid.<br>gallic acid; catechin hydrate; chlorogenic acid; caffeic acid; daidzin; ferulic acid; rutine; genistin; quercetin; genistein; trans-3-pinanone; 4, 5, 6, 7-tetrahydro-5-isopropenyl-3,6-β-metil-6-α-vinyl benzo furan; β-caryophyllene; spathulenol.	An et al., 2006<br>Kim et al., 2011<br>Vogl et al. 2013<br>Wang et al., 2017<br>Chou et al., 2018
Glechoma longituba (Nakai) Kuprian.	Whole plant	Apigenin-7-diglucoronide	Bian et al., 2017
Horminum pyrenaicum L.	Root	Horminone, 7-O-acetylhorminone, inuroyleanol, 15,16-dehydro-inuroroyleanol, agastaquinone, 3-deoxiagastaquinone, agastol, 15,16-dehydro-agastol	Becker et al., 2018
Hyptis capitata Jacq.	Leaves and seeds	Alkaloids, coumarins, glycosides, flavonoids, quinones.	Castro et al. 2021
Hyptis crenata Pohl ex Benth.	Leaves; Aerial parts.	1,8-cineo1e, α-pinene, camphor, β-pinene, 3-(Z)-hexenol, tricyclene, α-thujene, α-fenchene, camphene, thuja-2,4(10)-diene, myrcene, α-phellandrene, α-terpinene, p-cymene, o-cymene, γ-terpinene, terpinolene, exo-fenchol, cis-p-menth-2-en-1-ol, camphene hydrate, pinocarvone, borneol, terpinen-4-ol, p-cymen-8-ol, α-terpineol, myrtenol, α-longipinene, γ-maaliene, aromadendrene, selina-5,11-diene, α-himachalene,	De Jesus et al., 2009; Coelho-De-Souza et al., 2021; Allves-Soares et al., 2022; Shipa et al., 2022; De Lima et al., 2023;

Specie	Plant part	Phytochemistry	Reference
		α-humulene, 9-epi-E-caryophyllene, γ-himachalene, 11α-Himachala-1,4-diene, viridiflorene, β-himachalene, Spathulenol, viridiflorol, caryophylla-4(12),8(13)-dien-5β-ol, , allo-himachalol, pinene, β-caryophyllene.	
Hyptis marrubioides Epling	Leaves	α-thujene; β-thujene, α-copaene; α-caryophyllene; germacrene D; cadalene; cedrol	Botrel et al., 2010
Hyptis pectinata (L.) Poit.	Flowers	Pectinolide J; hyptolide; pectinolide E	Santana et al., 2022
Hyptis suaveolens (L.) Poit.	Leaves	Suaveolol y methyl suaveolate	Almeida-Bezerra et al., 2022
Hyptis umbrosa Salzm. ex Benth.	Leaves	Epigalocatechine; tannic acid; lupeol; borneol; gallic acid; gallocatechin; chlorogenic acid; protocatechuic acid; catechin; apigenin; kaempferol; chrysin	Anjos et al., 2017
Hissopus cuspidatus Boriss.	Aerial parts	Rosmarinic acid, 3β-hydroxy-7,8-dihydro-β-ionone, oleanolic acid, ferulic acid, ethyl ferulate, (E)-3–4-hydroxy-3-methoxyphenyl)acrylic acid, 3,4-dihydroxy benzaldehyde, quercetin, quercetin-3-O-glucopyranoside, luteolin, luteolin-7-O-rutinoside, diosmetin, diosmetin-3′-O-β-D-glucopyranoside, apigenin-6,8-di-C-β-D-glucopyranoside, 3-(4-hydroxy-3-methoxy-phenyl) acrylic acid carboxymethyl ester, caffeic acid methyl ester, Luteolin 7-O-α-L-rhamnopyranosyl (1 --> 6)-β-D-glucopyranoside, luteolin 7-O-β-D-glucuronide, diosmin, acacetin 7-O-α-L-rhamnopyranosyl(1 --> 6)-β-D-glucopyranoside.	Qin et al., 2023; Aihaiti et al., 2023; Cai et al., 2023; Ling-Fei, K. et al, 2023; Liu et al., 2021; Liu et al., 2021b: Yuan et al., 2019; Zhao et al., 2013
Hyssopus officinalis L.	Aerial parts	Chlorogenic acid; rosmarinic acid	Ma et al., 2014<br>Mohammad et al., 2019<br>Micovic et al., 2022
Isodon adenanthus (Diels) Kudô	Leaves	Adenantin	Yin et al., 2013
Isodon amethystoides (Benth.) H. Hara	Whole plant	Glaucocalyxin A	Xiang et al. 2014
Isodon coetsa (Buch.-Ham. ex D. Don) Kudô	Leaves	Spironolactone, 7α,20-epoxy-ent-kaurane	Neelamkavil Thoppil, 2013, 2014

Specie	Plant part	Phytochemistry	Reference
Isodon enanderianus (Hand. -Mazz.) H.W. Li	Aerial parts	Enanderinanine J	Na et al., 2010
Isodon eriocalyx (Dunn) Kudô		Eriocalyxin B, Phomopchalasin A, Phomopchalasin B, Phomopchalasin C, 11α,15α-diacetoxy-6β,7β-dihydroxy-7α,20-epoxi-ent-kaur-16-eno; 1α,15β-diacetoxy-6β,7β-dihydroxy-7α,20-epoxi-ent-kaur-16-ene; maoecrystal S; maoecrystal D; hikiokoshin C; maoecrystal A; eriocalyxin D; maoecrystal E	Lu et al., 2013<br>Leung et al., 2006<br>Yan et al., 2016<br>Lou et al. 2019<br>Zhang et al. 2020<br>Li et al., 2021
Isodon excisus (Maxim.) Kudô	Leaves	Inflexinol, inflexin	Lee et al. 2007; Ko et al. 2010.
Isodon henryi (Hemsl.) Kudô	Whole plant	Isohenolide A; isohenolide B; 6α, 15β-dihydroxy-6, 20-epoxi-enmein-16-en; isorubesin D; isorubesin A; epinodosin; isodocarpine; rabdoternine A; lasiokaurinin; 1α, 11α, 15β-triacetyl-6β, 7β-dihydroxyl-7α, 20-epoxi-kaur-16-ene; radbdosianin A; oridonina; effusanin A; lasiokaurin; rosthorin A; lasiodonin.	Cheng et al., 2022
Isodon japonicus (Burm. f.) H. Hara	Aerial parts	(-)-sesamin-2,2[illegible]diol, caffeic acid, isoquercitrin, rosmarinic acid, pedalitin, kamebanin, kamebacetal A, kamebakaurin,<br>excisanin A; Effusanin C: isodojaponin D.	Hwang et al. 2001; Shin et al., 2004; Hong et al., 2009; Lim et al. 2010; Kim et al., 2011; Kim et al., 2013; Kang, 2018; Ikoma et al., 2022
Isodon leucophyllus (Dunn) Kudô		11α-acetoxyeffusanin D; 6-acetylepinodosinol; 16β-ethoxymethyleneshikokianin; 16α-ethoxymethyleneshikokianin	Chen et al., 1999
Isodon melissoides (Benth.) H.W. Li	Aerial parts	Melissoidesin, α-thujene, α-pinene, camphene, sabinene, 1-Octen-3-ol, myrcene, 3-Octanol, δ-2-carene, α-phellandrene, α-terpinene, p-cymene, limonene, β-phellandrene, 1,8-Cineole, (Z)-β-ocimene, (E)-β-ocimene, γ-terpinene, acetophenone, cis-sabinene hydrate, terpinolene, linalool, trans-sabinene hydrate, camphor, menthone, borneol, menthol, terpinen-4-ol, p-cymen-8-ol, α-terpineol, thymol methyl ether, carvacrol methyl ether, bornyl acetate, thymol, carvacrol, thymol acetate, eugenol, carvacrol acetate, (E)-caryophyllene, α-trans-bergamotene, α-humulene, germacrene D, α-zingiberene, β-bisabolene, δ-cadinene, caryophyllene oxide, humulene epoxide II.	Yu et al. 2007; Kumar et al. 2021

Specie	Plant part	Phytochemistry	Reference
Isodon rugosiformis (Hand.-Mazz.) H. Hara	Aerial parts	Isorugosiformin A; isorugosiformin B; isorugosiformin C; isorugosiformin D; isorugosiformin E; isorugosiformin F	Zhang et al., 2020b
Isodon scoparius C.Y. Wu & H.W. Li) H. Hara	Aerial parts	Scopariusol L; scopariusol M; scopariusol N; scopariusol O; scopariusol P; scopariusols Q; scopariusols R: scopariusol S; scopariusol T	Jiang et al. 2018
Isodon serra (Maxim.) Kudô	Aerial parts	Oridonin, 15-acetylmegathyrin B, serrin E, 14b-hydroxyrabdocoestin A, serrin F, serrin G, 11-epi-rabdocoestin A, serrin H, serrin I, 15-acetylenanderianin N, rabdocoestin A, rabdocoestin B, enanderianin N, 1a,11b-dihydroxy-1a,11b-acetonide-7a,20-epoxy-ent-kaur-16-en-15-one, megathyrin A, enanderianin L, megathyrin B, enmein, nodosin, lasiodonin, epinodosin. isoserrin A, isoserrin B, isoserrin C, isoserrin D, isoserrin E, isoserrin F, isoserrin G, isoserrin H, isoserrin I, isoserrin J.	Zhang et al. 2005; Li et al. 2010; Zhou et al. 2013; Wan et al. 2016; Xing et al. 2020;
Isodon sculponeatus (Vaniot) Kudô	Aerial parts	sculponeatin I, sculponin A, sculponeatin A, sculponin T, sculponeatin J, sculponeatin K, sculponeatin C, sculponeatin Q, 6,7-seco-ent-kaurane diterpenoid; sculponin U–Z; wikstroemioidin A, 1α,6β,7β,15β-tetrahydroxy-7α,20-epoxy-ent-kaur-16-ene, bisjaponin A, lushanrubescensin J, ent-kaurane-7α,16β,17-triol, sculponeatin L, sculponeatin N, ent-abienervonin C, hebeiabinin B, maoyecrystal G, maoyecrystal H.	Jiang et al. 2014a; Jiang et al. 2014b.
Isodon ternifolius (D.Don) Kudô	Root	ternifoliuslignan A; ternifoliuslignan B; ternifoliuslignan C; ternifoliuslignan D; ternifoliuslignan E; ternifoliusoside F; ternifoliusoside G; ternifoliusoside H; 3-carboxi-6,7-dihydroxy-1-(3′,4′-hidroxi phenyl)-naftaleno, (+)-syringaresinol; 1-acetoxyl-2e,6e-dipiperonil-3,7-dioxabicyclo -[3,3,0]-octane; (7S,8S)-3-methoxy-3′,7-epoxi-8,4′-oxineolignan-4,9,9′ -triol, 3-(3,4-dihydroxyphenyl); acrylic-1-(3,4-dihydroxyphenyl)-2-methoxycarbonyl ethyl ester acid; oxyneolignan A; evofolin-B; 1,2-bis (4-hydroxy-3-methoxyphenyl)-1,3-propanediol	Zhang et al., 2018
Isodon rubescens (Hemsl.) H. Hara	Aerial parts	effusanin A; lasiodonin; oridonin; epinodosin; nodosin; ponicidin; rabdoternin A; enmenol; lasiokaurin; lasiokaurinol<br>isojiangrubesin A; isojiangrubesin B; isojiangrubesin C; isojiangrubesin D; isojiangrubesin E; isojiangrubesin F; isojiangrubesin G; 20(R*)-6β,7β,15β-trihydroxy-20-metoxi-7α,20-epoxy-ent-kaur-16-en-1α,11β-acetonide; nervosanin A;	Du et al., 2013<br>Zhang et al., 2017<br>He et al., 2018<br>Jia et al., 2019

Specie	Plant part	Phytochemistry	Reference
		Oridonin; rubescensin C; enmenol; longikaurin A; hebeirubescensin K; 15-acetylmegatirin B; lasiokaurinal; rostorin A; hebeirubescensin B; xerophinoid B; rabdoternin A; rabdoternin F; rabdoternin E; isodonhenrin D; asiodonin; wikstroemioidin B: 7,14-O-(1-methylethylidene)oridonin.	
Isodon wikstroemioides (Hand.-Mazz.) H. Hara	Aerial parts	isowikstroemins A–M, macrocalyxin B, pseudoirroratin A.	Wu et al. 2014; Wu et al. 2015.
Lallemantia royleana (Benth.) Benth.		Alkaloids, anthraquinones, flavonoids, glycosides, tannins, volatile oils, mixed fatty acids, terpenoids.	Bhardwaj et al., 2020; Bhardwaj Bhatia 2020
Lavandula angustifolia Mill.	Leaves; flowers	Rosmarinic acid; luteolin; apigenin; luteolin 7-O-β-D-glucoside; apigenin 7-O-β-D-glucoside; luteolin 7-O-β-D-glucuronide, limonene; eucalyptol; trans-β-ocimene; β-linalool; camphor; lavandulol; borneol; 4-terpineol; α-terpineol; lavandulol acetate; β-farnesen, pectines: chPS-L1 y chPS-L2, linalyl acetate; caryophyllene; (E)-3,7-dimethylocta-1,3,6-trieno; gallic acid; ferulic acid; quercetin; myricetin.	Hajhashemi et al., 2003; Zhao et al., 2015; Giovannini et al., 2016 Georgiev et al., 2017; Cardia et al., 2018; Souri et al., 2019; Chen et al., 2020; Donatello et al., 2020 Slighoua et al., 2022; Pandur et al., 2021; Xie et al., 2022
Lavandula coronopifolia Poir.	Aerial parts	α -Pinene, camphene, β-pinene, octan-3-ol, myrcene, yomogi alcohol , p-cymene, limonene, cineole, cis-ocimene, trans-ocimene, cis-linalool oxide, trans-linalool oxide, camphor, borneol, terpinen-4-ol, α-terpineol, linalyl formate, linalyl acetate, geranial, lavandulyl acetate, neryl acetate, caryophyllene.	Abeer et al. 2020; Nassef et al. 2022
Lavandula dentata L.	Aerial parts	α-pinene; camphene; verbenene; sabinene; p-cymene; 1,8-cineole; linalool oxide; α-campholene aldehyde; camphor; verbenol; pinocarvone; borneol; terpinen-4-ol; myrtenol; verbenone; α-selinene; α-eudesmol; Vanillic acid; chlorogenic acid; caffeic acid; coumaric acid hexoside; hydroxybenzoic acid; coumaric acid pentoside hexoside; ferulic acid hexoside; apigenin d-C-hexoside; hypolaetin di-glucuronide; luteolin glucuronide hexoside; yunnaneic acid E ; Yunnaneic acid D; Luteolin 7,4′-di-glucuronide; isoscutellarein 8-O-glucuronide; Sagerinic acid; Salvianolic acid C, Ursolic acid	Algieri et al., 2016; Contreras et al., 2018; Abdelhakim et al., 2023
Lavandula latifolia Medik.	Aerial parts	α-pinene, camphene, β-pinene, sabinene, δ-3-carene, myrcene, α-terpinene, limonene, 1,8-cineole, β-phellandrene, (Z)-β-ocimene, p-cymene, terpinolene, γ-campholene aldehyde, trans-linalool oxide, α-copaene, camphor, β-bourbonene, β-cubebene, linalyl	Shimizu et al., 1990; Carrasco et al., 2015; Vairinhos and Miguel,

Specie	Plant part	Phytochemistry	Reference
		acetate, trans-α-bergamotene, α-bergamotene, terpinen-4-ol, β-caryophyllene, lavandulyl acetate, (Z)-β-farnesene, sesquisabinene, isoborneol, α-humulene, α-terpineol, γ-terpineol, borneol, germacrene D, neryl acetate, β-bisabolene, δ-cadinene, (E)-α-bisabolene, geranyl acetate, nerol, calamenene, geraniol, (Z)-3-hexenyl nonanoate, caryophyllene oxide, carvacrol; fenchone; eremophylene; *trans*-α-necrodol; *trans*-α-necroyl acetate; tetramethyl-5-methylene-cyclopenten-2-enone; *cis*-necrodyl acetate; β-selinene; lavandulol; selina-3,7(11)-diene; Δ3-carene; myrtenol; verbenone, eucalyptol, coumarin , 7-methoxycoumarin, trans-phytol.	2020; Lucca et al., 2022; Karaka et al., 2023;
Lavandula multifida L.	Aerial parts	Maslinic acid; 15S,16-dihydroxy-7-oxopimar8(9)-ene; 15,16,17-trihydroxy-7-oxopimar-8(9)- ene, carvacrol-3-glucoside; 3*β*,19*α*, 23-trihydroxy-urs-12-en-28-oic acid; 15S,16-dihydroxyisopimar-8(9)-ene; 15,16-dihydroxy-7,11- dioxopimar-8(9)-ene; 15,16,17-trihydroxypimar-8(9)-ene; carvacrol; oleanolic acid; ursolic acid	Sosa et al. 2005
Lavandula pedunculata (Mill.) Cav.	Aerial parts	α-pinene; α-fenchene; camphene; sabinene α-fenchol; α-campholenal; β-pinene; myrcene; δ-3-carene; p-cymene; limonene; cis-β-ocimene/ trans- β-ocimene; cis-α-necrodol; 1,8-cineole; cis-linalool oxide; fenchone; linalool; thujone; camphor; menthone; borneol; p-Mentha-1,5-dien-8-ol; lavandulol; terpinen-4-ol; α-terpineol; myrtenal; myrtenol, verbenone; fenchyl acetate; eucarvone; pulegone; linalyl acetate; bornyl acetate; lavandulyl acetate; thymol; carvacrol; myrtenyl acetate; neryl acetate; lyratyl acetate; α gurjunene; caryophyllene; γ-selinene; δ-cadinene; selina-3,7(11)-diene; ledol; viridiflorol; *epi*-cubebol; τ-muurolol; β-eudesmol; α-cadinol; gentisic acid; caftaric acid, protocatechuic acid; esculetin; pinocembrin	Azevedo et al., 2015; Lopes et al., 2018; Vairinhos and Miguel 2020; Zuzarte et al. 2022; Domingues et al., 2023
Lavandula pubescens Decne.	Aerial parts	Acoradien, alloaromadendrene, α-bergamotene, α-calacorene, α-copaene, α-cubebene, α-cumene α-gurjunene, α-humulene, α-muurolene, α-phellandrene, α-terpinene, α-terpinolene, α-ylangene, β-bisabolene, β-copaene, β-elemene, β-eudesmene, β-guaiene, β-ylangene, carvacrol, caryophyllene, caryophyllene oxide, cyclosativene, E-β-ocimene, ε-caryophyllene, ledene, limonene, jolkinol D, longifolene, neo-allo-ocimene, p-cymenene, p-xylene, δ-valerolactone, thymol, calamenene.	Ali-Shtayeh et al. 2020; Ahmad et al. 2022; Acero et al. 2023.

Specie	Plant part	Phytochemistry	Reference
Lavandula stoechas L.	Aerial parts; flowers	Fenchone; camphor, caffeic acid; rosmarinic acid; 4-hydroxybenzoic acid; apigenin; gentisic acid; Apigetrin; fumaric acid; gallic acid.	Amira et al., 2012<br>Zuzarte et al. 2013<br>Zoubi et al., 2016<br>Kulabas et al., 2018
Lavandula viridis L'Her.	Aerial parts	α-pinene , 1,8-cineole, linalool, camphor , selina-3,7(11)-diene, camphene, camphor.	Skala E et al., 2020; Zuzarte et al. 2022
Lavandula x intermedia	Aerial parts	1,8-cineol, linalool, camphor , β-caryophyllene , borneol, α-pinene, β-pinene, sabinene, myrcene, limonene, p-cymene, 1-octen-3-ol, bornyl acetate, terpinen-4-ol, myrtenal, neryl acetate, carvone, cuminal, p-cymen-8-ol, caryophyllene oxide, α-bisabolol.	Carbone et al. 2018; Karakas Matpan 2022
Lepechinia caulescens (Ortega) Epling	Leaves	Ursolic acid; oleanolic acid; rosmarinic acid	Vergara-Martínez et al., 2021
Lepechinia meyenii (Walp.) Epling	Leaves	Flavonoids	Arenas-Chávez et al. 2018.
Melissa officinalis L.	Leaves	Rosmarinic acid, nerol; citral; isopulegol; caryophyllene; caryophyllene oxide, citronelle, caffeic acid, citronellal; m-cumaric acid, geranial; hesperidin, hesperetin; naringenin; neral; luteolin; eriodictiol-7-O-glucoside	Guginski et al., 2009<br>Müzell et al., 2013<br>Bohunihi et al., 2013<br>Zam et al., 2002<br>Draginic et al., 2022
Mentha arvensis L.	Whole plant; aerial parts	α-pinene; β-pinene; mircene; limonene; 3-octanol; menthone; isomenthone; methyl acetate; isopulegol; neomenthol; menthol; germacrene D; piperitone,<br>linarin	Feng et al., 2015; Thawkar et al., 2016; Demirci et al., 2021<br>Kim et al., 2021b
Mentha cervina L.	Aerial parts	Pulegone; terpinen-4-ol; carvacrol	Miguel et al., 2021
Mentha cordifolia Opiz ex Fresen.	Leaves	β-sitosterol; β-sitosterol-β-D-glucoside ; menthol	Bayat et al., 2019; Villaseñor et al., 2002

Specie	Plant part	Phytochemistry	Reference
Mentha longifolia (L.) Huds.	Whole plant	Piperitone oxide; pulegone; epoxy- trans-piperitone; menthone; 1,8-cineole; (Z)-β-ocimene; menthol; rosmarinic acid; rutin; caffeic acid; 2,5-dihydroxybenzoic acid; chlorogenic acid; vanillic acid; ferulic acid; longifolin A, eucalyptol	Karimian et al., 2013; Ibrahim et al., 2016; Murad et al., 2016; Dadkhah et al. 2018; Moshrefi-Araghi et al., 2021; Wang et al., 2022
Menta piperita L.	Leaves; aerial parts	Menthol; menthone; menthol acetate	Abdolmaleki et al. 2013; Sun et al., 2014; Li et al., 2017; Yi et al., 2022
Mentha pulegium L.	Aerial parts: leaves	Pulegone; menthol; isomenthone<br>menthone; piperitone.<br>hydroxycinnamic acids; hydroxymethoxyflavones; catechins; hydroxybenzoic acids	Luis Domingues, 2021<br>Moussaid et al., 2011<br>Brahmi et al., 2018<br>Messaoudi et al., 2022<br>Rocha et al., 2019
Mentha spicata L.	Whole plant; leaves	palmitic acid: oleic acid; linoleic acid; stearic acid,<br>stearidonic acid;<br>carvone; carvacrol; trans-carveol; piperitone oxide; limonene; 1,8-cineole; camphene; p-cymene; dihydrocarvone; pulegone; β-caryophyllene; germacrene D; menthone; *α*-pinene; linalool; ergosterol; stigmasterol; β-sitosterol; *α*-tocopherol; apigenin; naringenin; epicatechin; catechin; rutin; myricetin; luteolin; ferulic acid; coumaric acid; sinapic acid	el Menyiy et al., 2022
Mentha suaveolens Ehrh.	Aerial parts	α-pinene, sabinene, *β*-pinene, limonene, linalool, borneol, cis-dihydrocarvone, trans-carveol, carvone, carvone oxide, neo-dihydrocarveol acetate, trans-carvyl acetate, α-copaene, *β*-bourbonene, cis-jasmone, trans-*β*-caryophyllene, *β*-cedrene, *β*-copaene, α-humulene, *γ*-muurolene, germacrene D, bicyclogermacrene, *γ*-cadinene, di-epi-α-cedrene epoxide, 1,5-epoxy salvial-4(14)-ene, germacrene D-4-ol, spathulenol, globulol, 1, 10-di-epi-cubenol, epi-α-cadinol, epi-α-muurolol, bisabolene oxide, mint sulfide, α-bisabolene oxide, phytol, manoyl oxide, abietatriene, rosmarinic acid, myrcene, viridoflorol.	Moreno et al., 2002; El-Kashoury et al. 2012: Mogosan et al., 2017; Lee et al., 2021
Mentha x villosa	Leaves	Piperitone oxide	Souza PJC et al. 2009.
Micromeria biflora (Buch. Ham. ex D.Don) Benth.	Aerial part	Salicilalazina	Alhojani et al. 2022

Specie	Plant part	Phytochemistry	Reference
Minthostachys mollis Griseb.	Leaves	α-pineno; β-pineno; o-cimeno; eucaliptol; linalool; acetato de octen-1-ol; isomentona; mentona; trans-mentona; γ-elemene	Sanchéz-Tito et al. 2021
Minthostachys verticillata (Griseb.) Epling	Aerial parts; Leaves and steams	pulegone, menthone	Gonzalez-Pereira et al., 2005; Cariddi et al., 2006; Montironi et al., 2019; Rodriguez-Basso et al., 2021; Sharun et al., 2021
Monarda bradburiana L.C. Beck/ *Monarda punctata* L.	Aerial parts; Flowers	Thymol	Salehi et al., 2018
Monarda didyma L.	Aerial parts	α-phellandrene, α-pinene, α-terpinene, α-terpineol, α-terpinolene, α-thujene, β-caryophyllene, β-myrcene, β-pinene, bornyl acetate, camphene, carvacrol, carvacrol methyl ether, cis-sabinene-hydrate, D-limonene, eucalyptol, γ-cadinene, germacrene D, limonene, linalool, linalyl acetate, myrcene, p-cymene, δ-2-carene, Thymol	Cotê et al. 2021; Fraternale et al. 2022.
Monarda fistulosa L.		monardic acid A, monardic acid B, lithospermic acid B, neochlorogenic acid; chlorogenic acid, caffeic acid; luteolin-7-O-glucoside, apigenin-7-O-glucoside, rosmarinic acid, acacetin-7-O-glucoside, luteolin, apigenin, *α*-pinene, 1-octen-3-ol, *β*-phellandrene, 2-carene, p-cymene, *γ*-terpinene,thymoquinone, thymol, carvacrol, caryophyllene, *β*-copaene, 2-tert-butylhydroquinone, p-tert-butyl catechol, 1,4-eicosadiene, heptadecane, squalene, *α*-tocopherol, *β*-sitosterol.	Murata et al., 2013: Shanaida et al., 2021a; Shanaida et al., 2021b
Mosla chinensis Maxim.	Stem and leaf	α-caryophyllene, β-sitosterol, carvacrol, n-hexadecanoic acid, linoleic acid, linolenic acid, phytol, stearic acid, stigmasterol, terpinen-4-ol, thymol, apigenin-8-C-xylosyl-G-C-glucoside, isoorientin, isoorientin-2-O-rhamnoside, isoorientin-4-O-xyioside, isovitexin-2-O-rhamnoside, kaempferol 3-(2G-xylosylrutinoside), kaempferol 3-O-glucoside, kaempferol-3-O-(6 malonyl) glucoside, luteolin, luteolin-6,8-di-C-hexoside, orientin, orientin-2-O-rhamnoside, peonidin-3-O-glucoside, quercetin-acetyl- glycoside, quercetin-3-O-glucoside, quercetin-3-O-rutinoside, swertisin, vitexin-2-O-rhamnoside, (+)-catechin-3-O-b-D-gluco(2,6-bis-cinnamoyl)-pyranoside, 5,7-dihydroxy-4′-methoxy-6-methyl homoisoflavanone, gluconic acid, malic acid, citric acid, quinic acid, succinic acid, azelaic acid, caffeic acid, dicaffeoylferulic acid, dihydroxybenzoic acid hexoside, ellagic acid glucuronide, ferulic acid hexoside, p-hydroxybenzoic acid, protocatechuic acid,	Wang et al. 2021; Zhong et al. 2022.

Specie	Plant part	Phytochemistry	Reference
		rosmarinic acid, rosmarinic acid-O-hexoside, vanillic acid β-glucoside, 4-caffeoylquinic acid, 5-caffeoylquinic acid, acetyl-emodin, casticin, danshensu, diglycol laurate, everlastoside, forrestin A, gingerglycolipid, uridine, 28-β-D-glucopyranosyl-2α,3β,19α-trihydroxyolean-12-ene-24,28-dioic acid.	
Mosla scabra (Thunb.) C.Y.Wu & H.W.Li	Aerial parts	5-hydroxy-6,7-dimethoxyflavone; 5-hydroxy-7,8-dimethoxyflavone	Chen et al., 2013
Nepeta bracteata Benth.	Aerial parts	Nepetabrates E-J , 6-methyl-1,4-oxazocane-5,8-dione**,** angustanoic acid F-G, jiadifenoic acid, nepetabrates A-D, 7α-hydroxycallitrisic acid, 1-phenanthrenecarboxylic acid, nepetabrate K**,** vanguerolactone	Zhang et al. 2021; Yang et al. 2022.
Nepeta cataria Benth.	Aerial parts	Luteolin-7-0-glucuronide, luteolin-7-O-glucurono-(1-6)glucoside, apigenin-7-O-glucuronide, luteolin, apigenin, apigenin-7-O-glucoside, luteolin-7-O-glucoside, caffeic acid, rosmarinic acid, p-coumaric acid, lamiuside A, verbascoside, nepetalactone, actinidine, iridomyrmecin, *β*-caryophyllene, nerol, caryophyllene oxide, elemol, geraniol, geranial, 1,8-cineol, citronellol, citronellyl acetate, *β*-sitosterol, *α*-amyrin, ursolic acid, piperitone, humulene oxide, thymol, sabinene, *α*-humulene, *α*-pinene, *β*-farnesene, myrcene, photocitral B, citronellal, Z-isocitral, menthol, lavandulol, rosefuran epoxide, E-isocitral, terpineol, nerol, neral.	Modnicki et al. 2007; Prescott et al., 2011; Pargaien et al., 2020; Acimovic et al., 2022
Nepeta deflersiana Schweinf. ex Hedge	Aerial parts	Borneol, bulnesol, baffeic acid, bamphor, carvone, caryophyllene oxide, cemberene A, cis-carveol, decanoic acid, (E)-β-farnesene, (E)-β-caryophyllene, (E)-β-damascenone, (E)-2-hexenal, eugenol, epi-α-muuurolol, eudesm-11-en-4 α-ol, exo-fenchol, fenchone, geraniol, humulene epoxide II, irridoid glucoside 8-epi-7-deoxyloganic acid, limonene, linalool, lupeol, manool, methyleugenol, methyl rosmarinate, myrtenol, n-tetradecanoic acid, phytol, rosmarinic acid, terpinen-4-ol, thymol, trans-carveol, ursolic acid, α-alaskene, α-calacorene, α-guiaol, β-bisabolol, β-bourbonene, β-eudesmol, β–pinene, β-selinene, β-sitosterol, γ-eudesmol, τ-cadinol, 1α-hydroxy-7α,14α,18-triacetoxy-isopimara-8,15-diene, 2-methoxy-p-cresol, 2,3-dihydroxy ursolic acid, 6,10,14-trimethylpentadecane-2-one, 7-epi-α-selinene, 8-epi-7-deoxyloganic acid	Al Taweel et al. 2017; Orfali et al. 2018; Ahmad et al. 2022.
Nepeta menthoides (Boiss.) Buhse	Aerial parts	Dihydromyrcene, geranyl acetate, isothymusin, neryl acetate, rosmarinic acid, α-linalool, α-pinene, α-terpineol, β-pinene, γ-terpinene, 1, 8-cineole, 4α, 7α, 7α-nepetalactone, cirsimaritin, genkwanin	Memariani et al. 2018; Süntar et al. 2018:

Specie	Plant part	Phytochemistry	Reference
Nepeta meyeri Benth.	Aerial parts	Nepetalactone, epinepetalactone.	Cigremis et al. 2010; Süntar et al. 2018.
Nepeta pogonosperma Jamzad & Assadi	Aerial parts	1,8- cineole, 4α, 7α-nepetalactone	Ali et al., 2012; Khalighi-Sigaroodi et al., 2013
Nepeta tenuifolia Benth.	Aerial parts	Caryophyllene oxide, carvone, carveol, 1, 2-dihydroxy-8 (9) -ene-p-menthane, 2-hydroxy-2-isopropenyl-5- methylcyclohexanone, isopulegone, , limonene, menthone, menthofurane, β-myrcene, piperitenone, pulegone, pulegone oxide, schizonepetin, schizonepetoside A, schizonepetoside B, schizonepetoside C, schizonepetoside D, schizonepetoside E, schizonodiol, schizonol, γ-terpinene, verbenone, cedrenol, germacrene D, humulene, patchoulol, petafolia A, petafolia B, (+)-spatulenol, trans-phytol, maslinic acid, oleanolic acid, 2α,3α,24α-trihydroxyolean-12en28oic acid, ursolic acid, acaciin, apigenin, apigetrin, chrysoeriol-7-O-β-D-rutinoside, diosmetin, diosmetin-7-O-β-D-glucopyranoside, eupatilin, genkwanin, isorhoifolin, ladanein, luteolin, luteolin-3′,4′-dimethylether 7-O-β-D-glucuronic acid methyl ester, luteolin-3′,4′-dimethylether-7-O-β-D-rutinoside, luteolin-7-O-β-D-glucuronopyranoside, luteolin-7-O-β-D-rutinoside, luteoloside, pectolinarigenin, thermopsoside, tilianin, hesperidin, hesperetin-7-O-β-D-glucopyranoside, quercitrin, caffeic acid, trans-cinnamic acid, 2,3-di-O-cinnamoyltartaric acid, p-coumaric acid, eicosanoic acid, 3-O-feruloylquinic acid methyl ester, 4-O-feruloylquinic acid methyl ester, 3-hydroxybenzoic aicd, 12-O-β-D-glucopyranosyl oxyjasmonic acid methyl ester, Rosmarinic acid, salicylic acid, schizotenuin A, schizotenuin C1, schizotenuin C2, schizotenuin D, vanillactic acid, p-cymene-3,8-diol, daucosterol, 8,9-dehydrothymol, 3,9-dihydroxymegastigman-5-ene, 3,4-dihydroxyphenethyl alcohol-4-O-β-D-glucopyranoside, 2,6-dimethoxybenzoquinone, 5α,8α-epidioxyergosta-6,22-diol-3β-ol, isosyringin, methylconiferin, m-methylcyclohexanone, 3-methoxyl-4-hydroxycinnamic alcohol 9-O-β-D-glucopyranoside, prunasin, schitenoside A, schitenoside B, schitenoside C, stigmast-4-en-3-one, β-sitosterol	Shan et al., 2021
Ocimum basilicum L.	Aerial parts	Methyl chavicol; cineole; eugenol; methyl eugenol; elemicin; myristicin; rosmarinic acid; linalool; apigenin; ursolic acid; methyl cinnamate.	Aminian et al., 2022
Ocimum campechianum Mill.	Leaves; roots and rhizomes	Eugenol, (E)-β-caryophyllene, bicyclogermacrene, eugenol, 1,8-cineole, elemicin.	Lino et al., 2005; De Pinho et al., 2012; Pandey et al., 2014

Specie	Plant part	Phytochemistry	Reference
Ocimum kilimandscharicum Baker ex Gürke	Aerial parts	Santene, iso-citronelle, *α*-pinene, camphene, p-3-menthene, *α*-phellandrene, 2-acetyl-thiazole, limonene, 1,8-cineole, cis-arbuscolone, artemisia ketone, dihydro myrcenol, m-cymenene, terpinolene, linalool, 3-iso-thujanol, cis-verbenol, camphor, borneol, *α*-terpineol, myrtenol, verbenone, trans-piperitol, trans-carveol, carvone, *α*-ylangene, bakerol, D-germacrene, neryl isobutanone, *γ*-cadinene, vanillin acetate, flavesone, silphiperfol-5-en-3-ol, D-davanone, tetradecanal, eremoligenol, 3-iso-thujopsanone, valerianol, cadelene, sesquicineol-2-one, methyl linoleate, methyl octadecanoate.	de Lima et al., 2014; Nahak Kanta, 2014; dos Santos et al., 2021
Ocimum labiatum (N.E. Br.) A.J. Paton	Leaves	2*α*-hydroxylabda-8(17),12E,14-trien-18-oic acid, 4-epicommunic acid, labda-8(17),12E,14-triene-2R,18-diol.	Kapewangolo et al. 2015; Lambrechts Lall 2019.
Ocimum sanctum L.	Aerial parts	Eugenol; euginal; ursolic acid; carvacrol; linalool; limatrol; caryophyllene; methyl chavicol; rosmarinic acid; apigenin; cirsimaritin; isotimusin; isotimonin; orientin; vicenin.	Pattanayak et al., 2010; Singh et al., 2018
Ocimum selloi Benth.	Leaves	3-octenol, trans-ocimene, methyl chavicol, cis-anethol, *δ*-elemene, *α*-copaene, *β*-bourbonene, *β*-cubenene, *β*-elemene, methyl eugenol, trans-caryophyllene, *β*-gurjunene, *α*-humulene, allo-aromadendrene, germacrene D, *β*-selinene, *α*-(E, E)-farnesene, *δ*-cadinene, 3-octanone, bicyclogermacrene	Moraes et al., 2002; Piva et al., 2021
Origanum spp.	Aerial parts	4-caffeoylquinic acid, 3,5-dicaffeoylquinic acid, 4, 5-dicaffeoylquinic acid, 4-feruloylquinic acid, 1,3,4-tricaffeoylquinic acid, apigenin, vicenin–2, apigenin–7-O-glucoside, apigenin–7-O-pentosylhexoside, apigenin-O-hexosyl-(1 → 2)-hexoside, diosmetin-O-glucuronide, eriodictyol-O-acetyldipentoside, fustin-O-(hydroxymethylphenyl)-O-hexoside, hispidulin-O-pentosylhexoside, hispidulin-O-rhamnoside, homoorientin, luteolin–7-O-glucoside, luteolin–7-O-pentosylhexoside, naringenin, phlorizin, isoquercitrine, 3-hydroxyphlorizin, caffeic acid, gallic acid ethyl ester, gallic acid-O-hexoside, gentisic acid, gentisic acid derivative, gentisic acid-(dihydroxy-phenyl-methyl)-O-hexoside, gentisic acid- (hydroxy-phenyl-methyl)-O-dihexoside, gentisic acid-(hydroxy-phenyl-methyl)-O-hexoside, o-coumaric acid, p-coumaric acid, protocatechuic acid, protocatechuic acid O-hexoside, vannilic acid, lithospermic acid, rosmarinic acid, salvianolic acid H/I, salvianolic acid, *α*-thujene, *α*-pinene, *β*-myrcene, *α*-phellandrene, (+)-4-Carene, p-cymene, *β*-pinene, linalool, carvacrol, thymol, caryophyllene, camphene, *α*-terpinene, camphor, borneol, terpinen-4-ol, *α*-terpineol, carvacrol.	Kogiannou et al. 2013; Zengin et al. 2019; El kharraf et al., 2020; Hamamouchi et al., 2021; Sharifi-Rad et al. 2021; Al-Mijalli et. al., 2022

Specie	Plant part	Phytochemistry	Reference
Orthosiphon aristatus (Blume) Miq.	Aerial parts; leaves	Ursolic acid; oleanolic acid	Hsu et al., 2010; Vijayan et al., 2017
Orthosiphon stamineus Benth.	Aerial parts	Orthosiphone A; orthosiphone B; orthosiphon A; orthosiphon B; betulinic acid; β-elemene; caffeic acid; sinensetin; eupatorin; β-amyrin; camphor; terpineol; lirol; valencene.	Singh et al., 2015
Perilla frutescens (L.) Britton	Leaves; seed	Ursolic acid; corosolic acid; 3-epicorosolic acid; pomolic acid; tormentic acid; hiptadienic acid; oleanolic acid; augustic acid; 3-epimaslinic acid; linoleic acid; palmitic acid; stearic acid; linoleic acid; linolenic acid; luteolin; magnosalin; perfrancin	Banno et al., 2004; Huang et al., 2014; Ueda et al., 2002; Zuo et al., 2002; Liu et al., 2022b; Kangwan et al., 2021; Zhao et al., 2022.
Platostoma africanum P. Beauv.	Aerial parts	Ursolic acid; oleanolic acid; epimaslinic acid; corosolic acid; tomentic acid; β-sitosterol; stigmasterol.	Aladedunye et al., 2008
Plectranthus caninus Vatke	Aerial parts	α-thujone; thujo-2,4(10)-diene; α-terpinene; p-cymene; fenchone.	Tadesse et al., 2011
Plectranthus ecklonii Benth.	Aerial parts	Parvifloron D; β-sitosterol; stigmasterol	Andrade et al., 2018
Plectranthus ornatus Codd.	Aerial parts	(11R*,13E)-11-acetoxyhalima-5,13-dien-15-oic acid; 1α,6β-diacetoxy-8α,13R*-epoxy-14-labden-11-one; 1,6-di-O-acetylforskolin and, 1,6-di-O-acetyl-9-deoxyforskolin	Sitarek et al., 2022.
Plectranthus zeylanicus Benth.	Whole plant	Eudesm-7(11)-en-4-ol, hexadecanoic acid, phytol, 9,12,15-octadecatretinoic acid, callitrisic acid, cholest-5-en-3β-ol, ergosta-5,22-dien-3β-ol, campesterol, stigmasterol, β-sitosterol, β-amyrin, α-amyrin, stigmast-4-en-3-one, cinncassiol, coleone, stigmasterol-5,22,25-trien-3-β-ol, 7α-acetoxy-6β-hydroxyroyleanone.	Napagoda et al., 2014. Napagoda et al., 2022.
Prunella vulgaris L.	Aerial parts; leaves and stem; whole plant; fruit spikes	2α, 3α, 23-trihidroxiursa-12,20(30)-dien-28-oic acid, β-amyrin, eusapic acid; hexadecanoic acid; ethyl palmitate; phytol ; ethyl linoleate; linoleic acid; nerol; linalyl formate; betulinic acid; ursolic acid; 2α, 3α-hydroxy-urs-12-ene-28-oic acid; 2α, 3α-dihidroxiursolic acid; prunellanate A, prunellanate B, prunelladiterpenel A, gallic acid; caffeic acid; rutin; rosmarinic acid; quercetin.	Park et al., 2013; Li et al., 2020 Choia et al., 2016; Hwang et al., 2013; Yan, 2016; Tang et al., 2022 Ryu et al., 2000; Zheng et al., 2022 Zhang et al., 2018b

Specie	Plant part	Phytochemistry	Reference
Salvia aethiophis (Kunth) Lint & Epling	Root	Aethiopinone, rosmarinic acid, luteolin; apigenin	Hernández-Pérez et al., 1995; Benrezzouk et al., 2001; Nworu and Akah, 2015; Vulganová et al., 2019
Salvia africana-caerulea L.	Aerial parts	rosmarinic acid, yunnaneic acid, carnosic acid, carnosol, caffeic acid	Komatou et al., 2006; Kamatou et al., 2010; Bonito et al., 2011; Afonso et al., 2019a
Salvia albicaulis Benth.	Aerial parts	viridiflorol, 1,8-cineole, limonene.	Kamatou et al., 2007.
Salvia apiana Jeps.	Aerial parts	Quinic acid, danshensu, protocatechuic acid, caffeoylquinic acid, luteolin, hesperidin, quercetin, rutinoside, rosmarinic acid, sagerinic, salvianolic acid, cirsimaritin, rosmanol, cornosol, carnosic acid	Afonso et al., 2019b
Salvia barrelieri Benth.	Aerial parts	3β-acetoxy-olean-18-ene-2α-ol; pi-germanidiol; olean-18-ene-1β,2α,3β-triol; micromeric acid; ursolic acid; salvigenin; apigenin-7-O-β-d-glucopyranoside methyl ester; apigenin-7-O-β-d-glucopyranoside; apigenin-7-O-β-d-glucopyranoside; apigenin, cinaroside.	Lehbili et al., 2018
Salvia ceratophylla L.	Aerial parts	Linalool, germacrene D, β-caryophyllene, bicyclogermacrene, sclareol, spathulenol, 1,8-cineole, α-terpineol, α-copaene, kaempferol, pseudobaptigenin.	Shehadeh et al., 2014; Bonesi et al., 2017; Abu-Darwish et al., 2020
Salvia chamelaeagnea Bergius	Aerial parts	α-eudesmol, α-gurjunene, α-humulene, α-pinene, α-terpineol, α-thujene, allo-aromadendrene, aromadendrene, β-caryophyllene, β-eudesmol, β-gurjunene, β-pinene, betulafolientriol oxide, caffeic acid, camphene, carnosic acid, carnosol, carvone, caryophyllene alcohol, caryophyllene oxide, cis-sabinene hydrate, (E)-β-ocimene, (E)-nerolidol, Epi-cubenol, eudesmadiene, γ-cadinene, globulol, ledol, limonene, linalool, myrcene, oleanolic acid, rosmarinic acid, ursolic acid, viridiflorene, viridiflorol, (Z)-β-ocimene, 7-O-methyl-epirosmanol.	Kamatou et al. 2006; Kamatou et al. 2010
Salvia compressa Vent.	Root	Citrostadienol; β-sitosterol; linolenic acid, linoleic acid; palmitic acid; geraniol.	Noorbakhsh et al., 2022
Salvia desoleana Atzei & P. Picci	Aerial parts; leaves	β-pinene, cineole, α-terpineol, linalool, linalyl α-terpinyl acetate, linalyl acetate.	Peana Satta, 1993; Peana et al., 1999; Ceschel et al., 2000; Peana moretti, 2002.
Salvia digitaloides Diels	Root	Salviatalin A; salvitrijudin A	Wu et al., 2010

Specie	Plant part	Phytochemistry	Reference
Salvia dolomitica Codd.	Aerial parts	geraniol; Linalyl acetate; linalool; α-terpineol; cis-β-ocimene; sabinene; nerol oxide	Kamatou et al., 2007
Salvia fruticosa Mill.	Root; aerial parts	Luteolin; rutin	Boukhary et al., 2016
Salvia glutinosa L.	Aerial parts	Caffeic acid hexoside, apigenin-C-dihexoside, quercetin-O-hexoside, sagerinic acid, isorhamnetin-O-hexoside, rosmarinic acid, baicalein, luteolin acetyl-glucoside, salvianolic acid J, salvianolic acid A.	Nicolescu et al., 2022
Salvia hierosolymitana Boiss.	Aerial parts	3β,6α,23-trihydroxyurs- 12,19(29)-dien-28-oic acid; 23-(trans-p-coumaroyloxy)- 3β,6α,30-trihydroxyurs-12-en-28-oic acid; 2α,3β-dihydroxyolean-28-oic acid; 24-nor-2a,3b-dihydroxyolean-4,12-ene, 2α,3β,23-trihydroxyurs-12-en-28-oic; 3β,23-dihydroxyurs-12-en-28-oic acid; maslinic acid; arjunolic acid	De Felice et al., 2006; Miranda et al., 2022
Salvia hydrangea DC. ex Benth.	Aerial parts	β-pinene, 1-8-cineole, β-caryophyllene, sclareol.	Sonboli et al. 2009; Asadollahi et al., 2019;
Salvia japonica Thunb.	Aerial parts	α-terpinyl acetate camphor; linalool; triacetin; eucalyptol; (+)-2-bornanone; L-α-pinene; triacetin; plastolin I ; D-limonene	Li et al., 2021.; Sun et al., 2022.
Salvia lachnostachys Benth.	Leaves	Fruticulin A	Piccinelli et al., 2014.
Salvia lanceolata Lam.	Leaves	Oleanolic acid, ursolic acid, betulafolientriol oxide, carnosol, rosmarinic acid, carnosic acid caffeic acid.	Kamatou et al., 2010.
Salvia lanigera Poir.	Leaves	Podocarpa-5,8,11,13-tetraen-7-one; 13-hydroxy-14-isopropyl; sclareol ; sugiol; ferruginol, aromadendrene, cis-α-bisabolene, 1,8-cineole eucalyptol ; camphor; α-terpineol; bornyl acetate; α-terpinenyl acetate; veridiflorol ; caryophyllene oxide	Alonazi, et al., 2021.
Salvia lavandulifolia Vahl		α-pinene, sabinene, limonene, 1,8-cineole, linalool, camphor, borneol, terpinen-4-ol, linalyl acetate, α-terpinyl acetate, sabinil acetate.	Porres-Martínez et al., 2013
Salvia leriifolia Benth.	Aerial parts; leaves; seed	rosmarinic acid; caffeic acid; salvianolic acid B; camphor; 1,8-cineole; camphene; α-pineno	Hosseinzadeh Yavary, 1999; Loizzo et al., 2009; Hosseinzadeh et al., 2003; Modarres et al., 2014

Specie	Plant part	Phytochemistry	Reference
Salvia miltiorrhiza Bunge	Root	Tanshinone I; tanshinone IIA; tanshinona IIB; cryptotanshinone; cryptotanshinone I; danshensu; rosmarinic acid; caffeic acid; lithospermic acid, salvianolic acid A-N; danshenxinkun A; przewaqionona A	Feng et al., 2021<br>Mahalaksmi et al., 2021<br>Yang et al., 2022
Salvia mirzayanii Rech. f. & Esfand.	Aerial parts	α-terpinyl, linalyl acetate, 1,8-cineole, linalool, γ-cadinene, spathulenol, α-cadinol, (Z)-nerolidol, 5-neo-cedranol, germacrene, salvigenin, eupatorin, catechin, cirsimaritin, chrysoeriol, luteolin, rutin, rosmarinic acid, teuclatriol.	Zarshenas et al., 2014; Ziaei et al., 2015; Amirghofran et al., 2011
Salvia muirii (L.) Bol.	Aerial parts	Rosmarinic acid, carnosic acid, salvigenin, betulafolientriol oxide, carnosol, caffeic acid, oleanolic and ursolic acid, 1,8-cineole, α-pinene limonene.	Kamatou et al., 2006; Kamatou et al., 2010.
Salvia multicaulis Vahl	Aerial parts	Gallic acid; rosmarinic acid; chlorogenic acid; vanillin; p-coumaric acid; quercetin.	Rowshan Najafian, 2020
Salvia nipponica Miq.	Root; leaves	Taxodione; (+)-valeranone; nubiol; rosmarinic acid; salvianolic acid B; ursolic acid; 3-epicorosolic acid; caffeic acid; p-coumaric acid; vanillic acid; nicotinic acid	Chan et al., 2011
Salvia officinalis L.	Leaves	1,8-cineole; camphor; α-thujone; β-thujone; borneol: viridiflor, tannic acid; oleanolic acid; ursolic acid; carnosol; carnosic acid; fumaric acid; chlorogenic acid; niacin; rosmarinic acid; luteolin-7-glucoside.	Miraj Kiani, 2016
Salvia palaestina Benth.		Apigenin; Apigenin 7-O-glucuronide; rosmarinic acid; methyl rosmarinate	Mehmet et al., 2021
Salvia petrophilla G. X. Hu, E. D. Liu & Yan Liu	Whole plant	Petrofin A; petrofin B; petrofin C; petrofin D; petrofin E	Zou et al., 2022
Salvia plebeia R. Br.	Leaves; aerial parts	1β-acetoxy-8β-hydroxy-2-oxoeudesman-3,7(11)-dien-8,12-olide; plebeiolide B; 8-methoxyplebeiolide B; plebeiolide A; eudebeiolide E; phaeusmane G; eudebeiolide F; plebeiolide C; eudebeiolide A; eudebeiolide B; eudebeiolide C; eudebeiolide G; eudebeiolide H, (1S,5S,8S,10R)-1-acetoxy-8-methoxy-2-oxoeudesman-3,7(11)-dien-8,12-olide; eudebeiolide I; eudebeiolide J; 6-hydroxyplebeiolide A; eudebeiolide K; 8-epi-eudebeiolida C, Salviplenoide A; salviplenoide B; salviplenoide C; salviplenoide D; salviplenoide E; salviplenoide F, luteoloside; nepitrin; homoplantagenin; luteolin; nepetin; hispidulin; eupatorin	Jeong et al., 2012; Akram et al., 2015; Jang et al., 2016; Jang et al., 2017; Zou et al., 2018
Salvia prionitis Hance	Aerial parts	Prionidipene A, prionidipene B, prionidipene C, prionidipene D, prionidipene E	Li et al., 2018.

Specie	Plant part	Phytochemistry	Reference
Salvia przewalskii Maxim.		Rosmarinic acid, salvianolic acid B, tanshinone I, dihydrotanshinone I	Wang et al., 2010; Yang et al., 2017
Salvia radula Benth.	Aerial parts	Rosmarinic acid, salvigenin, betulafolientriol oxide.	Kamatou et al., 2010.
Salvia repens Burch. ex Benth.	Aerial parts	Rosmarinic acid, caffeic acid, carnosol, carnosic acid	Kamatou et al., 2010.
Salvia rosmarinus (L.) J.B. Walker, B.T. Drew & J.G. González (Syn. *Rosmarinus officinalis* L.)	Aerial parts	*β*-pinene, 1,8-cineole, borneol, camphor, limonene, verbenone, carnosol, carnosic acid, rosmarinic, ursolic, oleanolic, micromeric acid.	Sousa et al., 2019; Malvezzi et al., 2020
Salvia runcinata L. f.	Aerial parts	*α*-pinene, 1,8-cineole, linalool, limonene, myrcene, β-caryophyllene, spathulenol, β-caryophyllene oxide, viridiflorol, δ-3-carene; *α*-bisabolol. *α*-bisabolol, rosmarinic acid; *α*-humulene, guaiol, *α*-bisabolol, manool; caffeic acid	Komatou et al., 2005; Kamatou et al., 2008; Kamatou Viljoen, 2010; ; Komatou et al., 2010a.
Salvia sagittata Ruiz Pav.	Aerial parts	rosmarinic acid; chlorogenic acid, quercetin-3-O-glucoside, hesperetin, cinnamic acid, syringic acid.	Mahmoud et al., 2019<br>Tubon et al., 2019
Salvia sclarea L.	Aerial part; leaves	Rosmarinic acid; apigenin-7-O-glucoside<br>cineole; linalool; linalyl acetate; *α*-terpineol; methyl chavicol, sclareol	Moretti et al., 1997; Zhong et al., 2015; Kostic et al., 2017; Yang et al., 2022
Salvia splendens Sellow ex Nees	Aerial parts	rosmarinic acid; apigenin-7-glucoside, caffeic acid, vanillic acid, ferulic acid, rutin, p-coumaric acid, cinnamic acid, luteolin, apigenin, kaempferol; chrysin.	El Sawi et al., 2021
Salvia verbenaca L.	Whole plant; aerial parts	1,8-Cineole, p-cymene, *α*-pinene, *γ*-terpinene, *β*-caryophyllene, viridiflorol, epi-13-manool, thymol, limonene, camphor, germacrene D, manool, copaene, cadinol, *δ*-cadinene, p-cymen-8-ol, *α*-humulene, *α*-thujone, (Z)-*β*-ocimene, camphene, *α*-terpineol, bornyl acetate, bicyclogermacrene, *β*-ionone, Epi-*α*-cadinol, *cis*-muurola-4(14),5-diene, muurola-3,5-diene, spathulenol, *cis*-calamenene, 1,10-di-epi-cubenol, (Z)-*β*-ocimene, *β*-eudesmol, isopentyl isovalerate, *α*-gurjunene, *trans*-sabinene hydrate acetate, *β*-bourbonene, *E*-*β*-ocimene, *α*-copaene , *α*-phellandrene, *β*-cubebene, *cis*-*β*-guaiene, *β*-	Kamatou et al., 2008; Bonesi et al., 2017; Taarit et al., 2010; Khouchlaa et al., 2022

Specie	Plant part	Phytochemistry	Reference
		cedrene, p-methyl-acetophenone, 1,10-di-epi-cubenol, (E)-*β*-farnesene, *α*-muurolol, neryl acetate, fenchone, 9,12,15-octadecatrienal, (*E*)-*β*-ionone, *cis*-Muurola-3,5-diene, *γ*-amorphene, carvacrol, hexahydrofarnesyl acetone, p-cymen-8-ol, tricyclene, *α*-calacorene, palmitic acid, arachidic acid, stearic acid, arachidic acid	
Salvia virgata Ortega	Aerial parts	Rosmarinic acid; gallic acid, p-benzoic acid, caffeic acid, o-coumaric acid, luteolin-7-O-glucoside, luteolin; methyl rosmarinate, salvianolic acid F; verbascoside leucosceptoside A isoverbascoside martynoside	Akkol et al., 2008; Küpeli E et al. 2008; Grzegorczyk-Karolak et al. 2022
Salvia yunnanensis C.H. Wright	Aerial parts	Yunnannin A; danshenol C	Bonito et al. 2011
Satureja bachtiarica Bunge	Leaves	Anethole; thymol; carvacrol; piperitenone; carvacryl acetate; caryophyllene aromadendrene *α*-curcumene; ledene: *β*-bisabolene; cyclohexene; *α*-bisabolene; spathulenol; caryophyllene oxide.	Bakhtiarpoor et al., 2018
Satureja cuneifolia Ten.	Aerial parts in flowering	Thymol; p-cymene; terpinene; carvacrol.	Momtaz Abdollahi 2010; Taskin et al. 2020
Satureja hortensis L.	Leaves and flowers; aerial parts; seed	α-thujone, α-pinene; β-pinene; myrcene; α-terpinene; p-cymene; γ-terpinene; thymol; carvacrol acetate; β-caryophyllene	Hajhashemi et al., 2002 Hajhashemi et al., 2012
Satureja khuzistanica Jamzad	Aerial parts	Carvacrol, p-cimene; γ-terpinene; (Z)-β-ocimene; α-terpinole, α-terpinene; α-tujona; α-pinene.	Amanlou et al., 2005; Abbasloo et al., 2016; Darabad et al., 2022.
Satureja montana L.	Aerial parts	rosmarinic acid; hyperoside; rutin; chlorogenic acid; p-coumaric acid; caffeic acid; epicatechin; carvacrol	Miguel et al. 2021; Milijasevic et al., 2022
Satureja sahendica Bornm.	Aerial parts and seed	Thymol	Salehi et al., 2018; Omarizadeh et al., 2021
Satureja spicigera (K.Koch) Boiss.	Aerial parts	Thymol,p-cymene, terpinene, carvacrol	Momtaz Abdollahi 2010; Salehi et al. 2018

Specie	Plant part	Phytochemistry	Reference
Satureja thymbra L.		α-pinene, myrcene, γ-terpinene, p-cymene, linalool, β-caryophyllene, α-terpineol, borneol, geraniol, caryophyllene oxide, thymol, carvacrol	Karabay-Yavasoglu et al., 2006.
Tetradenia riparia (Hochst.) Codd	Leaves; stem	Limonene, 1,8-cineole, γ-terpinene, α-pinene, fenchone, α-terpineol, fenchyl alcohol, β-caryophyllene, perillyl alcohol endo-fenchol, camphor, borneol, terpinen-4-ol, geraniol, thymol, α-copaene, β-caryophyllene, α-trans-bergamotene, γ-gurjunene, α-muurolene, germacrene D, α-humulene, viridiflorol, isopimara- 8,15-diene , manoyl oxide.	Ghuman et al., 2019; Garzoli et al. 2022; Shimira 2022;
Thymbra capitata (L.) Cav.	Aerial parts	Carvacrol; thymol; p-cymene, γ-terpinene; linalool; β-caryophyllene; α-thujene, α-pinene, β-myrcene, α-terpinene, camphene, 3-octanone, β-pinene, α-phellandrene, 3-carene, terpinen-4-ol, α-terpineol, carvacrol methyl ether, α-humulene, ledene, β-bisabolene, δ-cadinene, caryophyllene oxide, rosmarinic acid-O-hexoside, syringaresinol-O-hexoside, hesperidin, rosmarinic acid, salvianolic acid, , salvianolic acid A, oxo-dihydroxy-octadecenoic acid, naringenin, apigenin, trihydroxy-octadecenoic acid. 6-hydroxyluteolin, 4-tert-butyl catechol, vanillic, acid, protocatechuic acid, taxifolin, eriodictyol, 6-hydroxyluteolin 7,3′-dimethyl ether, aromadendrin, B/E, eriodictyol, luteolin, 6-hydroxyluteolin, 7,3′,4′-trimethyl ether, ladanein, genkwanin.	Albano Miguel, 2011<br>Aazza et al., 2016<br>Taşkın et al., 2018<br>Aazza et al., 2016; Albano et al., 2011; Carrasco et al., 2016; Llorent-Martínez et al., 2022; Miguel et al., 2021; Saoulajan et al., 2022; Tsioutsiou et al., 2022.
Thymbra spicata L.	Leaves, stems and flowers on button	Carvacrol, γ-terpinene, p-cymene, β-caryophyllene, thymol.	Güneş et al., 2017; Karakaş et al., 2022; Mendi et al., 2017; Mohsenzadeh et al., 2016.
Thymus albicans Hoffmanns. & Link	Flowering parts	Tricyclene, α-pinene , camphene, sabinene, β-pinene, dehydro-1,8-cineole, β-myrcene, α-terpinene, p-cymene, limonene, trans-sabinene hydrate, camphor, geraniol, bornyl acetate, bornyl propionate. β-caryophyllene, allo-aromadendrene, bicyclogermacrene, globulol, viridiflorol, ledol, trans-verbenol, δ-terpineol.	Aazza etal. 2016; Roxo et al. 2020
Thymus caespititius Brot.	Flowers, stems and leaves	Apigenin quercetin-O-glucoside A, luteolin-O-rutoside, apigenin-O-glucoside A, rosmarinic acid, α-thujene, α-pinene, sabinene, 1-octen-3-ol, β-pinene, α-phellandrene, α-terpinene p-cymene, β-phellandrene limonene, trans-sabinene hydrate, terpinolene, thymol, carvacrol, trans-dihydroagarofuran.	Aazza et al. 2016; Alonso et al. 2017

Specie	Plant part	Phytochemistry	Reference
Thymus camphoratus Hoffmanns. & Link	Aerial parts in flowering	α-thujene, α-pinene, camphene, sabinene, β-pinene, myrcene, α-phellandrene α-terpinene, p-cymene, limonene, 1,8-cineole, Z-ocimene, γ-terpinene, cis-linalool oxyde, α-campholenal camphor, E-pinocarveol, E-verbenol, myrtenal, α-terpineol, myrtenol, E-carveol, carvone, geraniol, bornyl acetate, α-terpinyl acetate, α-cubebene, geranyl acetate, E-caryophyllene, aromadendrene.	Albano Miguel 2011; Zuzarte et al. 2018
Thymus citriodorus (Pers.) Schreb.	Aerial parts	1, 8-cineole; geraniol	Oliveira et al., 2022
Thymus daenensis Celak	Aerial parts	Thymol, carvacrol, linalool	Akbarinia etal. 2008; Soosani Sazegar, 2018
Thymus herbabarona Loisel.	flowers, leaves and stems	Caffeic acid, eriodictyol-O-glucoside, quercetin-O-glucoside A, luteolin-C-glucoside A, rosmarinic acid, caffeic acid, thujene, pinene, camphene, 3-octanone, myrcene, 3-octanol, terpinene, p-cymene, limonene, terpinolene, linalool, borneol, thymol methyl ether, carvacrol methyl ether.	Alfonso et al. 2017; Zuzarte et al. 2013
Thymus linearis Benth.	Leaves and stems	Thymol, p-cymene; germacrene-D, γ-terpinene.	Chandra et al. 2016; Qadir et al. 2016
Thymus longicaulis C. Presl		Terpinene, thymol, p-cymene; quinic acid; stachyose, 12-hydroxyjasmonic acid, lariciresinol O-dihexoside; rosmarinic acid O-hexoside, luteolin-O-hexoside, luteolin-O-pentoside, salvianolic acid K, luteolin-O-(acetylpentosyl)pentoside, methylapigenin, isosalvianolic acid, salvianolic acid K.	Galasso et al., 2014
Thymus praecox Opiz	Aerial parts	Chlorogenic acid, luteolin-7-O-glucoside, 3-O-feruloylquinic acid, quercetin-3-O-hexoside, apigenin-7-Oglucuronide	Cam et al. 2019; Taskin et al. 2019
Thymus quinquecostatus Celak		Thymol, γ-terpinene, p-cymene, geraniol, geranyl acetate, borneol , linalool, caryophyllene, anethole , carvacrol, 2-isopropyl-1-methoxy-4-methylbenzene, p-vinyl guaiacol, bicyclo [2.2.1] heptan-2-one, limonene, α-pineno, camphene, β-myrcene, α-terpineol, ascabin	Oh et al., 2008;<br>Kim et al., 2022.
Thymus serpyllum L.	Aerial parts	Camphene; myrcene; 1,8-cineole; limonene; camphor; α-terpineol; linalyl acetate; geranyl acetate; β-caryophyllene; germacrene D.	Raal et al., 2004; Ruiz-Malagón et al., 2022; Ruiz-Malagón et al. 2022b

Specie	Plant part	Phytochemistry	Reference
Thymus armeniacus Klokov & Des.-Shost.	Aerial parts	Sevanol, luteolin-7-0-glucuronide, luteolin-7-O-glucurono-(1-6)glucoside, apigenin-7-O-glucuronide, luteolin, apigenin, apigenin-7-O-glucoside, luteolin-7-O-glucoside, caffeic acid, rosmarinic acid, p-coumaric acid, lamiuside A, verbascoside, nepetalactone, actinidine, iridomyrmecin, *β*-caryophyllene, nerol, caryophyllene oxide, elemol, geraniol, geranial, 1,8-cineole, citronellol, citronellyl acetate, *β*-sitosterol, *α*-amyrin, sitosterol *β*-glucopyranoside, ursolic acid, piperitone, humulene oxide, dimethyl-3,7-oxa-1-bicyclo [3,3,0]oct-2-ene, hexenyl benzoato, thymol, sabinene, *β*-farnesene, photocitral B, citronellal, menthol, lavandulol, rosefuran epoxide, E-isocitral, neral.	Modnicki et al. 2007; Prescott et al., 2011; Pargaien et al., 2020; Acimovic et al., 2022
Thymus broussonetii Boiss.	Whole plant; leaves	Borneol, p-cymene, carvacrol, camphene, *α* -terpinene, *α*-pinene, trans-sabinene hydrate, caryophyllene oxide, (E)-β-caryophyllene, bornyl acetate, carvacrol methyl ether, camphor, linalool, cis-sabinene hydrate, 4-terpineol, p-cymen-8-ol, thymol, trans-verbenol, 1-octen-3ol, 1,8-cineol, β-pinene, (E)-β-caryophyllene, geraniol formate, p-menth-1,4(8)-diene, linalyl propionate, β-cadrene, thujol, cinerone, 1-octen-3-ol, viridiflorene, borneol, γ-terpinene, myrcene, camphene, *α*–thujene, aromadendrene, caryophyllene oxide, germacrene D, δ-cadinene, eucalyptol, *τ*-muurolol, γ–cadinene, ledene, luteolin, thymonin, eriodictyol, luteolin-7-O-glucoside, luteolin-3′-O-glucuronide, eriodictyol-7-O-glucoside	Ismaili et al., 2002; Salehi e al., 2018; Naceiri et al., 2021; El Yaagoubi et al., 2021;
Thymus carnosus Boiss.	Aerial parts	Santene, iso-citronelle, *α*-pinene, camphene, p-3-menthene, *α*-phellandrene, 2-acetyl-thiazole, limonene, 1,8-cineole, cis-arbuscolone, artemisia ketone, dihydro myrcenol, m-cymenene, terpinolene, linalool, myrcenol, 3-iso-thujanol, cis-verbenol, camphor, borneol, verbenone, trans-piperitol, trans-carveol, carvone, *α*-ylangene, bakerol, D-germacrene, neryl, vanillin acetate, flavesone, silphiperfol-5-en-3-ol, D-davanone, eremoligenol, 3-iso-thujopsanone, valerianol, cadelene, sesquicineol-2-one, methyl linoleate.	Nahak and Kanta, 2014; dos Santos et al., 2021; de Lima et al., 2014;
Thymus kotschyanus Boiss. & Hohen.	leaves	3-octenol, trans-ocimene, methyl chavicol, cis-anethol, *δ*-elemene, *α*-copaene, *β*-bourbonene, *β*-cubenene, *β*-elemene, methyl eugenol, trans-caryophyllene, *β*-gurjunene, *α*-humulene, allo-aromadendrene, germacrene D, *β*-selinene, *α*-(E, E)-farnesene, *δ*-cadinene.	Moraes et al., 2002; Piva et al., 2021
Thymus mastichina L.	Aerial parts	1,8- cineole, *α*-pinene, camphene, sabinene, *β*-pinene, camphor, *γ*-terpinene, borneol, terpinen-4-ol, *γ*-terpineol	Albano Miguel 2011; Aazza et al., 2016; Oliveira et al., 2023

Specie	Plant part	Phytochemistry	Reference
Thymus sipyleus Boiss.		rosmarinic acid, luteolin-7-O-glucoside, p-cymene, γ-terpinene, trans-dihydrocarvone, γ-muurolene, γ-cadinene, caryophyllene oxide, (R)-5-isopropyl-2-methylcyclohexa-1,3-diene, α-thujene, carvacrol methyl ether, sabinene hydrate, β-bisabolene, β-caryophyllene, β-phellandrene, crithmene, borneol, carvacryl acetate, camphene, isothymol, carvacrol, terpinolene, 1-octen-3-ol, 1-methyl-4-isopropenylbenzene, pinene, carvone, limonene.	Günes et al., 2017; Demirci et al., 2018; Ustuner et al., 2019; Llorente-Martínez et al., 2022
Thymus pulegioides L.	Aerial parts	Luteolin, apigenin, caffeic acid, rosmarinic acid.	Stalinska et al., 2005; Pavel et al., 2011
Thymus vulgaris L.	Aerial parts	α-thujone; α-pinene; camphene; myrcene; p-cymene; α-terpinene; eucalyptol; sabinene hydrate; borneol; terpinen-4-ol; thymol.	Bacalbasa et al. 2022<br>Pandur et al., 2022
Zataria multiflora Boiss.	Aerial parts	Thymol; carvacrol; linalool; p-cymene; γ-terpinene; α-pinene; eucalyptol; β-caryophyllene; borneol; 3-sitosterol; stigmasterol; oleanolic acid; betulinic acid; apigenin; luteolin.	Khazdair et al., 2018; Ghorani et al., 2022
Ziziphora clinopodioides Lam.	Aerial part; leaves	Cinaroside; apiin; diosmine; linarin, crisin; tanshinone IIA; caffeic acid; luteolin; quercetin; baicalein; kaempferol; curcumin; piceatannol, Exo-2-hydroxy cineole; limonene dioxide; blumenol C; sabinene; limonene; eucalyptol; menthone; isomenthone; menthol.	Chen et al., 2002; Shabbir et al., 2018; Ahmeda et al. 2020; Wu et al., 2020; Zhang et al., 2021; Ulikhanyan et al., 2022
Ziziphora tenuior L.	Aerial parts	p-menth-3-en-8-ol, isomenthone, 8-hydroxymenthone.	Nabiuni et al., 2015; Abu-Darwish et al., 2016; Kianpour et al., 2021

Aazza, S.; El-Guendouz, S.; Miguel, M.G. (2016). Antioxidant, Anti-inflammatory and anti-hyperglycaemic activities of essential oils from *Thymbra capitata, Thymus albicans, Thymus caespititius, Thymus carnosus, Thymus lotocephalus* and *Thymus mastichina* from Portugal. *Natural Product Communications,* 11(7). doi:10.1177/1934578X1601100739

Abbasloo, E.; Dehghan, F.; Khaksari, M.; Najafipour, H.; Vahidi, R.; Dabiri, S.; Sepehri, G.; Asadikaram, G. (2016). The anti-inflammatory properties of *Satureja khuzistanica* Jamzad essential oil attenuate the effects of traumatic brain injuries in rats. *Scientific Reports*, 6, 31866. doi.org/10.1038/srep31866

Abdolmaleki, A.; Rajabi, A.; Sanginabadi, F. (2013). Evaluation of the analgesic and anti-inflammatory effects of aqueous extract of peppermint (*Mentha piperita*). Scientific Journal of Kurdistan University of Medical Sciences 18(1), 67-74.

Abu-Darwish, M.S.; Cabral, C.; Ali; Z.; Wang, M.; Khan, S.I.; Jacob, M.R.; Efferth, T. (2020). *Salvia ceratophylla* L. from south of Jordan: New insights on chemical composition and biological activities. *Natural Products and Bioprospecting*, 10(5), 307-316. doi:10.1007/s13659-020-00259-9.

Acero, N.; Ortega, T.; Villagrasa, V.; Leon, G.; Muñoz-Mingarro, D.; Castillo, E.; González-Rosende, M.E.; Borrás, S.; Rios, J.L.; Bosch-Morell, F.; Martínez-Solís, I. (2023). Phytotherapeutic alternatives for neurodegenerative dementias: Scientific review, discussion and therapeutic proposal. *Phytotherapy Research*, 37, 1176–1211. doi:10.1002/ptr.7727.

Aćimović, M.; Šeregelj, V.; Simić, K.; Varga, A.; Pezo, L.; Vulić, J.; Čabarkapa, I. (2022). Chemical profile of *Nepeta cataria* l. var. *citriodora* Becker essential oil and in vitro evaluation of biological activities. *Acta Scientiarum Polonorum, Hortorum Cultus*, 21(4), 67–74. https://doi.org/10.24326/asphc.2022.4.7

Adriana Estrella, G.R.; María Eva, G. T.; Alberto, H. L.; María Guadalupe, V. D.; Azucena, C. V.; Sandra, O. S.; Noé, A. V.; Francisco Javier, L. M. (2021). Limonene from *Agastache mexicana* essential oil produces antinociceptive effects, gastrointestinal protection and improves experimental ulcerative colitis. *Journal of Ethnopharmacology*, 280. https://doi.org/10.1016/j.jep.2021.114462.

Afonso, A.F.; Pereira, O.R.; Fernandes, Â.S.F.; Calhelha, R.C.; Silva, A.M.S.; Ferreira, I.C.F.R.; Cardoso, S.M. (2019b). The health-benefits and phytochemical profile of *Salvia apiana* and *Salvia farinacea* var. *victoria* Blue decoctions. *Antioxidants* (Basel), 8(8):241. https://doi: 10.3390/antiox8080241. PMID: 31349575; PMCID: PMC6721217.

Afonso, A.F.; Pereira, O.R., Neto, R.T., Silva, A.M.S., Cardoso, S.M. (2017). Health-Promoting Effects of *Thymus herba-barona, Thymus pseudolanuginosus*, and *Thymus caespititius* Decoctions. *International Journal of Molecular Sciences,* 2017, 18, 1879. doi:10.3390/ijms18091879 .

Afonso, A. F., Pereira, O. R.; Fernandes, Â.; Calhelha, R. C.; Silva, A. M. S.; Ferreira, I. C. F.; Cardoso, S. M. (2019a). Phytochemical composition and bioactive effects of *Salvia africana*, *Salvia officinalis* ◎icterina◎and *Salvia mexicana* aqueous extracts. *Molecules*, 24(23) doi:10.3390/molecules24234327

Ahmad, F.; Kamaraj, B.; Mohamed, J.M.M.; El-Sagheer, A.M.; Emam, A.S.A. (2022). Ethnopharmacological and phytochemical study of major species from Labiatae family in Saudi Arabia: A Systematic Review. *International Journal of Applied Pharmaceutics*, 14 (ThematicSpecial Issue), pp. 44–57. DOI: https://dx.doi.org/10.22159/ijap.2022.v14ti.40

Ahmed, M.E.O.; Abdelgadir, A.A.; Ahmed, E.M. (2021). Traditional use of medicinal plants in Central Sudan Arabian. *Journal of Medicinal and Aromatic Plants*, 7(1), pp. 29–73. DOI: 10.48347/IMIST.PRSM/ajmap-v7i1.22273

Ahmeda, A.; Mahdavi, B.; Zaker, F.; Kaviani, S.; Hosseini, S.; Zangeneh, M. M.; Zangeneh, A.; Paydarfar, S.; Moradi, R. (2020). Chemical characterization and anti-hemolytic anemia potentials of tin nanoparticles synthesized by a green approach for bioremediation applications. *Applied Organometallic Chemistry*, 34(3) e5433. doi.org/10.1002/aoc.5433

Aihaiti, K.; Li, J.; Xu, N.-N.; Tang, D.; Aisa, H.A. (2023). Monoterpenoid derivatives from *Hyssopus cuspidatus* Boriss. and their bioactivities. *Fitoterapia*, 165, 105432. https://doi.org/10.1016/j.fitote.2023.105432

Akbarinia, A.; Sharifi-Ashabadi, A.; Sefidkon, F. (2008). Influence of harvest dynamics on herb and oil yield of *Thymus kotschyanus* and *Thymus daenensis* cultivated at two sites. *Acta Horticulturae*, 786, pp. 229–234. Doi: 10.17660/ActaHortic.2008.786.25

Akkol, E.K.; Göger, F.; Koşar, M.; Başer, K.H.C. (2008). Phenolic composition and biological activities of *Salvia halophila* and *Salvia virgata* from Turkey. *Food Chemistry*, *108*(3), 942–949. doi.org/10.1016/j.foodchem.2007.11.071

Akram, M.; Syed, A.S.; Kim, K.A.; Lee, J.S.; Chang, S.Y.; Kim, C.Y.; Bae, O.N. (2015). Heme oxygenase 1-mediated novel anti-inflammatory activities of *Salvia plebeia* and its active components. *Journal of Ethnopharmacology*, 174, 322–330. doi.org/10.1016/j.jep.2015.08.028

Aladedunye, F.A.; Okorie, D.A.; Ighodaro, O.M. (2008). Anti-inflammatory and antioxidant activities and constituents of *Platostoma africanum* P. Beauv. *Natural Product Research*, 22(12), 1067–1073. doi.org/10.1080/14786410802264004

Albano, S.M.; Miguel, M.G. (2011). Biological activities of extracts of plants grown in Portugal. *Industrial Crops and Products*, 33(2), 338–343. doi:10.1016/j.indcrop.2010.11.012

Algieri, F.; Rodriguez-Nogales, A.; Vezza, T.; ...Rodriguez-Cabezas, M.E.; Galvez, J. (2016). Anti-inflammatory activity of hydroalcoholic extracts of *Lavandula dentata* L. and *Lavandula stoechas* L. *Journal of Ethnopharmacology*, 190, pp. 142–158. https://doi.org/10.1016/j.jep.2016.05.063

Ali, T.; Javan, M.; Sonboli, A.; Semnanian, S. (2012). Antinociceptive and anti-inflammatory activities of the essential oil of *Nepeta crispa* Willd. in experimental rat models. *Natural Product Research*, *26*(16), 1529–1534. doi.org/10.1080/14786419.2011.565284

Ali-Shtayeh, M.S.; Abu-Zaitoun, S.Y.; Dudai, N.; Jamous, R.M. Downy lavender oil: a promising source of antimicrobial, antiobesity, and anti-Alzheimer's disease agents. *Evidence-Based Complementary and Alternative Medicine*, 2020, 5679408. doi: 10.1155/2020/5679408.

Aljohani, A.S.M.; Alhumaydhi, F.; Rauf, A.; Hamad, E.; Rashid, U. (2022). *In Vivo* and *In Vitro* Biological Evaluation and Molecular Docking Studies of Compounds Isolated from *Micromeria biflora* (Buch. Ham. ex D.Don) Benth. *Molecules*, 27(11), 3377. doi.org/10.3390/molecules27113377

Almeida-Bezerra, J.W.; Rodrigues, F.C.; Lima Bezerra, J.J.; Vieira Pinheiro, A.A.; Almeida De Menezes, S.; Tavares, A.B.; Costa, A.R.; Augusta De Sousa Fernandes, P.; Bezerra Da Silva, V.; Martins Da Costa, J.G.; Pereira Da Cruz, R.; Bezerra Morais-Braga, M.F.; Melo Coutinho, H.D.; Teixeira De Albergaria, E.; Meiado, M.V.; Siyadatpanah, A.; Kim, B.; Morais De Oliveira, A.F. (2022). Traditional Uses, Phytochemistry, and Bioactivities of *Mesosphaerum suaveolens* (L.) Kuntze. In *Evidence-based Complementary and Alternative Medicine*, 1-23 doi.org/10.1155/2022/3829180

Al-Mijalli, S. H.; Mrabti, N.N.; Ouassou, H.; Sheikh, R.A.; Assaggaf, H.; Bakrim, S.; Abdallah, E.M.; Alshahrani, M.M.; Al Awadh, A.A.; Lee, L.H.; AlDhaheri, Y.; Sahebkar, A.; Zengin, G.; Attar, A.A.; Bouyahya, A.; Mrabti, H.N. (2022). Chemical Composition and Antioxidant, Antimicrobial, and Anti-Inflammatory Properties of *Origanum compactum* Benth Essential Oils from Two Regions: In Vitro and In Vivo Evidence and In Silico. *Molecular Investigations. Molecules*, 27(21). https://doi.org/10.3390/molecules27217329

Alonazi M.A.; Jemel I.; Moubayed N.; Alwhibi M.; El-Sayed N.N.E.; Ben Bacha A. 2021. Evaluation of the *in vitro* anti-inflammatory and cytotoxic potential of ethanolic and aqueous extracts of *Origanum syriacum* and *Salvia lanigera* leaves. *Environmental Science and Pollution Research*, 28(16):19890-19900. https://doi: 10.1007/s11356-020-11961-z.

Al-Taweela, A.M.; Raish, M.; Perveen, S.; Fawzy, G.H.; Ahmad, A.; Ansari, M.A.; Mudassarg, S.; Ganaie, M.A. (2017). *Nepeta deflersiana* attenuates isoproterenol-induced myocardial injuries in rats: Possible involvement of oxidative stress, apoptosis, inflammation through nuclear factor (NF)-κB downregulation. *Phytomedicine*, 34, 67–75. doi: http://dx.doi.org/10.1016/j.phymed.2017.08.003

Álvarez-Santos, N.; Estrella-Parra, E.A.; Benítez-Flores, J. del C.; Serrano-Parrales, R.; Villamar-Duque, T.E.; Santiago-Santiago, M.A.; González-Valle, M. del R.; Avila-Acevedo, J.G.; García-Bores, A.M. (2022). *Asterohyptis stellulata*: Phytochemistry and wound healing activity. *Food Bioscience*, *50*, 102150. doi.org/10.1016/j.fbio.2022.102150

Alves-Silva, J.M.; Pedreiro, S.; Cavaleiro, C.; Figueirinha, A.; Salgueiro, L. (2023). Effect of *Thymbra capitata* (L.) Cav. on Inflammation, Senescence and Cell Migration. *Nutrients*, 15(8), 1930. https://doi.org/10.3390/nu15081930

Alves-Soares, R.; de Oliveira, H.D.; Campos, D.D.C.O.; Leal-Cardoso, J.H.; Coelho-de-Souza, A.N. (2022). The Essential Oil of *Hyptis crenata* Inhibits the Increase in Secretion of Inflammatory Mediators. *Plants*, 11(22), 3048. https://doi.org/10.3390/plants11223048

Al-Yousuf, M.H.; Bashir, A.K.; Ali, B.H.; Tanira, M.O.M.; Blunden, G. (2002). Some effects of *Salvia aegyptiaca* L. on the central nervous system in mice. Journal of Ethnopharmacology, 81, 121–127. www.elsevier.com/locate/jethpharm

Alzweiri, M.; Sarhan, A.A.; Mansi, K.; Hudaib, M.; Aburjai, T. (2011). Ethnopharmacological survey of medicinal herbs in Jordan, the Northern Badia region. *Journal of Ethnopharmacology*, 137(1), pp. 27–35. https://doi.org/10.1016/S0378-8741(02)00057-0

Amanlou, M.; Dadkhah, F.; Farsam, H.; Salehnia, A.; Dehpour, A. R. (2005). An anti-inflammatory and anti-nociceptive effect of hydroalcoholic extract of *Satureja khuzistanica* Jamzad extract. *Journal Pharmacology and Pharmaceutics Science*, *8*(1), 102–106. www.cspscanada.org

Aminian, A.R.; Mohebbati, R.; Boskabady, M.H. (2022). The Effect of *Ocimum basilicum* L. and Its Main Ingredients on Respiratory Disorders: An Experimental, Preclinical, and Clinical Review. *Frontiers in Pharmacology,* 12 805391. doi.org/10.3389/fphar.2021.805391

Amira, S.; Dade, M.; Schinella, G.; Rios, J.-L. (2012). Anti-inflammatory, anti-oxidant, and apoptotic activities of four plant species used in folk medicine in the Mediterranean basin T cell leukemia control via Ras-Raf pathway inhibition with peptides View project Mediterranean Plants for Skin Inflammation and Psoriasis View project. *Article in Pakistan Journal of Pharmaceutical Sciences*, www.researchgate.net/publication/51879323

Amirova, K.M.; Dimitrova, P.; Marchev, A.S.; Aneva, I.Y.; Georgiev, M.I. (2019). *Clinopodium vulgare* L. (wild basil) extract and its active constituents modulate cyclooxygenase-2 expression in neutrophils. *Food and Chemical Toxicology*, 124, pp. 1–9. https://doi.org/10.1016/j.fct.2018.11.054

An, H.J.; Jeong, H.J.; Um, J.Y.; Kim, H.M.; Hong, S.H. (2006). *Glechoma hederacea* inhibits inflammatory mediator release in IFN-γ and LPS-stimulated mouse peritoneal macrophages. *Journal of Ethnopharmacology*, *106*(3), 418–424. doi.org/10.1016/j.jep.2006.01.024

Andrade, J.M.; Custódio, L.; Romagnoli, A.; Reis, C.P.; Rodrigues, M.J.; Garcia, C.; Petruccioli, E.; Goletti, D.; Faustino, C.; Fimia, G.M.; Rijo, P. (2018). Antitubercular and anti-inflammatory properties screening of natural products from *Plectranthus* species. *Future medicinal chemistry*, 10(14), 1677–1691. https://doi.org/10.4155/fmc-2018-0043

Andreyeva, A.A.; Sivtseva, V.V.; Pashayeva, A.T.; Chirikova, N.D.; Okhlopkova, Z.M.; Kim, S.W.; Huseynova, I.M.; Zulfugarov, I.S. (2020). Dragonhead as a model plant for biotechnology of biologically active compounds. *Azerbaijan Pharmaceutical and Pharmacotherapy Journal*, 1, 31-35.

Anjos, K.S.D.; Araújo-Filho, H.G.; Duarte, M.C.; Costa, V.C.O.; Tavares, J.F.; Silva, M.S.; Almeida, J.R.G.S.; Souza, N.A.C.; Rolim, L.A.; Menezes, I.R.A.; Coutinho, H.D.M., Quintans, J.S. S.; Quintans-Júnior, L.J. (2017). HPLC-dad analysis, antinociceptive and anti-inflammatory properties of the ethanolic extract of *Hyptis umbrosa* in mice. *EXCLI Journal*, *16*, 14–24. doi.org/10.17179/excli2016-698

Antão, A.R.; Bangay, G.; Domínguez-Martín, E.M.; Díaz-Lanza, A.M.; Ríjo, P. (2021). *Plectranthus ecklonii* Benth.: A comprehensive review into its phytochemistry and exerted biological activities. *Frontiers in Pharmacology*, 12, 768268. doi: 10.3389/fphar.2021.768268.

Arenas-Chavez, C.A.; Wiche-Salinas, T.; Valencia-Mercado, I.; Calle-Valdez, R.; Vera-Gonzales, C.; Malaga-Contreras, S.; Huanqui-Guerra, C. Efecto antiiflamatorio de la fracción flavonoide de *Lepechinia meyenii* (Walp.) Epling (*Salvia*) sobre leucocitos de pacientes con artritis reumatoide. *Revista Peruana de Medicina Experimental y Salud Pública*, 35(1), 55-61 doi: 10.17843/rpmesp.2018.351.3600.

Asadi-Samani,M., Rafieian-Kopaei, M., Lorigooini. and Shirzad, H. (2018). A screening to determine total phenol and flavonoid content of some Iran's medicinal plants grown in Chaharmahal va Bakhtyari province. *Indian Journal of Natural Products and Resources,* 9(4): 296-302.

Asadollahi, M.; Firuzi, O.; Heidary Jamebozorgi, F.; Alizadeh, M.; Jassbi, A. R. (2019). Ethnopharmacological studies, chemical composition, antibacterial and cytotoxic activities of essential oils of eleven *Salvia* in Iran. *Journal of Herbal Medicine*, 17-18 doi:10.1016/j.hermed.2018.11.006

Azevedo, M.-M.; Pinheiro, C.; Dias, A.C.P.; Pinto-Ribeiro, F.; Baltazar, F. (2015). Impact of an educational hands-on project on the antimicrobial, antitumor and anti-inflammatory properties of plants on portuguese students' awareness, knowledge, and competences. *International Journal of Environmental Research and Public Health*, 12(3), pp. 2437–2453. https://doi.org/10.3390/ijerph120302437

Bacalbasa, N.; Balescu, I.; Stoica, C.; Pop, L.; Varlas, V.; Martac, C.; Voichitoiu, A.; Gaspar, B. (2022). The anti-inflammatory, anti-infectious and anti-cancerous effects of *Thymus vulgaris*. *Romanian Journal of Medical Practice*, *17*, 21–23. doi.org/10.37897/RJMP. 2022.S3.6

Bakhtiarian, A.; Aarabi Moghaddam, F.; Zamani, M.M.; ...Farahanikia, B.; Khanavi, M. (2011). Anti-inflammatory effect of *Thymus kotschyanus* Boiss. Hohen. extract on rats hind paw edema induced by carrageenan. *Journal of Medicinal Plants*, 10(37), pp. 25–32.

Banerjee, P.; Kumar, T.; Sarangi, S.C.; Meetei, U.D.; Devi, A.S.; Kumar, R. (2021). Anti-inflammatory potential of aqueous extract of *Elsholtzia stachyodes* on experimental models of inflammation in rats. *Journal of Natural Science, Biology and Medicine*, *12*(1), 103–108. doi.org/10.4103/jnsbm.JNSBM_5_20

Banno, N.; Akihisa, T.; Tokuda, H.; Yasukawa, K.; Higashihara, H.; Ukiya, M.; Watanabe, K.; Kimura, Y.; Hasegawa, J.-I.; Nishino, H. (2004). Triterpene Acids from the Leaves of *Perilla frutescens* and Their Anti-inflammatory and Antitumor-promoting Effects. *Bioscience, Biotechnology and Biochemistry*, *68*(1), 85–90.

Batista, D.; Falé, P.L.; Serralheiro, M.L.; Araújo, M.E., Dias; C., Branco, I.; Grosso, C.; Coelho, J.; Palavra, A.; Madeira, P.J.A.; Martins, A.; Rauter, A.P. (2017). Phytochemical Characterization and Biological Evaluation of the Aqueous and Supercritical Fluid Extracts from *Salvia sclareoides* Brot. *Open Chemistry*, *15*(1), 82–91. doi.org/10.1515/chem-2017-0011

Bayat, M.; Kalantar, K.; Amirghofran, Z. (2019). Inhibition of interferon-γ production and T-bet expression by menthol treatment of human peripheral blood mononuclear cells. *Immunopharmacology and Immunotoxicology*, *41*(2), 267–276. doi.org/10.1080/08923973.2019.1588294

Becker, K.; Schwaiger, S.; Waltenberger, B.; Fuchs, D.; Pezzei, C. K.; Schennach, H.; Stuppner, H.; Gostner, J. M. (2018). Immunomodulatory Effects of Diterpene Quinone Derivatives from the Roots of *Horminum pyrenaicum* in Human PBMC. Oxidative Medicine and Cellular Longevity, 2018. doi.org/10.1155/2018/2980295

Belozerova, O.A.; Osmakov, D.I.; Vladimirov, A.; Koshelev, S.G.; Chugunov, A.O.; Andreev, Y.A.; Palikov, V.A.; Palikova, Y.A.; Shaykhutdinova, E.R.; Gvozd, A.N.; et al. (2020). Sevanol and Its Analogues: Chemical Synthesis, Biological Effects and Molecular Docking. *Pharmaceuticals*, 13, 163. https://doi.org/10.3390/ph13080163

Benrezzouk, R.; Terencio, M.C.; Ferrandiz, M.L.; Hernandez-Perez, M.; Rabanal, R.; Alcaraz, M. J. (2001). Inhibition of 5-lipoxygenase activity by the natural anti-inflammatory compound aethiopinone. *Inflammation Research*, 50(2), 96-101. doi:10.1007/s000110050730.

Beserra-Filho, J.I.A.; de Macêdo, A.M.; Leão, A.H.F.F.; Bispo, J.M.M.; Santos, J.R.; de Oliveira-Melo, A.J.; Passos Menezes, P.D.; Duarte, M.C.; de Souza Araújo, A.A., Silva, R.H.; Quintans-Júnior, L.J.; Ribeiro, A.M. (2019). *Eplingiella fruticosa* leaf essential oil complexed with β-cyclodextrin produces a superior neuroprotective and behavioral profile in a mice model of Parkinson's disease. *Food and Chemical Toxicology*, 124, 17–29. https://doi.org/10.1016/j.fct.2018.11.056.

Bhardwaj, S.; Bhatia, S. (2020). Development and characterization of niosomal gel system using *Lallementia royaleana* Benth. mucilage for the treatment of rheumatoid arthritis. *Iranian Journal of Pharmaceutical Research*, IJPR, 19(3), 465–482. doi.org/10.22037/ijpr.2020.112887.14003

Bian, J.; Wang, K.; Wang, Q,.; Wang, P.; Wang, T.; Shi, W.; Ruan, Q. (2020). *Dracocephalum heterophyllum* (DH) exhibits potent anti-proliferative effects on autoreactive CD4+ T cells and ameliorates the development of experimental autoimmune uveitis. *Frontiers in Immunology*, 11:575669. doi: 10.3389/fimmu.2020.575669

Bian, M.; Zhang, Y.; Du, X.; Xu, J.; Cui, J.; Gu, J.; Zhu, W.; Zhang, T.; Chen, Y. (2017). Apigenin-7-diglucuronide protects retinas against bright light-induced photoreceptor degeneration through the inhibition of retinal oxidative stress and inflammation. *Brain Research*, *1663*, 141–150. doi.org/10.1016/j.brainres.2017.03.019

Bonesi, M.; Loizzo, M.R.; Acquaviva, R.; Malfa, G.A.; Aiello, F.; Tundis, R. (2017). Anti-inflammatory and antioxidant agents from *Salvia* genus (Lamiaceae): An assessment of the current state of knowledge. *Anti-Inflammatory and Anti-Allergy Agents in Medicinal Chemistry*, 16(2), 70-86. doi:10.2174/1871523016666170502121419

Bonito, M.C.; Cicala, C.; Marcotullio, M.C.; Maione, F.; Mascolo, N. (2011). Biological activity of bicyclic and tricyclic diterpenoids from *Salvia* species of immediate pharmacological and pharmaceutical interest. *Natural Product Communications*, 6(8), 1205-1215. doi:10.1177/1934578x1100600839

Borges, R.S.; Ortiz, B.L.S.; Pereira, A.C.M.; Keita, H.; Carvalho, J.C.T. (2019). *Rosmarinus officinalis* essential oil: A review of its phytochemistry, anti-inflammatory activity, and mechanisms of action involved. J*ournal of Ethnopharmacology*, 229, 29–45. Elsevier Ireland Ltd. https://doi.org/10.1016/j.jep.2018.09.038

Botrel, P.P.; Pinto, J.E.B.P.; Ferraz, V.; Bertolucci, S.K.V.; Figueiredo, F.C. (2010). Teor e composição química do óleo essencial de *Hyptis marrubioides* Epl., Lamiaceae em função da sazonalidade. *Acta Scientiarum - Agronomy*, *32*(3), 533–538. doi.org/10.4025/actasciagron.v32i3.3415

Boukhary, R.; Raafat, K.; Ghoneim, A.I.; Aboul-Ela, M.; El-Lakany, A. (2016). Anti-Inflammatory and Antioxidant Activities of *Salvia fruticosa*: An HPLC Determination of Phenolic Contents. *Evidence-Based Complementary and Alternative Medicine*, *2016*. doi.org/10.1155/2016/7178105

Bounihi, A.; Hajjaj, G.; Alnamer, R.; Cherrah, Y.; Zellou, A. (2013). In vivo potential anti-inflammatory activity of *Melissa officinalis* l. essential oil. *Advances in Pharmacological Sciences*, *2013*. doi.org/10.1155/2013/101759

Bouyahya, A.; Chamkhi, I.; Menyiy, N.E.; Simal-Gandara, J.; El Omari, N. (2023). Traditional use, phytochemistry, toxicology, and pharmacological properties of *Lavandula dentata* L.,: A comprehensive review. *South African Journal of Botany*, 154, pp. 67–87.

Brahmi, F.; Nury, T.; Debbabi, M.; Hadj-Ahmed, S.; Zarrouk, A.; Prost, M.; Madani, K.; Boulekbache-Makhlouf, L.; Lizard, G. (2018). Evaluation of antioxidant, anti-inflammatory and cytoprotective properties of ethanolic mint extracts from Algeria on 7-ketocholesterol-treated murine RAW 264.7 macrophages. *Antioxidants*, *7*(12), 1-15. doi.org/10.3390/antiox7120184

Burk, D.R.; Senechal-Willis, P.; Lopez, L.C.; Hogue, B.G.; Daskalova, S.M. (2009). Suppression of lipopolysaccharide-induced inflammatory responses in RAW 264.7 murine macrophages by aqueous extract of *Clinopodium vulgare* L. (Lamiaceae). *Journal of Ethnopharmacology*, 2009, 126(3), pp. 397–405.

Cai, X.; Mao, Y.; Shen, X.; ...He, J.; Zhang, M. (2023). The extract from *Hyssopus cuspidatus* Boriss. Prevents bronchial airway remodeling by inhibiting mouse bronchial wall thickening and hASMC proliferation and migration. *Journal of Ethnopharmacology*, 2023, 303, 116047.

Calvo, M.I.; Cavero, R.Y. (2015). Medicinal plants used for neurological and mental disorders in Navarra and their validation from official sources. *Journal of Ethnopharmacology*, 169, pp. 263–268.

Cam M.E.; Hazar-Yavuz A.N.; Yildiz S.; Ertas B.; Ayaz Adakul B.; Taskin T.; Alan S.; Kabasakal L. (2019). The methanolic extract of *Thymus praecox* subsp. *skorpilii* var. *skorpilii* restores glucose homeostasis, ameliorates insulin resistance and improves pancreatic β-cell function on streptozotocin/nicotinamide-induced type 2 diabetic rats. *Journal of Ethnopharmacology*, 231 (2019) 29–38. https://doi.org/10.1016/j.jep.2018.10.028.

Carbone C.; Martins-Gomes C.; Caddeo C.; Silva A.M.; Musumeci T.; Pignatello R.; Puglisi G.; Souto E.B. (2018). Mediterranean essential oils as precious matrix components and active T ingredients of lipid nanoparticles. *International Journal of Pharmaceutics*, 548 (2018) 217–226. doi.org/10.1016/j.ijpharm.2018.06.064

Cardia, G.F.E.; Silva-Filho, S.E.; Silva, E.L.; Uchida, N.S.; Cavalcante, H.A.O.; Cassarotti, L.L.; Salvadego, V.E.C.; Spironello, R.A.; Bersani-Amado, C.A.; Cuman, R.K.N. (2018). Effect of Lavender (*Lavandula angustifolia*) Essential Oil on Acute Inflammatory Response. *Evidence-Based Complementary and Alternative Medicine*, *2018*. doi.org/10.1155/2018/1413940.

Cariddi, L.N.; González-Pereyra, M.L.; Gambero, M.L.; Demo, M.S.; Isola, M.C.; Franzoni, L.; Sabini, L.I.; Maldonado, A.M. (2006). *Minthostachys verticillata* (Griseb.) Epling: A South American plant with antiinflammatory effect *in vitro*. *Allergy and Clinical Immunology International*, 18(6), 234–241. https://doi.org/10.1027/0838-1925.18.6.234.

Carrasco, A.; Martinez-Gutierrez, R.; Tomas, V.; Tudela, J. (2015). *Lavandula angustifolia* and *Lavandula latifolia* Essential Oils from Spain: Aromatic Profile and Bioactivities. *Planta Medica*, 82(1-2), pp. 163–170.

Carrasco, A.; Perez, E.; Cutillas, A.; Martinez-Gutierrez, R.; Tomas, V.; Tudela, J. (2016). *Origanum vulgare* and T*hymbra capitata* essential oils from spain: Determination of aromatic profile and bioactivities. *Natural Product Communications*, 11(1), 113-120. doi:10.1177/1934578x1601100133

Castro, J.P.; Franco, L.A.; Diaz, F. (2021). Anti-inflammatory screening of plant species from the Colombian Caribbean Coast. *Journal of Applied Pharmaceutical Science*, 11(4), 106-117. doi: 10.7324/JAPS.2021.110413.

Ceschel, G.C.; Maffei, P.; Moretti, M.D.L.; Demontis, S.; Peana, A.T. (2000). *In vitro* permeation through porcine buccal mucosa of *Salvia desoleana* atzei and picci essential oil from topical formulations. *International Journal of Pharmaceutics*, 195(1-2), 171-177. doi:10.1016/S0378-5173(99)00381-6

Chan, H.H.; Hwang, T.L.; Su, C.R.; Reddy, M.V.B.; Wu, T.S. (2011). Anti-inflammatory, anticholinesterase and antioxidative constituents from the roots and the leaves of *Salvia nipponica* Miq. var. *formosana*. *Phytomedicine*, *18*(2–3), 148–150. doi.org/10.1016/j.phymed.2010.06.017

Chang, C.T.; Soo, W.N.; Chen, Y.H.; Shyur, L.F. (2019). Essential Oil of *Mentha aquatica* var. *kenting* water mint suppresses two-stage skin carcinogenesis accelerated by BRAF inhibitor vemurafenib. *Molecules*, 24(12). https://doi.org/10.3390/molecules24122344

Chen, J.; Wang, J.B.; Yu, C.H.; Chen, L.Q.; Xu, P.; Yu, W.Y. (2013). Total flavonoids of *Mosla scabra* leaves attenuates lipopolysaccharide-induced acute lung injury via down-regulation of inflammatory signaling in mice. *Journal of ethnopharmacology*, 148(3), 835–841. doi.org/10.1016/j.jep.2013.05.020

Chen, S.; Fang, A.; Zhong, Y.; Tang, J. (2022). *Ziziphora clinopodioides* Lam leaf aqueous extract mediated novel green synthesis of iron nanoparticles and its anti-hemolytic anemia potential: A chemobiological study. *Arabian Journal of Chemistry*, 15(3), 103561. doi.org/10.1016/j.arabjc.2021.103561

Chen, S.; Lin, Z.; Qin, G.; Sun, H.; Chen, Y. (1999). Diterpenoids from *Isodon leucophyllus*. *Planta Medica*, *65*(5), 472–474.

Chen, X.; Zhang, L.; Qian, C.; Du, Z.; Xu, P.; Xiang, Z. (2020). Chemical compositions of essential oil extracted from *Lavandula angustifolia* and its prevention of TPA-induced inflammation. *Microchemical Journal*, *153*. doi.org/10.1016/j.microc.2019.104458

Cheng, E.; Chi, J.; Li, Y.X.; Zhang, W.J.; Huang, N.; Wang, Z.M.; Dai, L.P.; Xu, E.P. (2022). Diverse ent-kaurane diterpenoids from *Isodon henryi*. *Tetrahedron Letters*, *108*. doi.org/10.1016/j.tetlet.2022.154119

Choia, H.; Kim, T.; Kim, S.-H.; Kim, J. (2016). Anti-allergic inflammatory triterpenoids isolated from the spikes of *Prunella vulgaris*. *Natural Product Communications*, *11*(1), 2–31.

Chou, S.; Lai, C.; Lai, C.; Chao, W. (2018). Chemical composition, antioxidant, anti-melanogenic and anti-inflammatory activities o *Glechoma hederacea* (Lamiaceae) essential oil. *Industrial Crops and Products* 122, 675-685. doi.org/10.1016/j.indcrop.2018.06.032.

Choudhary, M.I.; Siddiqui, Z.A.; Nawaz, S.A.; Atta-ur-Rahman. (2006). Microbial transformation and butyrylcholinesterase inhibitory activity of (-)-caryophyllene oxide and its derivatives. *Journal of Natural Products*, 69(10), pp. 1429–1434.

Cigremis, Y.; Ulukanli, Z.; Ilcim, A.; Akgoz, M. (2010). *In vitro* antioxidant and antimicrobial assays of acetone extracts from *Nepeta meyeri* Benth. *European Review for Medical and Pharmacological Sciences*, 14, 661–668.

Coelho, M.N.; Soares, P.A.G.; Frattani, F.S.; Camargo, L.M.M.; Tovar, A.M.F.; de Aguiar P.F.; Zingali R.B.; Mourão P.A.S.; Costa S.S. Polysaccharide composition of an anticoagulant fraction from the aqueous extract of *Marsypianthes chamaedrys* (Lamiaceae). *International Journal of Biological Macromolecules,* 145 (2020) 668–681. doi.org/10.1016/j.ijbiomac.2019.12.176 0141-8130/.

Coelho-De-souza, A.N.; Alves-Soares, R.; Oliveira, H.D.; Guimarães-Pereira, J.; Leal-Cardoso, J.H. (2021). The essential oil of *Hyptis crenata* Pohl ex Benth. Presents an antiedematogenic effect in mice. *Brazilian Journal of Medical and Biological Research*, 54(3), pp. 1–9, e9422

Contreras, M.D.M.; Algieri, F.; Rodriguez-Nogales, A.; Gálvez, J.; Segura-Carretero, A. (2018). Phytochemical profiling of anti-inflammatory *Lavandula* extracts via RP–HPLC–DAD–QTOF–MS and –MS/MS: Assessment of their qualitative and quantitative differences. *Electrophoresis*, 2018, 39(9-10), pp. 1284–1293.

Côté, H.; Pichette, A.; St-Gelais, A.; Legault, J. (2021). The biological activity of *Monarda didyma* L. essential oil and its effect as a diet supplement in mice and broiler chicken. *Molecules*, 26, 3368. doi: https://doi.org/10.3390/molecules26113368.

Dadkhah, A.; Fatemi, F.; Rasooli, A.; Malayeri, M.R.M., Torabi, F. (2018). Assessing the effect of *Mentha longifolia* essential oils on cox-2 expression in animal model of sepsis induced by caecal ligation and puncture. *Pharmaceutical Biology*, *56*(1), 495–504. doi.org/10.1080/13880209.2018.1510972

Dal Piaz F.; Imparato S.; Lepore L.; Bader A.; De Tommasi N. 2010. A fast and efficient LC-MS/MS method for detection, identification and quantitative analysis of bioactive sesterterpenes in *Salvia dominica* crude extracts. *Journal Pharmaceutical Biomedical Analysis,* 51(1):70-77. https://10.1016/j.jpba.2009.08.006.

Darabad, A.; Rahimi, M.; Rafati, H. (2022). Continuous hydrodistillation-ultrasonication flow batch-mode operation system to enhance essential oil extraction from *Satureja khuzistanica* and evaluation of in vitro anti-inflammatory activity. *Industrial Crops and Products*, 188(A), 115540. doi.org/10.1016/j.indcrop.2022.115540

De Felice A.; Bader A.; Leone A.; Sosa S.; Loggia R.D.; Tubaro A.; De Tommasi N. 2006. New polyhydroxylated triterpenes and anti-inflammatory activity of *Salvia hierosolymitana*. Planta Medica 72(7):643-649. https://doi: 10.1055/s-2006-931573

De Jesus, N.Z.T.; Lima, J.C.D.S.; Da Silva, R.M.; Espinosa, M.M.; Martins, D.T.D.O. (2009). Ethnobotanical survey of plants popularly used as anti-ulcer and anti-inflammatory in Pirizal, Nossa Senhora do Livramento, MT, Brazil | Levantamento etnobotânico de plantas popularmente utilizadas como antiúlceras e antiinflamatórias pela comunidade de Pirizal, Nossa Senhora do Livramento-MT, Brasil. *Revista Brasileira de Farmacognosia*, 19(1 A), pp. 130–139.

De Lima, M.N.N.; Guimarães, B.A.; de Castro, A.L.S.; ...Maia, J.G.S.; Figueiredo, P.L.B. (2023). Chemical composition and antinociceptive and anti-inflammatory activity of the essential oil of *Hyptis crenata* Pohl ex Benth. from the Brazilian Amazon. *Journal of Ethnopharmacology*, 300, 115720.

De Lima, V.T.; Vieira, M.C.; Kassuya, C.A.L.; Cardoso, C.A.L.; Alves, J.M.; Foglio, M.A.; De Carvalho, J.E.; Formagio, A.S.N. (2014). Chemical composition and free radical-scavenging, anticancer and anti-inflammatory activities of the essential oil from *Ocimum kilimandscharicum*. *Phytomedicine*, 21(11), 1298–1302. https://doi.org/10.1016/j.phymed.2014.07.004.

De Macedo, L.M.; Dos Santos, É.M.; Militão, L.; Tundisi, L.L.; Ataide, J.A.; Souto, E.B.; Mazzola, P.G. (2020). Rosemary (*Rosmarinus officinalis* L., syn. *salvia rosmarinus* Spenn.) and its topical applications: A review. *Plants*, 9, 5. MDPI AG. https://doi.org/10.3390/plants9050651.

De Oliveira Ferreira, E.; Soares Dias, M.Y.; Rocha de Lima, N.M.; Tavares Neves, K.R.; Santos do Carmo, M.R.; Viana Lima, F.A.; Aragão Fonteles, A.; Fontenele Menezes, A.P.; Matos de Andrade, G. (2016). Neuroinflammatory response to experimental stroke is inhibited by eriodictyol. *Behavioural Brain Research*, 312, 321-332. http://dx.doi.org/10.1016/j.bbr.2016.06.046.

De Pinho, J.P.M.; Silva, A.S.B.; Pinheiro, B.G.; ...Da Cunha Sousa, P.J.; Magalhães, P.J.C. (2012). Antinociceptive and antispasmodic effects of the essential oil of *Ocimum micranthum*: Potential anti-inflammatory properties. *Planta Medica*, 78(7), pp. 681–685.

De Sousa Araújo, T.A.; Alencar, N.L.; de Amorim, E.L.C.; de Albuquerque, U.P. (2008). A new approach to study medicinal plants with tannins and flavonoids contents from the local knowledge. *Journal of Ethnopharmacology*, 120(1), pp. 72–80.

Demirci, F.; Karaca, N.; Tekin, M.; Demirci, B. (2018). Anti-inflammatory and antibacterial evaluation of *Thymus sipyleus* Boiss. subsp. *sipyleus* var. *sipyleus* essential oil against rhinosinusitis pathogens. *Microbial Pathogenesis*, 122, pp. 117–121.

Demirci, F.; Karadağ, A.E.; Biltekin, S.N.; Demirci, B. (2021). In Vitro ACE2 and 5-LOX Enzyme Inhibition by Menthol and Three Different Mint Essential Oils. *Natural Product Communications*, *16*(11). doi.org/10.1177/1934578X211055014

Deng, Y.; Hua, J.; Wang, W.; Zhan, Z.; Wang, A.; Luo, S. (2018). Cytotoxic terpenoids from the roots of *Dracocephalum taliense*. *Molecules*, *23*(1). doi.org/10.3390/molecules23010057

Dobrev, H.P. (2021). Treatment of numerous hand warts with *Clinopodium vulgare* tea. *Wiener Medizinische Wochenschrift*, 171(3-4), pp. 82–83.

Donatello, N.N.; Emer, A.A.; Salm, D.C.; Ludtke, D.D.; Bordignon, S.A.S.R.; Ferreira, J.K.; Salgado, A.S.I.; Venzke, D.; Bretanha, L.C.; Micke, G.A.; Martins, D.F. (2020). *Lavandula angustifolia* essential oil inhalation reduces mechanical hyperalgesia in a model of inflammatory and neuropathic pain: The involvement of opioid and cannabinoid receptors. *Journal of Neuroimmunology*, *340*. doi.org/10.1016/j.jneuroim.2020.577145

Dos Santos, E.; Leitão, M.M.; Aguero Ito, C.N.; Silva-Filho, S.E.; Arena, A.C.; Silva-Comar, F.M. de S.; Nakamura Cuman, R.K.; Oliveira, R.J.; Nazari Formagio, A.S. Leite Kassuya, C. A. (2021). Analgesic and anti-inflammatory articular effects of essential oil and camphor isolated from *Ocimum kilimandscharicum* Gürke leaves. *Journal of Ethnopharmacology*, 269. https://doi.org/10.1016/j.jep.2020.113697

Draginic, N.; Andjic, M.; Jeremic, J.; Zivkovic, V.; Kocovic, A.; Tomovic, M.; Bozin, B.; Kladar, N.; Bolevich, S.; Jakovljevic, V.; Milosavljevic, I. (2022). Anti-inflammatory and Antioxidant Effects of *Melissa officinalis* Extracts: A Comparative Study. *Iranian Journal of Pharmaceutical Research*, *21*(1). doi.org/10.5812/ijpr-126561

Du, Y.; Jin, Y.; Liu, P.; Shi, X.; Xu, H.; Wang, Q.; Zhang, L. (2013). Quantitative analysis of ten diterpenoids in rat bile after oral administration of *Isodon rubescens* extract by high performance liquid chromatography-electrospray ionization tandem mass spectrometry. *Journal of Liquid Chromatography and Related Technologies*, *36*(9), 1264–1279. doi.org/10.1080/10826076.2012.686137

Dubinnyi, M.A.; Osmakov, D.I.; Koshelev, S.G.; ...Arseniev, A.S.; Grishin, E.V. 2012. Lignan from thyme possesses inhibitory effects on ASIC3 channel current. *Journal of Biological Chemistry*, 287(39), pp. 32993–33000.

Eidi, A.; Eidi, T.M.M.; Mozaffarian, V.; Rustaiyan A.; Mazooji A.S.; Khaboori Z.; Nabiuni F. 2011. Antinociceptive and Anti-inflammatory Effects of Ethanolic Extract of *Salvia syriaca* L. in Mice. *International Journal of Pharmacology*, 7(3): 394-399, 2011 DOI: 10.3923/p.2011.394.399

El Kharraf, S.; El-Guendouz, S.; Farah, A.; Bennani, B.; Mateus, M.C.; El Hadrami, E.M.; Miguel, M.G. (2021). Hydrodistillation and simultaneous hydrodistillation-steam distillation of *Rosmarinus officinalis* and *Origanum compactum*: Antioxidant, anti-inflammatory, and antibacterial effect of the essential oils. , 168. https://doi.org/10.1016/j.indcrop.2021.113591.

El Menyiy, N.; Mrabti, H.N.; el Omari, N.; Bakili, A.E.I.; Bakrim, S.; Mekkaoui, M.; Balahbib, A.; Amiri-Ardekani, E.; Ullah, R.; Alqahtani, A.S.; Shahat, A.A.; Bouyahya, A. (2022). Medicinal Uses, Phytochemistry, Pharmacology, and Toxicology of *Mentha spicata*. In *Evidence-based Complementary and Alternative Medicine* (Vol. 2022). Hindawi Limited. doi.org/10.1155/2022/7990508

El Sawi, S.A.; Sleem, A.A.; Merghany, R.M. 2021. *In vivo* antioxidant, anti-inflammatory and anti-hyperglycemic effects of *Salvia splendens* aerial parts in male albino rat models. *Journal of Herbs, Spices Medicinal Plants*, 27(2): 149-160. https://doi.org/10.1080/10496475.2021.1891173.

El Yaagoubi, M.; Mechqoq, H.; El Hamdaoui, A.; ...Msanda, F.; El Aouad, N. (2021). A review on Moroccan *Thymus* species: Traditional uses, essential oils chemical composition and biological effects. *Journal of Ethnopharmacology*, 278, 114205.

El-Askary, H.; El-Kashoury, E.; Kandil, Z.; Salem, M.; Sleem, A. (2012). Chemical composition and biological activities of the essential oil of *Mentha suaveolens* Ehrh. Z. Naturforsch, 67(3), 571–579.

Esquivel, B.; Bustos-Brito, C.; Sánchez-Castellanos, M.; Nieto-Camacho, A.; Ramírez-Apan, T.; Joseph-Nathan, P.; Quijano, L. (2017). Structure, absolute configuration, and antiproliferative activity of Abietane and Icetexane diterpenoids from *Salvia ballotiflora*. *Molecules*, 22(10), 1690. doi: 10.3390/molecules22101690.

Estakhr, J.; Javdan, N. (2011). Evaluation of anti-inflammatory effects of ethanolic extract of *Salvia hypoleuca*. *Pharmacologyonline*, 2, 879-885.

Estakhr, J.; Javdan, N. (2011a). *Salvia hypoleuca*; a new source of compounds which are useful for treatment of ocular inflammation. *Pharmacologyonline*, 3, 338-346. Retrieved from www.scopus.com

Fawole, O.A.; Amoo, S.O.; Ndhlala, A.R.; Light, M.E.; Finnie, J. F.; van Staden, J. (2010). Anti-inflammatory, anticholinesterase, antioxidant, and phytochemical properties of medicinal plants used for pain-related ailments in South Africa. *Journal of Ethnopharmacology*, *127*(2), 235–241. doi.org/10.1016/j.jep.2009.11.015

Feng, J.; Liu, L.; Yao, F.; Zhou, D.; He, Y.; Wang, J. (2021). The protective effect of tanshinone IIA on endothelial cells: a generalist among clinical therapeutics. *Expert Review of Clinical Pharmacology,* 14(2), 239–248. doi.org/10.1080/17512433.2021.1878877

Feng, X.; Wang, X.; Liu, Y.; Di, X. (2015). Linarin Inhibits the Acetylcholinesterase Activity In-vitro and Ex-vivo. In *Shaheed Beheshti University of Medical Sciences and Health Services Iranian Journal of Pharmaceutical Research,* 14(3), 949-954.

Fiuza, T.; Rezende M.; Sabóia-Morais, S.; Tresvenzol, L.; Ferreira H.; Paula, J. 2008. Estudo das folhas e caule de *Hyptidendron canum* (Pohl ex Benth.) Harley, Lamiaceae. Revista Brasileira de Farmacognosia Brazilian Journal of Pharmacognosy 20(2): 192-200.

Fraternale, D.; Dufat, H.; Albertini, M.C.; Bouzidi, C.; D'Adderio, R.; Coppari, S.; Di Giacomo, B.; Melandri, D.; Ramakrishna, S.; Colomba, M. (2022). Chemical composition, antioxidant and anti-inflammatory properties of *Monarda didyma* L. essential oil. *PeerJ*, 10, e14433. doi: 10.7717/peerj.14433.

Fushiya, S.; Batkhuu, J.; Takano, F.; Hayasaka, H.; Ohba, K.; Yoshizaki, F.; Dashi, T.; Batsuren, D.; Sanchir, C. (2000). Effects of Mongolian plants on nitric oxide production in activated macrophages. *Natural Medicines*, *54*(6), 352–357.

Galasso, S.; Pacifico, S.; Kretschmer, N.; Pan, S.; Marciano, S.; Piccolella, S.; Monaco, P.; Bauer, R. (2014). Influence of seasonal variation on *Thymus longicaulis* C. Presl chemical composition and its antioxidant and anti-inflammatory properties. *Phytochemistry,* 107: 80–90. http://dx.doi.org/10.1016/j.phytochem.2014.08.015

García-Díaz, J.A.; Navarrete-Vázquez, G.; García-Jiménez, S.; Hidalgo-Figueroa, S.; Almanza-Pérez, J.C.; Alarcón-Aguilar, F.J.; Gómez-Zamudio, J.; Cruz, M.; Ibarra-Barajas, M.; Estrada-Soto, S. (2016). Antidiabetic, antihyperlipidemic and anti-inflammatory effects of tilianin in streptozotocin-nicotinamide diabetic rats. *Biomedicine Pharmacotherapy*, 83, 667–675. doi.org/10.1016/j.biopha.2016.07.023

Garzoli, S.; Alarcón-Zapata, P.; Seitimova, G.; ...Pezzani, R.; Vitalini, S. (2022). Natural essential oils as a new therapeutic tool in colorectal cancer. *Cancer Cell International*, 22(1), 407.

Georgiev, Y.N.; Paulsen, B. S.; Kiyohara, H.; Ciz, M.; Ognyanov, M.H.; Vasicek, O.; Rise, F.; Denev, P.N.; Yamada, H.; Lojek, A.; Kussovski, V.; Barsett, H.; Krastanov, A.I., Yanakieva, I. Z.; Kratchanova, M.G. (2017). The common lavender (*Lavandula angustifolia* Mill.) pectic polysaccharides modulate phagocytic leukocytes and intestinal Peyer's patch cells. *Carbohydrate Polymers*, *174*, 948–959. doi.org/10.1016/j.carbpol.2017.07.011

Ghorani, V.; Beigoli, S.; Khazdair, M.R., Boskabady, M.H. 2022. The effect of *Zataria multiflora* on respiratory allergic and immunologic disorders, experimental and clinical evidence: A comprehensive review. In *Phytotherapy Research* 36(3), 1135–1155. doi.org/10.1002/ptr.7382

Ghuman, S.; Ncube, B.; Finnie, J.F.; Mcgaw, L.J.; Njoya, E.M.; Coopoosamy, R.M.; Van-Staden, J., 2019. Antioxidant, anti-inflammatory and wound healing properties of medicinal plant extracts used to treat wounds and dermatological disorders. *South African Journal of Botany*. 126, 232–240. doi.org/10.1016/j.sajb.2019.07.013.

Giovannini, D.; Gismondi, A.; Basso, A.; Canuti, L.; Braglia, R.; Canini, A.; Mariani, F.; Cappelli, G. (2016). *Lavandula angustifolia* mill. Essential oil exerts antibacterial and anti-inflammatory effects in macrophage mediated immune response to staphylococcus aureus. *Immunological Investigations*, *45*(1), 11–28. doi.org/10.3109/08820139.2015.1085392

González-Ramírez, A.; González-Trujano, M.E.; Pellicer, F.; López-Muñoz F. J. (2012). Anti-nociceptive and anti-inflammatory activities of the *Agastache mexicana* extracts by using several experimental models in rodents. *Journal of Ethnopharmacology*, 142(3), 700–705. doi.org/10.1016/j.jep.2012.05.044

Grzegorczyk-Karolak, I.; Staniewska, P.; Lebelt, L.; Piotrowska, D. (2022). Optimization of cultivation conditions of *Salvia viridis* L. shoots in the Plantform bioreactor to increase polyphenol production. *Plant Cell, Tissue and Organ Culture* (PCTOC), 149:269–280. https://doi.org/10.1007/s11240-021-02168-2

Guginski, G.; Luiz, A.P.; Silva, M.D.; Massaro, M.; Martins, D.F.; Chaves, J.; Mattos, R.W.; Silveira, D.; Ferreira, V.M.M.; Calixto, J. B.; Santos, A.R.S. (2009). Mechanisms involved in the antinociception caused by ethanolic extract obtained from the leaves of *Melissa officinalis* (lemon balm) in mice. *Pharmacology Biochemistry and Behavior*, *93*(1), 10–16. doi.org/10.1016/j.pbb.2009.03.014

Güneş, S.; Savran, A.; Paksoy, M.Y.; Koşar, M.; Çakılcıoğlu, U. (2017). Ethnopharmacological survey of medicinal plants in Karaisalı and its surrounding (Adana-turkey). *Journal of Herbal Medicine*, 8, 68-75. doi:10.1016/j.hermed.2017.04.002

Hajhashemi, V.; Ghannadi, A.; Karim Pezeshkian, S. (2002). Antinociceptive and anti-inflammatory effects of *Satureja hortensis* L. extracts and essential oil. *Journal of Ethnopharmacology*, *82*, 83–87. www.elsevier.com/locate/jethpharm

Hajhashemi, V.; Ghannadi, A.; Sharif, B. (2003). Anti-inflammatory and analgesic properties of the leaf extracts and essential oil of *Lavandula angustifolia* Mill. *Journal of Ethnopharmacology*, *89*(1), 67–71. doi.org/10.1016/S0378-8741(03)00234-4

Hajhashemi, V.; Zolfaghari, B.; Yousefi, A. (2012). Antinociceptive and anti-inflammatory activities of *Satureja hortensis* seed essential oil, hydroalcoholic and polyphenolic extracts in animal models. *Medical Principles and Practice*, *21*(2), 178–182. doi.org/10.1159/000333555

Hamamouchi, J.; El Mahi, M.; El Abbes Faouzi, M. (2021). Investigation of *Origanum compactum* essential oil for analgesic and anti-inflammatory activities. E3S Web of Conferences, 319. https://doi.org/10.1051/e3sconf/202131901104

Harput, U.S.; Saracoglu, .I; Ogihara, Y. (2006). Effects of two *Prunella* species on lymphocyte proliferation and nitric oxide production. *Phytotherapy Research*, 20(2):157-159. https://doi: 10.1002/ptr.1805. PMID: 16444671.

Hassan, Z.Y.; Hassan, T.Y.; Abu-Raghif, A.R. (2022). Evaluation the Effectiveness of Phenolic Compound of *Salvia frigida* on Induced Atopic Dermatitis in Experimental Mice. *Iraqi Journal of Pharmaceutical Sciences*, *31*(1), 154–166. doi.org/10.31351/vol31iss1pp154-166

He, H.; Jiang, H.; Chen, Y.; Ye, J.; Wang, A.; Wang, C.; Liu, Q.; Liang, G.; Deng, X.; Jiang, W.; Zhou, R. (2018). Oridonin is a covalent NLRP3 inhibitor with strong anti-inflammasome activity. *Nature Communications*, *9*(1). doi.org/10.1038/s41467-018-04947-6

Hernández-Herrera, A.D.; Luna-Herrera, J.; Del Rocío González-Martínez, M.; Prieto-Hinojosa, A.I.; Turcios-Esquivel, A.M.; Castillo-Maldonado, I.; Delgadillo-Guzmán, D.; Ramírez-Moreno, A.; Bustos-Brito, C.; Esquivel, B.; Vega-Menchaca, M.D.; Pedroza-Escobar, D. (2023). Immunomodulatory activity of diterpenes over innate immunity and cytokine production in a human alveolar epithelial cell line infected with *Mycobacterium tuberculosis*. *Current Molecular Pharmacology*, 16(6), 682-689. doi: 10.2174/1874467215666221005115007.

Hernandez-Perez, M.; Rabanal, R.M.; De La Torre, M.C.; Rodriguez, B. (1995). Analgesic, anti-inflammatory, antipyretic and haematological effects of aethiopinone, an o-naphthoquinone diterpenoid from *Salvia aethiopis* roots and two hemisynthetic derivatives. *Planta Medica*, 61(6), 505-509. doi:10.1055/s-2006-959358

Hong, S.S.; Lee, C.; Lee, C.H.; Park, M.; Lee, M.S.; Hong, J.T.; Lee, H.; Lee, M.K.; Hwang, B.Y. (2009). A new furofuran lignan from *Isodon japonicus*. *Archives of Pharmacal Research*, 32(4), 501–504. https://doi.org/10.1007/s12272-009-1404-x

Hosseinzadeh, H.; Yavary, M. (1999). Anti-inflammatory effect of *Salvia leriifolia* Benth. leaf extract in mice and rats. *Pharmaceutical and Pharmacological Letters*, *9*(2), 60–61.

Hosseinzadeh, H.; Haddadkhodaparast, M.H.; Arash, A.R. (2003). Antinociceptive, antiinflammatory and acute toxicity effects of *Salvia leriifolia* benth. Seed extract in mice and rats. *Phytotherapy Research*, *17*(4), 422–425. doi.org/10.1002/ptr.1154

Hsu, C.L.; Hong, B.O.H.; Shan, Y.U.; Yen, G.C. (2010). Antioxidant and Anti-Inflammatory effects of *Orthosiphon aristatus* and its bioactive compounds. *Journal of Agricultural and Food Chemistry*, *58*(4), 2150–2156. doi.org/10.1021/jf903557c

Huang, B.P.; Lin, C.H.; Chen, Y.C.; Kao, S.H. (2014). Anti-inflammatory effects of *Perilla frutescens* leaf extract on lipopolysaccharide stimulated RAW264.7 cells. *Molecular Medicine Reports*, *10*(2), 1077–1083. doi.org/10.3892/mmr.2014.2298

Hussain, L.; Sajid Hamid Akash, M.; Imran Qadir, M.; Iqbal, R.; Irfan, M.; Imran Qadir, M. (2017). Analgesic, anti-inflammatory, and antipyretic activity of *Salvia moorcroftiana*. *Article in Pakistan Journal of Pharmaceutical Sciences,* 30(2). www.researchgate.net/publication/316007134.

Hwang, B.; Lee, J.; Koo, T.; Kim, H.; Hong, Y.; Ro, J.; Lee, K.; Lee, J. (2001). Kaurane diterpenes from *Isodon japonicus* inhibit nitric oxide and prostaglandin E2 production and NF-KB activation in LPS-stimulated macrophage RAW264.7 cells. *Planta Medica,* 67, 406–410.

Hwang, Y J.; Lee, E.J.; Kim, H.R.; Hwang, K.A. (2013). NF-κB-targeted anti-inflammatory activity of *Prunella vulgaris* var. *lilacina* in macrophages RAW 264.7. *International Journal of Molecular Sciences*, *14*(11), 21489–21503. doi.org/10.3390/ijms141121489.

Ibrahim, A.Y.; Shahat, A.; Mohamed, T.; Elshamy, A.; Alqahtani, A.; Saleh, I.; Hassan, E.; Hegazy, M. 2020. *In vitro* anti-inflammatory and acetylcholinesterase inhibition efficiency of plant extracts from Sinai-Egypt. I*ndian Journal of Traditional Knowledge*, 19 (3): 635-641.

Ibrahim, A.Y.; Youness, E.R.; Shalaby, A.S.; Magd El-Din, M. (2016). Anti-acetylcholinesterase, Anti-inflammatory and Anti-oxidant Activities of *Mentha longifolia* for Treating Alzheimer Disease. *Der Pharmacia Lettre*, *8*(7), 34–39.

Ikoma, K.; Takahama, M.; Kimishima, A.; Pan, Y.; Taura, M.; Nakayama, A.; Arai, M.; Takemura, N.; Saitoh, T. (2022). Oridonin suppresses particulate-induced NLRP3-independent IL-1α release to prevent crystallopathy in the lung. *International Immunology*, 34(10), 493–504. https://doi.org/10.1093/intimm/dxac018

Ismaili, H.; Sosa, S.; Brkic, D.; Aquino, R.P.; Tubaro, A. (2002). Topical anti-inflammatory activity of extracts and compounds from *Thymus broussonettii*. *Journal of Pharmacy and Pharmacology*, 54(8), pp. 1137–1140.

Jang, H.H.; Cho, S.Y.; Kim, M.J.; Kim, J.B.; Lee, S.H.; Lee, M.Y.; Lee, Y.M. (2016). Anti-inflammatory effects of *Salvia plebeia* R. Br extract in vitro and in ovalbumin-induced mouse model. *Biological Research*, *49*(1). doi.org/10.1186/s40659-016-0102-7

Jang, H.H.; Lee, S.; Lee, S.J.; Lim, H.J.; Jung, K.; Kim, Y.H.; Lee, S.W.; Rho, M.C. (2017). Anti-inflammatory Activity of Eudesmane-Type Sesquiterpenoids from *Salvia plebeia*. *Journal of Natural Products*, *80*(10), 2666–2676. doi.org/10.1021/acs.jnatprod.7b00326

Javdan, N.; Estakhr, J. (2011b). Evaluation of the effects of *Salvia hypoleuca* on the expression of cytokines: IL-6, IL-10 and TNF-α in high fat diet-fed mice towards a cure for *Diabetes mellitus*. *Pharmacologyonline*, 2, 842-852. Retrieved from www.scopus.com

Jeong, H.; Sung, M.; Kim, Y.; Ham, H.; Choi, Y.; Lee, J. (2012). Anti-inflammatory activity of *Salvia plebeia* R. Br. leaf through heme oxygenase-1 induction in LPS-stimulated RAW264.7 macrophages. *Journal of the Korean Society of Food Science and Nutrition*, *41*(7), 888–894. doi.org/10.3746/jkfn.2012.41.7.888

Jia, T.; Cai, M.; Ma, X., Li, M.; Qiao, J.; Chen, T. (2019). Oridonin inhibits IL-1β-induced inflammation in human osteoarthritis chondrocytes by activating PPAR-γ. *International Immunopharmacology*, 69, 382–388. doi.org/10.1016/j.intimp.2019.01.049

Jiang, H.; Li, X.; Sun, H.; Zhang, H.; Puno, P. (2018). Scopariusols L-T, nine new ent-kaurane diterpenoids isolated from *Isodon scoparius*. *Chinese Journal of Natural Medicines*, 16(6), 456–464.

Jiang, H.-Y.; Wang, W.-G.; Zhou, M.; Wu, H.-Y.; Zhan, R.; Du, X.; Pu, J.-X.; Sun, H.-D. (2014a). 6,7-seco-ent-Kaurane diterpenoids from *Isodon sculponeatus* and their bioactivity. *Chinese Chemical Letters*, 25, 541–544. http://dx.doi.org/10.1016/j.cclet.2014.01.041

Jiang, H.-Y.; Wang, W.-G.; Zhou, M.; Wu, H.-Y.; Zhan, R.; Li, X.-N.; Du, X.; Li, Y.; Pu, J.-X.; Sun, H.-D. (2014b). Diterpenoids from *Isodon sculponeatus*, *Fitoterapia*, 93, 142–149. http://dx.doi.org/10.1016/j.fitote.2013.12.025.

Kamatou, G.P.P.; Viljoen, A.M. (2010). A review of the application and pharmacological properties of α-bisabolol and α-bisabolol-rich oils. *JAOCS, Journal of the American Oil Chemists' Society*, 87(1), 1-7. doi:10.1007/s11746-009-1483-3

Kamatou, G.P.P.; Makunga, N.P.; Ramogola, W.P.N.; Viljoen, A.M. (2008). South african *Salvia* species: A review of biological activities and phytochemistry. *Journal of Ethnopharmacology*, 119(3), 664-672. doi:10.1016/j.jep.2008.06.030

Kamatou, G.P.P.; Van Zyl, R.L.; Van Vuuren, S.F.; Viljoen, A.M.; Figueiredo, A.C.; Barroso, J.G.; Tilney, P.M. (2006). Chemical composition, leaf trichome types and biological activities of the essential oils of four related *Salvia* species indigenous to southern Africa. *Journal of Essential Oil Research*, 18, 72-79. doi:10.1080/10412905.2006.12067125

Kamatou, G.P.P.; Viljoen, A.M.; Steenkamp, P. (2010). Antioxidant, antiinflammatory activities and HPLC analysis of south african *Salvia* species. *Food Chemistry*, 119(2), 684-688. doi:10.1016/j.foodchem.2009.07.010.

Kamatou, G.P.P.; Viljoen, A.M.; Gono-Bwalya, A. B.; Van Zyl, R.L.; Van Vuuren, S.F.; Lourens, A.C.U.; Steenkamp, P. (2005). The *in vitro* pharmacological activities and a chemical investigation of three south african *Salvia* species. *Journal of Ethnopharmacology*, 102(3), 382-390. doi:10.1016/j.jep.2005.06.034

Kamatou, G.P.P.; Van Zyl, R.L.; Van Vuuren, S.F.; Viljoen, A.M.; Figueiredo, A.C.; Barroso, J.G.; Pedro, L.G. (2006). Biological activities and composition of *Salvia muirii* L. essential oil. *Journal of Essential Oil Research*, 18, 48-51. https://doi.org/10.1080/10412905.2006.12067119.

Kamatou, G.P.P.; Viljoen, A.M.; Figueiredo, A.C.; Tilney, P.M.; Van Zyl, R.L.; Barroso, J.G.; Pedro, L.G.; Van Vuuren, S.F. (2007). Trichomes, essential oil composition and biological activities of *Salvia albicaulis* Benth. and *S. dolomitica* Codd., two species from the Cape region of South Africa. *South African Journal of Botany*, 73: 102-108. https://doi.org/10.1016/j.sajb.2006.08.001

Kang, H. (2018). Anti-inflammatory mechanism of I*sodon Japonicas* (Burm.) Hara on lipopolysaccharide-induced neuroinflammation. *Tropical Journal of Pharmaceutical Research*, 17(1), 85–89. https://doi.org/10.4314/tjpr.v17i1.13

Suttajit, M. (2021). Anti-inflammatory effect of *Perilla frutescens* seed oil rich in omega-3 fatty acid on dextran sodium sulfate-induced colitis in mice. *Research in Pharmaceutical Sciences*, *16*(5), 464–473. doi.org/10.4103/1735-5362.323913

Kapewangolo, P.; Hussein, A.A.; Meyer, D. (2013). Inhibition of HIV-1 enzymes, antioxidant and anti-inflammatory activities of *Plectranthus barbatus*. *Journal of Ethnopharmacology*, 149(1), 184–190. doi.org/10.1016/j.jep.2013.06.019

Kapewangolo, P.; Omolo, J.J.; Bruwer, R.; Fonteh, P.; Meyer, D. (2015). Antioxidant and anti-inflammatory activity of *Ocimum labiatum* extract and isolated labdane diterpenoid. *Journal of Inflammation,* 12, 4.doi: 10.1186/s12950-015-0049-4.

Karabay-Yavasoglu, N.U.; Baykan, S.; Ozturk, B.; Apaydin, S.; Tuglular, I. (2006). Evaluation of the antinociceptive and anti-inflammatory activities of *Satureja thymbra* L. essential oil. *Pharmaceutical Biology*, 44(8), pp. 585–591.

Karaca, N.; Demirci, B.; Gavahian, M.; Demirci, F. (2023). Enhanced Bioactivity of Rosemary, Sage, Lavender, and Chamomile Essential Oils by Fractionation, Combination, and Emulsification. *ACS Omega,* 8(12), pp. 10941–10953.

Karakaş, Ö.; Bekler, F.M. (2022). Essential oil compositions and antimicrobial activities of *Thymbra spicata* L. var. *spicata* L., *Lavandula* X *intermedia* Emeric ex Loisel., *Satureja macrantha* C. A. MEYER and *Rosmarinus officinalis* L. *Brazilian Archives of Biology and Technology*, 65, 1-11. doi:10.1590/1678-4324-2022210297.

Karimian, P.; Kavoosi, G.; Amirghofran, Z. (2013). Anti-inflammatory effect of *Mentha longifolia* in lipopolysaccharide- stimulated macrophages: Reduction of nitric oxide production through inhibition of inducible nitric oxide synthase. *Journal of Immunotoxicology*, *10*(4), 393–400. doi.org/10.3109/1547691X.2012.758679

Kenari, H.M.; Yousefsani, B.S.; Eghbalian, F.; ...Jamshidi, A.H.; Mahroozade, S. (2021). Herbal recommendations for treatment of COVID-19 symptoms according to Persian medicine. *Journal of Medicinal Plants*, 20(77), pp. 1–14

Khalighi-Sigaroodi, F.; Ahvazi, M.; Ebrahimzadeh, H.; Rahimifard, N. (2013). Chemical composition of the essential oil and antioxidant activities, total phenol and flavonoid content of the extract of *Nepeta pogonosperma*. *Journal of Medicinal Plants*, 12(48), 185–199.

Khan, S.; Iqbal Yatoo, M. (n.d.). Potential herbs for bovine mastitis research-a mini review Anti-Neoplastic effects of Garcinol View project NFBSFARA project in 2010-11 session View project. https://www.researchgate.net/publication/358925222

Khankandi, H.P.; Behzad, S.; Mojab, F.; Ahmadian-Attari, M.M.; Sahranavard, S. (2019). Effects of some Lamiaceae species on NO production and cell injury in hydrogen peroxide-induced stress. *Iranian Journal of Pharmaceutical Research*, *18*(2), 826–835. doi.org/10.22037/ijpr.2019.1100685

Khazdair, M.R.; Ghorani, V.; Alavinezhad, A.; Boskabady, M.H. (2018). Pharmacological effects of *Zataria multiflora* Boiss L. and its constituents focus on their anti-inflammatory, antioxidant, and immunomodulatory effects. *Fundamental and Clinical Pharmacology* (Vol. 32, Issue 1, pp. 26–50). Blackwell Publishing Ltd. doi.org/10.1111/fcp.12331

Khouchlaa, A.; Et-Touys, A.; Lakhdar, F.; ...El Idrissi, A.E.Y.; Zaakour, F. (2022). Ethnomedicinal use, phytochemistry, pharmacology, and toxicology of *Salvia verbenaca* L.: A review. *Biointerface Research in Applied Chemistry*, 12(2), pp. 1437–1469.

Kim, B.W.; Koppula, S.; Kim, I.S.; Lim, H.W.; Hong, S.M.; Han, S.D.; Hwang, B.Y. (2011). Anti-neuroinflammatory activity of kamebakaurin from *Isodon japonicus* via inhibition of c-Jun NH2-terminal kinase and p38 mitogen-activated protein kinase p Choathway in activated microglial cells. *Journal of Pharmacological Sciences*, 116(3), 296–308. https://doi.org/10.1254/jphs.10324FP

Kim, B.; Han, Y.E.; Lee, H.J. (2017). Antioxidant and anti-inflammatory effects of the ethyl acetate fraction of the *Agastache rugosa* extract. *Korean Journal of Food Science and Technology*, *49*(3), 331–337. doi.org/10.9721/KJFST.2017.49.3.331

Kim, D.; Lee, S.K.; Park, K.-S.; Park, H.-J. 2020. Isolation of the constituents from *Clinopodium chinense* var. *shibetchense* and inhibition activity on cancer cell growth and nitric oxide production. *Korean Journal of Pharmacognosy*, 51(2), pp. 93–99.

Kim, H.; Hong, J.Y.; Jeon, W.J.; Lee, J.; Baek, S.H.; Ha, I.H. (2021). *Lycopus lucidus* Turcz exerts neuroprotective effects against H2O2-induced neuroinflammation by inhibiting NLRP3 inflammasome activation in cortical neurons. *Journal of Inflammation Research*, 14, 1759–1773. https://doi.org/10.2147/JIR.S305031

Kim, H.-H.; Yoo, J.-S.; Lee, H.-S.; ...Shin, T.-Y.; Kim, S.-H. (2011). *Elsholtzia ciliata* inhibits mast cell-mediated allergic inflammation: Role of calcium, p38 mitogen-activated protein kinase and nuclear factor-kB. *Experimental Biology and Medicine*, 236(9), pp. 1070–1077.

Kim, J.Y.; Kim, H.S.; Kim, Y.J.; Lee, H.K.; Kim, J.S.; Kang, J.S.; Hong, J.T.; Kim, Y.; Hwang, B.Y.; Han, S.B. (2013). Effusanin C inhibits inflammatory responses via blocking NF-κB and MAPK signaling in monocytes. *International Immunopharmacology*, 15(1), 84–88. https://doi.org/10.1016/j.intimp.2012.11.002

Kim, J.; Song, S.; Lee, I.; Kim, Y.; Yoo, I.; Ryoo, I.; Bae, K. (2011). Anti-inflammatory activity of constituents from *Glechoma hederacea* var. l*ongituba*. *Bioorganic and Medicinal Chemistry Letters*, *21*(11), 3483–3487. doi.org/10.1016/j.bmcl.2011.02.002

Kim, K.M.; Kim, S.Y.; Mony, T.J.; Bae, H.J.; Han, S.D.; Lee, E.S.; Choi, S.H.; Hong, S.H.; Lee, S.D.; Park, S.J. (2021). *Dracocephalum moldavica* ethanol extract suppresses LPS-induced inflammatory responses through inhibition of the jnk/erk/nf-κb signaling pathway and il-6 production in raw 264.7 macrophages and in endotoxic-treated mice. *Nutrients*, *13*(12). doi.org/10.3390/nu13124501

Kim, M.; Sowndhararajan, K.; Kim, S. 2022. The Chemical Composition and Biological Activities of Essential Oil from Korean Native Thyme Bak-Ri-Hyang (*Thymus quinquecostatus* Celak.). Molecules 2022,27,4251. doi.org/10.3390/molecules27134251.

Kim, S.Y.; Han, S.D.; Kim, M.; Mony, T.J.; Lee, E.S.; Kim, K.M.; Choi, S.H.; Hong, S.H.; Choi, J.W.; Park, S.J. (2021b). *Mentha Arvensis* essential oil exerts anti-inflammatory in LPS-stimulated inflammatory responses via inhibition of ERK/nf-κb signaling pathway and anti-atopic dermatitis-like effects in 2,4-dinitrochlorobezene-induced BALB/C mice. *Antioxidants*, *10*(12). doi.org/10.3390/antiox10121941

Kim, T.-W.; Kim, Y.-J.; Seo, C.-S.; ...Lee, M.-Y.; Jung, J.-Y. (2016). *Elsholtzia ciliata* (Thunb.) Hylander attenuates renal inflammation and interstitial fibrosis via regulation of TGF-ß and Smad3 expression on unilateral ureteral obstruction rat model. *Phytomedicine*, 23(4), pp. 331–339.

Kim, W.; Son, K.; Chang, H.; Bae, K.; Kang, S.; Kim, H. (2003). Anti-Inflammatory activity of *Elsholtzia splendens*. *Archives of Pharmacal Researchs,* 26(3), 232–236.

Ko, H.M.; Koppula, S.; Kim, B.-W.; Kim, I.S.; Hwang, B.Y.; Suk, K.; Park, E.J.; Choi, D.-K. (2010). *European Journal of Pharmacology*, 633, 98–106. doi:10.1016/j.ejphar.2010.02.011.

Kogiannou, D.A.A.; Kalogeropoulos, N.; Kefalas, P.; Polissiou, M.G.; Kaliora, A.C. (2013). Herbal infusions; their phenolic profile, antioxidant and anti-inflammatory effects in HT29 and PC3 cells. *Food and Chemical Toxicology*, 61, 152–159. http://dx.doi.org/10.1016/j.fct.2013.05.027.

Kosar, M.; Goger, F.; Can Baser, K.H. *In vitro* antioxidant properties and phenolic composition of *Salvia virgata* Jacq. from Turkey. *J. Agric. Food Chem*., 2008, 6, 2369-2374.

Kostić, M.; Kitić, D.; Petrović, M.B.; Jevtović-Stoimenov, T.; Jović, M.; Petrović, A.; iŽvanović, S. (2017). Anti-inflammatory effect of the *Salvia sclarea* L. ethanolic extract on lipopolysaccharide-induced periodontitis in rats. *Journal of Ethnopharmacology*, 199, 52–59. doi.org/10.1016/j.jep.2017.01.020

Kulabas, S.S.; Ipek, H.; Tufekci, A.R.; Arslan, S.; Demirtas, I.; Ekren, R.; Sezerman, U.; T. B. Tumer. (2018). Ameliorative potential of *Lavandula stoechas* in metabolic syndrome via multitarget interactions. *Journal of Ethnopharmacology*, *223*, 88–98. doi.org/10.1016/j.jep.2018.04.043

Kumar, A.; Singh, S.; Kumar, A.; Umrao Bawankule, D.; Tandon, S.; Kumar, A.; Swaroop Verma, R.; Saikia, D. (2021). Chemical composition, bactericidal kinetics, mechanism of action, and anti-inflammatory activity of *Isodon melissoides* (Benth.) H. Hara essential oil, *Natural Product Research*, 35:4, 690-695. https://doi.org/10.1080/14786419.2019.1591399.

González Pereyra, M.; Cariddi, L. N.; Ybarra, F.; Isola, M. C.; Demo, M. S.; Sabini, L.; Maldonado, A. M. (n.d.). Immunomodulating properties of *Minthostachys verticillata* on human lymphocytes and basophils. www.revistasmedicasmexicanas.com.mx

Lee, J.W.; Lee, M.S.; Kim, T.H.; Lee, H.J.; Hong, S.S.; Noh, Y.H.; Hwang, B.Y.; Ro, J.S.; Hong, J.T. (2007). Inhibitory effect of Inflexinol on Nitric Oxide Generation and iNOS expression via inhibition of NF-κB activation. *Mediators of Inflammation*, 2007, 93148. doi: 10.1155/2007/93148.

Lee, Y.J.; Kang, D.G.; Kim, J.S.; Lee, H.S. (2008). *Lycopus lucidus* inhibits high glucose-induced vascular inflammation in human umbilical vein endothelial cells. *Vascular Pharmacology*, 48(1), 38–46. https://doi.org/10.1016/j.vph.2007.11.004

Lee, Y.; Lim, H.W.; Ryu, I.W.; Huang, Y.H.; Park, M.; Chi, Y.M.; Lim, C.J. (2020). Anti-Inflammatory, Barrier-Protective, and Antiwrinkle Properties of *Agastache rugosa* Kuntze in Human Epidermal Keratinocytes. *BioMed Research International*, *2020*. doi.org/10.1155/2020/1759067

Lehbili, M.; Alabdul Magid, A.; Kabouche, A.; Voutquenne-Nazabadioko, L.; Abedini, A.; Morjani, H.; Gangloff, S.C.; Kabouche, Z. (2018). Antibacterial, antioxidant and cytotoxic activities of triterpenes and flavonoids from the aerial parts of *Salvia barrelieri* Etl. *Natural Product Research*, *32*(22), 2683–2691. doi.org/10.1080/14786419.2017.1378207

Leung, C.H.; Grill, S.P.; Lam, W.; Gao, W.; Sun, H.D.; Cheng, Y.C. (2006). Eriocalyxin B inhibits nuclear factor-κB activation by interfering with the binding of both p65 and p50 to the response element in a noncompetitive manner. *Molecular Pharmacology*, *70*(6), 1946–1955. doi.org/10.1124/mol.106.028480

Li C.; Luo Y.; Zhang W.; Cai Q.; Wu X.; Tan Z.; Chen R.; Chen Z.; Wang S.; Zhang L. 2021. A comparative study on chemical compositions and biological activities of four essential oils: *Cymbopogon citratus* (DC.) Stapf, *Cinnamomum cassia* (L.) Presl, *Salvia japonica* Thunb. and *Rosa rugosa* Thunb. *Journal of Ethnopharmacology,* 280:114472. https://doi: 10.1016/j.jep.2021.114472

Li L.; Zhou M.; Xue G.; Wang W.; Zhou X.; Wang X.; Kong L.; Luo J. 2018. Bioactive seco-abietane rearranged diterpenoids from the aerial parts of *Salvia prionitis*. *Bioorganic Chemistry*, 81:454-460. https://doi: 10.1016/j.bioorg.2018.08.040

Li, J.; Du, J., Sun, L.; Liu, J.; Quan, Z. (2010). Anti-Inflammatory Function of Nodosin via Inhibition of IL-2. *The American Journal of Chinese Medicine* (Vol. 38, Issue 1). www.worldscientific.com

Li, L.; Huang, Q.; Duan, X.; Han, L.; Peng, D. (2020). Protective effect of *Clinopodium chinense* (Benth.) O. Kuntze against abnormal uterine bleeding in female rats. *Journal of Pharmacological Sciences*, 143(1), pp. 1–8

Li, L.-L.; Huang, Q.; Peng, D.-Y.; Qi, J.-J.; Yao, M. (2022). Anti-inflammatory and hemostatic effects of total extract, saponins, and flavonoids of *Clinopodium chinense* in female rats with abnormal uterine bleeding and mechanism. *Zhongguo Zhongyao Zazhi*, 47(12), pp. 3372–337

Li, S.M.; Yang, X.W.; Li, Y.L.; Shen, Y.H.; Feng, L.; Wang, Y.H.; Zeng, H.W.; Liu, X.H.; Zhang, C.S.; Long, C.L.; Zhang, W.D. (2009). Chemical constituents of *Dracocephalum forrestii*. *Planta Medica*, *75*(15), 1591–1596. doi.org/10.1055/s-0029-1185868

Li, S. Y.; Zhu, L.H.; Rao, Z.Y.; Wang, C.J.; Jiang, M.; Zhang, J.; Yin, Z.Q. (2021). Two new 7, 20-epoxy-ent-kauranes from the aerial parts of *Isodon eriocalyx*. *Natural Product Research*, *35*(21), 3859–3864. doi.org/10.1080/14786419.2020.1744141

Li, Y.X.; Liu, Y.B.; Ma, A.Q.; Bao, Y.; Wang, M.; Sun, Z.L. (2017). In vitro antiviral, anti-inflammatory, and antioxidant activities of the ethanol extract of *Mentha piperita* L. *Food Science and Biotechnology*, 26(6), 1675–1683. doi.org/10.1007/s10068-017-0217-9

Li, Y.-M.; Zou, H.-Y.; Hu, H.-Y.; Di, L.; Li, H.-L.; Hu, Z.; Lin, L.; Liao, D.; Xia, B.; Lin, Y. (2020). Effect of water extract of *Prunella vulgaris* on NLRP3/Caspase-1/IL-1B pathway in rats with allergic conjunctivitis. *Chinese Journal of New Drugs*, 29(8), 929–933. doi.org/10.1097/ACI.0000000000000100

Lim, H.W.; Lee, Y.; Huang, Y.H.; Yoon, J.Y.; Lee, S.H.; Kim, K.; Lim, C.J. (2017). Enhancement of skin antioxidant and anti-inflammatory potentials of *Agastache rugosa* leaf extract by probiotic bacterial fermentation in human epidermal keratinocytes. *Microbiology and Biotechnology Letters*, *45*(1), 35–42. doi.org/10.4014/mbl.1701.01002.

Lim, J.Y.; Won, T.J.; Hwang, B.Y.; Kim, H.R.; Hwang, K.W.; Sul, D.; Park, S.Y. (2010). The new diterpene isodojaponin D inhibited LPS-induced microglial activation through NF-kappaB and MAPK signaling pathways. *European Journal of Pharmacology*, 642(1–3), 10–18. https://doi.org/10.1016/j.ejphar.2010.05.047

Ling-Fei, K.; Xiao-Juan, R.; Pan, Y.; ...Tian-Le, G.; Cai, T. (2023). The influence of *Hyssopus cuspidatus* Boriss extract on lipid mediators metabolism network in asthmatic mice. *Frontiers in Pharmacology*, 14, 1066643.

Lino, C.S.; Gomes, P.B.; Lucetti, D.L.; ...Silva, M.G.V.; Viana, G.S.B. (2005). Evaluation of antinociceptive and antiinflammatory activities of the essential oil (EO) of *Ocimum micranthum* Willd. from northeastern Brazil. *Phytotherapy Research*, 19(8), pp. 708–712.

Liu, D.; Wang, Y.; Lu, Z.; Lv, F.; Bie, X.; Zhao, H. (2022b). Separation, characterization, and anti-inflammatory activities of galactoglycerolipids from *Perilla frutescens* (L.) Britton. *Natural Product Research*. doi.org/10.1080/14786419.2022.2095637.

Liu, X.; Su, J.; Wang, G.; ...Wang, S.; Huang, Y. (2021). Discovery of phenolic glycoside from *Hyssopus cuspidatus* attenuates LPS-induced inflammatory responses by inhibition of iNOS and COX-2 expression through suppression of NF-κB activation. *International Journal of Molecular Sciences*, 22(22), 12128.

Liu, X.; Wang, G.; Lv, C.; ...Wang, S.; Huang, Y. (202). 1Two new phenolic acids and one new phenolic glycoside from *Hyssopus cuspidatus* Boriss and their anti-inflammatory activities. *Phytochemistry Letters*, 46, pp. 15–20.

Liu, Y.; Song, H.; Xu, J.; Bi, G.; Meng, D. (2022). Anti-inflammatory abietanes diterpenes and triterpenoids isolated from *Clinopodium polycephalum*. *Fitoterapia, 161*. doi.org/10.1016/j.fitote.2022.105244

Llorent-Martínez, E.J.; Ruiz-Medina, A.; Zengin, G.; Ak, G.; Jugreet, S.; Mahomoodally, M.F.; Chiavaroli, A. (2022). New biological and chemical evidences of two Lamiaceae species (*Thymbra capitata* and *Thymus sipyleus* subsp. *rosulans*): In vitro, in silico and ex vivo approaches. *Molecules*, 27(24) doi:10.3390/molecules27249029

Loizzo, M.; Menichini, F.; Tundis, R.; Bonesi, M.; Conforti, F.; Nadjafi, F.; Statti, G.; Frega, N.; Menichini, F. (2009). *In vitro* biological activity of *Salvia lerrifolia* Benth. essential oil relevant to the treatment of Alzheimer´s disease. *Journal of Oleo Science, 58*(8), 443–446.

Lopes, C.L.; Pereira, E.; Soković, M.; ...Barros, L.; Ferreira, I.C.F.R. (2018). Phenolic Composition and Bioactivity of *Lavandula pedunculata* (Mill.) Cav. Samples from Different Geographical Origin. *Molecules*, 23(5), 1037.

Ĺopez-Garcia, R.E.; Rabanal, R.M.; Darias, V.; Martín-Herrera, D.; Carreiras, M.C.; Rodríguez, B. (1991), A preliminary study of *Cedronella canariensis* (L.) var. *canariensis* extracts for antiinflammatory and analgesic activity in rats and mice. *Phytotherapy Research*, 5: 273-275. doi.org/10.1002/ptr.2650050610,

Lou, D.; Yan, D.; Guo, M.; Wang, Q.; Jiang, C.; Yin, Z. (2019). Antipyretic effect of *Isodon eriocalyx* extract on yeast-induced fever in rats. *Journal of China Pharmaceutical University* 6, 87-92.

Lu, Y.; Chen, B.; Song, J.H.; Zhen, T.; Wang, B.Y.; Li, X.; Liu, P.; Yang, X.; Zhang, Q.L.; Xi, X.D.; Chen, S.; di, Zuo, J.P.; Chen, Z.; Chen, S.J. (2013). Eriocalyxin B ameliorates experimental autoimmune encephalomyelitis by suppressing Th1 and Th17 cells. *Proceedings of the National Academy of Sciences of the United States of America*, *110*(6), 2258–2263. doi.org/10.1073/pnas.1222426110

Lucca, L.G.; Romão, P.R.T.; Vignoli-Silva, M.; da VeigaJunior, V.F.; Koester, L.S. (2022). *In vivo* Acute Anti-Inflammatory Activity of Essential Oils: A Review. *Mini-Reviews in Medicinal Chemistry*, 22(11), pp. 1495–1515.

Luís, Â.; Domingues, F. (2021). Screening of the potential bioactivities of pennyroyal (*Mentha pulegium* L.) essential oil. *Antibiotics*, *10*(10). doi.org/10.3390/antibiotics10101266

Ma, X.; Ma, X.; Ma, Z.; Wang, J.; Sun, Z.; Yu, W.; Li, F.; Ding, J. (2014). Effect of *Hyssopus officinalis* L. on inhibiting airway inflammation and immune regulation in a chronic asthmatic mouse model. *Experimental and Therapeutic Medicine*, *8*(5), 1371–1374. doi.org/10.3892/etm.2014.1978

Magalhães, A.; Santos, G.B.; Verdam, M.C.; Fraporti, L.; Malheiro, A.; Lima, E.S.; Dos-Santos, M.C. (2011). Inhibition of the inflammatory and coagulant action of *Bothrops atrox* venom by the plant species *Marsypianthes chamaedrys*. *Journal of Ethnopharmacology*, 134(1), 82–88. doi.org/10.1016/j.jep.2010.11.062.

Mahalakshmi, B.; Huang, C.Y.; Lee, S.; da Maurya, N.; Kiefer, R.; Bharath Kumar, V. (2021). Review of Danshen: From its metabolism to possible mechanisms of its biological activities. In *Journal of Functional Foods*, 85. Elsevier Ltd. doi.org/10.1016/j.jff.2021.104613

Mahmoud, A.M.; Wilkinson, F.L.; Sandhu, M.A.; Dos Santos, J.M.; Alexander, M.Y. 2019. Modulating oxidative stress in drug-induced injury and metabolic disorders: the role of natural and synthetic antioxidants. *Oxidative Medicine and Cellular Longevity*, 2019:3206401. https://doi: 10.1155/2019/3206401.

Mahmoudi, M.; Morteza-Semnani, K.; Mojra, E. (2008). Anti-inflammatory and antinociceptive activity of *Thymus pubescens* extract. *Fitoterapia*, *79*(5), 361–365. doi.org/10.1016/j.fitote.2008.04.004

Mahomoodally, M.F.; Devi Dursun, P.; Venugopala, K.N. (2021). *Collinsonia canadensis* L. In *Naturally Occurring Chemicals against Alzheimer's Disease*, 373–377. Elsevier. doi.org/10.1016/B978-0-12-819212-2.00031-1

Maklad, Y.A.; Aboutabl, E.A.; El-Sherei, M.M.; Meselhy, K.M. (1999). Bioactivity studies of *Salvia transsylvanica* (Schur. ex Griseb.) grown in Egypt. *Phytotherapy Research*, *13*(2), 147–150. doi.org/10.1002/(SICI)1099-1573(199903)13:2<147:AID-PTR397>3.0.CO;2-E

Martins-Gomes, C.; Taghouti, M.; Schäfer, J.; Silva, A.M.; Nunes, F.M. 2018. Chemical characterization and bioactive properties of decoctions and hydroethanolic extracts of *Thymus carnosus* Boiss. *Journal of Functional Foods*, 43, pp. 154–164.

Mehmet, S.I.; Gürbüz, I.; Bedir, E.; Günbatan, T.; Demirci, F., 2021. Isolation of rosmarinic acid and methyl rosmarinate as lipoxygenase inhibitors from *Salvia palaestina* Benth. by activity-guided fractionation. *South African Journal of Botany*, 141: 177-182. https://doi.org/10.1016/j.sajb.2021.04.030

Memariani, Z.; Rahimi, A.; Farzaei, M.H.; Zakaria Nejad, N. (2019). *Nepeta menthoides* Boiss. Buhse, an endemic species in Iran: A review of traditional uses, phytochemistry and pharmacology. *Journal of Herbmed Pharmacology*, 8(3), 194-204. doi: 10.15171/jhp.2019.29.

Mendi, A.; Yagci, B. G.; Kiziloglu, M.; Saraç, N.; Ugur, A.; Yilmaz, D.; Uçkan, D. (2017). *Thymbra spicata* var. *intricata* induces mesenchymal stem cell proliferation and osteogenic differentiation. *Brazilian Archives of Biology and Technology*, 60 doi:10.1590/1678-4324-2017160391

Messaoudi, M.; Rebiai, A.; Sawicka, B.; Atanassova, M.; Ouakouak, H.; Larkem, I.; Egbuna, C.; Awuchi, C.G.; Boubekeur, S.; Ferhat, M.A.; Begaa, S.; Benchikha, N. (2022). Effect of extraction methods on polyphenols, flavonoids, mineral elements, and biological activities of essential oil and extracts of *Mentha pulegium* L. *Molecules*, *27*(1). doi.org/10.3390/molecules27010011

Mićović, T.; Katanić Stanković, J. S.; Bauer, R.; Nöst, X.; Marković, Z.; Milenković, D.; Jakovljević, V.; Tomović, M.; Bradić, J.; Stešević, D.; Stojanović, D.; Maksimović, Z. (2022). In vitro, in vivo and in silico evaluation of the anti-inflammatory potential of *Hyssopus officinalis* L. subsp. *aristatus* (Godr.) Nyman (Lamiaceae). *Journal of Ethnopharmacology*, 293. doi.org/10.1016/j.jep.2022.115201

Miguel, M.G.; da Silva, C.I.; Farah, L.; Braga, F.C.; Figueiredo, A.C. (2021). Effect of essential oils on the release of TNF-α and CCL2 by LPS-stimulated THP-1 cells. *Plants*, *10*(1), 1–14. doi.org/10.3390/plants10010050

Milijasevic, B.; Steinbach, M.; Mikov, M.; Raskovic, A.; Capo, I.; Zivkovic, J.; Borisev, I.; Canji, J.; Teofilovic, B.; Vujcic, M.; Popovic, M. (2022). Impact of winter savory extract (*Satureja montana* L.) on biochemical parameters in serum and oxidative status of liver with application of the principal component analysis in extraction solvent selection. *European Review for Medical and Pharmacological Sciences*, *26*, 4721–4734.

Min, G.Y.; Kim, E.Y.; Hong, S.; Kim, J.H.; Kim, M.; Kim, E.J.; Park, J.H.; Sohn, Y.; Jung, H.S. (2021). *Lycopus lucidus* Turcz. ameliorates DNCB-induced atopic dermatitis in BALB/c mice. *Molecular Medicine Reports*, 24(6). https://doi.org/10.3892/mmr.2021.12467.

Minaiyan, M.; Sadraei, H.; Asghari, G.; Khanabadi, M.; Minaiyan, M. (2017). Anti-inflammatory effect of apigenin and hydroalcoholic extract of *Dracocephalum kotschyi* on acetic acid-induced colitis in rats. *Research in Pharmaceutical Sciences* (Vol. 12, Issue 4).

Minaiyan, M.; Sadraei, H.; Yousefi, I.; Sajjadi, S.E.; Talebi, A. (2021). Evaluation of the effect of hydroalcoholic and flavonoid-enriched extracts of *Dracocephalum kotschyi* on indomethacin-induced gastric ulcer in rats. *Research in Pharmaceutical Sciences*, 16(2), 141–152. https://doi.org/10.4103/1735-5362.310521

Miraj, S.; Kiani, S. (2016). A review study of therapeutic effects of *Salvia officinalis* L. *Der Pharmacia Lettre*, *8*(6), 299–303. www.researchgate.net/publication/305147951

Miranda, R.S.; de Jesus, B.D.S.M.; da Silva Luiz, S.R.; Viana, C.B.; Adão Malafaia, C.R.; Figueiredo, F.S.; Carvalho, T.D.S.C.; Silva, M.L.; Londero, V.S.; da Costa-Silva, T.A.; Lago J.H.G., Martins, R.C.C. 2022. Antiinflammatory activity of natural triterpenes-An overview from 2006 to 2021. *Phytother Research*, 36(4):1459-1506. https://doi: 10.1002/ptr.7359.

Modarres, M.; Asili, J.; Lahouti, M.; Gangali, A.; Iranshahy, M.; Sahebkar, A. (2014). Simultaneous determination of rosmarinic acid, salvianolic acid B and caffeic acid in *Salvia leriifolia* Benth. root, leaf and callus extracts using a high-performance liquid chromatography with diode-array detection technique. *Journal of Liquid Chromatography and Related Technologies*, *37*(12), 1721–1730. doi.org/10.1080/10826076.2013.807466

Mogosan, C.; Vostinaru, O.; Oprean, R.; Heghes, C.; Filip, L.; Balica, G.; Moldovan, R.I.; Schmidt, T.J. (2017). A comparative analysis of the chemical composition, anti-inflammatory, and antinociceptive effects of the essential oils from three species of *Mentha* cultivated in Romania. *Molecules*, 22(2). https://doi.org/10.3390/molecules22020263

Mohanty, S.; Kamolvit, W.; Zambrana, S.; Sandström, C.; Gonzales, E.; Östenson, C. G.; Brauner, A. (2017). Extract of *Clinopodium bolivianum* protects against *E. coli* invasion of uroepithelial cells. *Journal of Ethnopharmacology*, *198*, 214–220. doi.org/10.1016/j.jep.2017.01.011

Mohsenzadeh, A.; Ahmadipour, S.; Ahmadipour, S.; Asadi-Samani, M. (2016). A review of the most important medicinal plants effective on cough in children and adults. *Der Pharmacia Lettre*, 8(1), 90-96.

Momtaz, S.; Abdollahi, M. 2010. An Update on Pharmacology of *Satureja* Species; From Antioxidant, Antimicrobial, Antidiabetes and Anti-hyperlipidemic to Reproductive Stimulation. *International Journ .,al of Pharmacology*, 6 (4): 346-353.

Montironi, I.D.; Reinoso, E. B.; Paullier, V.C.; Siri, M.I.; Pianzzola, M.J.; Moliva, M.; Campra, N.; Bagnis, G.; Ferreira LaRocque-de-Freitas, I.; Decote-Ricardo, D.; Freire-de-Lima, C. G.; Raviolo, J.M.; Cariddi, L.N. (2019). *Minthostachys verticillata* essential oil activates macrophage phagocytosis and modulates the innate immune response in a murine model of *Enterococcus faecium* mastitis. *Research in Veterinary Science*, 125, 333–344. https://doi.org/10.1016/j.rvsc.2019.07.015

Moraes, L.A.S.; Facanali, R.; Ortiz, M.; Marques, M.; Ming, L.C.; Angela, M., Meireles, A. (2002). Phytochemical characterization of essential oil from *Ocimum selloi*. 74(1), 183–186. www.scielo.br/aabc

Moreno, L.; Bello, R.; Primo, E.; Esplugues, J. (2002). Pharmacological properties of the methanol extract from *Mentha suaveolens* Ehrh. *Phytotherapy Research*, 16(S1), 10–13. https://doi.org/10.1002/ptr.744

Moretti, M.D.L.; Peana, A.T.; Satta, M. (1997). A study on anti-inflammatory and peripheral analgesic action of *Salvia sclarea* oil and its main components. *Journal of Essential Oil Research*, *9*(2), 199–204. doi.org/10.1080/10412905.1997.9699459

Moshrefi-Araghi, A.; Nemati, H.; Azizi, M.; Moshtaghi, N.; Shoor, M. (2021). Association of Genetic Structure and Diversity in Iranian Wild Germplasms of *Mentha longifolia* L. Based on Phenotypical, Biochemical, and Molecular Markers. *Chemistry and Biodiversity*, *18*(4). doi.org/10.1002/cbdv.202001044

Mousavi Nia, A.; Pari Kalantaripour, T.; Basiri, M.; Vafaee, F.; Asadi-Shekaari, M.; Eslami, A.; Darvish Zadeh, F. (2017). *Nepeta Dschuparensis* Bornm Extract Moderates COX-2 and IL-1β Proteins in a Rat Model of Cerebral Ischemia. *Iran Journal of Medicine Sciences*, *42*(2), 179–186.

Mousavi, Z.; Hosaini, A.S.; Asgarpanah, J.; Najafizadeh, P. (2016). Antinociceptive effect of the endemic species *Nepeta depauperata* Benth. *Jundishapur Journal of Natural Pharmaceutical Products*, *11*(2). doi.org/10.17795/jjnpp-25623

Moussaid, M.; Elamrani, A.E.; Bourhim, N.; Benaissa, M. (2011). *In Vivo* Anti-Inflammatory and *In Vitro* Antioxidant Activities of Moroccan Medicinal Plants. In *NPC Natural Product Communications* (Vol. 6, Issue 10).

Murad, H.A.S.; Abdallah, H.M.; Ali, S.S. (2016). *Mentha longifolia* protects against acetic-acid induced colitis in rats. *Journal of Ethnopharmacology*, *190*, 354–361. doi.org/10.1016/j.jep.2016.06.016

Murata, T.; Oyama, K.; Fujiyama, M.; Oobayashi, B.; Umehara, K.; Miyase, T.; Yoshizaki, F. (2013). Diastereomers of lithospermic acid and lithospermic acid B from *Monarda fistulosa* and *Lithospermum erythrorhizon*. *Fitoterapia*, 91, 51–59. https://doi.org/10.1016/j.fitote.2013.08.009

Mustarichie, R.; Moektiwardojo, M.; Apriani Dewi W. (2017). Isolation, Identification, and Characteristic of Essential Oil of Iler (*Plectranthus scutellarioides* (L.) R.Br leaves. *Journal of Pharmaceutical Sciences and Research*, 9(11), 2218-2223.

Mustarichie, R.; Ramdhani, D.; Mekar Saptarini, N. (2022). The anti-inflammatory tablet formulation of *Coleus* (*Plectranthus scutellariodes*) leaves extract using Kollicoat® protect coating. *International Journal of Applied Pharmaceutics*, 14(4), 159-162.

Müzell, D.; Lunardelli, A.; Leite, C.E.; Medeiros Fagundes, R.; Saciura, V.C.; Reichel, C.L.; Rodrigues De Oliveira, J.; Vieira Astarita, L. (2013). Nephroprotective and Anti-inflammatory Effects of Aqueous Extract of *Melissa officinalis* L. on Acetaminophen-induced and Pleurisy-induced Lesions in Rats. *Brazilian Archives of Biology and Technology*, *56*(3), 383–392.

Na, Z.; Li, S.-H.; Xiang, W.; Zhao, A.-H.; Li, C.-M.; Sun, H. (2010), A novel asymmetric ent-Kauranoid dimer from *Isodon enanderianus*. *Chinese Journal of. Chemistry*, 20: 884-886. doi.org/10.1002/cjoc.20020200915

Naceiri Mrabti, H.; Doudach, L.; El Menyiy, N.; ...El Omari, N.; Bouyahya, A. (2018). Phytochemistry and Pharmacology of *Thymus broussonetii* Boiss. *Evidence-based Complementary and Alternative Medicine*, 2021,6350035.

Nahak, G.; Sahu, R. (2014). Immunomodulatory activity of aqueous leaf extract of *Ocimum kilimandscharicum* Guerke in *Clarias batrachus*. *Int. J. Pharm. Sci. Rev. Res*., 28(2), 137–146.

Napagoda, M.; Gerstmeier, J.; Butschek, H.; Lorenz, S.; De Soyza, S.; Qader, M.; Nagahawatte, A.; Wijayaratne, G.B.; Schneider, B.; Svatoš, A.; Jayasinghe, L., Koeberle, A.; Werz, O. 2022. *Plectranthus zeylanicus*: A rich source of secondary metabolites with antimicrobial, disinfectant and anti-inflammatory activities. *Pharmaceuticals* (Basel) 15(4):436. https://doi: 10.3390/ph15040436.

Napagoda, M.; Gerstmeier, J.; Wesely, S.; Popella, S.; Lorenz, S.; Scheubert, K.; Svatoš, A.; Werz, O. 2014. Inhibition of 5-lipoxygenase as anti-inflammatory mode of action of *Plectranthus zeylanicus* Benth and chemical characterization of ingredients by a mass spectrometric approach. *Journal of Ethnopharmacology* 151(2):800-809. https:// doi: 10.1016/j.jep.2013.11.004

Naseef, H.; Al-Maharik, N.; Rabba, A.; Sharifi-Rad, M.; Hawash, M.; Jaradat, N. 2022. Phytochemical characterization and assessments of antimicrobial, cytotoxic and anti-inflammatory properties of *Lavandula coronopifolia* Poir. volatile oil from Palestine. *Arabian Journal of Chemistry* (2022) 15, 104069. doi.org/10.1016/j.arabjc.2022.104069

Neelamkavil, S.V.; Thoppil, J.E. (2013). C-mitotic effect of aqueous extracts of *Isodon coetsa* (Buch.-Ham. ex D. Don) Kudo (Lamiaceae). *Nucleus*, 56(2), 117–122. https://doi.org/10.1007/s13237-013-0083-7.

Neelamkavil, S.V.; Thoppil, J.E. (2014). Toxicological evaluation of polar and nonpolar components of *Isodon coetsa* (Lamiaceae). *Turkish Journal of Botany*, 38, 252-257. doi: 10.3906/bot-1306-4.

Nguyen, D.T.X.; Tran, H.; Schwaiger, S.; Stuppner, H.; Marzocco, S. (2021). Effect of Non-Volatile Constituents of *Elsholtzia ciliata* (Thunb.) Hyl. from Southern Vietnam on Reactive Oxygen Species and Nitric Oxide Release in Macrophages. *Chemistry and Biodiversity*, 18(1), e2000577.

Nicolescu, A.; Babotă, M.; Ilea, M.; Dias, M.I.; Calhelha, R.C.; Gavrilaș, L.; Rocchetti, G.; Crișan, G.; Mocan, A.; Barros, L.; Pârvu, A.E. (2022). Potential therapeutic applications of infusions and hydroalcoholic extracts of Romanian glutinous sage (*Salvia glutinosa* L.). *Frontiers in Pharmacology*, 13. https://doi.org/10.3389/fphar.2022.975800

Nie, L.; Li, R., Huang, J.; Wang, L.; Ma, M.; Huang, C.; Wu, T.; Yan, R.; Hu, X. (2021) Abietane diterpenoids from *Dracocephalum moldavica* L. and their anti-inflammatory activities *in vitro*. *Phytochemistry*. 184:112680. doi: 10.1016/j.phytochem.2021.112680.

Noorbakhsh, F.; Zare, S.; Firuzi, O.; Sakhteman, A.; Chandran, J.N.; Schneider, B.; Jassbi, A.R. (2022). Phytochemical Analysis and Biological Activity of *Salvia compressa* Vent. *Iranian Journal of Pharmaceutical Research*, 21(1). doi.org/10.5812/ijpr-127031

Nworu, C.S.; Akah, P.A. (2015). Anti-inflammatory medicinal plants and the molecular mechanisms underlying their activities. *African Journal of Traditional, Complementary and Alternative Medicines*, 12(S1), 52-61. doi:10.4314/ajtcam.v12i5.3S.

Oh, T.H.; Kim, S.S.; Yoon, W.J.; Kim, J.Y.; Yang, E.J.; Lee, N.H.; Hyun, C.G. 2009. Chemical composition and biological activities of Jeju *Thymus quinquecostatus* essential oils against *Propionibacterium* species inducing acne. *The Journal of General and Applied Microbiology,* 55 I 1 Pages 63-68. doi.org/10.2323/jgam.55.63

Olennikov, D.N.; Chirikova, N.K.; Okhlopkova, Z.M.; Zulfugarov, I.S. (2013). Chemical composition and antioxidant activity of *Tánara Ótó* (*Dracocephalum palmatum* Stephan), a medicinal plant used by the North-Yakutian nomads. *Molecules*, 18(11), 14105-14121. doi: 10.3390/molecules181114105.

Oliveira, A. S.; Rolo, J.; Gaspar, C.; Cavaleiro, C.; Salgueiro, L.; Palmeira-de-Oliveira, R.; Ferraz, C.; Coelho, S.; Pastorinho, M.R.; Sousa, A.C.; Teixeira, J.P.; Martinez-de-Oliveira, J.; Palmeira-de-Oliveira, A. (2022). Chemical characterization and bioactive potential of *Thymus × citriodorus* (Pers.) Schreb. preparations for anti-acne applications: Antimicrobial, anti-biofilm, anti-inflammatory and safety profiles. *Journal of Ethnopharmacology*, *287*. doi.org/10.1016/j.jep.2021.114935

Oliveira, A.S.; Rolo, J.; Gaspar, C.; Ramos, L.; Cavaleiro, C.; Salgueiro, L.; Palmeira-de-Oliveira, R.; Teixeira, J. P.; Martinez-de-Oliveira, J.; Palmeira-de-Oliveira, A. (2023). *Thymus mastichina* (L.) L. and *Cistus ladanifer* L. for skin application: chemical characterization and in vitro bioactivity assessment. *Journal of Ethnopharmacology*, 302. https://doi.org/10.1016/j.jep.2022.115830

Orfali, R.; Siddiqui, N.A.; Alam, P.; Alhowiriny, T.A.; Al-Taweel, A.M.; Al-Yahya, S.; Majrashi, N.M.; Mehmood, R.; Khan, S.I.; Perveen, S. (2018). Biological evaluation of different extracts of aerial parts of *Nepeta deflersiana* and standardization of active extracts using 8-Epi-7-Deoxyloganic acid and ursolic acid by Validated HPTLC method", *Evidence-Based Complementary and Alternative Medicine*, 2018, 8790769, 11. doi: https://doi.org/10.1155/2018/8790769.

Osmakov, D.I.; Koshelev, S.G.; Belozerova, O.A.; ...Grishin, E.V.; Kozlov, S.A. (2015). Biological activity of Sevanol and its analogues. *Russian Journal of Bioorganic Chemistry*, 41(5), pp. 543–547.

Osmakov, D.I.; Koshelev, S.G.; Belozerova, O.A.; ...Grishin, E.V.; Kozlov, S.A. (2015).The Biological Activity of the Sevanol and Its Analogues. *Bioorganicheskaia khimiia*, 41(5), pp. 606–611.

Paksoy, M.Y.; Selvi, S.; Savran, A. (2016). Ethnopharmacological survey of medicinal plants in UlukIşla (Niğde-Turkey). *Journal of Herbal Medicine*, 6(1), pp. 42–48.

Pandey, A.K.; Singh, P.; Tripathi, N.N. (2014). Chemistry and bioactivities of essential oils of some *Ocimum* species: An overview. *Asian Pacific Journal of Tropical Biomedicine*, 4(9), pp. 682–694.

Pandur, E.; Balatinácz, A.; Micalizzi, G.; Mondello, L.; Horváth, A.; Sipos, K.; Horváth, G. (2021). Anti-inflammatory effect of lavender (*Lavandula angustifolia* Mill.) essential oil prepared during different plant phenophases on THP-1 macrophages. *BMC Complementary Medicine and Therapies*, *21*(1). doi.org/10.1186/s12906-021-03461-5

Pandur, E.; Micalizzi, G.; Mondello, L.; Horváth, A.; Sipos, K.; Horváth, G. (2022). Antioxidant and Anti-Inflammatory Effects of Thyme (*Thymus vulgaris* L.) Essential Oils Prepared at Different Plant Phenophases on Pseudomonas aeruginosa LPS-Activated THP-1 Macrophages. *Antioxidants*, *11*(7). doi.org/10.3390/antiox11071330

Pargaien, A. V., Bisht, N., Joshi, H., Pargaien, S., Adhikari, M. (n.d.). Analysis of ethanomedicinally potential extract of *Nepeta cataria*. In Copyright@ EM International (Vol. 26).

Park, S.H.; Koo, H J.; Sung, Y.Y.; Kim, H.K. (2013). The protective effect of *Prunella vulgaris* ethanol extract against vascular inflammation in TNF-α-stimulated human aortic smooth muscle cells. *BMB Reports*, *46*(7), 352–357. doi.org/10.5483/BMBRep.2013.46.7.214

Park, S.; Kim, S.; Suk, K.; Lee, H.; Kwon, T.; Ju, M.; Jeon, H.; Kim, D.; Lim, J.; Shin, T. (2010) *Clinopodium gracile* inhibits mast cell-mediated allergic inflammation: involvement of calcium and nuclear factor-kappaB. *Experimental Biological Medicine*, 235(5), 606-613. doi: 10.1258/ebm.2010.009292. PMID: 20463301

Parsaei, P.; Bahmani, M.; Karimi, M.; Naghdi, N.; Asadi-Samani, M.; Rafieian-Kopaei, M. (2016). A review of analgesic medicinal plants in Iran. *Der Pharmacia Lettre*, 8(2), 43-51. Retrieved from www.scopus.com

Pattanayak, P.; Behera, P.; Das, D.; Panda, S. (2010). *Ocimum sanctum* Linn. A reservoir plant for therapeutic applications: An overview. In *Pharmacognosy Reviews,* 4(7), 95–105. doi.org/10.4103/0973-7847.65323

Pavel, M.; Vostinaru, O.; Mogosan, C.; Ghibu, S. (2011). Phytochemical and pharmacological research on some extracts obtained from Serpylli herba. *Farmacia*, 59(1), 77–84.

Peana, A.T.; Moretti, M.D.L. (2002). Pharmacological activities and applications of *Salvia sclarea* and *Salvia desoleana* essential oils doi:10.1016/S1572-5995(02)80012-6 Retrieved from www.scopus.com

Peana, A.T.; Moretti, M.D.L., Juliano, C. (1999). Chemical composition and antimicrobial action of the essential oils of *Salvia desoleana* and *S. sclarea*. *Planta Medica*, 65(8), 752-754. doi:10.1055/s-2006-960857

Peana, A.; Satta, M. (1993). A preliminary research on essential oils of *Salvia sclarea* l. and *Salvia desoleana* A. et p. *Pharmacological Research*, 27, 25-26. doi:10.1006/phrs.1993.1048

Petrova, M.; Dimitrova, L.; Dimitrova, M.; ...Lazarova, M.; Tasheva, K. (2023). Antitumor and Antioxidant Activities of *In Vitro* Cultivated and Wild-Growing *Clinopodium vulgare* L. Plants. *Plants*, 12(8), 1591

Piccinelli A.C.; Figueiredo de Santana Aquino D.; Morato P.N.; Kuraoka-Oliveira A.M.; Strapasson R.L.; Dos Santos E.P.; Stefanello M.É.; Oliveira R.J.; Kassuya C.A. 2014. Anti-inflammatory and antihyperalgesic activities of ethanolic extract and fruticulin A from *Salvia lachnostachys* leaves in mice. Evidence Based Complementary and Alternative Medicine 2014:835914. https://doi: 10.1155/2014/835914

Piva, R.C.; Verdan, M.H.; Branquinho, L.S.; Kassuya, C.A.L.; Cardoso, C.A.L. (2021). Anti-inflammatory activity and chemical composition of aqueous extract and essential oil from leaves of *Ocimum selloi* Benth. *Journal of Ethnopharmacology*, 275. https://doi.org/10.1016/j.jep.2021.114136

Porres-Martínez, M.; Accame, M.E.C.; Gómez-Serranillos, M.P. (2013). Pharmacological activity of *Salvia lavandulifolia* and chemical components of its essential oil. A review. *Lazaroa* (Vol. 34, pp. 237–254). Universidad Complutense. https://doi.org/10.5209/rev_LAZA.2013.v34.n1.43298

Prescott, T.A.K.; Veitch, N.C.; Simmonds, M.S.J. (2011). Direct inhibition of calcineurin by caffeoyl phenylethanoid glycosides from *Teucrium chamaedrys* and *Nepeta cataria*. *Journal of Ethnopharmacology*, 137(3), 1306–1310. https://doi.org/10.1016/j.jep.2011.07.063

Pudziuvelyte, L.; Liaudanskas, M.; Jekabsone, A.; Sadauskiene, I., (2020). Bernatoniene, J. *Elsholtzia ciliata* (Thunb.) Hyl. extracts from different plant parts: Phenolic composition, antioxidant, and anti-inflammatory activities. *Molecules*, 25(5), 1153.

Qadir, M.I.; Parveen, A.; Abbas, K.; Ali, M. 2016. Analgesic, anti-inflammatory and anti-pyretic activities of *Thymus Linearis*. Pak. J. Pharm. Sci., 29, 2, 2016, pp.591-594.

Qi, J.; Zhang, Q.; Li, L.; ...Wang, N.; Peng, D. (2022). Spectrum-effect relationship between UPLC-Q-TOF-MS fingerprint and anti-AUB effect of *Clinopodium chinense* (Benth.) O. Kuntze. *Journal of Pharmaceutical and Biomedical Analysis*, 217, 114828. https://doi.org/10.1016/j.jpba.2022.114828.

Raal, A.; Paaver, U.; Arak, E.; Orav, A. (2004). Content and composition of the essential oil of *Thymus serpyllum* L. growing wild in Estonia. *Medicina (Kaunas)*, *40*(8), 795–800.

Righi, N.; Boumerfeg, S.; Deghima, A.; ...Coimbra, M.A.; Baghiani, A. (2021). Phenolic profile, safety assessment, and anti-inflammatory activity of *Salvia verbenaca* L. *Journal of Ethnopharmacology*, 272, 113940.

Rocha, J.; Direito, R.; Lima, A.; Mota, J.; Gonçalves, M.; Duarte, M.P.; Solas, J.; Peniche, B.F.; Fernandes, A.; Pinto, R.; Ferreira, R.B.; Sepodes, B.; Figueira, M.E. (2019). Reduction of inflammation and colon injury by a Pennyroyal phenolic extract in experimental inflammatory bowel disease in mice. *Biomedicine and Pharmacotherapy*, *118*. doi.org/10.1016/j.biopha.2019.109351

Rodríguez Basso, A.; Carranza, A.; Zainutti, V.M.; Bach, H.; Gorzalczany, S.B. (2021). Pharmacologycal activity of peperina (*Minthostachys verticillata*) on gastrointestinal tract. *Journal of Ethnopharmacology*, 269. https://doi.org/10.1016/j.jep.2020.113712

Rowshan, V.; Najafian, S. (2020). Polyphenolic contents and antioxidant activities of aerial parts of *Salvia multicaulis* from the Iran flora. *Natural Product Research*, 34(16), 2351-2353. doi:10.1080/14786419.2018.1533832

Roxana, S.P.; Vivian, M.C.; Yazmín, R.C.; Daisy, C.Q.; Victor, G.C.; David, M.D.; . . . Lourdes, A.V. (2012). Effects of a hydroalcoholic extract from cuban population of *Salvia coccinea* L. on a model of acute inflammation. *Revista Electronica De Veterinaria*, 13(6).

Roxo, M.; Zuzarte, M.; Gonçalves, M.J. et al. 2020. Antifungal and anti-inflammatory potential of the endangered aromatic plant *Thymus albicans.* (2020) 10:18859. doi.org/10.1038/s41598-020-75244-w.

Ruiz-Malagón, A.J.; Rodríguez-Sanchez, M.J.; Rodríguez-Sojo, M.J.; Vezza, T.; Pischel, I.; Algieri, F.; Rodríguez-Cabezas, M.E.; Rodríguez-Nogales, A.; Gálvez, J. (2022). Intestinal anti-inflammatory and visceral analgesic effects of a *Serpylli herba* extract in an experimental model of irritable bowel syndrome in rats. *Frontiers in Pharmacology*, *13*. doi.org/10.3389/fphar.2022.967644

Ruiz-Malagón, A.J., Rodríguez-Sojo, M.J., Hidalgo-García, L., Molina-Tijeras, J.A., García, F., Pischel, I., Romero, M., Duarte, J., Diez-Echave, P., Rodríguez-Cabezas, M.E.; Rodríguez-Nogales, A.; Gálvez, J. (2022b). The Antioxidant Activity of *Thymus serpyllum* Extract Protects against the Inflammatory State and Modulates Gut Dysbiosis in Diet-Induced Obesity in Mice. *Antioxidants*, *11*(6). doi.org/10.3390/antiox11061073

Ruppelt, B.M.; Pereira, E.F.; Gonçalves, L.; Pereira, N.A. (1991). Pharmacological screening of plants recommended by folk medicine as anti-snake venom-I. Analgesic and anti-inflammatory activities. *Memorias do Instituto Oswaldo Cruz*, 86(2), 203-5.

Ryu, S.Y.; Oak, M.-H.; Yoon, S.-K.; Cho, D.-I.; Yoo, G.-S.; Kim, T.-S.; Kim, K.-M.; Thieme, G.; Stuttgart, V.; York, N. (2000). Anti-Allergic and Anti-Inflammatory Triterpenes from the Herb of *Prunella vulgaris*. *Planta Medica*, *66*, 358.

Sahrial, I.; Solfaine, R. (2019). *Coleus amboinicus* extract increases transforming growth factor-1β expression in Wistar rats with cisplatin-induced nephropathy. *Veterinary World*, 12(8): 1346-1351. doi: 10.14202/vetworld.2019.1346-1351.

Salehi, B.; Mishra, A.P.; Shukla, I.; Sharifi-Rad, M.; Contreras, M. del M.; Segura-Carretero, A.; Fathi, H.; Nasrabadi, N.N.; Kobarfard, F.; Sharifi-Rad, J. (2018). Thymol, thyme, and other plant sources: Health and potential uses. *Phytotherapy Research,* (Vol. 32, Issue 9, pp. 1688–1706). John Wiley and Sons Ltd. doi.org/10.1002/ptr.6109

Sánchez-Tito, J.; Cartagena-Cutipa, R.; Flores-Valencia, E. (2021). Chemical composition and antimicrobial activity of essential oil from *Minthostachys mollis* against oral pathogens. *Revista Cubana de Estomatología*, 58(4), e3647. www.revestomatologia.sld.cu/index.php/est/article/view/3647

Santana, F.R.; de Santana Souza, M.T.; Camargo, E. A.; Silva, J.A. (2022). Anti-inflammatory and antinociceptive effects of a pectinolide-enriched fraction from *Mesosphaerum pectinatum* (L.) Kuntze. *Journal of Ethnopharmacology*, 115916. doi.org/10.1016/j.jep.2022.115916

Saoulajan, C.; Boujida, N.; El Mihyaoui, A.; El Baakili, A.; Alshahrani, M.M.; Lee, L. -.; Bouyahya, A. (2022). Phytochemistry, pharmacological investigations, industrial applications, and encapsulation of *Thymbra capitata* L., a review. *Trends in Food Science and Technology*, 129, 463-491. doi:10.1016/j.tifs.2022.10.014.

Sarikurkcu, C.; Sabih Ozer, M.; Eskici, M.; Tepe, B.; Can, S.; Mete, E., 2010. Essential oil composition and antioxidant activity of *Thymus longicaulis* C. Presl subsp. *longicaulis* var. l*ongicaulis*. *Food Chem. Toxicol.* 48, 1801–1805.

Semaoui, R.; Ouafi, S.; Machado, S.; Barros, L.; Ferreira, I.C.F.R.; Oliveira, M.B.P.P. (2021). Infusion of aerial parts of *Salvia chudaei* Batt. Trab. from Algeria: Chemical, toxicological and bioactivities characterization. *Journal of Ethnopharmacology*, *280*. doi.org/10.1016/j.jep.2021.114455

Shabbir, A.; Batool, S.A.; Basheer, M.I.; Shahzad, M.; Sultana, K.; Tareen, R.B.; Iqbal, J.; Saeed-ul-Hassan. (2018). *Ziziphora clinopodioides* ameliorated rheumatoid arthritis and inflammatory paw edema in different models of acute and chronic inflammation. *Biomedicine and Pharmacotherapy*, *97*, 1710–1721. doi.org/10.1016/j.biopha.2017.11.118

Shaikh, R.; Pund, M.; Dawane, A.; Iliyas, S. (2014). Evaluation of anticancer, antioxidant, and possible anti-inflammatory properties of selected medicinal plants used in Indian traditional medication. *Journal of Traditional and Complementary Medicine*, 4(4), 253-257.

Shan, M.; Jiang, Y.; Fu, Y.; ...Zhang, L.; Wu, Q. (2021). A review of the botany, traditional uses, phytochemistry and pharmacology of *Nepeta tenuifolia* Briq. *Phytochemistry Reviews*, 20(5), pp. 991–1012.

Shanaida, M.; Hudz, N.; Jasicka-Misiak, I.; Wieczorek, P.P. (2021). Polyphenols and Pharmacological Screening of a *Monarda fistulosa* L. dry Extract Based on a Hydrodistilled Residue By-Product. *Frontiers in Pharmacology*, 12. https://doi.org/10.3389/fphar.2021.563436

Shanaida, M.; Jasicka-Misiak, I.; Bialon, M.; Korablova, O.; Wieczorek, P.P. (2021). Chromatographic profiles of the main secondary metabolites in the *Monarda fistulosa* l. Aerial part. Research Journal of Pharmacy and Technology, 14(4), 2179–2184. https://doi.org/10.52711/0974-360X.2021.00385

Shanmugam, G.; Sundaramoorthy, A.; Shanmugam, N. (2021). Biosynthesis of silver nanoparticles from leaf extract of *Salvia coccinea* and its effects of anti-inflammatory potential in human monocytic THP-1 cells. *ACS Applied Bio Materials*, 4(12), 8433-8442. doi:10.1021/acsabm.1c00963

Sharifabad, A.H.; Sadraei, H.; Hashemnia, M.; Sajjadi, S.E.; Mirdamadi, Z. (2021). Effect of hydroalcoholic and aqueous extracts of *Dracocephalum kotschyi* on bleomycin induced pulmonary fibrosis. *Journal of HerbMed Pharmacology*, 10(2), 209–217. https://doi.org/10.34172/jhp.2021.23

Sharifi-Rad, M.; Berkay Yılmaz, Y.; Antika, G.; Salehi, B.; Tumer, T. B.; Kulandaisamy Venil, C.; Das, G.; Patra, J. K.; Karazhan, N.; Akram, M.; Iqbal, M.; Imran, M.; Sen, S.; Acharya, K.; Dey, A.; Sharifi-Rad, J. (2021). Phytochemical constituents, biological activities, and health-promoting effects of the genus *Origanum*. *Phytotherapy Research,* (Vol. 35, Issue 1, pp. 95–121). John Wiley and Sons Ltd. doi.org/10.1002/ptr.6785

Shehadeh, M. B.; Sosa, S.; Suaifan, Darwish, R. M.; Giangaspero, A.; Vassallo, A.; Lepore, L.; Oran, S.; Hammad, H.; Tubaro, A.; De Tommasi, N.; Loggia, R. D. (2014). Topical anti-inflammatory potential of six *Salvia* species grown in Jordan. *Jordan Journal of Pharmaceutical Sciences*, 7(2), 153-161. doi:10.12816/0026806

Shen, W.; Anwaier, G.; Cao, Y.; Lian, G.; Chen, C.; Liu, S.; Tuerdi, N.; Qi, R. (2019). Atheroprotective Mechanisms of Tilianin by Inhibiting Inflammation Through Down-Regulating NF-κB Pathway and Foam Cells Formation. *Frontiers in Physiology*, *10*. doi.org/10.3389/fphys.2019.00825

Sheychenko, O.P.; Sheychenko, V.I.; Goryainov, S.V.; Zvezdina, E.V.; Kurmanova, E.N.; Ferubko, E.V.; Uyutova, E.V.; Potanina, O.G.; Fadi, K. (2021). Chemical composition and biological activity of the dry extract "Rosmatin" from the herb of *Dracocephalum moldavica* L. *Khimiya Rastitel'nogo Syr'ya*, *3*, 253–264. doi.org/10.14258/JCPRM.2021039161

Shi, Q.Q.; Dang, J.; Wen, H.X.; Yuan, X.; Tao, Y.D.; Wang, Q.L. (2016). Anti-hepatitis, antioxidant activities and bioactive compounds of *Dracocephalum heterophyllum* extracts. *Botanical Studies*, 57(1):16. doi: 10.1186/s40529-016-0133-y.

Shimizu, M.; Shogawa, H.; Matsuzawa, T.; ...Suzuki, S.; Berganza, A. (1990). Anti-inflammatory Constituents of Topically Applied Crude Drugs: IV: Constituents And Anti-inflammatory Effect of Paraguayan Crude Drug "Alhucema" (*Lavandula latifolia* Vill.). *Chemical and Pharmaceutical Bulletin*, 38(8), pp. 2283–2284

Shin, T.Y.; Kim, S.H.; Choi, C.H.; Shin, H.Y.; Kim, H.M. (2004). *Isodon japonicus* decreases immediate-type allergic reaction and tumor necrosis factor-α production. International Archives of Allergy and Immunology, 135(1), 17–23. https://doi.org/10.1159/000080038

Shin, T.Y.; Kim, S.H.; Suk, K.; Ha, J.H.; Kim, I.K.; Lee, M.G.; Jun, C.D.; Kim, S.Y.; Lim, J.P.; Eun, J.S.; Shin, H.Y.; Kim, H.M. (2005). Anti-allergic effects of *Lycopus lucidus* on mast cell-mediated allergy model. *Toxicology and Applied Pharmacology*, *209*(3), 255–262. doi.org/10.1016/j.taap.2005.04.011

Shipa, S.J.; Khandokar, L.; Bari, M.S.; ...Haque, M.A.; Mohamed, I.N. (2022). An insight into the anti-ulcerogenic potentials of medicinal herbs and their bioactive metabolites. *Journal of Ethnopharmacology*, 293, 115245.

Singh, D.; Chaudhuri, P.K. (2018). A review on phytochemical and pharmacological properties of Holy basil (*Ocimum sanctum* L.). In *Industrial Crops and Products* (Vol. 118, pp. 367–382). Elsevier B.V. doi.org/10.1016/j.indcrop.2018.03.048

Singh, M.K.; Gidwani, B.; Gupta, A.; Dhongade, H.; Kaur, C.D.; Kashyap, P.P.; Tripathi, D.K. (2015). A review of the medicinal plants of genus *Orthosiphon* (Lamiaceae). *International Journal of Biological Chemistry*, *9*(6), 318–331. doi.org/10.3923/ijbc.2015.318.331

Sitarek, P.; Kowalczyk, T.; Synowiec, E.; Merecz-Sadowska, A.; Bangay, G.; Princiotto, S.; Sliwinski, T.; Rijo, P. 2022. An evaluation of the novel biological properties of diterpenes isolated from *Plectranthus ornatus* Codd. In vitro and in silico. Cells 11, 3243. https://doi.org/10.3390/cells11203243.

Slighoua, M.; Mahdi, I.; Ez-zahra Amrati, F.; Boucetta, N.; Cristo, F.; di, Boukhira, S.; el youbi el Hamsas, A.; Tattou, M.I.; Grafov, A.; Bari, A.; Bousta, D. (2022). Pharmacological effects of *Lavandula officinalis* Chaix and its polyphenols: Focus on their in vivo estrogenic and anti-inflammatory properties. *South African Journal of Botany*, *146*, 354–364. doi.org/10.1016/j.sajb.2021.11.014

Solfaine, R.; Muniroh, L.; Sadarman, A.; Irawan, A. (2021). Anti-inflammatory effect of *Coleus amboinicus* leaves extract on uric acid-induced nephrotoxicity rats. *Advances in Animal and Veterinary Sciences*, 9(10), 1553-1558. doi:10.17582/journal.aavs/2021/9.10.1553.1558.

Sonboli, A.; Kanani, M.R.; Yousefzadi, M.; Mojarad, M. (2009). Chemical composition and antibacterial activity of the essential oil of *Salvia hydrangea* from two localities of Iran. *Journal of Medicinal Plants*, 8(30), 20-28+172.

Soosani, B Sazegar, H. (2018). Effects of *Thymus daenensis* on inflammatory factors and liver toxicity induced by thioacetamide in rats. *J Herbmed Pharmacol* 7(1): 56-60. doi: 10.15171/jhp.2018.10

Sosa, S.; Altinier, G.; Politi, M.; Braca, A.; Morelli, I.; Della Loggia, R. 2005. Extracts and constituents of *Lavandula multifida* with topical anti-inflammatory activity. *Phytomedicine* 12 (2005) 271–277. doi:10.1016/j.phymed.2004.02.007.

Souri, F.; Rakhshan, K.; Erfani, S.; Azizi, Y.; Maleki, S.; Aboutaleb, N. (2019). Natural lavender oil (*Lavandula angustifolia*) exerts cardioprotective effects against myocardial infarction by targeting inflammation and oxidative stress. *Inflammopharmacology*, 27(4), 799–807.

Sousa, P.J.; Linard, C.; Azevedo-Batista, D.; Oliveira, A.C.; Coelho-de-Souza, A.N.; Leal-Cardoso, J.H. 2009. Antinociceptive effects of the essential oil of *Mentha x villosa* leaf and its major constituent piperitenone oxide in mice. *Brazilian Journal of Medical and Biological Research*, (2009) 42: 655-659.

Sudaramoorthy, A.; Shanmugam, G.; Shanmugam, N. (2021). Inhibitory effect of *Salvia coccinea* on inflammatory responses through NF-κB signaling pathways in THP-1 cells and acute rat *diabetes mellitus*. *Acta Histochemica*, 123(5) doi:10.1016/j.acthis.2021.151735

Sun J.; Sun P.; Kang C.; Zhang L.; Guo L.; Kou Y. 2022. Chemical composition and biological activities of essential oils from six Lamiaceae folk medicinal plants. *Frontiers in Plant Science* 13:919294. https://doi: 10.3389/fpls.2022.919294.

Sun, Z.; Wang, H.; Wang, J.; Zhou, L.; Yang, P. (2014). Chemical composition and anti-inflammatory, cytotoxic and antioxidant activities of essential oil from leaves of *Mentha piperita* grown in China. *PLoS ONE*, *9*(12). doi.org/10.1371/journal.pone.0114767

Süntar, I.; Nabavi, S.M.; Barreca, D.; Fischer, N.; Efferth, T. (2018). Pharmacological and chemical features of *Nepeta* L. genus: Its importance as a therapeutic agent. *Phytotherapy Research*, 32, 185–198. doi: 10.1002/ptr.5946.

Taarit, M.B.; Msaada, K.; Hosni, K.; Chahed, T.; Marzouk, B. (2010). Essential oil composition of *Salvia verbenaca* L. growing wild in Tunisia. *Journal of Food Biochemistry*, 34(1), pp. 142–151.

Tadesse, S.; Mazumder, A.; Bucar, F.; Veeresham, C. Asres, K. (2011). Chemical composition and biological activities of the essential oil of *Plectranthus caninus* Roth. *Pharmacognosy Journal*, *3*(26), 1–7. doi.org/10.5530/pj.2011.26.1

Taheri, S.; Khalifeh, S.; Shajiee, H.; Ashabi, G. (2021). Dietary uptake of *Salvia macilenta* extract improves Nrf2 antioxidant signaling pathway and diminishes inflammation and apoptosis in amyloid beta-induced rats. *Molecular Biology Reports*, *48*(12), 7667–7676. doi.org/10.1007/s11033-021-06772-5.

Tang, Y.Q.N.; Deng, J.; Li, L.; Yan, J.; Lin, L.M.; Li, Y. M.; Lin, Y.; Xia, B.H. (2022). Essential oil from *Prunella vulgaris* L. as a valuable source of bioactive constituents: *In vitro* anti-bacterial, anti-viral, immunoregulatory, anti-inflammatory, and chemical profiles. *South African Journal of Botany*, *151*, 614–627. doi.org/10.1016/j.sajb.2022.10.029

Taşkın, T.; Emin Çam, M.; Bulut, G.; Nur Hazar-Yavuz, A.; Kabasakal, L.; Bitiş, L. (2018) Antioxidant and anti-inflammatory activities of *Phlomis pungens* and *Coridothymus capitatus*. *Marmara Pharmaceutical Journal*: 22(1), 80-85. dx.doi.org/10.12991/mpj.2018.44

Taşkın, T.; Çam, M.E.; Taşkın, D. et al. 2019. *In vitro* and *In vivo* biological activities and phenolic characterization of *Thymus praecox* subsp. *skorpilii* var. *skorpilii*. *Journal of Food Measurement and Characterization* (2019) 13:536–544 https://doi.org/10.1007/s11694-018-9967-1

Taskin, T.; Dogan, M.; Cam, M. E.; Sahin, T.; Senkardes, I. (2020). In vitro anti-urease, antioxidant, anticholinesterase, cytotoxic and in vivo anti-inflammatory potential of *Satureja cuneifolia* Ten. *Notulae Scientia Biologicae*, 12(2), 222–232. doi.org/10.15835/nsb12210687

Tepe, B.; Cakir, A.; Sihoglu Tepe, A. (2016). Medicinal Uses, Phytochemistry, and Pharmacology of *Origanum onites* (L.): A Review. *Chemistry and Biodiversity* (Vol. 13, Issue 5, pp. 504–520). Wiley-VCH Verlag. https://doi.org/10.1002/cbdv.201500069

Thawkar, B.; Jawarkar, A.G.; Kalamkar, P.; Pawar, K.; Kale, M.K. (2016). Phytochemical and pharmacological review of *Mentha arvensis*. *International Journal of Green Pharmacy*, *10*(2), 71–76. www.researchgate.net/publication/316256954

Tsioutsiou, E.E.; Amountzias, V.; Vontzalidou, A.; Dina, E.; Stevanović, Z.D.; Cheilari, A.; Aligiannis, N. (2022). Medicinal plants used traditionally for skin related problems in the south balkan and east mediterranean Region—A review. *Frontiers in Pharmacology*, 13 doi:10.3389/fphar.2022.936047

Tubon, I.; Zannoni, A.; Bernardini, C.; Salaroli, R.; Bertocchi, M.; Mandrioli, R.; Vinueza, D.; Antognoni, F.; Forni, M. 2019. *In vitro* anti-inflammatory effect of *Salvia sagittata* ethanolic extract on primary cultures of porcine aortic endothelial cells. *Oxidative Medicine and Cellular Longevity* 2019:6829173. https://doi: 10.1155/2019/6829173

Tubon, I.; Bernardini, C.; Antognoni, F.; Mandrioli, R.; Potente, G.; Bertocchi, M.; Vaca, G.; Zannoni, A.; Salaroli, R.; Forni, M. (2020). *Clinopodium tomentosum* (Kunth) Govaerts Leaf Extract Influences in vitro Cell Proliferation and Angiogenesis on Primary Cultures of Porcine Aortic Endothelial Cells. *Oxidative Medicine and Cellular Longevity*, *2020*. doi.org/10.1155/2020/2984613

Ueda, H.; Yamazaki, C.; Yamazaki, M. (2002). Luteolin as an anti-inflamamtory and anti-allergic constituent of *Perilla frutescens*. *Biological Pharmaceutical Bulletin*, *25*(9), 1197–1202.

Ulikhanyan, G.; Galstyan, H.; Dumanyan, K.; Beglaryan, M.; Poghosyan, G.; Shekoyan, V.; Ananikyan, H.; Ananikyan, V.; Goginyan, V.; Ulikhanyan, G.; Chichoyan, N.; Taschyan, S. (2022). The study of the biological activities of *Ziziphora clinopodioides*. *Brazilian Journal of Pharmaceutical Sciences*, *58*. doi.org/10.1590/s2175-97902022e19331

Ustuner, O.; Anlas, C.; Bakirel, T.; ...Donmez, C.; Koca-Caliskan, U. (2019). *In vitro* evaluation of antioxidant, anti-inflammatory, antimicrobial and wound healing potential of *Thymus sipyleus* Boiss. subsp. *rosulans* (Borbas) Jalas. *Molecules*, 24(18), 3353.

Vairinhos, J.; Miguel, M.G. (2020). Essential oils of spontaneous species of the genus *Lavandula* from Portugal: A brief review. *Zeitschrift fur Naturforschung - Section C Journal of Biosciences*, 75(78), pp. 233–245.

Vazirian, F.; Samadi, S.; Abbaspour, M.; ...Mohammadpour, A.H.; Emami, S.A. (2022). Evaluation of the efficacy of *Thymus kotschyanus* extract as an additive treatment in patients with ulcerative colitis: a randomized double-blind placebo-controlled trial. *Inflammopharmacology*, 30(6), pp. 2145–2152

Verano, J.; González-Trujano, M. E.; Déciga-Campos, M.; Ventura-Martínez, R.; Pellicer, F. (2013). Ursolic acid from *Agastache mexicana* aerial parts produces antinociceptive activity involving TRPV1 receptors, cGMP and a serotonergic synergism. *Pharmacology Biochemistry and Behavior*, 110, 255–264. https://doi.org/10.1016/j.pbb.2013.07.020

Vergara-Martínez, V.M.; Estrada-Soto, S.E.; Valencia-Díaz, S.; Garcia-Sosa, K.; Peña-Rodríguez, L.M.; de Jesús Arellano-García, J.; Perea-Arango, I. (2021). Methyl jasmonate enhances ursolic, oleanolic and rosmarinic acid production and sucrose induced biomass accumulation, in hairy roots of *Lepechinia caulescens*. *PeerJ*, *9*, 1–18. doi.org/10.7717/peerj.11279

Vijayan, C.; Murugan, K.; Reji, S.; Nair, G. (2017). Screening biological activities of *Orthoshiphon aristatus*: some observations. *International Journal of Advanced Research*, *5*(12), 757–764. doi.org/10.21474/IJAR01/6016

Villaseñor, I. M.; Angelada, J.; Canlas, A. P.; Echegoyen, D. (2002). Bioactivity studies on β-sitosterol and its glucoside. *Phytotherapy Research*, *16*(5), 417–421. doi.org/10.1002/ptr.910

Vladimir-Knežević, S.; Cvijanović, O.; Blažeković, B.; Kindl, M.; Štefan, M.B.; Domitrović, R. (2015). Hepatoprotective effects of *Micromeria croatica* ethanolic extract against CCl4-induced liver injury in mice. *BMC Complementary and Alternative Medicine*, *15*(1). doi.org/10.1186/s12906-015-0763-8

Vogl, S.; Picker, P.; Mihaly-Bison, J.; Fakhrudin, N.; Atanasov, A.G.; Heiss, E.H.; Wawrosch, C.; Reznicek, G.; Dirsch, V.M.; Saukel, J.; Kopp, B. (2013). Ethnopharmacological *in vitro* studies on Austria's folk medicine - An unexplored lore *in vitro* anti-inflammatory activities of 71 Austrian traditional herbal drugs. *Journal of Ethnopharmacology*, *149*(3), 750–771. doi.org/10.1016/j.jep.2013.06.007

Vulganová, K.; Maliar, T.; Maliarová, M.; Nemeček, P.; Viskupičová, J.; BaláŽová, A.; Sokol, J. (2019). Biologically valuable components, antioxidant activity and proteinase inhibition activity of leaf and callus extracts of *Salvia* sp. *Nova Biotechnologica et Chimica*, 18(1), 25-36. doi:10.2478/nbec-2019-0004

Wan, J.; Liu, M.; Jiang, H.Y.; Yang, J.; Du, X.; Li, X.N.; Wang, W.G.; Li, Y.; Pu, J.X.; Sun, H.D. (2016). Bioactive ent-kaurane diterpenoids from *Isodon serra*. *Phytochemistry*, 130, 244–251. doi.org/10.1016/j.phytochem.2016.05.014

Wang, M.; Dai, H.; Li, X.; ...Wang, L.; Xue, M. (2010). Structural elucidation of metabolites of tanshinone I and its analogue dihydrotanshinone I in rats by HPLC-ESI-MSn. *Journal of Chromatography B: Analytical Technologies in the Biomedical and Life Sciences*, 878(13-14), pp. 915–924.

Wang, M.; Wang, Y.; Shi, L.; Lu, Y.; Zhu, H.; Liang, L.; Sun, Z. L. (2022). A New Phenylpropanoid-Substituted Flavan-3-ol from Aerial Part of *Mentha longifolia*. *Chemistry of Natural Compounds*, *58*(2), 237–239. doi.org/10.1007/s10600-022-03649-2

Wang, X.; Cheng, K.; Liu, Z.; Sun, Y.; Zhou. L.; Xu. M.; Dai, X.; Xiong, Y.; Zhang, H. (2021). Bioactive constituents of *Mosla chinensis*-cv. Jiangxiangru ameliorate inflammation through MAPK signaling pathways and modify intestinal microbiota in DSS-induced colitis mice. *Phytomedicine* 93,153804. doi: 10.1016/j.phymed.2021.153804.

Wang, Y.Y.; Lin, S.Y.; Chen, W.Y.; Liao, S.L.; Wu, C.C.; Pan, P.H.; Chou, S.T.; Chen, C.J. (2017). *Glechoma hederacea* extracts attenuate cholestatic liver injury in a bile duct-ligated rat model. *Journal of Ethnopharmacology*, *204*, 58–66. doi.org/10.1016/j.jep.2017.04.011

Wang, Y.; Shao, Z.; Song, C.; ...Zhou, G.; Meng, D. (2023). *Clinopodium chinense* Kuntze ameliorates dextran sulfate sodium-induced ulcerative colitis in mice by reducing systematic inflammation and regulating metabolism. *Journal of Ethnopharmacology*, 309, 116330.

Wang, Z.; Hwang, S.H.; Guillen Quispe, Y.N.; Gonzales Arce, P.H.; Lim, S.S. (2017). Investigation of the antioxidant and aldose reductase inhibitory activities of extracts from Peruvian tea plant infusions. *Food Chemistry*, 231, 222–230. http://dx.doi.org/10.1016/j.foodchem.2017.03.107.

Wu, H.-Y.; Wang, W.-G.; Du, X.; Yang, J.; Pu, J.-X.; Sun, H.-D. (2015). Six new cytotoxic and anti-inflammatory 11, 20-epoxy-ent-kaurane diterpenoids from *Isodon wikstroemioides*. *Chinese Journal of Natural Medicines*, 13(5), 383–389.

Wu, H.-Y.; Wang, W.-G.; Jiang, H.-Y.; Du, X.; Li, X.-N.; Pu, J.-X.; Sun, H.-D. (2014). Cytotoxic and anti-inflammatory ent-kaurane diterpenoids from *Isodon wikstroemioides*. *Fitoterapia*, 98, 192–198. http://dx.doi.org/10.1016/j.fitote.2014.08.012.

Wu, S.J.; Chan, H.H.; Hwang, T.L.; Qian, K.; Morris-Natschke, S.; Lee, K.H.; Tian-Shung Wu, T.S. 2010. Salviatalin A and salvitrijudin A, two diterpenes with novel skeletons from roots of *Salvia digitaloides* and anti-inflammatory evaluation. *Tetrahedron Letters* 51(32):4287-4290. DOI: https://10.1016/j.tetlet.2010.06.048.

Wu, Y.; Wang, Y.; Liu, X.; Jiang, L.; Guli, A.; Sailike, J.; Sun, X.; Abuduwaili, N.; Tuoliuhan, H.; Maney, K.; Nabi, X. (2020). *Ziziphora clinopodioides* flavonoids based on network pharmacology attenuates atherosclerosis in rats induced by high-fat emulsion combined with vitamin D3 by down-regulating VEGF/AKT/NF-κB signaling pathway. *Biomedicine and Pharmacotherapy*, 129. doi.org/10.1016/j.biopha.2020.110399.

Xia, F.; Wu, C.Y.; Yang, X.W.; Li, X.; Xu, G. 2015. Diterpenoids from the Roots of *Salvia yunnanensis*. *Nat. Prod. Bioprospect.* (2015) 5:307–312. doi.10.1007/s13659-015-0080-4.

Xiang, Z.; Wu, X.; Liu, X.; Jin, Y. (2014). Glaucocalyxin A: a review. *Natural Product Research*, 28:24, 2221-2236. https://doi.org/10.1080/14786419.2014.934235.

Xie, Q.; Wang, Y.; Zou, G.L. (2022). Protective effects of lavender oil on sepsis-induced acute lung injury via regulation of the NF-κB pathway. *Pharmaceutical Biology*, *60*(1), 968–978. doi.org/10.1080/13880209.2022.2067570

Xing, H.; An, L.; Song, Z.; Li, S.; Wang, H.; Wang, C.; Zhang, J.; Tuerhong, M.; Abudukeremu, M.; Li, D.; Lee, D.; Xu, J.; Lall, N.; Guo, Y. (2020). Anti-Inflammatory ent-Kaurane Diterpenoids from *Isodon serra*. *Journal of Natural Products*, 83(10), 2844–2853. https://doi.org/10.1021/acs.jnatprod.9b01281

Xing, J.; Peng, K.; Cao, W.; Lian, X.; Wang, Q.; Wang, X. (2013). Effects of total flavonoids from *Dracocephalum moldavica* on the proliferation, migration, and adhesion molecule expression of rat vascular smooth muscle cells induced by TNF-α. *Pharmaceutical Biology*, *51*(1), 74–83. doi.org/10.3109/13880209.2012.711839.

Xu, G.; Peng, L.Y.; Lu, L.; Weng, Z.Y.; Zhao, Y.; Li, X.L.; Zhao, Q.S.; Sun, H.D. 2005. Two New Abietane Diterpenoids from *Salvia yunnanensis*. *Planta Med* 2006; 72: 84±86. DOI 10.1055/s-2005-873184.

Yan, B.C.; Wang, W.G.; Hu, D.B.; Sun, X.; Kong, L.M.; Li, X.N.; Du, X.; Luo, S.H.; Liu, Y.; Li, Y.; Sun, H.D.; Pu, J.X. (2016). Phomopchalasins A and B, Two Cytochalasans with Polycyclic-Fused Skeletons from the Endophytic Fungus *Phomopsis sp*. *Organic Letters*, *18*(5), 1108–1111. doi.org/10.1021/acs.orglett.6b00214

Yan, D. (2016). Substitutability of *Prunella vulgaris* L. stem, leaf and ear based on HPLC-ESI-MS analysis and anti-inflammatory and antioxidant activity. *Chinese Pharmaceutical Journal*, *24*, 792–797. pesquisa.bvsalud.org/portal/resource/pt/wpr-859105

Yang, E.-L.; Hou, Y.; Ma, G.-X.; Zou, L.-J.; Xu, X.-D.; Wu, H.-F.; Yang, J.-S.; Wei, H.-W.; Fan, C.-Z.; Sun, Z.-C.; Shi, L.-L. (2022). Abietane-Type diterpenoids from *Nepeta bracteata* Benth. and their anti-inflammatory activity. *Frontiers in Chemistry*, 10, 944972. doi: 10.3389/fchem.2022.944972.

Yang, F.; Pu, H.-Y.; Yaseen, A., Chen, B., Li, F. Gu, Y.-Ch., Shen, X.-F., Wang, M.-K. Guo, D.-L., Wang, L. (2021). Terpenoid and phenolic derivatives from the aerial parts of *Elsholtzia rugulosa* and their anti-inflammatory activity. *Phytochemistry*, 181: 112543. https://doi.org/10.1016/j.phytochem.2020.112543.

Yang, N.; Zou, C.; Luo, W.; Xu, D.; Wang, M.; Wang, Y.; Wu, G.; Shan, P.; Liang, G. (2022). Sclareol attenuates angiotensin II -induced cardiac remodeling and inflammation via inhibiting MAPK signaling. *Phytotherapy Research*, 1-14 doi.org/10.1002/ptr.7635

Yang, Y.; Wang, Z.-P.; Gao, S.-H.; ...Zhong, R.-Q.; Chen, W.-S. (2017). The effects of *Salvia przewalskii* total phenolic acid extract on immune complex glomerulonephritis. *Pharmaceutical Biology*, 55(1), pp. 2153–2160.

Yang, Z.; Qi, J.; Ping, D.; Sun, X.; Tao, Y.; Liu, C.; Peng, Y. (2022). *Salvia miltiorrhiza* in thorax and abdominal organ fibrosis: A review of its pharmacology. *Frontiers in Pharmacology* (Vol. 13). *Frontiers Media S.A.* doi.org/10.3389/fphar.2022.999604.

Yazbek, P.B.; Tezoto, L.; Cassas, F.; Rodrigues, E. 2016. Review Plants used during maternity, menstrual cycle and other women⊗ health conditions among Brazilian cultures. *Journal of Ethnopharmacology* 179 (2016) 310–331. dx.doi.org/10.1016/j.jep.2015.12.054

Yi, W.; Chen, R.; Xie, F.; Xu, C.; Tian, W. (2022). Anti-inflammatory and immunomodulatory properties of *Mentha piperita* green-formulated gold nanoparticles and its effect on ovalbumin-induced asthma and lung pathological changes in rats. *Journal of Experimental Nanoscience*, *17*(1), 163–172. doi.org/10.1080/17458080.2022.2033730

Yin, Q.-Q.; Liu, C.-X.; Wu, Y.-L.; Wu, S.-F.; Wang, Y.; Zhang, X.; Hu, X.-J.; Pu, J.-X.; Lu, Y.; Zhou, H.-C.; Wang, H.-L.; Nie, H.; Sun, H.-D.; Chen, G.-Q. (2013). Preventive and Therapeutic Effects of Adenanthin on Experimental Autoimmune Encephalomyelitis by Inhibiting NF-κB Signaling. *The Journal of Immunology*, *191*(5), 2115–2125. doi.org/10.4049/jimmunol.1203546

Yu, Y.; Xing, N.; Xu, X.; ...Sun, G.; Sun, X. (2019). Tournefolic acid B, derived from *Clinopodium chinense* (Benth.) Kuntze protects against myocardial ischemia/reperfusion injury by inhibiting endoplasmic reticulum stress-regulated apoptosis via PI3K/AKT pathways. *Phytomedicine*, 52, pp. 178–186.

Yu, Z.-Y.; Liang, Y.-G.; Xiao, H.; Shan, Y.-J.; Dong, B.; Huang, R.; Fu, Y.-L.; Zhao, Z.-H.; Liu, Z.-Y.; Zhao, Q.-S.; Wang, S.-Q.; Chen, J.-P.; Mao, B.-Z.; Cong, Y.-W. (2007). Melissoidesin G, a diterpenoid purified from *Isodon melissoides*, induces leukemic-cell apoptosis through induction of redox imbalance and exhibits synergy with other anticancer agents. *International Journal of Cancer*, 121, 2084–2094. doi: 10.1002/ijc.22945.

Yuan, F.; Liu, R.; Hu, M.; Sun, Y.; He, J. JAX2, an ethanol extract of *Hyssopus cuspidatus* Boriss., can prevent bronchial asthma by inhibiting MAPK/NF-κB inflammatory signaling. *Phytomedicine*, 57, pp. 305–314.

Zam, W.; Quispe, C.; Sharifi-Rad, J.; López, M.D.; Schoebitz, M.; Martorell, M.; Sharopov, F., Fokou, P.V.T.; Mishra, A.P.; Chandran, D.; Kumar, M.; Chen, J.T.; Pezzani, R. (2022). An Updated Review on The Properties of *Melissa officinalis* L.: Not Exclusively Anti-anxiety. In *Frontiers in bioscience* 14(2). doi.org/10.31083/j.fbs1402016

Zargar, O.A.; Bashir, R.; Ganie, S.A.; Hamid, R. (2019). The effect of *Elsholtzia densa* methanolic extract modulates inflammation in vitro and in vivo. *Oriental Pharmacy and Experimental Medicine*, *19*(1), 49–58. doi.org/10.1007/s13596-018-0346-6.

Zarshenas, M.M.; Krenn, L. (2015). Phytochemical and pharmacological aspects of *Salvia mirzayanii* Rech. f. Esfand. *Journal of Evidence-Based Complementary and Alternative Medicine*, 20(1), 65–72. https://doi.org/10.1177/2156587214553938

Zengin, G.; Ferrante, C.; Orlando, G.; Zheleva-Dimitrova, D.; Gevrenova, R.; Recinella, L.; Chiavaroli, A.; Leone, S.; Brunetti, L.; Aumeeruddy, M.Z.; Aktumsek, A.; Mahomoodally, M.F. Angelini, P.; Covino, S.; Venanzoni, R.; Tirillini, B.; Menghini, L. (2019). Chemical profiling and pharmaco-toxicological activity of *Origanum sipyleum* extracts: Exploring for novel sources for potential therapeutic agents. *Journal of Food Biochemistry*, 43, e13003. doi: https://doi.org/10.1111/jfbc.13003.

Zhang, J.; Yan, X.; Xu, S.; Wang, D.; Liu, H. (2021) High-performance thin-layer chromatographic quantification of four active compounds in total flavonoids of *Ziziphora clinopodioides* Lam. and TLC–DPPH test for screening antioxidant components. *JPC-J Planar Chromat* 34, 481–492. doi.org/10.1007/s00764-021-00147-z

Zhang, L.G.; Yu, Z.Q.; Yang, C.; Chen, J.; Zhan, C.S.; Chen, X.G.; Zhang, L.; Hao, Z.Y.; Liang, C.Z. (2020). Effect of Eriocalyxin B on prostatic inflammation and pelvic pain in a mouse model of experimental autoimmune prostatitis. *The Prostate*, *80*(16), 1394–1404. doi.org/10.1002/pros.24065

Zhang, M.; Chen, M.; Hou, Y.; Fan, C.; Wei, H.; Shi, L.; Ma, G.; Zhang, J. (2021). Inflammatory and cytotoxic activities of Abietane terpenoids from *Nepeta bracteata* Benth.. *Molecules*, 26, 5603. https://doi.org/10.3390/molecules26185603.

Zhang, M.; Hwang, E.; Lin, P.; Gao, W.; Ngo, H.T.T.; Yi, T.H. (2018b). *Prunella vulgaris* L. Exerts a Protective Effect Against Extrinsic Aging Through NF-κB, MAPKs, AP-1, and TGF-β/Smad Signaling Pathways in UVB-Aged Normal Human Dermal Fibroblasts. *Rejuvenation Research*, *21*(4), 313–322. doi.org/10.1089/rej.2017.1971

Zhang, Q.Y.; Yan, B.C.; Hu, K.; Li, X.N.; Sun, H.D.; Puno, P.T. (2020b). Isorugosiformins A–F, six ent-kaurane diterpenoids from *Isodon rugosiformis*. *Fitoterapia*, *142*. doi.org/10.1016/j.fitote.2020.104529

Zhang, Q.; Porto, N.M.; Guilhon, C.C.; ...Fernandes, P.D.; Boylan, F. (2021). Pharmacognostic study on *Elsholtzia ciliata* (Thumb.) Hyl.: Anatomy, phytochemistry and pharmacological activities. *Pharmaceuticals*, 14(11), 1152.

Zhang, Y.Y.; Jiang, H.Y.; Liu, M.; Hu, K.; Wang, W.G.; Du, X.; Li, X.N.; Pu, J.X.; Sun, H.D. (2017). Bioactive ent-kaurane diterpenoids from *Isodon rubescens*. *Phytochemistry*, *143*, 199–207. doi.org/10.1016/j.phytochem.2017.08.009

Zhang, Y.; Liu, J.; Jia, W.; Zhao, A.; Zhang, T.L.; Liu, Y.; Jia, J.; Zhao, W.; Li, A. (2005). Distinct immunosuppressive effect by *Isodon serra* extracts. *International Immunopharmacology*, (Vol. 5). http://www.sciencedirect.com/science/article/pii/S1567576905001803

Zhang, Y.; Wang, K.; Chen, H.; He, R.; Cai, R.; Li, J.; Zhou, D.; Liu, W.; Huang, X.; Yang, R.; Deng, S.; Li, J.; Guan, X. (2018). Anti-inflammatory lignans and phenylethanoid glycosides from the root of *Isodon ternifolius* (D.Don) Kudô. *Phytochemistry*, *153*, 36–47. doi.org/10.1016/j.phytochem.2018.05.017

Zhao, J.; Xu, F.; He, J.H.; Gu, Z.Y.; Ma, L. (2013). [Study on chemical constituents of *Hyssopus cuspidatus*. Zhong yao cai = Zhongyaocai = *Journal of Chinese medicinal materials*, 36(1), pp. 54–57.

Zhao, J.; Xu, F.; Huang, H.; Ji, T.; Li, C.; Tan, W.; Chen, Y. (2015). Evaluation on bioactivities of total flavonoids from *Lavandula angustifolia*. *Pakistan Journal of Pharmaceutical Sciences*, *28*(4), 1245–1251. pubmed-ncbi-nlm-nih-gov.pbidi.unam.mx:2443/26142514/

Zhao, J.; Xu, L.; Jin, D.; Xin, Y.; Tian, L.;Wang, T.; Zhao, D.; Wang, Z.;Wang, J. 2022. Rosmarinic acid and related dietary supplements: potential applications in the prevention and treatment of Cancer. *Biomolecules,* 12, 1410. https://doi.org/10.3390/biom12101410

Zheng, S.; Lu, J.; Shen, T.; Liut, H.; Shen, X. (2001). New C-methylated flavones from *Elsholtzia blanda* Benth. *Indian Journal of Chemistry*, *408*(B), 232–234.

Zheng, X.Q.; Song, L.X.; Qiu, H.; Yang, Y.B.; Han, Z.Z.; Wang, Z.T.; Gu, L.H. (2022). Novel phenolic and diterpenoid compounds isolated from the fruit spikes of *Prunella vulgaris* L. and their anti-inflammatory activities. *Phytochemistry Letters*, *49*, 60–64. doi.org/10.1016/j.phytol.2022.03.005

Zhong, J.; Muhammad, N.; Yang, X.; Li, J. (2022). Antimicrobial, antioxidant, anti-inflammatory activities of eight essential oils obtained from traditional chinese medicines using supercritical fluid extraction coupled molecular distillation. *Journal of Essential Oil-Bear Plants* 25 (5) 1145 – 1158.

Zhong, J.; Muhammad, N.; Yang, X.; Li, J. (2022). Antimicrobial, antioxidant, anti-inflammatory activities of eight essential oils obtained from traditional Chinese medicines using supercritical fluid extraction coupled molecular distillation. *Journal of Essential Oil Bearing Plants*, 25:5, 1145-1158, doi: 10.1080/0972060X.2022.2142484.

Zhong, Y.; Huang, Y.; Santoso, M.B.; Wu, L.D. (2015). Sclareol exerts anti-osteoarthritic activities in interleukin-1β-induced rabbit chondrocytes and a rabbit osteoarthritis model. *International Journal of Clinical and Experimental Pathology* 8(3), 2365-2374.

Zhou, L.; Sun, L.; Wu, H.; Zhang, L.; Chen, M.; Liu, J.; Zhong, R. (2013). Oridonin ameliorates lupus-like symptoms of MRLlpr/lpr mice by inhibition of B-cell activating factor (BAFF). *European Journal of Pharmacology*, 715(1–3), 230–237. https://doi.org/10.1016/j.ejphar.2013.05.016

Zhu, C.-S.; Liu, K.; Wang, J.-L.; Li, J.-F.; Liu, M.-F.; Hao, N.; Lin, Y.-X.; Xiao, Z.-F. (2018). Antioxidant activities and hepatoprotective potential of *Dracocephalum rupestre* Hance extract against CCl4-induced hepatotoxicity in Kunming mice. *Journal of Food Biochemistry*, 42:e12484. https://doi.org/10.1111/jfbc.12484

Ziaei, A.; Hoppstädter, J.; Kiemer, A. K.; Ramezani, M.; Amirghofran, Z.; Diesel, B. (2015). Inhibitory effects of teuclatriol, a sesquiterpene from *Salvia mirzayanii*, on nuclear factor-κB activation and expression of inflammatory mediators. *Journal of Ethnopharmacology*, 160, 94–100. https://doi.org/10.1016/j.jep.2014.10.041

Zotsenko, L.; Kyslychenko, V.; Kalko, K.; Drogovoz, S. (2021). The study of phenolic composition and acute toxicity, anti-inflammatory and analgesic effects of dry extracts of some *Elsholtzia* genus (Lamiaceae) species. *Archives*, *2*, 637–649. httppharmacologyonline.silae.it

Zou, Y.H.; Zhao, L.; Xu, Y.K.; Bao, J.M.; Liu, X.; Zhang, J.S.; Li, W.; Ahmed, A.; Yin, S.; Tang, G.H. (2018). Anti-inflammatory sesquiterpenoids from the Traditional Chinese Medicine *Salvia plebeia*: Regulates pro-inflammatory mediators through inhibition of NF-κB and Erk1/2 signaling pathways in LPS-induced Raw 264.7 cells. *Journal of Ethnopharmacology*, *210*, 95–106. doi.org/10.1016/j.jep.2017.08.034

Zou, Z.Q.; Ning, D.S.; Liu, Y.; Fu, Y.X.; Li, L.C.; Pan, Z.H. (2022). Five new germacrane sesquiterpenes with anti-inflammatory activity from *Salvia petrophila*. *Phytochemistry Letters*, *47*, 111–114. doi.org/10.1016/j.phytol.2021.12.001

Zoubi, Y.E.; El, L.; Sidi, M.; Ben, M.; Boukhira, S.; Ben, S.M.; Farah, A. (2016). Phytochemical Screening, Anti-inflammatory Activity and Acute Toxicity of Hydro-ethanolic, Flavonoid, Tannin and Mucilage Extracts of *Lavandula stoechas* L. from Morocco Valorisation des plantes médicinales View project pharmacological activities of plants extracts View project. *Article in International Journal of Pharmacognosy and Phytochemical Research* 8(1), 31-37.www.researchgate.net/publication/287935765

Zuo, J.; Zhang, T.H.; Xiong, L.; Huang, L.; Peng, C.; Zhou, Q.M.; Dai, O. (2022). Two Pairs of 7,7′-Cyclolignan Enantiomers with Anti-Inflammatory Activities from *Perilla frutescens*. *Molecules*, 27(18). doi.org/10.3390/molecules27186102

Zuzarte, M.; Francisco, V.; Neves, B.; Liberal, J.; Cavaleiro, C.; Canhoto, J.; Salgueiro, L.; Cruz, M.T. (2022). *Lavandula viridis* L'Hér. Essential Oil Inhibits the Inflammatory Response in Macrophages Through Blockade of NF-KB Signaling Cascade. *Front. Pharmacol.* 12:695911. doi: 10.3389/fphar.2021.695911

Zuzarte, M.; Alves-Silva, J.M.; Alves, M.; ...Salgueiro, L.; Cruz, M.T. (2018). New insights on the anti-inflammatory potential and safety profile of *Thymus carnosus* and *Thymus camphoratus* essential oils and their main compounds. *Journal of Ethnopharmacology*, 2018, 225, pp. 10–17

Zuzarte, M.; Gonçalves, M.J.; Cavaleiro, C.; Cruz, M.T.; Benzarti, A.; Marongiu, B.; Maxia, A.; Piras, A.; Salgueiro, L. (2013). Antifungal and anti-inflammatory potential of *Lavandula stoechas* and *Thymus herbabarona* essential oils. *Industrial Crops and Products*, *44*, 97–103. doi.org/10.1016/j.indcrop.2012.11.002.

Zuzarte, M.; Sousa, C.; Cavaleiro, C.; Cruz, M.T.; Salgueiro, L. (2022). The Anti-Inflammatory Response of *Lavandula luisieri* and *Lavandula pedunculata.* Essential Oils. *Plants*, 2022, 11(3), 370.
